# Hypertension With High-Risk Features in Cryptogenic Stroke: An Exploratory Analysis of the ARCADIA Randomized Clinical Trial

**DOI:** 10.1001/jamaneurol.2026.0855

**Published:** 2026-04-20

**Authors:** Mohamed Ridha, Raed Hailat, Robert Stanton, Alexander E. Merkler, Mitchell S. V. Elkind, W. T. Longstreth, David L. Tirschwell, Richard A. Kronmal, Yousef Hannawi, Hooman Kamel, James Burke, Daniel Woo

**Affiliations:** 1 Department of Neurology, The Ohio State University, Columbus; 2 Department of Neurology, University of Cincinnati, Cincinnati, Ohio; 3 Department of Neurology, Weill Cornell Medical Center, New York, New York; 4 Department of Neurology, Vagelos College of Physicians and Surgeons, Columbia University, New York, New York; 5 Department of Epidemiology, Mailman School of Public Health, Columbia University, New York, New York; 6 Department of Neurology, University of Washington, Seattle; 7 Department of Epidemiology, University of Washington, Seattle; 8 Department of Biostatistics, University of Washington, Seattle; 9 Deputy Editor, *JAMA Neurology*; 10 Department of Neurology, State University of New York at Buffalo, Buffalo

## Abstract

**Importance:**

Multiple trials have found no difference in secondary stroke prevention between anticoagulation and antiplatelet therapy after cryptogenic stroke. Due to limitations of current stroke mechanism classification, one possible explanation is the failure to exclude patients with hypertension-related cerebrovascular disease.

**Objective:**

To determine whether hypertension with high-risk features is associated with treatment effect modification of anticoagulation vs antiplatelet therapy.

**Design, Setting, and Participants:**

This exploratory analysis of the Apixaban to Prevent Recurrence After Cryptogenic Stroke in Patients With Atrial Cardiopathy (ARCADIA) randomized clinical trial was conducted between April and August 2025. The original trial was conducted from February 2018 to February 2023 at 185 sites in North America. From 1015 randomized patients with a recent cryptogenic stroke and atrial cardiopathy, 945 with available hypertension data were included in this analysis after exclusions for missing blood pressure and echocardiography data.

**Interventions:**

Apixaban, 5 mg or 2.5 mg, twice daily vs aspirin, 81 mg, once daily.

**Main Outcomes and Measures:**

The primary outcome was recurrent ischemic stroke or systemic embolism. Hypertension with high-risk features was defined as systolic blood pressure ≥160 mm Hg at enrollment, left ventricular hypertrophy on echocardiography, or both. Cox proportional hazards models evaluated treatment interaction with hypertension with high-risk features and estimated hazard ratios within hypertension with high-risk features subgroups.

**Results:**

Among 945 patients (mean [SD] age, 68.0 [10.8] years; 513 [54.3%] female), 351 (37.1%) met criteria for hypertension with high-risk features. Over a median (IQR) follow-up of 1.6 (0.7-3.0) years within the analytic cohort, 67 patients experienced a recurrent ischemic stroke or systemic embolism. A significant interaction between hypertension with high-risk features and antithrombotic treatment was observed. In 594 patients without hypertension with high-risk features, apixaban was associated with lower risk compared to aspirin (hazard ratio [HR], 0.43; 95% CI, 0.22-0.85; annualized rate difference: −3.4%), whereas no significant association was observed in patients with hypertension with high-risk features (HR, 1.68; 95% CI, 0.78-3.62; annualized rate difference: 2.4%).

**Conclusions and Relevance:**

The findings in this study indicate that hypertension with high-risk features may be associated with modifications in the effect of antithrombotic treatment in patients with cryptogenic stroke. An unappreciated inclusion of strokes due to hypertensive arteriopathy may account for the lack of benefit with anticoagulation in prior trials of embolic stroke of undetermined source.

**Trial Registration:**

ClinicalTrials.gov Identifier: NCT03192215

## Key Points

**Question:**

Among patients with cryptogenic stroke, are high-risk features of hypertension associated with effect modification of anticoagulation compared to antiplatelet treatment for secondary prevention?

**Findings:**

In this exploratory analysis of a randomized clinical trial including 945 participants, high-risk features of hypertension were associated with effect modification of antithrombotic treatment assignment on recurrent ischemic stroke or systemic embolism. Anticoagulation was associated with fewer events among patients without high-risk features of hypertension, and no difference was observed between antithrombotic assignments among patients with high-risk features.

**Meaning:**

The findings suggest that hypertension risk stratification may identify a subgroup with cryptogenic stroke who are likely to benefit from anticoagulation treatment.

## Teaser

This secondary analysis of a randomized clinical trial evaluates the association between hypertension with high-risk features and treatment effect.

## Introduction

Antithrombotic selection for secondary prevention after ischemic stroke depends on the presumed mechanistic cause.^1^ Despite extensive diagnostic testing, 25% to 30% of ischemic strokes are cryptogenic.^2^ Cryptogenic strokes likely comprise multiple distinct mechanisms, including occult cardioembolic sources.^3^ Since anticoagulation is effective in stroke prevention for definite cardioembolic sources, it has been hypothesized that anticoagulation may be similarly effective after an embolic stroke of undetermined source. However, trials have not demonstrated superiority of anticoagulation compared to antiplatelet therapy in embolic stroke of undetermined source.^4,5,6^

The theoretical rationale for using anticoagulation in embolic stroke of undetermined source relies on the exclusion of patients with alternative, noncardioembolic stroke mechanisms.^7^ One plausible explanation for neutral trial results is a limitation of current stroke mechanism subtyping to accurately identify the entire spectrum of hypertension-related cerebrovascular disease. Inclusion of patients with severe hypertension may attenuate a benefit of anticoagulation if a substantial proportion of recurrent ischemic events are driven by noncardioembolic mechanisms, such as hypertensive arteriopathy. We therefore hypothesized that hypertension risk stratification may identify heterogeneity of antithrombotic response in patients with cryptogenic stroke. Using data from the Apixaban to Prevent Recurrence After Cryptogenic Stroke in Patients With Atrial Cardiopathy (ARCADIA) trial, we examined whether the effect of anticoagulation compared to antiplatelet therapy was modified by the presence of hypertension with high-risk features.

## Methods

### Design

ARCADIA was a multicenter, randomized, phase 3 clinical trial comparing the effect of apixaban vs aspirin on stroke recurrence in patients with a recent cryptogenic stroke and atrial cardiopathy. The trial was conducted in 185 sites throughout North America from February 1, 2018, to February 28, 2023. Regional institutional review boards approved the protocol, and all participants provided written, informed consent. The trial protocol (Supplement 1) and statistical analysis plan did not prespecify the present analysis.^8^ This analysis was conducted between April 6 to August 31, 2025. Results were reported in accordance with the Consolidated Standards of Reporting Trials (CONSORT) reporting guideline.^9^

### Population

Eligible participants were at least 45 years old, experienced a cryptogenic ischemic stroke attributed to embolic stroke of undetermined source within 180 days, and had evidence of atrial cardiopathy. Atrial cardiopathy was defined as 1 or more of the following: (1) P-wave terminal force in lead V_1_ greater than 5000 μV · ms, (2) serum N-terminal pro-B-type natriuretic peptide greater than 250 pg/mL, and (3) a left atrial diameter index of 3.0 cm/m^2^ or greater on echocardiography.

A cryptogenic stroke was designated as embolic stroke of undetermined source using consensus criteria that required brain imaging (computed tomography or magnetic resonance imaging [MRI]), cervical and intracranial vascular imaging, echocardiography, electrocardiography, and at minimum 24-hour of continuous cardiac monitoring. Additional evaluations were performed at the discretion of the treating physician. Patients with a known cardiac source of embolism, 50% or greater stenosis of a parent intra- or extracranial vessel, lacunar stroke, or another established stroke mechanism were excluded from enrollment. Lacunar stroke was defined as a single, subcortical infarct in the distribution of a small, penetrating vessel. The infarct diameter could be no larger than 1.5 cm on computed tomography, 1.5 cm on T2 MRI imaging, or 2 cm on MRI diffusion imaging. Patients with a typical lacunar syndrome without evidence of infarct on imaging were excluded. Those with multiple, simultaneous small subcortical infarcts, lateral medullary infarcts, and/or cerebellar infarcts were eligible for enrollment. Sites recorded the infarct pattern from the qualifying stroke as cerebral cortex, large deep, small deep, or cerebellar cortex. The vascular territory of the qualifying stroke was recorded as left anterior, right anterior, posterior circulation, or a combination.

Patients with uncontrolled hypertension at the time of recruitment were eligible for enrollment but randomization was delayed until at least 2 weeks after the index stroke. Other major exclusionary criteria for the study were prior atrial fibrillation, ejection fraction less than 30%, an alternative indication for either anticoagulation or antiplatelet therapy, history of intracranial hemorrhage, creatinine of 2.5 mg/dL or greater, clinically important anemia, bleeding diathesis, or severe disability (Modified Rankin Scale score of 5). Complete inclusion and exclusion criteria are detailed within the study protocol.^8^ Self-reported race and ethnicity were collected at enrollment interview to account for potential differences in hypertension-related risk.

### Hypertension Risk Stratification

Using a similar framework for hypertension risk stratification proposed in the 2023 European Society of Hypertension guidelines, the primary definition of hypertension with high-risk features was a systolic blood pressure (SBP) measurement of 160 mm Hg or greater or cardiac evidence of hypertension-mediated organ damage.^10^ Baseline SBP measurements were collected by trial personnel at the time of recruitment. Cardiac hypertension-mediated organ damage was defined as left ventricular hypertrophy on echocardiography, using sex-specific left ventricular mass index cutoffs (men: >115 g/m^2^; women: >95 g/m^2^).^11^ Left ventricular mass index measurements were centrally adjudicated by the echocardiography core lab using measured septal thickness, posterior wall thickness, left ventricular end-diastolic diameter, and body surface area.

Impaired kidney function is another manifestation of hypertension-mediated organ damage and was assessed as an alternative feature.^10,12^ Kidney hypertension-mediated organ damage was defined as estimated glomerular filtration rate (eGFR) less than 60 mL/min/1.73 m^2^. The 2021 Chronic Kidney Disease Epidemiology Collaboration (race-free) formula was used to calculate baseline eGFR.^13^ Secondary definitions of hypertension with high-risk features were specified as (1) SBP 160 mm Hg or greater or eGFRless than 60 mL/min/1.73 m^2^; (2) SBP 160 mm Hg or greater, left ventricular hypertrophy, eGFR less than 60 mL/min/1.73 m^2^, or any combination of these. Primary and secondary definitions of hypertension with high-risk features are shown in eTable 1 in Supplement 2.

### Intervention

Patients were randomly assigned to treatment with apixaban or aspirin, 81 mg, daily in a 1:1 ratio between 3 and 180 days after the qualifying stroke. Apixaban was administered as 5 mg twice daily unless at least 2 criteria were met for dose reduction to 2.5 mg twice daily (age ≥80 years, creatinine ≥1.5 mg/dL, or weight ≤60 kg). Patients, investigators, and medical providers were blinded to treatment assignments.

### Outcomes

The primary outcome for this analysis was the composite of ischemic stroke or systemic embolism (a prespecified secondary end point of the original trial design). Two secondary outcomes were assessed: (1) recurrent ischemic stroke and (2) any recurrent stroke (ischemic, hemorrhagic, or undetermined). Outcomes in the trial were adjudicated by 2 vascular neurologists blinded to treatment status.

### Statistical Analysis

Baseline characteristics were compared using *t* test for continuous variables and χ^2^ or Fisher exact test for categorical variables. Cox proportional hazards models were used to evaluate whether hypertension with high-risk features modified the treatment effect of apixaban compared to aspirin. Crude (adjusted for treatment randomization arm, presence of hypertension with high-risk features, and interaction between treatment and hypertension with high-risk features) and fully adjusted models (additional adjustment for CHA_2_DS_2_VASc score and Black race) were fit. A sensitivity analysis replaced CHA_2_DS_2_VASc with individual vascular risk factors associated with the primary outcome (diabetes and prior ischemic stroke/transient ischemic attack). Proportional hazards assumptions were confirmed using the Schoenfeld residual test. The analysis was performed according to randomized treatment assignments. Cases missing baseline SBP measurement and left ventricular mass index measurements were excluded. Censoring occurred at the time of adjudicated outcome, study completion, or last follow-up. Significant interactions were explored with stratum-specific hazard ratios estimated from fully adjusted interaction models, and cumulative event rate curves were generated for visualization of time-to-event differences. Secondary outcomes were assessed using identical models. Incidence rates and exact Poisson 95% confidence intervals were calculated for primary and secondary outcomes within each treatment arm.

Five sensitivity analyses used modified definitions of hypertension with high-risk features: (1) SBP 160 mm Hg or greater and secondary definitions of hypertension-mediated organ damage, (2) number of high-risk features, (3) left ventricular hypertrophy and alternative thresholds of SBP of 140 mm Hg or greater or 150 mm Hg or greater, (4) only SBP ranges (<140 mm Hg,140-149 mm Hg,150-159 mm Hg, or ≥160 mm Hg), and (5) only presence of hypertension-mediated organ damage. A sensitivity analysis was conducted to assess if hypertension with high-risk features modified treatment effect in index infarcts that do not share radiographic characteristics of lacunes (small, deep infarct in a single vascular territory). An additional analysis was performed using the per-protocol cohort (only patients who met protocol eligibility criteria and started the study drug). All sensitivity analyses were adjusted for CHA_2_DS_2_VASc score and race.

Statistical significance was defined with 2-sided *P* value <.05. Given the post hoc, exploratory nature of these analyses, *P* values <.05 were interpreted as nominal evidence of association rather than confirmatory statistical significance. Analyses were performed using SPSS version 29.0 (SPSS) and Stata version 19 (StataCorp).

## Results

### Characteristics

**Figure 1.**
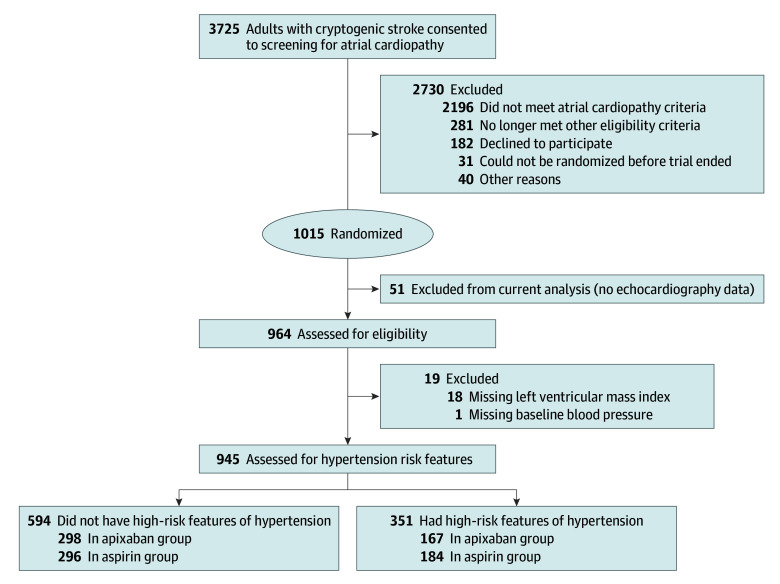
Participant Flow Diagram

Of 1015 randomized patients, 945 had complete SBP and echocardiography data (Figure 1). An additional 10 patients were excluded from CHA_2_DS_2_VASc-adjusted models due to missing score components. The mean (SD) age was 68.0 (10.8) years; 513 (54.3%) were female and 432 (45.7%) were male. Of 931 participants with known race, 201 (21.3%) were Black, 703 (74.4%) were White, and 27 (2.9%) were of another race (including Alaska Native, Asian, Native American, and Pacific Islander, consolidated because of small sample sizes). Of 940 with known ethnicity, 80 (8.5%) were Hispanic and 860 (91.5%) were non-Hispanic. Evidence of hypertension with high-risk features was present in 351 (37.1%) patients. Hypertension with high-risk features was identified by left ventricular hypertrophy alone in 210 (59.8%), SBP 160 mm Hg or greater alone in 88 (25.1%), and both in 53 (15.1%) patients. Apixaban was assigned to 298 patients without hypertension with high-risk features (50.2%) and 167 patients with hypertension with high-risk features (47.6%) (*P* = .44) (Table 1). Baseline characteristics were balanced between treatment arms within hypertension with high-risk features strata (eTable 2 in Supplement 2). Concordance between SBP and hypertension-mediated organ damage features is shown in eTable 3 in Supplement 2.

**Table 1.** Baseline Characteristics of Patients With and Without High-Risk Features of Hypertension

Characteristic	No. (%)		*P* value
	No hypertension with high-risk features (n = 594)^a^	Hypertension with high-risk features (n = 351)^a^	
Age, mean (SD), y	68.3 (10.7)	67.4 (11.0)	.20
Sex			
Female	307 (51.7)	206 (58.7)	.04
Male	287 (48.3)	145 (41.3)	
Race^b^			
Black	95 (16.0)	106 (30.2)	<.001
White	473 (79.6)	230 (65.5)	
Other^c^	22 (3.7)	5 (1.4)	
Unknown	4 (0.7)	10 (2.8)	
Ethnicity^b^			
Hispanic	48 (8.1)	32 (9.1)	.88
Non-Hispanic	543 (91.4)	317 (90.3)	
Unknown	3 (0.5)	2 (0.6)	
Medical comorbidities			
Coronary artery disease	54 (9.2)	42 (12.0)	.16
Heart failure	21 (3.6)	44 (12.5)	<.001
Diabetes	170 (28.7)	123 (35.1)	.04
Hypertension	431 (72.8)	298 (85.1)	<.001
Peripheral vascular disease	8 (1.4)	8 (2.3)	.31
Obstructive sleep apnea	77 (13.8)	47 (14.1)	.90
Prior stroke or TIA	101 (17.0)	74 (21.1)	.12
Cancer	87 (14.7)	41 (11.7)	.20
Tobacco use			
Former	159 (26.8)	81 (23.1)	.14
Current	90 (15.2)	69 (19.7)	
BMI, mean (SD)	29.5 (6.6)	30.4 (6.9)	.04
CHA_2_DS_2_VASc score, mean (SD)^d^	2.9 (1.6)	3.3 (1.6)	<.001
NIHSS, mean (SD)^e^	2.2 (3.5)	2.2 (3.4)	.97
Time from qualifying stroke to enrollment, mean (SD), d	39.9 (45.1)	35.1 (42.3)	.11
Time from BP measurement to enrollment, mean (SD), d	2.1 (13.8)	4.2 (19.9)	.08
SBP, mean (SD), mm Hg	131.7 (15.5)	149.8 (23.7)	<.001
DBP, mean (SD), mm Hg	75.6 (11.8)	80.6 (15.0)	<.001
eGFR, mean (SD), mL/min/1.73 m^2^	78.5 (18.9)	73.9 (21.3)	<.001
Atrial cardiopathy biomarkers			
PTFV_1_, mean (SD), μV × ms	4751.4 (2510.4)	4807.4 (3042.3)	.77
NT-proBNP, median (IQR), pg/mL	265.1 (74.2-455.5)	401.6 (212.8-799.4)	<.001
LAD index, mean (SD), cm/m^2^	1.8 (0.5)	2.0 (0.4)	<.001
Type of echocardiography			
Transthoracic	520 (87.5)	308 (87.7)	.17
Transesophageal	0 (0.0)	2 (0.6)	
Both	74 (12.5)	41 (11.7)	
LV wall measurements			
Posterior wall thickness, mean (SD), cm	1.0 (0.2)	1.2 (0.3)	<.001
Septal thickness, mean (SD), cm	1.0 (0.2)	1.3 (0.3)	<.001
End diastolic diameter, mean (SD), cm	4.5 (0.6)	4.7 (0.7)	<.001
LV mass, mean (SD), g	152.2 (42.1)	229.9 (74.7)	<.001
LV mass index, mean (SD), g/m^2^	77.4 (16.7)	118.5 (35.1)	<.001
Relative wall thickness, mean (SD)	0.44 (0.13)	0.53 (0.16)	<.001
Ejection fraction, mean (SD), %	61.2 (6.5)	58.9 (9.2)	<.001
Infarct pattern			
Cerebral cortex	386 (65.0)	226 (64.4)	.85
Cerebellar cortex	69 (11.6)	44 (12.5)	.67
Deep and small	128 (21.5)	83 (23.6)	.45
Deep and large	65 (10.9)	43 (12.3)	.54
Multiple	99 (16.7)	70 (19.9)	.20
Randomized treatment arm			
Apixaban	298 (50.2)	167 (47.6)	.44
Aspirin	296 (49.8)	184 (52.4)	

Abbreviations: BMI, body mass index (calculated as weight in kilograms divided by height in meters squared); DBP, diastolic blood pressure; eGFR, estimated glomerular filtration rate; LAD, left atrial diameter; LV, left ventricle; LVH, left ventricular hypertrophy; NIHSS, National Institute of Health Stroke scale; NT-proBNP, N-terminal pro-B-type natriuretic peptide; PTFV_1_, P-wave terminal force in lead V_1_; SBP, systolic blood pressure; TIA, transient ischemic attack.

^a^ Hypertension with high-risk features defined as systolic blood pressure ≥160 mm Hg or left ventricular hypertrophy.

^b^ Self-reported race were collected at enrollment interview to account for potential differences in hypertension-related risk.

^c^ Other race and ethnicity groups included including Alaska Native, Asian, Native American, and Pacific Islander, consolidated because of small sample sizes.

^d^ CHA2DS2VASc range, 0-9, with higher scores indicating greater risk.

^e^ NIHSS range, 0-42, with higher scores indicating greater neurologic deficit.

### Primary Outcome

Over a median (IQR) follow-up period of 1.6 (0.7-3.0) years, 67 (7.1%) patients experienced a recurrent ischemic stroke or systemic embolism. Among patients without hypertension with high-risk features, the incidence rate (per 1000 person-years) of the primary outcome was lower in those randomized to apixaban compared with aspirin (apixaban: 21.5; 95% CI, 11.8-36.2; 12 events vs aspirin: 55.1; 95% CI, 37.7-77.5, 28 events). Among patients with hypertension with high-risk features, the incidence rate was higher in patients randomized to apixaban compared with aspirin (apixaban: 55.8; 95% CI, 33.6-87.0; 16 events vs aspirin: 31.8; 95% CI, 17.0-54.4; 11 events).

**Figure 2.**
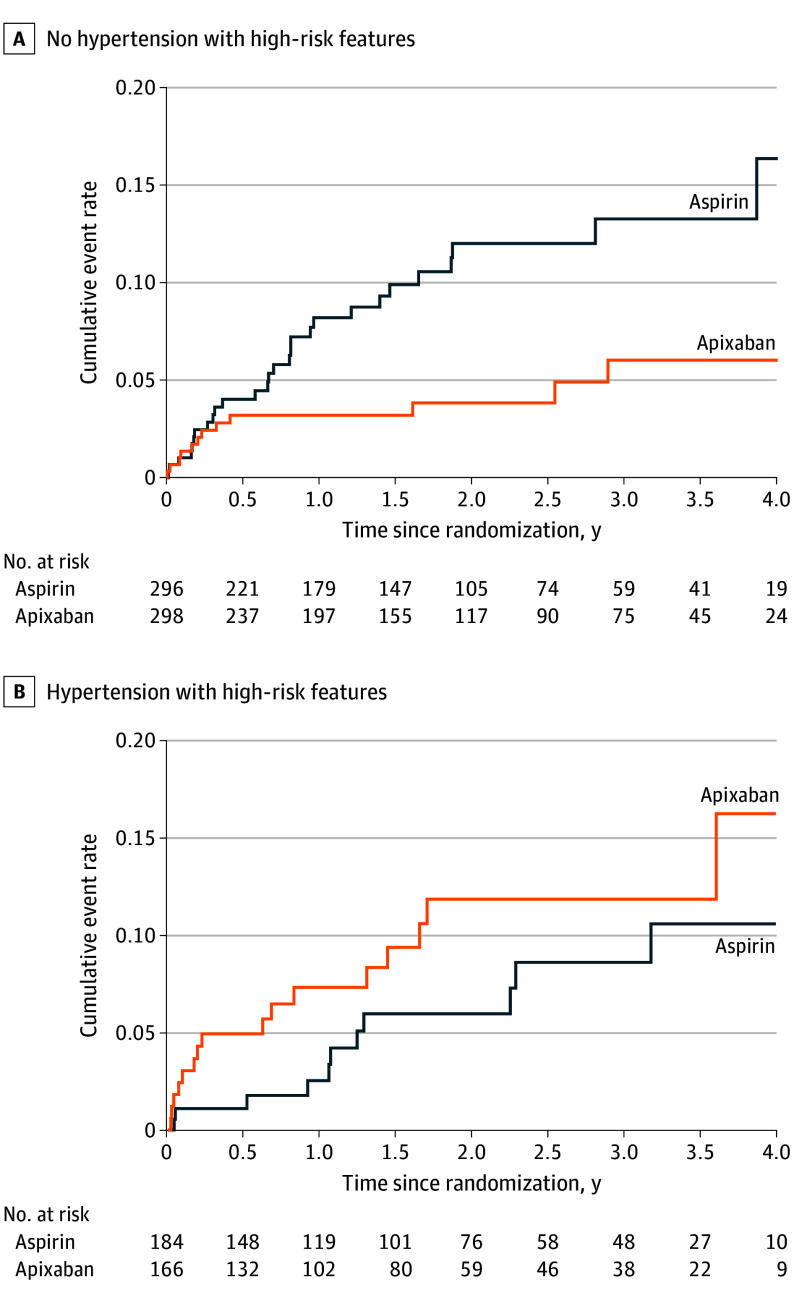
Cumulative Event Curves for Recurrent Ischemic Stroke or Systemic Embolism in Patients With and Without High-Risk Features of Hypertension

The primary adjusted model demonstrated a significant interaction between treatment group and hypertension with high-risk features (interaction hazard ratio [HR], 3.87; 95% CI, 1.39-10.82; *P* = .01) (Table 2). Stratum-specific HRs for the effect of apixaban vs aspirin were derived from the interaction model and represent conditional treatment effects evaluated at each reference category of hypertension with high-risk features. Apixaban was associated with a significantly lower risk of ischemic stroke or systemic embolism compared to aspirin among patients without hypertension with high-risk features (HR, 0.43; 95% CI, 0.22-0.85; annualized rate difference, −3.4%). No significant association was found among patients with hypertension with high-risk features (HR, 1.68; 95% CI, 0.78-3.62; annualized rate difference, 2.4%) (Table 3). Inferences from the crude model and model with adjustment for individual risk factors were similar (eTable 4 in Supplement 2). Cumulative event rate curves are shown in Figure 2.

**Table 2.** Conditional Hazard Ratios for the Interaction Between Hypertension With High-Risk Features and Antithrombotic Treatment on Recurrent Ischemic Stroke or Systemic Embolism^a^

Model^b^	HR (95% CI)	*P* value
**Primary hypertension with high-risk features (SBP ≥160 mm Hg or LVH)**		
Apixaban vs aspirin	0.43 (0.22-0.85)	.02
Hypertension with high-risk features	0.51 (0.25-1.03)	.06
Apixaban × hypertension with high-risk features	3.87 (1.39-10.82)	.01
**Secondary hypertension with high-risk features (definition 1: SBP ≥160 mm Hg or eGFR <60 ml/min/1.73 m^2^**)		
Apixaban vs aspirin	0.51 (0.28-0.93)	.03
Hypertension with high-risk features	0.36 (0.16-0.82)	.02
Apixaban × hypertension with high-risk features	3.86 (1.27-11.74)	.02
**Secondary hypertension with high-risk features (definition 2: SBP ≥160 mm Hg, LVH, eGFR <60 ml/min/1.73 m**^2^, **or any combination)**		
Apixaban vs aspirin	0.37 (0.17-0.79)	.01
Hypertension with high-risk features	0.50 (0.26-0.96)	.04
Apixaban × hypertension with high-risk features	3.96 (1.42-11.03)	.009
**No. of high-risk features^c^**		
Apixaban vs aspirin	0.43 (0.22-0.82)	.01
No. of features	0.53 (0.32-0.89)	.02
Apixaban × No. of features	2.44 (1.29-4.62)	.006

Abbreviations: eGFR, estimated glomerular filtration rate; HR, hazard ratio; LVH, left ventricular hypertrophy; SBP, systolic blood pressure.

^a^ Interpretation of the main effects is conditional on the reference categories specified in the interaction model. The hazard ratio for apixaban vs aspirin represents the treatment effect among patients without high-risk features of hypertension, while the hazard ratio for hypertension with high-risk features represents the association among patients assigned to aspirin. The interaction hazard ratio reflects effect modification, quantifying the relative difference in the apixaban vs aspirin effect between patients with and without high-risk hypertension features.

^b^ All models adjusted for CHA_2_DS_2_VASc score and Black race.

^c^ Cumulative number of high-risk hypertension features identified (systolic blood pressure ≥160 mm Hg, estimated glomerular filtration rate <60 ml/min/1.73 m^2^).

**Table 3.** Stratum-Specific Hazard Ratios for Primary and Secondary Outcomes

Outcome	Apixaban vs aspirin, HR (95% CI)^a^
**Recurrent ischemic stroke or systemic embolism** ^b^ ^,^ ^c^	
No hypertension with high-risk features	0.43 (0.22-0.85)
Hypertension with high-risk features	1.68 (0.78-3.62)
**Recurrent ischemic stroke** ^b^ ^,^ ^c^	
No hypertension with high-risk features	0.47 (0.24-0.93)
Hypertension with high-risk features	1.85 (0.84-4.08)
**Stroke of any type** ^b^ ^,^ ^c^ ^,^ ^d^	
No hypertension with high-risk features	0.53 (0.28-1.00)
Hypertension with high-risk features	1.64 (0.78-3.43)

^a^ Hazard ratios (HRs) are derived from fully adjusted interaction models and represent conditional treatment effects of apixaban vs aspirin evaluated within each stratum of hypertension with high-risk features.

^b^ Stratum-specific hazard ratios estimated from interaction models adjusted for CHA_2_DS_2_VASc score and Black race.

^c^ Hypertension with high-risk features defined as systolic blood pressure ≥160 mm Hg or left ventricular hypertrophy.

^d^ Ischemic, hemorrhagic, and undetermined subtypes.

### Alternative Definitions for Hypertension With High-Risk Features

Among 944 patients with available eGFR, 299 (31.7%) met criteria for hypertension with high-risk features based on renal evidence of hypertension-mediated organ damage (SBP ≥160 mm Hg or eGFR <60 mL/min/1.73 m^2^). Using kidney hypertension-mediated organ damage to define hypertension with high-risk features, interaction between treatment arm and hypertension with high-risk features status was observed (interaction HR, 3.86; 95% CI, 1.27-11.74; *P* = .02) (Table 2). Stratum-specific estimates demonstrated that apixaban was associated with a lower risk of recurrent ischemic stroke and systemic embolism among patients without HWRF (HR, 0.51; 95% CI, 0.28-0.93), whereas no association was seen in patients with hypertension with high-risk features (HR, 0.76; 95% CI, 0.47-1.24).

When hypertension with high-risk features was defined using SBP and either form of hypertension-mediated organ damage (SBP ≥160 mm Hg, left ventricular hypertrophy, eGFR <60 mL/min/1.73 m^2^, or any combination), 456 patients (48.3%) met criteria for hypertension with high-risk features. Using either hypertension-mediated organ damage feature to define hypertension with high-risk features, a consistent interaction between treatment arm and hypertension with high-risk features definition was detected (interaction HR, 3.96; 95% CI, 1.42-11.03; *P* = .009) (Table 2). Inferences from stratum-specific estimates were similar; apixaban was associated with lower risk among patients without hypertension with high-risk features (HR, 0.37; 95% CI, 0.17-0.79) but not among those with hypertension with high-risk features (HR, 1.45; 95% CI, 0.74-2.86).

The cumulative number of high-risk features modified the effect of treatment assignment (interaction HR, 2.44; 95% CI, 1.29-4.62; *P* = .006) (Table 2). Increased high-risk feature burden altered directionality of the association between apixaban compared to aspirin and recurrent ischemic stroke or systemic embolism (0 features: HR, 0.37; 95% CI, 0.17-0.79; 1 feature: HR, 1.12; 95% CI, 0.51-2.43; >1 features: HR, 3.57; 95% CI, 0.74-17.2).

### Secondary Outcomes

A total of 70 strokes of any type (ischemic, hemorrhagic, and undetermined) with 64 ischemic strokes were adjudicated over the follow-up period. In patients without hypertension with high-risk features, the incidence rates of recurrent ischemic stroke and any stroke were lower in patients randomized to apixaban (ischemic stroke: 21.5; 95% CI, 11.8-36.2; any stroke: 25.1; 95% CI, 14.5-40.7) compared to aspirin (ischemic stroke: 51.0; 95% CI, 34.4-72.7; any stroke: 53.0; 95% CI, 36.0-75.0). In patients with hypertension with high-risk features, the incidence rates of recurrent ischemic stroke and any stroke were higher in those assigned to apixaban (ischemic stroke: 55.8; 95% CI, 33.6-87.0; any stroke: 59.3; 95% CI, 36.3-91.2) compared to aspirin (ischemic stroke: 28.9; 95% CI, 14.9-50.6; any stroke: 34.6; 95% CI, 19.1-57.9).

A significant interaction between hypertension with high-risk features and treatment was observed for recurrent ischemic stroke (interaction HR, 3.97; 95% CI, 1.39-11.33; *P* = .01) and any stroke (interaction HR, 3.11; 95% CI, 1.16-8.34; *P* = .02) (eTable 5 in Supplement 2). Strata-specific estimates for patients without hypertension with high-risk features demonstrated that apixaban was associated with reduced risk of recurrent ischemic stroke but not any stroke (ischemic stroke: HR, 0.47; 95% CI, 0.24-0.93; any stroke: HR, 0.53; 95% CI, 0.28-1.00). Strata-specific estimates for patients with hypertension with high-risk features found no association between apixaban assignment with either outcome (ischemic stroke: HR, 1.85; 95% CI, 0.84-4.08; any stroke: HR, 1.64; 95% CI, 0.78-3.43) (Table 3). Cumulative event rate curves of the secondary outcomes are shown in eFigures 1-2 in Supplement 2.

### Sensitivity Analyses

Across alternative SBP thresholds to define hypertension with high-risk features, the magnitude of the interaction between treatment and hypertension with high-risk features varied with the largest estimate observed at a threshold of 160 mm Hg or greater. Using only evidence of hypertension-mediated organ damage to define hypertension with high-risk features, the interaction estimate was consistent with the primary analysis. Model estimates using SBP ranges alone to define hypertension risk did not demonstrate treatment effect modification (eTables 6-7 in Supplement 2).

A total of 80 patients (8.5%) had an index infarct classified as exclusively small and deep in a single vascular territory. Exclusion of these patients yielded similar estimates for treatment and interaction terms. Analysis of the per-protocol cohort demonstrated similar results (eTable 7 in Supplement 2).

## Discussion

In this exploratory analysis of the ARCADIA trial, we found a substantial proportion of patients with cryptogenic stroke and atrial cardiopathy had hypertension with high-risk features, and the presence of hypertension with high-risk features modified the association between antithrombotic strategy and recurrent ischemic events. The results suggest a potential benefit of anticoagulation for secondary prevention among patients without high-risk features of hypertension.

One possible explanation for the neutral results of the ARCADIA trial is the incomplete exclusion of strokes predominantly driven by hypertension. For a subgroup of patients, atrial cardiopathy may represent an epiphenomenon where the index or recurrent strokes are due to hypertension-mediated mechanisms, including hypertensive arteriopathy. An MRI substudy of the ARCADIA trial found 40.7% (22/54) of subsequent covert or clinical ischemic strokes were lacunar.^14^ Stratification of hypertension severity may therefore improve identification of patients with strokes due to hypertensive arteriopathy, in whom anticoagulation would not be expected to confer benefit. Although the results of this post hoc analysis should be considered exploratory, they highlight both the potential benefit of anticoagulation in this select group of patients with cryptogenic stroke as well as shortcomings in the current conceptual framework of stroke mechanism classification.

Within the nosologic construct of embolic stroke of undetermined source the lacunar criteria is used to exclude ischemic strokes due to hypertensive small vessel disease.^7^ However, the modern criteria are largely derived from historic clinicopathologic descriptions and may inadequately capture the entire spectrum of hypertensive arteriopathy.^15^ Population-based studies with standardized stroke subtyping have demonstrated an inconsistent and small excess risk of hypertension in lacunar strokes, suggesting the lacunar definition poorly differentiates strokes due to hypertensive arteriopathy.^16,17,18,19^ While infarcts related to hypertensive arteriopathy may have preferentially present as small and subcortical, they may not be restricted to this single phenotype. Advances in neuroimaging support an expanded spectrum of cerebral small vessel disease, which may account for a meaningful proportion of cryptogenic stroke.^20,21^ Hypertension is also implicated in the pathogenesis of atherosclerotic disease and contributes a similar attributable risk fraction as lacunar stroke.^22^ Although the ARCADIA trial excluded patients with intracranial or extracranial atherosclerosis causing at least 50% stenosis, other mechanisms such as artery-artery embolism or branched atheromatous disease may be implicated.^23^

The integration of hypertension risk stratification into current stroke mechanism classification criteria may improve patient selection for future secondary prevention trials. Premorbid diagnosis of hypertension is prevalent in patients with cryptogenic strokes, but significant variability may exist in hypertension duration, control, antihypertensive medication use, and end-organ damage. In this study, most patients without hypertension with high-risk features had a history of hypertension, demonstrating hypertension risk stratification may better distinguish a differential impact of hypertension on stroke treatment. Although BP measurement is the traditional approach to measure hypertension severity, a substantial proportion have unrecognized hypertension when undergoing ambulatory monitoring.^24^ Furthermore, BP may fluctuate in the acute setting after an ischemic stroke, and antihypertensive treatment is frequently initiated during hospitalization.^25^ In ARCADIA, 79.8% of patients with left ventricular hypertrophy did not have SBP 160 mm Hg or greater, highlighting the potential misclassification of hypertension with high-risk features with sole reliance on isolated BP measurements. Evidence of hypertension-mediated organ damage improves identification of clinically important hypertensive disease and is independently associated with cardiovascular events.^26,27^ Features of hypertension-mediated organ damage specific to the cerebrovasculature, such as cerebral microbleeds, white matter hyperintensity, or retinal vascular changes, may further refine risk stratification in future studies.^10^

Strengths of this analysis include a well-characterized cohort of patients with ischemic strokes that meet the criteria for embolic stroke of undetermined source. Strokes meeting the conventional criteria for lacunar were specifically excluded. Brain MRI was performed in most of the cohort to ensure accurate assessment of infarct size and pattern. Moreover, this is one of the few cryptogenic stroke cohorts with detailed adjudication of left ventricular wall measurements. As this was a large randomized trial, covariate imbalances between treatment groups were minimal, and adjustment for potential confounders yielded similar results.

### Limitations

Several limitations are notable. This was a post hoc, exploratory analysis of a trial not designed to evaluate effect modification by hypertensive disease, and the number of outcome events was modest. Enrollment was limited to patients with atrial cardiopathy, and findings may not be generalizable to the entire embolic stroke of undetermined source population. Left ventricular hypertrophy and kidney impairment can be caused by conditions other than hypertension, and escalation of antihypertensive treatment after stroke may have resulted in lower BP measurements at the time of enrollment. However, the use of multiple modalities to assess hypertension risk and the consistent association with various definitions of high-risk features mitigate this concern.

## Conclusions

Cryptogenic stroke represents a heterogeneous clinical entity encompassing various underlying mechanisms. Anticoagulation may be superior to antiplatelet therapy in patients without high-risk features of hypertension. Investigators should reconsider patient selection for stroke prevention trials given the potential limitations of current subtype classification to capture the spectrum of hypertension-related cerebrovascular disease.

## Notes

Supplement 1. Trial protocol

Supplement 2. eTable 1. Hypertension With High-Risk Features Definitions eTable 2. Baseline Characteristics Between Treatment Arms Stratified by Hypertension With High-Risk Features eTable 3. Systolic Blood Pressure by Hypertension-Mediated Organ Damage Definitions eTable 4. Crude and Alternatively Adjusted Interaction Models of Recurrent Ischemic Stroke or Systemic Embolism eTable 5. Fully Adjusted Interaction Models of Secondary Outcomes eTable 6. Interaction Models Using Alternative Systolic Blood Pressure Thresholds of Hypertension With High-Risk Features eTable 7. Additional Sensitivity Analyses eFigure 1. Cumulative Event Rates for Recurrent Ischemic Stroke In Patients With and Without High-Risk Features of Hypertension eFigure 2. Cumulative Event Rates for Stroke of Any Type In Patients With and Without High-Risk Features of Hypertension

Supplement 3. Data sharing statement
