# Supplementary material for: Hypertension With High-Risk Features in Cryptogenic Stroke: An Exploratory Analysis of the ARCADIA Randomized Clinical Trial
Source: JAMA Neurol. 2026 Apr 20;83(6):573–81. doi: 10.1001/jamaneurol.2026.0855 (PMC13097029; doi:10.1001/jamaneurol.2026.0855)
Supplement: Supplement 1. — Trial protocol [file jamaneurol-e260855-s001.pdf]

Protocol and Statistical Analysis Plan for the ARCADIA Randomized Clinical Trial

This supplement includes:

1. Final trial protocol (v6, dated 17 Aug 2022)
2. Log of changes to the protocol
3. Protocol in effect when trial enrollment began (v3, dated 1 Dec 2017)
4. Final statistical analysis plan (v1.4, dated 15 Feb 2023)
5. Log of changes to the statistical analysis plan
6. Statistical analysis plan in effect when trial enrollment began (v1.1, dated 8 Jan 2018)

ARCADIA

AtRial Cardiopathy and Antithrombotic Drugs In prevention After cryptogenic stroke

Protocol Identifying Number: NCT03192215

Principal Investigators:
Hooman Kamel, MD
Mitchell S.V. Elkind, MD, MS
W.T. Longstreth Jr, MD, MPH
David L. Tirschwell, MD, MSc
Randolph S. Marshall, MD

Funded by: NINDS
Supported by: BMS-Pfizer Partnership and Roche Diagnostics

Version Number: v6

17 Aug 2022

Table of Contents

LIST OF ABBREVIATIONS 5
STATEMENT OF COMPLIANCE 7
PROTOCOL SUMMARY 9
SCHEMATIC OF STUDY DESIGN 10
1 KEY ROLES 11
2 INTRODUCTION: BACKGROUND AND SCIENTIFIC RATIONALE 12
2.1 BACKGROUND 12
2.2 RATIONALE 13
2.3 POTENTIAL RISKS AND BENEFITS 13
2.3.1 KNOWN POTENTIAL RISKS 13
2.3.2 KNOWN POTENTIAL BENEFITS 14
3 OBJECTIVES AND PURPOSE 14
4 STUDY DESIGN AND ENDPOINTS 15
4.1 DESCRIPTION OF THE STUDY DESIGN 15
4.2 STUDY ENDPOINTS 15
4.2.1 PRIMARY ENDPOINTS 15
4.2.2 SECONDARY ENDPOINTS 15
4.2.3 EXPLORATORY ENDPOINTS 15
4.2.4 DEFINITIONS OF PRIMARY AND SECONDARY ENDPOINTS 15
4.2.5 ADJUDICATION OF ENDPOINTS 16
5 STUDY ENROLLMENT AND WITHDRAWAL 16
5.1 PARTICIPANT INCLUSION CRITERIA 16
5.2 PARTICIPANT EXCLUSION CRITERIA 17
5.3 INFORMED CONSENT FOR SCREENING AND RANDOMIZATION 18
5.4 ATRIAL CARDIOPATHY CRITERIA REQUIRED FOR RANDOMIZATION 19
5.5 STRATEGIES FOR RETENTION AND RECRUITMENT 19
5.5.1 TIME PERIOD FOR RECRUITMENT 19
5.5.2 INCLUSION OF WOMEN AND MINORITIES 20
5.6 PARTICIPANT WITHDRAWAL OR TERMINATION 20
5.6.1 REASONS FOR WITHDRAWAL OR TERMINATION 20
5.6.2 HANDLING OF PARTICIPANT WITHDRAWALS OR TERMINATION 21
5.7 PREMATURE TERMINATION OR SUSPENSION OF STUDY 21
6 STUDY AGENT 22
6.1 STUDY AGENT AND CONTROL DESCRIPTION 22
6.1.1 ACQUISITION 22
6.1.2 FORMULATION, APPEARANCE, PACKAGING, AND LABELING 22
6.1.3 PRODUCT STORAGE AND STABILITY 22
6.1.4 DISTRIBUTION 22
6.1.5 DOSING 22
6.1.6 ADMINISTRATION 23
6.1.7 LABORATORY MONITORING AND DOSE ADJUSTMENTS/INTERRUPTIONS 23
6.1.8 TRACKING OF DOSE 23
6.2 STUDY DRUG ACCOUNTABILITY PROCEDURES 23
7 STUDY PROCEDURES AND SCHEDULE 24
7.1 STUDY PROCEDURES/EVALUATIONS 24
7.1.1 STUDY-SPECIFIC PROCEDURES 24
7.1.2 STANDARD-OF-CARE STUDY PROCEDURES 25

7.2 STUDY SCHEDULE 26
7.2.1 SCREENING 26
7.2.2 RANDOMIZATION 28
7.2.3 FOLLOW-UP 28
7.2.4 FINAL STUDY VISIT 29
7.2.5 UNSCHEDULED VISIT 29
7.2.6 SCHEDULE OF EVENTS TABLE 30
7.3 CONCOMITANT AND PROHIBITED MEDICATIONS 31
7.3.1 ANTIPLATELET MEDICATIONS 31
7.3.2 ANTICOAGULANT MEDICATIONS 31
7.3.3 STRONG INDUCERS AND INHIBITORS OF CYP3A4 AND P-gp 31
7.4 DETECTION AND MANAGEMENT OF ATRIAL FIBRILLATION 31
7.5 INTERRUPTION OF STUDY DRUG FOR ELECTIVE INVASIVE PROCEDURES 32
7.6 MANAGEMENT OF BLEEDING 32
7.7 MANAGEMENT OF ACUTE ISCHEMIC STROKE IN SUBJECTS ON STUDY DRUG 32
7.8 VASCULAR RISK FACTOR MANAGEMENT 33
7.9 PARTICIPANT ACCESS TO STUDY DRUG AFTER STUDY CLOSURE 33
8 ASSESSMENT OF SAFETY 33
8.1 SPECIFICATION OF SAFETY PARAMETERS 33
8.1.1 DEFINITION OF ADVERSE EVENTS 34
8.1.2 DEFINITION OF SERIOUS ADVERSE EVENTS 34
8.1.3 ADVERSE EVENTS OF SPECIAL INTEREST 35
8.1.4 UNANTICIPATED PROBLEMS AND UNANTICIPATED EVENTS 36
8.2 CLASSIFICATION OF AN ADVERSE EVENT 36
8.2.1 SEVERITY OF EVENT 36
8.2.2 RELATIONSHIP TO STUDY AGENT 37
8.2.3 EXPECTEDNESS 37
8.3 TIME PERIOD AND FREQUENCY FOR EVENT ASSESSMENT AND FOLLOW-UP 37
8.4 REPORTING PROCEDURES 38
8.4.1 ADVERSE EVENT REPORTING 38
8.4.2 SAFETY REPORTING 38
8.4.3 UNANTICIPATED EVENT REPORTING 39
8.5 STUDY OVERSIGHT 39
8.6 STUDY HALTING RULES 40
9 CLINICAL MONITORING 40
10 STATISTICAL CONSIDERATIONS 40
10.1 STATISTICAL AND ANALYTICAL PLAN 40
10.2 STATISTICAL HYPOTHESES 41
10.3 ANALYSIS DATASETS 41
10.4 DESCRIPTION OF STATISTICAL METHODS 41
10.4.1 GENERAL APPROACH 41
10.4.2 ANALYSIS OF THE PRIMARY AND SECONDARY ENDPOINTS 41
10.4.3 SAFETY ANALYSES 41
10.4.4 PLANNED INTERIM ANALYSES 42
10.4.5 ADDITIONAL SUBGROUP ANALYSES 42
10.4.6 EXPLORATORY AND SENSITIVITY ANALYSES 42
10.5 SAMPLE SIZE 42
10.5.1 ASSUMPTIONS 42
10.5.2 SAMPLE SIZE CALCULATION 42

10.6 MEASURES TO MINIMIZE BIAS 43
10.6.1 ENROLLMENT, RANDOMIZATION, AND MASKING PROCEDURES 43
10.6.2 BREAKING THE STUDY BLIND 43
11 SOURCE DOCUMENTS AND ACCESS TO SOURCE DATA 43
12 QUALITY ASSURANCE AND QUALITY CONTROL 44
13 ETHICS/PROTECTION OF HUMAN SUBJECTS 44
13.1 ETHICAL STANDARD 44
13.2 INSTITUTIONAL REVIEW BOARD 44
13.3 INFORMED CONSENT PROCESS 45
13.3.1 CONSENT AND OTHER INFORMATION DOCUMENTS PROVIDED TO PARTICIPANTS 45
13.3.2 CONSENT PROCEDURES AND DOCUMENTATION 45
13.3.3 USE OF SURROGATES FOR CONSENT 45
13.4 PARTICIPANT AND DATA CONFIDENTIALITY 47
13.4.1 CONFIDENTIALITY AND DATA STORAGE 47
13.4.2 RESEARCH USE OF STORED HUMAN SAMPLES 47
13.5 FUTURE USE OF STORED SPECIMENS 48
14 DATA HANDLING AND RECORD KEEPING 49
14.1 DATA COLLECTION AND MANAGEMENT RESPONSIBILITIES 49
14.2 STUDY RECORDS RETENTION 50
14.3 PROTOCOL DEVIATIONS 50
14.4 PUBLICATION AND DATA SHARING POLICY 50
15 STUDY ADMINISTRATION 51
15.1 OVERVIEW 51
15.2 STUDY MANAGEMENT CORES (NCC and CCC) 51
15.3 DATA MANAGEMENT CORE (NDMC) 52
15.4 EXECUTIVE COMMITTEE 52
15.5 TRIAL OPERATIONS COMMITTEE 52
15.6 PUBLICATIONS COMMITTEE 52
15.7 ANCILLARY STUDIES COMMITTEE 52
16 CONFLICT OF INTEREST POLICY 53
17 LITERATURE REFERENCES 53
CHANGE LOG 56

LIST OF ABBREVIATIONS

AE	Adverse event
AF	Atrial fibrillation/flutter
AHA/ASA	American Heart Association/American Stroke Association
ALT	Alanine aminotransferase
ARCADIA	AtRial Cardiopathy and Antithrombotic Drugs In prevention After cryptogenic stroke
AST	Aspartate aminotransferase
BMI	Body mass index
CALM	Center for Advanced Laboratory Medicine at Columbia University Medical Center
CCC	Canadian Coordinating Center
CFR	Code of Federal Regulations
cIRB	StrokeNet central institutional review board
CRF	Case report form
CT	Computed tomography
CTA	CT angiogram
CTCAE	Common Terminology Criteria for Adverse Events
CUMC	Columbia University Medical Center
DSMB	Data and safety monitoring board
ECG	Electrocardiogram
eCrCl	Estimated creatinine clearance
ESUS	Embolic stroke of undetermined source
FDA	Food and Drug Administration
FDAAA	FDA Amendments Act
GFR	Glomerular filtration rate
GI	Gastrointestinal
GCP	Good clinical practices
GLP	Good laboratory practices
GMP	Good manufacturing practices
HIPAA	Health Insurance Portability and Accountability Act
ICH	International Council for Harmonization
ICMJE	International Committee of Medical Journal Editors
ID	Identification
IRB	Institutional review board
INR	International normalized ratio
MOP	Manual of procedures
MRA	MR angiogram
MRI	Magnetic resonance imaging
MRS	Modified Rankin Scale
MSM	Medical safety monitor
NCC	NIH StrokeNet National Coordinating Center
NDMC	NIH StrokeNet National Data Management Center
NIH	National Institutes of Health
NIHSS	National Institutes of Health Stroke Scale
NINDS	National Institute of Neurological Disorders and Stroke

NOAC	Non-vitamin K antagonist oral anticoagulant drug
NT-proBNP	Amino terminal pro-B-type natriuretic peptide
OHRP	Office of Human Research Protections
PHI	Protected health information
PI	Principal Investigator
PM	Project manager
PROMIS	Patient-Reported Outcomes Measurement Information System
PT	Prothrombin time
$PTFV_1$	P-wave terminal force in ECG lead V_1
PTT	Partial thromboplastin time
PUDS	Public use data sets
QC	Quality control
QVSFS	Questionnaire for Verification of Stroke-Free Status
REB	Research ethics board
SAE	Serious adverse event
SAP	Statistical analysis plan
SOP	Standard operating procedure
TEE	Transesophageal echocardiogram
TTE	Transthoracic echocardiogram
UP	Unanticipated problem

STATEMENT OF COMPLIANCE

The trial will be conducted in accordance with the International Council for Harmonization (ICH) E6, the Code of Federal Regulations on the Protection of Human Subjects (45 CFR Part 46), and the NINDS Terms of Award. The co-Principal Investigators (co-PIs) will assure that no deviation from or changes to the protocol will take place without prior agreement from NINDS and documented approval from the central institutional review board (cIRB) and/or other applicable IRB/REBs with study oversight, except where necessary to eliminate an immediate hazard to the trial participants. All personnel involved in the conduct of this study have completed human subjects protection training.

We agree to ensure that all staff members involved in the conduct of this study are informed about their obligations in meeting the above commitments.

PI: Hooman Kamel, MD

Signed: ____ Date: 17 Aug 2022

PI: Mitchell S.V. Elkind, MD, MS

Signed: ____ Date: 17 Aug 2022

PI: W.T. Longstreth, Jr, MD, MPH

Signed: ____ Date: 17 Aug 2022

PI: David Tirschwell, MD, MSc

Signed: ____ Date: 17 Aug 2022

PI: Randolph S. Marshall, MD

Signed: ____ Date: 17 Aug 2022

ARCADIA

AtRial Cardiopathy and Antithrombotic Drugs In prevention After cryptogenic stroke

Protocol Identifying Number: NCT03192215

PROTOCOL ACCEPTANCE FORM / SIGNATURE PAGE

Version: 6
Date: 17 Aug 2022

By signing below I confirm that:

1. I have read this protocol and it contains all necessary details for conducting this study

AND

2. I agree to conduct the trial in compliance with this protocol and to adhere to all regulations that govern the conduct of the study.

______________________________ ______________

Principal Investigator's Signature **Date**

____

Principal Investigator's Name (Print)

____

Site Name

PROTOCOL SUMMARY

**Title:**	AtRial Cardiopathy and Antithrombotic Drugs In prevention After cryptogenic stroke.
**Précis:**	ARCADIA is a multicenter, biomarker-driven, randomized, double-blind, active-control, phase 3 clinical trial of apixaban versus aspirin in patients who have evidence of atrial cardiopathy and a recent stroke of unknown cause. Eleven hundred subjects will be recruited at up to 200 sites in the United States and Canada. Subjects will be followed for a minimum of 1.5 years for the primary efficacy outcome of recurrent stroke and the primary safety outcomes of symptomatic intracranial hemorrhage and major hemorrhage other than intracranial hemorrhage.
**Objectives:**	• *Primary*: To test the hypothesis that apixaban is superior to aspirin for the prevention of recurrent stroke in patients with cryptogenic ischemic stroke and atrial cardiopathy.<br>• *Secondary*: To test the hypothesis that the relative efficacy of apixaban over aspirin increases with the severity of atrial cardiopathy.
**Endpoints:**	• *Primary efficacy outcome*: Recurrent stroke of any type (ischemic, hemorrhagic, or of undetermined type).<br>• *Primary safety outcomes*: (A) Symptomatic intracranial hemorrhage (including symptomatic hemorrhagic transformation of an ischemic stroke), and (B) major hemorrhage other than intracranial hemorrhage.<br>• *Secondary efficacy outcomes*: (A) Composite outcome of recurrent ischemic stroke or systemic embolism, and (B) composite outcome of recurrent stroke of any type or death from any cause.<br>• *Secondary safety outcome*: All-cause mortality.
**Population:**	1,100 patients with a recent embolic stroke of undetermined source (ESUS) and evidence of atrial cardiopathy.
**Phase:**	Phase 3.
**Number of sites:**	Up to 200.
**Study agent:**	• *Active agent*: Apixaban (trade name Eliquis®) 5 mg by mouth twice daily (2.5 mg twice daily if standard criteria for adjusted dose are met).<br>• *Active control*: Aspirin 81 mg by mouth once daily.
**Study duration:**	7 years.
**Participant duration:**	Minimum of 1.5 years and maximum of 7 years.

SCHEMATIC OF STUDY DESIGN

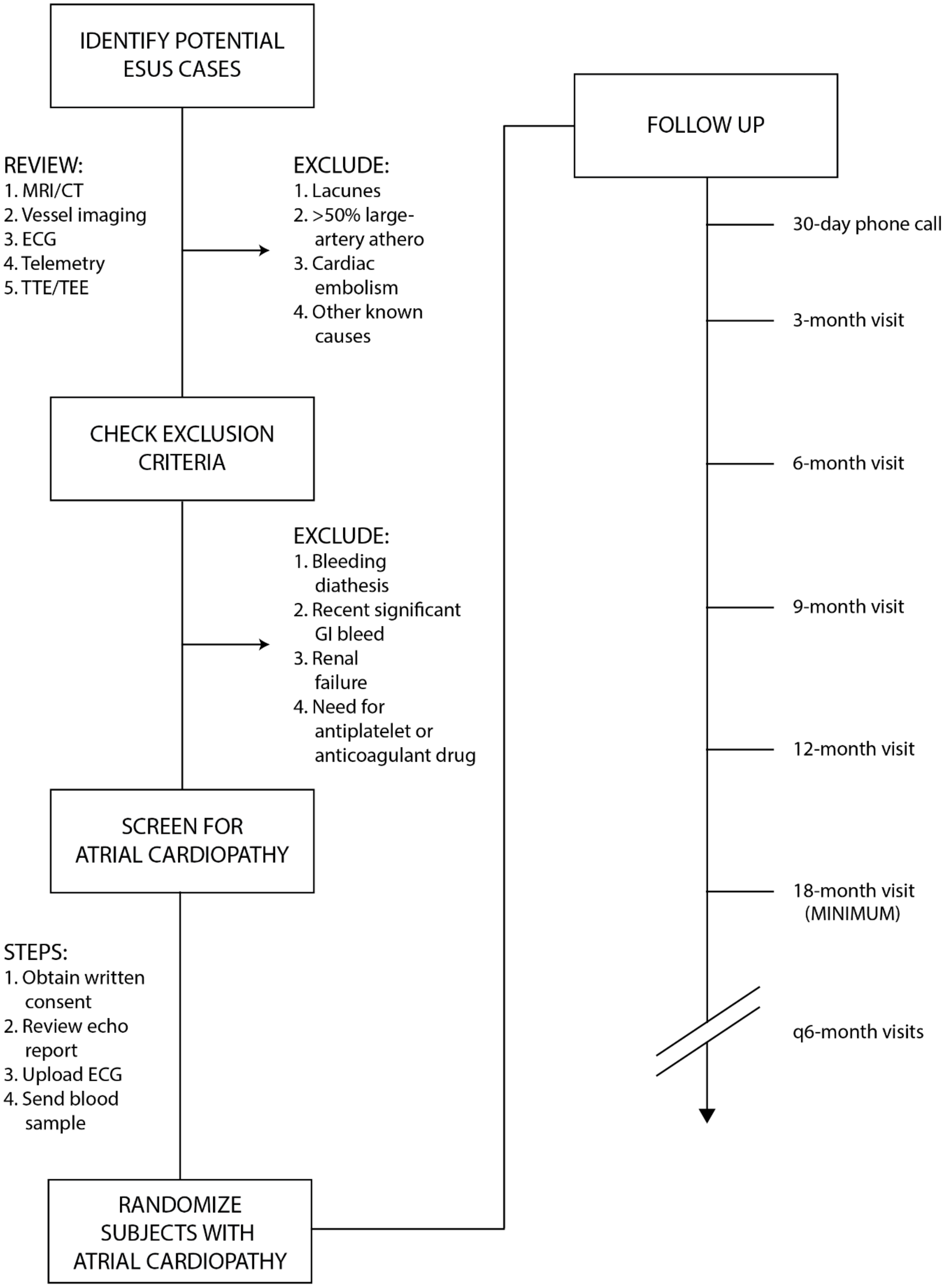

1 KEY ROLES

**Contact PI**	Randolph S. Marshall, MD<br>rsm2@cumc.columbia.edu
**Co-PI**	Mitchell S. V. Elkind, MD, MS<br>mse13@cumc.columbia.edu
**Protocol PI**	Hooman Kamel, MD<br>hok9010@med.cornell.edu
**Co-PI, Director of Eligibility Core and Recruitment Core**	David Tirschwell, MD<br>tirsch@uw.edu
**Co-PI, Director of Outcomes Adjudication Core**	W.T. Longstreth, Jr, MD, MPH<br>wl@uw.edu
**Blinded Statistician**	Richard Kronmal, PhD<br>kronmal@uw.edu
**StrokeNet NCC PI**	Joseph Broderick, MD<br>broderjp@ucmail.uc.edu
**StrokeNet NDMC PI**	Jordan Elm, PhD<br>elmj@musc.edu
**NINDS Project Scientists**	Scott Janis, PhD<br>janiss@ninds.nih.gov
**Canadian Sites Lead PI**	David Gladstone, MD, PhD<br>David.gladstone@sunnybrook.ca
**Canadian Stroke Consortium Chair**	Mike Sharma, MD, Msc<br>Mike.sharma@phri.ca
**Canadian Coordination Centre (CCC) Lead PI**	Jeff Healey, MD, Msc<br>Jeff.healey@phri.ca

A full list of key personnel and their contact information is provided in the Manual of Operations.

2 INTRODUCTION: BACKGROUND AND SCIENTIFIC RATIONALE

2.1 BACKGROUND

In one-third of ischemic strokes, a specific cause cannot be identified.[1] Many of these cryptogenic strokes appear to arise from a distant embolic source.[2] Recent evidence suggests that some cryptogenic strokes may arise from left atrial thromboembolism that goes unrecognized because it has not manifested with atrial fibrillation/flutter (AF). In the ASSERT study, which showed that even a single 6-minute episode of subclinical AF was associated with a heightened risk of stroke,[3] 31% of patients with both subclinical AF and stroke had no episodes of AF during a median 8 months of continuous cardiac monitoring before the stroke, only manifesting AF for the first time after the stroke.[4] These findings were closely replicated in the more recent IMPACT trial.[5] Such a lack of temporal relationship between AF and stroke undermines the concept that AF itself directly causes cardiac embolism, and is more consistent with AF as a marker of a diseased left atrium characterized by inflammation, endothelial dysfunction, fibrosis, contractile dysfunction, structural derangement, or some combination of these factors. Supporting this possibility, other markers of left atrial abnormality are associated with stroke in the absence of AF. Clinical diagnoses of paroxysmal supraventricular tachycardia have been associated with stroke risk in patients without AF.[6] More recently, increased P-wave terminal force in electrocardiogram (ECG) lead V_1 ($PTFV_1$)—a well-established marker of left atrial abnormality[7]—has been associated with stroke even in the absence of AF.[8-11] Left atrial size on echocardiogram has also been correlated with stroke risk in the absence of AF.[12-15] Lastly, serum levels of NT-pro-BNP, a marker of cardiac myonecrosis and elevated filling pressures, correlate with stroke risk independent of AF.[16,17] These data suggest that an underlying atrial cardiopathy may provide the substrate for thrombus formation and embolization even in the absence of AF.[18] These observations may also explain why fewer than one-third of patients with cryptogenic stroke in the CRYSTAL-AF trial manifested AF even after 3 years of continuous heart-rhythm monitoring.[19]

In parallel with these new insights about cryptogenic stroke, new therapeutic options for stroke prevention have become available. Non-vitamin K antagonist oral anticoagulant (NOAC) drugs such as apixaban may be more effective than aspirin for treatment of cryptogenic stroke. A benefit is especially likely in cryptogenic stroke patients with atrial cardiopathy because of parallels with AF and because an analysis of data from the WARSS/APASS studies suggests that cryptogenic stroke patients with one marker of atrial cardiopathy (elevated NT-proBNP) and no obvious AF benefit from anticoagulant therapy.[20] Apixaban is a particularly attractive choice because it has a low bleeding risk,[21,22] lowers mortality more than warfarin in patients with AF,[23] and is the only NOAC drug with a Class 1A recommendation in recent AHA/ASA guidelines.[24]

Among patients with causes of cryptogenic stroke other than atrial cardiopathy, data are less compelling on the efficacy of anticoagulant therapy.[25] Many cryptogenic strokes may be due to artery-to-artery embolism from non-stenotic and unrecognized large-artery atherosclerosis.[26-28] In patients with stroke from known intracranial atherosclerosis, the WASID trial found no benefit of warfarin over aspirin[29] and the SAMMPRIS trial found historically low rates of recurrence with dual antiplatelet therapy.[30] The ARCH trial demonstrated a trend towards fewer recurrent strokes with antiplatelet therapy compared to anticoagulant therapy in those with aortic arch atheroma.[31] These data suggest that new oral anticoagulant agents are unlikely to be superior to antiplatelet therapy in cryptogenic stroke patients without atrial cardiopathy.

2.2 RATIONALE

These data justify a randomized trial of apixaban versus aspirin specifically in patients with cryptogenic stroke who have evidence of atrial cardiopathy. This trial design will address several important knowledge gaps. First, it will advance our understanding of stroke pathophysiology by assessing whether atrial cardiopathy is a valid therapeutic target, which may set the stage for a primary prevention trial. Second, this trial will advance our understanding of optimal secondary stroke prevention therapy. One industry-sponsored trial (NAVIGATE-ESUS) that compared rivaroxaban to aspirin in patients with cryptogenic stroke was stopped early for futility. Another industry-sponsored trial (RESPECT-ESUS) is comparing dabigatran versus aspirin in patients with cryptogenic stroke. By including all cryptogenic stroke patients and by including those with up to 6 minutes of AF per day, these trials have mixed patients with heterogeneous stroke mechanisms. These trials may thus fail to show an overall benefit (as in NAVIGATE-ESUS), or may show an overall benefit driven mostly by patients with known AF and atrial cardiopathy, resulting in an overly broad indication for anticoagulant therapy. In the first instance, our trial may ensure that a valuable treatment in a specifically targeted subgroup will not be prematurely abandoned. In the second instance, the results of our trial would provide a compelling rationale to perform subgroup analyses of the industry-sponsored trials and conduct future trials to determine the risks and benefits of anticoagulant therapy across biologically distinct subgroups of stroke patients. Given the expense and risks of bleeding associated with anticoagulant drugs, it is imperative to define as precisely as possible the groups of stroke patients who would and would not benefit from their use. Therefore, our approach comports well with the general move toward precision medicine.

2.3 POTENTIAL RISKS AND BENEFITS

2.3.1 KNOWN POTENTIAL RISKS

Major hemorrhage. Both treatment arms (apixaban versus aspirin) will involve antithrombotic medications that necessarily increase the risk for hemorrhage. Despite its associated bleeding risks, the benefit of aspirin in secondary stroke prevention has long been established to outweigh these risks, and aspirin receives a Class I, Level of Evidence A recommendation in American Heart Association guidelines on secondary stroke prevention.[24] Apixaban is a much newer drug than aspirin, and as an anticoagulant drug, it has a theoretically higher risk of bleeding than an antiplatelet drug such as aspirin. However, in the secondary stroke prevention substudy of the AVERROES trial, which enrolled a comparable population of patients as in our proposed trial, the rate of major hemorrhage was not significantly higher with apixaban compared to aspirin.[32]

Recurrent stroke. A 7% annual risk of recurrent stroke is anticipated. It is hypothesized that patients in the intervention arm (apixaban) will face a 40% lower relative risk of recurrent stroke compared to those in the active-control arm receiving standard therapy (aspirin).

Delay of or exclusion from treatment with thrombolytic drugs for recurrent ischemic stroke. Patients actively taking oral anticoagulant drugs such as apixaban are generally excluded from treatment with intravenous thrombolysis for acute ischemic stroke.

Discovery of incidental conditions. This risk is expected to be low because the only diagnostic test that will be performed solely for the purposes of the proposed trial is the collection of a blood sample that will be assayed for NT-proBNP. NT-proBNP can reflect ventricular as well as atrial dysfunction, but all

patients will undergo echocardiography and those with significant heart failure will be excluded prior to the collection of this blood sample. Therefore, an elevated NT-proBNP level in this study will be most likely to reflect atrial cardiopathy rather than another incidental condition. The other diagnostic tests used in this trial—echocardiography and ECG—will have been done as part of standard care and will have been interpreted by clinicians at each site, so that usual clinical protocols will be in place to document and manage incidental findings.

Loss of confidentiality. It is expected that this risk will be low because of numerous safeguards that will be in place, including standard data management procedures at the NDMC.

Discomfort from phlebotomy. Patients who consent to screening for atrial cardiopathy will need to undergo collection of a blood sample for an NT-proBNP assay. Discomfort from this procedure will be minimized by adding on this blood sample to already-scheduled phlebotomies for routine, clinically indicated laboratory tests whenever possible.

Allergic reaction or liver injury. It is expected that these risks will be low based on existing data.

2.3.2 KNOWN POTENTIAL BENEFITS

A successful outcome in the proposed trial would have immediate implications for secondary stroke prevention by identifying a new group of stroke patients who benefit from anticoagulant therapy. Such a result would answer a significant unmet need by providing robust evidence to guide directed treatments for cryptogenic stroke.[2] Such a result would also have near-term implications for primary stroke prevention. Any success of anticoagulant therapy in reducing stroke risk in this high-risk population with atrial cardiopathy would suggest the possibility of benefit in patients with atrial cardiopathy and no history of stroke. If anticoagulation can prevent recurrent stroke from atrial cardiopathy, it should also prevent stroke from occurring in the first place. Thus, validation of atrial cardiopathy as a therapeutic target may set the stage for primary prevention trials. These likely potential benefits, for which we have compelling preliminary data, are highly likely to outweigh the potential risks of the interventions in this trial, both of which involve FDA-approved antithrombotic agents in widespread clinical use and with good safety data in comparable populations.[32] In addition to the benefits to public health, this information could also be of benefit to individual participants in the study, as their own future secondary stroke prevention treatment after the completion of this trial would be influenced by its results.

3 OBJECTIVES AND PURPOSE

The primary aim is to test the hypothesis that apixaban is superior to aspirin for the prevention of recurrent stroke in subjects with cryptogenic ischemic stroke and atrial cardiopathy.

The secondary aim is to test the hypothesis that the relative efficacy of apixaban over aspirin increases with the severity of atrial cardiopathy.

4 STUDY DESIGN AND ENDPOINTS

4.1 DESCRIPTION OF THE STUDY DESIGN

Multicenter, biomarker-driven, randomized, double-blind, active-control, phase 3 clinical trial of apixaban versus aspirin.

4.2 STUDY ENDPOINTS

4.2.1 PRIMARY ENDPOINTS

Efficacy: Recurrent stroke of any type (ischemic, hemorrhagic, or of undetermined type).
Safety: (A) Symptomatic intracranial hemorrhage (including symptomatic hemorrhagic transformation of an ischemic stroke), and (B) major hemorrhage other than intracranial hemorrhage.

4.2.2 SECONDARY ENDPOINTS

Efficacy: (A) Composite of recurrent ischemic stroke or systemic embolism, and (B) composite of recurrent stroke of any type or death from any cause.
Safety: All-cause mortality.

4.2.3 EXPLORATORY ENDPOINTS

See the manual of procedures (MOP) for definitions of other endpoints used for exploratory analyses: AF, any intracranial hemorrhage, major hemorrhage including any intracranial hemorrhage, symptomatic hemorrhagic transformation of an ischemic stroke, transient ischemic attack, myocardial infarction, minor hemorrhage, systemic embolism, symptomatic deep venous thrombosis, symptomatic pulmonary embolism, ischemic vascular death, and hemorrhagic vascular death.

4.2.4 DEFINITIONS OF PRIMARY AND SECONDARY ENDPOINTS

**Stroke:**	• Composite of ischemic stroke, hemorrhagic stroke, or stroke of undetermined type.
**Ischemic stroke:**	• Rapid onset of a new focal neurological deficit: 1) imaging or other evidence of infarction in a part of the central nervous system consistent with symptoms OR 2) lasting ≥24 hours without imaging evidence of infarction, AND 3) not attributable to a non-ischemic etiology, such as intracranial hemorrhage, edema, infection, trauma, tumor, seizure, severe metabolic disease, or degenerative neurological disease.
**Hemorrhagic stroke:**	• Acute extravasation of blood into the brain parenchyma, subarachnoid space, or intraventricular space, judged to be non-traumatic and not in the area of an acute or subacute ischemic infarct but associated with and identified as the predominant

	cause of new neurologic symptoms, including headache, or leading to death.
**Stroke of undetermined type:**	• Rapid onset of a new focal neurological deficit: 1) lasting ≥24 hours AND 2) not meeting criteria for ischemic stroke or hemorrhagic stroke AND 3) not attributable to a non-vascular etiology, such as edema, infection, trauma, tumor, seizure, severe metabolic disease, or degenerative neurological disease.
**Symptomatic intracranial hemorrhage:**	• Any extravascular blood within the cranium, including subdural and epidural blood, associated with and identified as the predominant cause of new neurologic symptoms, including headache, or leading to death.
**Major hemorrhage other than intracranial hemorrhage:**	• Clinically overt bleeding accompanied by a ≥2 g/dL decrease in the hemoglobin level during a 24-hour period, transfusion of ≥2 units of whole blood or red cells, involvement of a critical non-intracranial site (intraspinal, intraocular, pericardial, intraarticular, intramuscular with compartment syndrome, or retroperitoneal), or death.[33]
**Systemic embolism:**	• Clinical history consistent with an acute loss of blood flow to a peripheral artery or arteries and supported by evidence of embolism from surgical specimens, autopsy, angiography, or other objective testing.

4.2.5 ADJUDICATION OF ENDPOINTS

Study endpoints will be adjudicated using procedures defined in the MOP and approved by the NINDS-appointed DSMB. Regulatory reporting of SAEs and AEs of special interest will not be delayed for this adjudication to occur.

5 STUDY ENROLLMENT AND WITHDRAWAL

5.1 PARTICIPANT INCLUSION CRITERIA

- Age ≥45 years.
- Clinical diagnosis of ischemic stroke + brain imaging to rule out hemorrhagic stroke.
- Modified Rankin Scale (MRS) score ≤4.
- Ability to be randomized no later than 180 days after stroke onset.
- ESUS, defined as **all** of the following[2]:
  - Stroke that is not lacunar. Lacunar is defined as a subcortical (this includes pons and midbrain) infarct in the distribution of the small, penetrating cerebral arteries whose largest dimension is ≤1.5 cm on CT, ≤2.0 cm on MRI diffusion images, or ≤1.5 cm on MRI T2-weighted images. The following are not considered lacunes: multiple simultaneous small

deep infarcts, lateral medullary infarcts, and cerebellar infarcts. Patients with a clinical lacunar stroke syndrome and no infarct on imaging are excluded.

- Absence of extracranial or intracranial atherosclerosis causing ≥50% luminal stenosis of the artery supplying the area of ischemia. Patients must undergo vascular imaging of the extracranial and intracranial vessels using either catheter angiography, CT angiogram (CTA), MR angiogram (MRA), or ultrasound, as considered appropriate by the treating physician and local principal investigator. Training will be provided to the investigators that will encourage the use of CTA and MRA over ultrasound for the evaluation of patients to minimize operator-dependent variation.
- No major-risk cardioembolic source of embolism, including AF, intracardiac thrombus, mechanical prosthetic cardiac valve, atrial myxoma or other cardiac tumors, moderate or severe mitral stenosis, myocardial infarction within the last 4 weeks, left ventricular ejection fraction <30%, valvular vegetations, or infective endocarditis. Patent foramen ovale is not an exclusion unless it is determined to be causally related to the stroke. All patients must undergo electrocardiogram, transthoracic or transesophageal echocardiography (TTE or TEE), and at least 24 hours of cardiac rhythm monitoring (Holter monitor or telemetry or equivalent). Additional cardiac imaging, such as cardiac MRI or cardiac CT, will be performed at the discretion of the local treating physician and principal investigator. Additional cardiac rhythm monitoring, such as monitored cardiac outpatient telemetry (MCOT) or an implanted cardiac monitor, will be at the discretion of the treating physician and local principal investigator. Although standard-of-care echocardiography will be used to make diagnoses of cryptogenic stroke and for atrial cardiopathy criteria, these echocardiograms will be centrally read as well to confirm local readings and provide feedback to local sites.
- No other specific cause of stroke identified, such as arteritis, dissection, migraine, vasospasm, drug abuse, or hypercoagulability. Special testing, such as toxicological screens, serological testing for syphilis, and tests for hypercoagulability, will be performed at the discretion of the treating physician and local principal investigator. Training will be provided to local investigators to consider obtaining hypercoagulability tests among patients with patent foramen ovale.

5.2 PARTICIPANT EXCLUSION CRITERIA

- History of AF, AF on 12-lead ECG, or any AF of any duration during heart-rhythm monitoring prior to randomization.
- Clear indication for treatment-dose anticoagulant therapy, such as venous thromboembolism or a mechanical heart valve.
- Left ventricular ejection fraction <30%.
- Definite indication for antiplatelet agent (e.g., aspirin or clopidogrel after implantation of a coronary artery stent).
- History of spontaneous intracranial hemorrhage.
- Chronic kidney disease with serum creatinine ≥2.5 mg/dL. For Canadian sites only, estimated creatinine clearance (eCrCl) <15 mL/min is also an exclusion criterion.
- Active hepatitis or hepatic insufficiency with Child-Pugh score B or C (see MOP for definition).
- Clinically significant bleeding diathesis.
- Anemia (hemoglobin <9 g/dL) or thrombocytopenia (<100 x 10^9/L) that is chronic in the judgment of the investigator.

- GI bleeding within the past year considered clinically significant by the investigator.
- Pregnancy risk:
    - Female patient who is known to be pregnant.
    - Female patient who is sexually active and premenopausal without a negative pregnancy test performed after stroke onset.
    - Female patient who is sexually active and premenopausal, and who does not commit to adequate birth control.
    - Male patient who is sexually active with a premenopausal female partner, and who does not commit to adequate birth control.
- Known allergy or intolerance to aspirin or apixaban.
- Concomitant participation in another clinical trial involving a drug or acute stroke intervention.
- Considered by the investigator to have a condition that precludes follow-up or safe participation in the trial.
- Inability to obtain written, informed consent from patient or surrogate for trial participation.

5.3 INFORMED CONSENT FOR SCREENING AND RANDOMIZATION

If a patient fulfills all of the inclusion/exclusion criteria, they or their surrogate will be approached for consent per the process described in Section 7.2.1 and according to local regulations.

Written consent should preferably be obtained in person. This can be done using a paper form or a HIPAA-compliant method for eConsent (e.g., REDCap). If consent cannot be obtained in person, written consent can be obtained via a HIPAA-compliant method per the following procedure:

- Contact the patient or surrogate by telephone or other HIPAA-compliant telehealth technology.
- Transmit the entire consent form so that the patient or surrogate has it available to read during the discussion.
- Provide a comprehensive explanation of the purpose, procedures, and possible risks/benefits of the study in language that is understandable to a non-medically trained person; explain the participant responsibilities and the fact that participation is voluntary; that the participant may withdraw from the study at any time; and that the decision not to participate or to withdraw will not affect the patient's care in any way. Provide ample opportunity for the patient or surrogate to ask questions and to consider the decision.
- If the patient or surrogate expresses a sustained interest, give instructions about how the patient or surrogate needs to sign and date the consent form and how to transmit the signed form back.
- The patient or surrogate should return the entire signed and dated informed consent form back at which point the person obtaining consent should sign and date the form and transmit a copy of the fully signed consent form back to the patient or surrogate for his/her records. The informed consent form is not valid and study enrollment cannot proceed unless all pages are received and appropriately filled out/signed/dated by the patient or surrogate.

Patients who provide consent at this stage will be considered consented but not randomized. Patients will be randomized only if they meet ≥1 of the atrial cardiopathy criteria below.

Reconsenting Process

If changes are made to the consent during the course of the trial, the cIRB or other IRB/REBs with study oversight will determine whether active subjects will need to be re-consented. Ideally re-consenting should be done in person and mirror the initial consenting process. Because some subjects in the ARCADIA trial may have phone follow up visits before or in lieu of in person visits, the procedure below should be followed if it is necessary to mail a new consent to a subject:

1. Contact the person that you are planning to re-consent by phone and explain that you need to re-consent them. Explain why you need to re-consent them, make arrangements for mailing them the new consent and a time to schedule the re-consenting call. Ask them not to sign the consent until the time of the re-consenting call.
2. Mail 2 copies of the consent to the participant with instruction not to sign the form until the re-consent call has been made. Include a self-addressed stamped envelope for the participant to return one of the signed consents to the researcher. The person obtaining the verbal consent would have signed their part of the consent before mailing (they would need to make a note that the date they entered is the date they mailed the consent to the participant).
3. The study staff will have the consent process discussion with the participant on the phone and answer any questions the participant may have. Both parties will have the consent in front of them during the call and reference it during the re-consenting process (same as would be done in-person). Ensure that all of the participant's questions have been answered.
4. Record documentation of this entire conversation and the verbal phone consent. Have the participant sign both of the consents and mail one signed consent back to the site.
5. The site would then document the process used and the date they received the fully signed consent from the participant.
6. Reconsenting can be done via eConsent as allowed by location regulations and IRB/REB requirements.

5.4 ATRIAL CARDIOPATHY CRITERIA REQUIRED FOR RANDOMIZATION

To be eligible for randomization, patients must meet criteria for atrial cardiopathy in addition to the inclusion/exclusion criteria above. Atrial cardiopathy is defined as ≥1 of the following:

- $PTFV_1$ >5,000 µV*ms on 12-lead ECG (ECG criterion).
- Serum NT-proBNP >250 pg/mL (NT-proBNP criterion).
- Left atrial diameter index ≥3 cm/m^2 on echocardiogram (i.e., severe left atrial enlargement) (ECHO criterion).

5.5 STRATEGIES FOR RETENTION AND RECRUITMENT

5.5.1 TIME PERIOD FOR RECRUITMENT

Subject recruitment is planned to be carried out over a maximum of 6 years. Study subjects will be followed for a minimum of 1.5 years.

5.5.2 INCLUSION OF WOMEN AND MINORITIES

Inclusion or exclusion of subjects will not differ based on sex/gender, and the aim is to recruit similar numbers of men and women. The absence of an upper age limit will aid recruitment of women given that they are generally older than men at the time of a first cardiovascular event such as stroke. Inclusion or exclusion of subjects will not differ based on race/ethnicity. To ensure representative enrollment of minority groups, site PIs and their clinical research staff will be trained and periodically reminded to:

- Be mindful of cultural, historical, social, and political factors that may influence minority participation. It should be noted that many individuals (particularly certain racial/ethnic groups) need clarification on what a clinical trial is; understanding of what type of research is being conducted; who is conducting the research study, benefits to individuals and the community; place of worship's encouragement, etc.
- Emphasize that the goal is to recruit patients regardless of race or ethnicity.
- Clearly explain that the purpose of informed consent is to protect, not relinquish, participants' rights.
- Provide time if necessary between screening and randomization for patients to reflect and discuss participation with family.
- Emphasize the proven track record of the study drugs.
- Emphasize the trial's potential to help find better treatments to prevent stroke.
- Mitigate concern about randomization by clearly explaining its rationale and highlighting the possibility of cross over if clinically indicated.
- Emphasize that participants will be kept up to date on scientific developments in this field throughout their participation in the trial.
- Think about modes of communication for outreach and retention, such as follow-up phone calls, social media campaigns, text messaging, to identify what works best for the community/population (as allowed by local regulations).
- Provide translation services or bilingual staff, if needed for recruiting populations for which English is not the primary language.
- Use motivational interviewing techniques as a method to improve communication between the research coordinator and patient population to enhance recruitment efforts. This includes investigators and staff taking the time to understand the patient's personal circumstances that may well affect enrollment and follow-up.

5.6 PARTICIPANT WITHDRAWAL OR TERMINATION

5.6.1 REASONS FOR WITHDRAWAL OR TERMINATION

An investigator may discontinue study drug if, in the investigator's opinion, any clinical AE, laboratory abnormality, or other medical condition or situation occurs such that continued use would not be in the best interest of the participant. Additionally, participants are free to discontinue study drug at any time. Subjects who discontinue study drug will continue to be followed and study data collected through the end of the study.

Alternatively, participants are free to withdraw entirely from participation in the study at any time. Subjects who withdraw consent for study participation will not be followed and further study data will

not be collected. Every effort must be made to undertake protocol-specified safety follow-up procedures to capture SAEs, clinical outcomes, and AEs of special interest prior to study discontinuation.

If a subject wishes to withdraw from active participation in the study, sites should request permission to contact the patient at less frequent intervals (every 6 months or 12 months, depending on subject preference) via telephone or other HIPAA-compliant telehealth technology. If the subject declines this option, sites should request permission to contact the subject's physicians or family members for follow-up (if allowed by local regulations) and to periodically remotely review the subject's electronic medical record and claims data. If the subject declines this option, sites should ask permission for ongoing remote monitoring of the subject's status via the electronic medical record and claims data. If granted, permission for one or more of the above options should be documented in writing and retained in the study subject's file in a secure and confidential manner. Sites should contact subjects' physicians/family (as allowed by location regulations) and/or review all available medical records of such patients every 3 months (as per subject's follow up window) and make their best effort at ascertaining the subject's stroke and vital status. Regardless of the method chosen for modified follow-up, the appropriate CRFs should be completed every 3 months using available information from at least the electronic medical record. If there is no available follow-up information of any kind for 1 year, the subject should be reported as officially lost to follow-up.

At the end of the trial, publicly available vital records will be used to attempt to verify the vital status of any subjects withdrawn from the study or lost to follow-up.

5.6.2 HANDLING OF PARTICIPANT WITHDRAWALS OR TERMINATION

All randomized participants will be analyzed according to the intention-to-treat principle and therefore will be analyzed in the group they were assigned to regardless of premature discontinuation of study drug, withdrawal from the study, or changes in study treatment.

5.7 PREMATURE TERMINATION OR SUSPENSION OF STUDY

This study may be temporarily suspended or prematurely terminated if there is sufficient reasonable cause. Written notification, documenting the reason for study suspension or termination, will be provided by the suspending or terminating party to site investigators, NINDS, and the StrokeNet Data and Safety Monitoring Board (DSMB). If the study is prematurely terminated or suspended, the co-PIs will promptly inform the cIRB and other IRB/REBs with study oversight and will provide the reason(s) for the termination or suspension.

Circumstances that may warrant termination or suspension include but are not limited to:

- Determination of unexpected, significant, or unacceptable risk to participants.
- Insufficient compliance to protocol requirements.
- Data that are not sufficiently complete and/or evaluable.
- Demonstration of efficacy or futility (see Section 10.4.4).

The study may resume once concerns about safety, protocol compliance, and data quality are addressed and satisfy any concerns of NINDS, the DSMB, and the cIRB and other IRB/REBs with study oversight.

6 STUDY AGENT

6.1 STUDY AGENT AND CONTROL DESCRIPTION

6.1.1 ACQUISITION

Study drug and matching placebo will be supplied by the BMS-Pfizer Partnership. Study drug will be sent from BMS-Pfizer to the NCC Central Pharmacy, which will label the study drug bottles, distribute study drug to the sites (via a central depot for Canadian sites) as they are released to enroll, and resupply sites with study drug as recruitment progresses.

6.1.2 FORMULATION, APPEARANCE, PACKAGING, AND LABELING

Study treatments to be administered are active apixaban or matching placebo and active aspirin or matching placebo. Blinding will be provided by the use of the same color of formulations, same drug packaging, and same method of drug administration in every subject.

6.1.3 PRODUCT STORAGE AND STABILITY

Study product should be stored under controlled room temperature at 15°C to 25°C (59°F-77°F). The NCC Central Pharmacy must be notified of known temperature excursions beyond this range. Excursions must be reviewed by the study team before product that has experienced a known excursion may be used. Excursions must be reported immediately, preferably within 48 hours of occurrence to the NCC Central Pharmacy.

6.1.4 DISTRIBUTION

Local site pharmacies will distribute study drugs to enrolled participants every 3 months either in person or by mail depending on each site's local practice in accordance with local laws and regulations. Study drug will be provided in 90-day supply bottles, with a buffer of 10-day supply of extra pills to account for a delayed resupply visit. At the time of each resupply, subjects will return unused study drug to the investigator/coordinator.

6.1.5 DOSING

Active treatment will be either apixaban 5 mg or aspirin 81 mg. An adjusted dose of apixaban 2.5 mg will be used for subjects with ≥2 of the following: age ≥80 years, body weight ≤60 kg, or known serum creatinine ≥1.5 mg/dL. For Canadian sites only, an adjusted dose of apixaban 2.5 mg will be used for subjects with eCrCl 15-24 mL/min regardless of other factors, as per national regulatory requirements. Thus, there will be six possible study tablets: apixaban 5 mg (regular dose), apixaban 2.5 mg (adjusted dose), apixaban 5 mg placebo, apixaban 2.5 mg placebo, aspirin 81 mg, and aspirin placebo. This dosing schema is consistent with previous trials of stroke prevention in AF.[21,32] Aspirin will be used as the active control agent since it is the proven, standard-of-care antithrombotic drug recommended for patients with stroke.[24]

6.1.6 ADMINISTRATION

All subjects will be randomized to receive active treatment with either active apixaban or active aspirin. Study treatments will be supplied in a double-dummy fashion as aspirin 81 mg or matching placebo, and as apixaban 5 mg (2.5 mg for the adjusted dose) or matching placebo. Study participants will take 1 tablet of aspirin or placebo once daily by mouth from Bottle 1 and 1 tablet of apixaban or placebo twice daily by mouth from Bottle 2. Starting at the randomization visit and then at regular intervals thereafter until study completion, subjects will be provided with two high-density polyethylene pill bottles. Depending on the treatment assignment, Bottle 1 will contain placebo aspirin and Bottle 2 will contain active apixaban, or Bottle 1 active aspirin and Bottle 2 will contain placebo apixaban.

The first doses of study medication must be initiated within 48 hours of randomization.

If a dose of study drug is not taken at the scheduled time, the dose should be taken as soon as possible on the same day, except if less than 6 hours from the next dose, then the missed dose should be skipped and the usual schedule of administration should be resumed. The dose should not be doubled to make up for a missed dose.

6.1.7 LABORATORY MONITORING AND DOSE ADJUSTMENTS/INTERRUPTIONS

The U.S. FDA package insert for apixaban does not recommend regular monitoring of laboratory parameters such as creatinine or liver function tests. Thus, such tests are not required as part of this study in the United States. For Canadian sites only, laboratory determination of renal function must occur at least once per year as part of standard care, as per national regulatory requirements. All site investigators and study coordinators will be educated, via training at the start of the study and regular study newsletters, that a reduced dose of apixaban study drug is required if subjects meet the dose adjustment criteria. The MOP provides details on switching doses if necessary.

6.1.8 TRACKING OF DOSE

Study drug adherence will be assessed using pill counts at each study visit. For subjects who have intentionally stopped study drug, the reason for discontinuation or temporary interruption will be collected (e.g., elective procedure, new contraindication to treatment, etc).

6.2 STUDY DRUG ACCOUNTABILITY PROCEDURES

Upon receipt of the study drug, the site pharmacist or designee will inspect the study drug supply and confirm receipt of the study drug in the WebDCU™, the study's clinical trial management system. The pharmacist or pharmacy designee at each clinical site must also document in WebDCU™ study drug dispensing, return, and destruction. Drug accountability records and storage temperature logs may be inspected by the study monitor or subjected to inspection by relevant authorities.

7 STUDY PROCEDURES AND SCHEDULE

7.1 STUDY PROCEDURES/EVALUATIONS

7.1.1 STUDY-SPECIFIC PROCEDURES

Medical history:
- Obtained at baseline.
- Should focus on inclusion/exclusion criteria and vascular comorbidities.
- Can be obtained from up-to-date medical records and/or interview.

Vital signs:
- Obtained at baseline.
- Should include height and weight to allow calculation of the body mass index (BMI).

NIHSS:
- Obtained at baseline.

QVSFS:
- Validated screening tool for identifying strokes.[34]
- Obtained at the randomization visit and all follow-up visits.
- Should be obtained via telephone if subjects cannot make in-person study visits.
- Should be obtained from a proxy if necessary.
- Medical records should be reviewed to confirm reports of possible strokes.

mRS:
- Obtained at baseline and at the q6-month follow-up visits by a certified investigator.
- Should be obtained via telephone if subjects cannot make an in-person q6-month study visit.
- Should be obtained from a proxy if necessary.

Blood sample:
- Obtained at baseline and sent to Laboratory Core.
- See MOP for instructions for collection and shipment.

PROMIS scales:
- Global Health Form and Physical Function Short Form.[35]
- Obtained at the 12-month follow-up visit from subjects who are able to complete the forms themselves.

Clinical outcome events:
- Obtained at all follow-up visits.
- Should be obtained via telephone or other HIPAA-compliant telehealth technology if subjects cannot make in-person study visits.
- Should be obtained from a proxy or medical records if necessary.

SAEs and AEs of special interest:

- Obtained at all follow-up visits until 30 days after permanent study drug discontinuation.
- Should be obtained via telephone or other HIPAA-compliant telehealth technology if subjects cannot make in-person study visits.
- Should be obtained from a proxy or medical records if necessary.

Medication adherence:

- Pill counts should be documented at each follow-up visit.
- Reasons for discontinuation/interruption should be documented if applicable.

Concomitant medications:

- Subjects should be asked about antiplatelet and anticoagulant drugs at baseline and each follow-up visit.

7.1.2 STANDARD-OF-CARE STUDY PROCEDURES

Each of the tests below, which are all required to establish a diagnosis of ESUS, should ideally be performed after the index stroke. If necessary, tests that were done no earlier than 3 months prior to the index stroke can be used to establish ESUS and eligibility for consenting. Note that 12-lead ECGs or TTEs done before the index stroke cannot be used to assess atrial cardiopathy and qualification for randomization.

Brain imaging:

- Either CT or MRI is acceptable.

Vascular imaging:

- Acceptable modalities include ultrasound, CTA, MRA, or catheter angiogram.
- Both the cervical and intracranial cerebral circulation must be evaluated.

12-lead ECG:

- The first technically adequate ECG done after stroke onset should be used.
- See MOP for instructions on uploading a copy of the ECG to WebDCU™.

Continuous heart-rhythm monitoring:

- ≥24 hours required.
- Either hospital telemetry or formal Holter monitoring is acceptable.
- Hospital telemetry does not require an automated AF detection algorithm if reviewed by investigators or other clinical staff. Review of ECG or heart-rhythm monitoring by an attending cardiologist at the site, if it occurs, is the gold standard for defining whether AF is present. Eligible patients must not be randomized until any heart-rhythm monitoring performed prior to the date of randomization is interpreted, to avoid randomizing patients with AF found before randomization.

**Echocardiogram:**	• Either TTE or TEE is acceptable to determine ESUS.<br>• The first technically adequate TTE done after stroke onset should be used to assess for atrial cardiopathy.<br>• See MOP for instructions on sending echocardiogram images.
**Laboratory tests:**	• Chemistry: creatinine.<br>• CBC: white blood cell count, hemoglobin, hematocrit, platelet count.<br>• Pregnancy test, if applicable.

7.2 STUDY SCHEDULE

7.2.1 SCREENING

Study subjects are expected to be recruited from both the inpatient setting, such as from an acute stroke service, and the outpatient setting, such as from a stroke clinic. Ideally, the trial should be introduced to a potential subject by individuals who, by virtue of their position, would normally have access to the potential subject's confidential information, for example the subject's primary care physician or member of current treating team. Potential subjects who show an interest in study participation should be asked for their permission to be approached by the study team. The person who introduced the study to the potential subject should document this permission in the medical record.

If a study investigator was a treating physician for a potential subject who expresses interest in the study, or if a treating physician introduces the potential subject to the trial and that potential subject provides permission to be approached by study investigators or coordinators, that subject can be contacted about the trial by phone by a study team member.

If a potential subject was not introduced to the trial by a treating physician who asked for permission for ARCADIA investigators or coordinators to approach the patient, the steps below must be followed in sequence before a patient can be called about ARCADIA:

1. If required at a site, a waiver of HIPAA authorization must be in place to screen medical records to identify potentially eligible subjects.
2. Permission to approach the patient must be obtained from the potential subject's primary care physician or neurologist.
3. Recruitment materials must be mailed to the potential subject, along with instructions to return a postcard or make a telephone call indicating that they do not wish to participate.
4. If the potential subject does not indicate refusal to participate, the study team may call them about the study after the time indicated in the instructions.

The investigators at each site will be required to maintain a screen failure log for patients with ESUS who are found ineligible to participate in the study, documenting the patients' age, demographics, and the reason(s) for exclusion from the current study. The study coordinator at each site is required to enter the screen failure log data into WebDCU™ on a monthly basis.

Screening for Atrial Cardiopathy

Among patients who provide consent for trial participation, only those who meet at least one atrial cardiopathy criterion will be randomized. A hotline coordinated via the Eligibility Core will be available to help with reviewing screening data and determining eligibility.

- To determine whether a patient meets the ECHO criterion, the site investigator will determine from the report of the first available post-stroke TTE with a reported left atrial diameter whether the patient has severe left atrial enlargement, defined as left atrial diameter index ≥3 cm/m^2. If possible, echocardiograms should be sent via procedures outlined in the MOP to the Echocardiography Core at Columbia for additional measurements to be used in secondary analyses.

- To determine whether a patient meets the ECG criterion, the first ECG done as part of the standard stroke evaluation will be used. A copy of this ECG will be sent via procedures outlined in the MOP to the ECG Core at Wake Forest for standardized measurement of $PTFV_1$. The ECG Core will enter the assessment of $PTFV_1$ into WebDCU™ within 2 business days of receipt of the ECG so that eligibility can be determined.

- To determine whether a patient meets the BNP criterion, a blood sample will be sent via procedures outlined in the MOP to the study Laboratory Core at Columbia for NT-proBNP measurement. The Laboratory Core will enter the NT-proBNP measurement into WebDCU™ within 2 business days of receipt of the blood sample so that eligibility can be determined.

Baseline Visit (Day 0-180 after stroke onset)

- Ensure that required standard-of-care tests have been performed (see Section 7.1.2).
- Review medical history, medications, and standard-of-care tests to confirm eligibility based on inclusion/exclusion criteria (see Sections 5.1 and 5.2).
- Obtain informed consent of potential participant verified by signature on written informed consent form.
- Assess mRS score. If possible, record vital signs and NIHSS score. Collect baseline information such as medical history, imaging results, and Medicare information (beneficiary name, DOB, and ID) if applicable. All data will be entered into WebDCU™ except for the Medicare information which will be entered into a separate HIPAA-compliant electronic database (REDCap) and kept separate from the main WebDCU™-collected data for an added level of protection.
- Collect blood samples for NT-proBNP assay and potential future use and ship to Laboratory Core (see MOP).
- Upload a copy of the first technically adequate post-stroke 12-lead ECG to WebDCU™ (see MOP).
- If possible, send a copy of images of the first technically adequate post-stroke TTE to the Echocardiography Core (see MOP).
- Schedule Randomization Visit. This visit can occur later during the index hospitalization or at a subsequent visit (randomization must occur on or before Day 180 after stroke onset).

7.2.2 RANDOMIZATION

Randomization should occur as close to the first administration of study drug as possible. In general, randomization can occur as early as post-stroke day 3. However, randomization must be delayed until at least post-stroke day 14 for patients with severe strokes (initial NIHSS score ≥11), hemorrhagic transformation of the index stroke, or uncontrolled hypertension. For all strokes, randomization must occur no later than post-stroke day 180. The calendar day (12:00 a.m. through 11:59 p.m.) of stroke onset is considered post-stroke day 0. If the time of onset of the stroke is unclear, the day of the first presentation for medical care will be considered post-stroke day 0.

Randomization Visit (Day 3-180 after stroke onset)

- Rescreen participants immediately prior to randomization. Review medical history, medications, QVSFS, and vital signs (if possible). Participants must continue to meet all inclusion and exclusion criteria at the time of randomization. None of the tests specified in Section 5.1 need to be repeated as part of the rescreening process; investigators should simply screen for interval events that would make the patient ineligible (e.g., development of spontaneous intracranial hemorrhage, AF, recurrent stroke). If any of the tests in Section 5.1 have been repeated as part of standard clinical care, those results should be reviewed to ensure continued eligibility.
- Once it is determined that a patient meets all the randomization criteria, the site investigator or their designee will log on to WebDCU™ to enter the required randomization data. The database will generate the randomization which will correlate to the appropriate study drug to be retrieved from the study pharmacy.
- Those who are not considered eligible for randomization due to the absence of all of the three atrial cardiopathy biomarkers may be followed in the future as part of ancillary studies, for clinical outcomes through telephone calls and review of medical records and insurance claims. Permission for follow up of this group is included in the study consent form.
- If randomization cannot be performed during an in-person visit, randomization can be performed during a visit using telephone or other HIPAA-compliant telehealth technology (please see MOP for details).

7.2.3 FOLLOW-UP

Follow-Up Visit 1 (30 ± 7 days after randomization)

- Contact participants via telephone or other HIPAA-compliant telehealth technology and assess eligibility for study medication, study drug safety, clinical outcome events, SAEs and AEs of special interest as indicated depending on patient's follow-up and study treatment status, medication adherence, and concomitant medications.

Follow-up Visit 2 (90 days ± 5 days after randomization)

- Schedule in-person visit (or televisit if necessary) to assess eligibility for study medication, study drug safety, clinical outcome events, SAEs and AEs of special interest as indicated depending on patient's follow-up and study treatment status, medication adherence, and concomitant medications. Televisits must be via telephone or other HIPAA-compliant telehealth technology.

Follow-up Visit 3 (180 days ± 5 days after randomization)

- Schedule in-person visit (or televisit if necessary) to assess eligibility for study medication, study drug safety, mRS, clinical outcome events, SAEs and AEs of special interest as indicated depending on patient's follow-up and study treatment status, medication adherence, and concomitant medications. Televisits must be via telephone or other HIPAA-compliant telehealth technology.

Follow-up Visit 4 (270 days ± 5 days after randomization)

- Schedule in-person visit (or televisit if necessary) to assess eligibility for study medication, study drug safety, clinical outcome events, SAEs and AEs of special interest as indicated depending on patient's follow-up and study treatment status, medication adherence, and concomitant medications. Televisits must be via telephone or other HIPAA-compliant telehealth technology.

Follow-up Visit 5 (360 days ± 5 days after randomization)

- Schedule in-person visit (or televisit if necessary) to assess eligibility for study medication, study drug safety, mRS, clinical outcome events, SAEs and AEs of special interest as indicated depending on patient's follow-up and study treatment status, medication adherence, concomitant medications, ambulatory status, and PROMIS quality-of-life scales. Televisits must be via telephone or other HIPAA-compliant telehealth technology.

Follow-up Visits 6 and beyond (through completion of subject participation, minimum 540 days)

- Schedule in-person visits (or televisits if necessary) every 180 days ± 5 days to assess eligibility for study medication, study drug safety, mRS, clinical outcome events, SAEs and AEs of special interest as indicated depending on patient's follow-up and study treatment status, medication adherence, and concomitant medications. Televisits must be via telephone or other HIPAA-compliant telehealth technology. Medication resupply will need to occur at the 3-month mid-point. Sites are also required every 3 months to assess clinical outcome events, as well as SAEs and AEs of special interest as indicated depending on patient's follow-up and study treatment status. This can be in-person or via telephone or other HIPAA-compliant telehealth technology.

7.2.4 FINAL STUDY VISIT

At the end of the study, or at the time of subject withdrawal from the study or premature treatment discontinuation, the site investigator will be responsible for choosing and initiating appropriate antithrombotic therapy. Subjects should be treated at least with aspirin (or open-label anticoagulation if they have documented AF). There will also be a close-out telephone visit 30 days after the end of study drug treatment to ascertain SAEs, clinical outcome events, and AEs of special interest (details in MOP).

7.2.5 UNSCHEDULED VISIT

An unscheduled visit should be arranged for SAEs, clinical outcome events, AEs of special interest, or other developments that in the opinion of the site investigator necessitate an in-person evaluation to ensure the subject's safe ongoing participation in the study.

7.2.6 SCHEDULE OF EVENTS TABLE

Procedure	B	R	30 ±7 days*	90 ±5 days	180 ±5 days	270 ±5 days	360 ±5 days	q180 ±5 days[#] afterward	Close out[$]
Eligibility check^	x	x	x	x	x	x	x	x	
Consent	x								
Randomization form		x							
Medical history	x	x							
QVSFS		x	x	x	x	x	x	x	
Modified Rankin Scale	x				x		x	x	
Vital signs	x	x							
NIHSS	x								
Brain imaging	o								
Vascular imaging	o								
12-lead ECG	o								
≥24 hrs cardiac monitoring	o								
Echocardiogram	o								
Serum chemistry	o						‖		
Complete blood count	o								
Pregnancy test, if applicable	o								
Blood sample to core lab	x								
ECG to core lab	x								
Echo to core lab	x								
PROMIS Global Health							x		
PROMIS Phys. Func. Short							x		
Ambulatory status							x		
Safety assessment			x	x	x	x	x	x	x
Study drug safety			x	x	x	x	x	x	
Medication adherence			x	x	x	x	x	x	
Medication resupply				x	x	x	x	x	
Concomitant med.	x	x	x	x	x	x	x	x	

B = Baseline visit; R = Randomization visit; QVSFS = Questionnaire for Verification of Stroke-Free Status.
* Telephone visit.
[#] This visit includes medication resupply at mid-point (3 months) which will require assessing QVSFS, clinical outcomes, as well as SAEs and AEs of special interest as indicated depending on patient's follow-up and study treatment status. These should occur within ±5 days of the 3-month mark.
[$] Telephone visit 30 days after study drug discontinuation.
^ After randomization, eligibility check will involve ensuring continued suitability for study drug administration.
o Cost billed as standard of care.
x Cost covered by study.
[‖] For Canadian sites only, laboratory determination of renal function must be performed at least once per year as part of standard care, as per national regulatory requirements.

7.3 CONCOMITANT AND PROHIBITED MEDICATIONS

7.3.1 ANTIPLATELET MEDICATIONS

If an antiplatelet agent is newly indicated after randomization (e.g., aspirin or clopidogrel after implantation of a coronary artery stent), then study drug must be stopped until the antiplatelet agent is stopped.

For subjects who were receiving antiplatelet therapy prior to their qualifying stroke, there is no high-quality evidence to support switching to another antiplatelet agent empirically or based on the results of platelet resistance assays.[24] Therefore, subjects receiving aspirin, clopidogrel, or aspirin/dipyridamole should be considered eligible for this trial and randomization to either aspirin or apixaban monotherapy. All baseline antiplatelet therapy must be stopped after randomization; the last dose of antiplatelet therapy allowed is on the day before randomization.

If the site investigator feels that a short course of dual antiplatelet therapy is indicated, randomization cannot occur until after this course is completed.

7.3.2 ANTICOAGULANT MEDICATIONS

The first dose of study drug cannot be given until at least 12 hours after the last dose of an anticoagulant (heparin, enoxaparin, etc), even if at a prophylactic dose. Guidelines from the AHA/ASA recommend prophylactic-dose anticoagulation for “treatment of immobilized subjects to prevent DVT.”[36] For immobilized subjects who are receiving prophylactic-dose anticoagulation per these guidelines, randomization should be performed at a time such that study drug is not started until after discontinuation of prophylactic-dose anticoagulation.

After randomization, if open-label anticoagulant therapy is newly indicated at a full treatment dose, such as for newly diagnosed venous thromboembolism, or at a prophylactic dose during an intercurrent hospitalization and/or a rehabilitation stay, then study drug must be stopped until open-label anticoagulation is stopped.

7.3.3 STRONG INDUCERS AND INHIBITORS OF CYP3A4 AND P-gp

The concomitant use of strong inhibitors of CYP3A4 and P-gp, such as azole-antimycotics (e.g., ketoconazole, itraconazole, voriconazole, or posaconazole) or HIV protease inhibitors (e.g., ritonavir), can increase apixaban exposure. The concomitant use of strong inducers of CYP3A4 and P-gp (e.g., rifampin, phenytoin, carbamazepine, phenobarbital, or St. John’s Wort) can reduce apixaban exposure. Concomitant use of strong inhibitors or inducers of CYP3A4 and P-gp is discouraged for U.S. sites. Concomitant use of strong inhibitors or inducers of CYP3A4 and P-gp is prohibited for Canadian sites, as per national regulatory requirements.

7.4 DETECTION AND MANAGEMENT OF ATRIAL FIBRILLATION

The use of continuous heart-rhythm monitoring after randomization is allowed. Subjects who, as part of standard-of-care follow-up/testing after randomization, manifest AF of any duration, as determined by

the judgment of the site investigator and treating physicians, should be switched to open-label anticoagulant therapy per the discretion of the site investigator and treating physicians. We recommend but do not mandate switching to open-label apixaban using the same dosing as the study protocol. These subjects will continue to be followed for outcome events in the study according to the intention-to-treat paradigm.

7.5 INTERRUPTION OF STUDY DRUG FOR ELECTIVE INVASIVE PROCEDURES

Unblinding will not be performed for elective procedures. Investigators will be educated and reminded that subjects have a 50% chance of being on anticoagulation, and investigators will be provided with and referred to guidelines from the American Academy of Neurology on periprocedural management of antithrombotic medications in subjects with ischemic cerebrovascular disease.[37] For further reference, the FDA label for apixaban states: "ELIQUIS should be discontinued at least 48 hours prior to elective surgery or invasive procedures with a moderate or high risk of unacceptable or clinically significant bleeding. ELIQUIS should be discontinued at least 24 hours prior to elective surgery or invasive procedures with a low risk of bleeding or where the bleeding would be non-critical in location and easily controlled." Site investigators and study coordinators will be educated about these guidelines through training at study initiation and through regular study newsletters.

7.6 MANAGEMENT OF BLEEDING

In cases of bleeding that do not meet the definition of serious bleeding, subjects should be advised to not take further doses of study drugs until the bleeding has stopped and the investigator judges that the benefits of resuming study drug outweigh the risk of recurrent bleeding.

The following steps should be taken in all cases of serious bleeding:

- Further doses of study drug should be held until the bleeding is controlled and the investigator judges that the benefits of resuming study drug outweigh the risk of recurrent bleeding.
- Standard measures should be taken to control and mitigate the effects of bleeding, such as local control of the bleeding source if possible and administration of intravenous fluids and blood products as necessary.

If it is considered likely that bleeding cannot be managed with only the steps above, and that measures specific to reversal of apixaban are required, treating physicians and/or site investigator can perform unblinding by calling the study hotline. The MOP provides detailed guidance on management of serious bleeding in subjects assigned to apixaban.

7.7 MANAGEMENT OF ACUTE ISCHEMIC STROKE IN SUBJECTS ON STUDY DRUG

If intravenous thrombolysis is being considered for the acute treatment of recurrent ischemic stroke, the treating physicians and/or site investigators can call the study hotline for unblinding. After unblinding, subjects assigned to aspirin can be treated with intravenous thrombolysis if indicated per each site's standard practice.

Subjects assigned to apixaban may be at an increased risk of bleeding if treated with intravenous thrombolysis unless all of the following conditions are met:

- The subject or surrogate can confirm that no study drug has been taken for the past 48 hours;
- The subject's renal function is normal (GFR ≥60);
- The subject's INR and PTT values are normal;
- Intravenous thrombolysis is otherwise indicated per the site's standard practice.

Unblinding should only occur for subjects who would be eligible for treatment only if they were on aspirin and not on apixaban. Unblinding is discouraged for subjects who are not eligible regardless of being on aspirin or apixaban, or subjects who meet all of the criteria above and may be able to receive thrombolysis while being on apixaban.

7.8 VASCULAR RISK FACTOR MANAGEMENT

Investigators will be provided with and referred to the most recent guidelines from the AHA/ASA on secondary stroke prevention.[24] Site investigators and study coordinators will be educated about these guidelines through training at study initiation and through regular study newsletters.

7.9 PARTICIPANT ACCESS TO STUDY DRUG AFTER STUDY CLOSURE

Study drug will not be provided free of charge to participants after study closure. Similarly, participants who switch to open-label anticoagulant therapy due to interval diagnosis of AF will no longer receive study drug free of charge.

If a site identifies an event as a possible primary or secondary efficacy outcome, the subject will stop study medication. If the adjudication committee determines the event does not meet the primary or secondary efficacy outcome definition, the subject may resume treatment at the discretion of the site investigator and/or treating physician.

8 ASSESSMENT OF SAFETY

8.1 SPECIFICATION OF SAFETY PARAMETERS

Primary safety endpoints: (A) Symptomatic intracranial hemorrhage (including symptomatic hemorrhagic transformation of an ischemic stroke), and (B) major hemorrhage other than intracranial hemorrhage.

Secondary safety endpoints: All-cause mortality.

The following exploratory endpoints will also be recorded: AF, any intracranial hemorrhage, major hemorrhage including any intracranial hemorrhage, symptomatic hemorrhagic transformation of an ischemic stroke, transient ischemic attack, myocardial infarction, minor hemorrhage, systemic embolism, symptomatic deep venous thrombosis, symptomatic pulmonary embolism, ischemic vascular death, and hemorrhagic vascular death. The endpoint of any intracranial hemorrhage will be subclassified as: 1) symptomatic versus asymptomatic, and 2) consisting of hemorrhagic transformation of the index brain infarct versus not.

8.1.1 DEFINITION OF ADVERSE EVENTS

An AE is defined as any new untoward medical occurrence or worsening of a preexisting medical condition in a clinical investigation participant administered study drug and that does not necessarily have a causal relationship with this treatment. An AE can therefore be any unfavorable and unintended sign (such as an abnormal laboratory finding), symptom, or disease temporally associated with the use of investigational product, whether or not considered related to the investigational product. Adverse events can be spontaneously reported or elicited during open-ended questioning, examination, or evaluation of a subject. For the purposes of this trial, only AEs that meet the definition of serious, clinical outcomes, and AE of special interest will be reported.

8.1.2 DEFINITION OF SERIOUS ADVERSE EVENTS

An SAE is any untoward medical occurrence that at any dose:

- results in death;
- is life-threatening (defined as an event in which the participant was at risk of death at the time of the event; it does not refer to an event which hypothetically might have caused death if it were more severe);
- requires inpatient hospitalization or causes prolongation of existing hospitalization;
- results in persistent or significant disability/incapacity;
- is a congenital anomaly/birth defect;
- is an important medical event (defined as a medical event(s) that may not be immediately life-threatening or result in death or hospitalization but, based upon appropriate medical and scientific judgment, may jeopardize the subject or may require intervention [e.g., medical, surgical] to prevent one of the other serious outcomes listed in the definition above. Examples of such events include, but are not limited to, intensive treatment in an emergency room or at home for allergic bronchospasm; blood dyscrasias or convulsions that do not result in hospitalization.)
- results in suspected transmission of an infectious agent (e.g., pathogenic or nonpathogenic) via the study drug.

The definition of SAE excludes the following hospitalizations:

- A visit to the emergency room or other hospital department < 24 hours, that does not result in admission (unless considered an important medical or life-threatening event);
- Elective surgery, planned prior to signing consent;
- Admissions as per protocol for a planned medical/surgical procedure;
- Routine health assessment requiring admission for baseline/trending of health status (e.g., routine colonoscopy);
- Medical/surgical admission other than to remedy ill health and planned prior to entry into the study (appropriate documentation is required in these cases);
- Admission encountered for another life circumstance that carries no bearing on health status and requires no medical/surgical intervention (e.g., lack of housing, economic inadequacy, caregiver respite, family circumstances, administrative reason).

A life-threatening adverse event is defined as an event in which the participant was at risk of death at the time of the event; it does not refer to an event that hypothetically might have caused death if it were more severe.

Important medical events that may not result in death, be life-threatening, or require hospitalization may be considered serious when, based upon appropriate medical judgment, they may jeopardize the patient or subject and may require medical or surgical intervention to prevent one of the outcomes listed in this definition. Examples of such medical events include allergic bronchospasm requiring intensive treatment in an emergency room or at home, blood dyscrasias or convulsions that do not result in inpatient hospitalization, or the development of drug dependency or drug abuse. Other study-specific SAEs are defined in the MOP.

8.1.3 ADVERSE EVENTS OF SPECIAL INTEREST

Pregnancy, overdose, potential drug-induced liver injury, cancer, and COVID-19 are AEs of special interest and will be assessed and reported.

Potential or suspected cases of liver injury:
This includes but is not limited to liver test abnormalities, jaundice, hepatitis, or cholestasis.

Pregnancy:
If, following initiation of the investigational product, it is subsequently discovered that a study participant is pregnant or may have been pregnant at the time of investigational product exposure, including during at least 5 half-lives after product administration, the investigational product will be permanently discontinued in an appropriate manner (e.g., dose tapering if necessary for participant).

Protocol-required procedures for study discontinuation and follow-up must be performed on the participant. Follow-up information regarding the course of the pregnancy, including perinatal and neonatal outcome and, where applicable, offspring information must be reported on a Pregnancy Surveillance Form.

Any pregnancy that occurs in a female partner of a male study participant will also be reported. Information on this pregnancy will be collected on the Pregnancy Surveillance Form. In order for the investigators to collect any pregnancy surveillance information from the female partner, the female partner must sign an informed consent form for disclosure of this information.

Overdose:
An overdose is defined as the accidental or intentional administration of any dose of a product that is considered both excessive and medically important.

COVID-19:
COVID-19 is defined as the patient reporting or the medical record indicating that the patient was tested for SARS-CoV-2 and found to be positive.

8.1.4 UNANTICIPATED PROBLEMS AND UNANTICIPATED EVENTS

The Office for Human Research Protections (OHRP) considers *unanticipated problems* (UPs) involving risks to participants or others to include, in general, any incident, experience, or outcome that meets **all** of the following criteria:

- Unexpected in terms of nature, severity, or frequency given (a) the research procedures that are described in the protocol-related documents, such as the cIRB/IRB/REB-approved research protocol and informed consent document; and (b) the characteristics of the participant population being studied;
- Related or possibly related to participation in the research ("possibly related" means there is a reasonable possibility that the incident, experience, or outcome may have been caused by the procedures involved in the research); and
- Suggests that the research places participants or others at a greater risk of harm (including physical, psychological, economic, or social harm) than was previously known or recognized.

This study will use the OHRP definition of UP.

Unanticipated events include UPs, but may also include other events that do not rise to the level of UPs as outlined above. These include protocol deviations or other unexpected problems that do not necessarily pose a safety concern, and will be reported according to cIRB/IRB/REB requirements and StrokeNet Standard Operating Procedures as defined in detail in the MOP.

8.2 CLASSIFICATION OF AN ADVERSE EVENT

8.2.1 SEVERITY OF EVENT

The severity of SAEs, clinical outcome events, and AEs of special interest will be reported using the grading system outlined in the NCI Common Terminology Criteria for Adverse Events Version 4.03 (CTCAE). The CTCAE provides a grading (severity) scale for each AE term and AEs are listed alphabetically within categories based on anatomy or pathophysiology. The CTCAE (v4.03) displays Grades 1-5 with unique clinical descriptions of severity for each AE based on this general guidance:

CTCAE Severity Grading Summary	
Grade 1:	Mild AE
Grade 2:	Moderate AE
Grade 3:	Severe or Disabling AE
Grade 4:	Life-Threatening AE
Grade 5:	Death related to AE

The complete definitions of these grades are:

- Grade 1: Mild; asymptomatic or mild symptoms; clinical or diagnostic observations only; intervention not indicated AE.

- Grade 2: Moderate; minimal, local or noninvasive intervention indicated; limiting age-appropriate instrumental activities of daily living (preparing meals, shopping for groceries or clothes, using the telephone, managing money, etc.).
- Grade 3: Severe or medically significant but not immediately life-threatening; hospitalization or prolongation of hospitalization indicated; disabling; limiting self-care activities of daily living (bathing, dressing and undressing, feeding self, using the toilet, taking medications, and not bedridden).
- Grade 4: Life-threatening consequences; urgent intervention indicated.
- Grade 5: Death related to AE.

8.2.2 RELATIONSHIP TO STUDY AGENT

For each SAE, clinical outcome event, and AE of special interest, the relationship to the study treatment will be assessed and documented using the following algorithm:

Algorithm to Determine Relatedness of Adverse Event to Study Agent	
**Not Related**	The temporal relationship between treatment exposure and the adverse event is unreasonable or incompatible and/or adverse event is clearly due to extraneous causes (e.g., underlying disease, environment)
**Unlikely**	**Must have both of the following** 2 conditions, but may have reasonable or only tenuous temporal relationship to intervention:
	1. Could readily have been produced by the subject's clinical state, or environmental or other interventions.<br>2. Does not follow known pattern of response to intervention.
**Reasonable Possibility**	**Must have at least 2** of the following 3 conditions:
	1. Has a reasonable temporal relationship to intervention.<br>2. Could not readily have been produced by the subject's clinical state or environmental or other interventions.<br>3. Follows a known pattern of response to intervention.
**Definitely**	**Must have all 3** of the following conditions:
	1. Has a reasonable temporal relationship to intervention.<br>2. Could not possibly have been produced by the subject's clinical state or have been due to environmental or other interventions.<br>3. Follows a known pattern of response to intervention.

8.2.3 EXPECTEDNESS

The independent Medical Safety Monitor (MSM) will be responsible for determining whether an SAE is **expected** or **unexpected**. An SAE will be considered unexpected if the nature, severity, or frequency of the event is not consistent with the risk information previously described for the study agent.

8.3 TIME PERIOD AND FREQUENCY FOR EVENT ASSESSMENT AND FOLLOW-UP

The site investigator will regularly assess and report all clinical outcome events occurring after randomization until the last day of study participation. The site investigator will regularly assess and report all SAEs other than clinical outcome events, as well as all AEs of special interest, occurring after

randomization until 30 days after permanent study drug discontinuation. All such events will be captured on the appropriate CRF and entered into WebDCU™. Information to be collected includes event description, time of onset, clinician's assessment of severity, relationship to study product (assessed only by those with the training and authority to make a diagnosis), time of resolution/stabilization of the event, a description of the event, relevant history, and concomitant medications/procedures. All clinical outcome events, and all reportable SAEs and AEs of special interest, will be followed for outcome information until resolution or until 30 days after the subject's participation in the study ends. The information in WebDCU™ will be updated as more information becomes available.

8.4 REPORTING PROCEDURES

8.4.1 ADVERSE EVENT REPORTING

This study is being conducted as an investigator-initiated, NINDS-funded protocol. The FDA has granted a waiver for an investigational new drug (IND) application. Thus, no formal safety reporting to FDA will be done.

Only AEs that meet the definition of an SAE, clinical outcome event, or AE of special interest will be recorded and reported per the reporting timelines. All reporting will be done in accordance with StrokeNet Standard Operating Procedures for Safety Monitoring and Reporting as outlined in the administrative documents available on the website (https://www.nihstrokenet.org/docs/default-source/default-document-library/adm13-safety-monitoring-and-reporting-12-19-16.pdf?sfvrsn=0).

8.4.2 SAFETY REPORTING

Site Responsibilities

Site investigators or their designees must report SAEs, clinical outcome events, and AEs of special interest that occur during the time periods established in Section 8.3 through WebDCU™ within 24 hours of site awareness of the event. The investigators are required to provide relevant information such as a description of the event, date/time of onset and resolution, severity, suspected relationship to the study treatment, and action taken. Supporting documentation of the event should be provided as soon as possible. Additional supporting documentation may be requested by the NCC and should also be provided as soon as possible.

All clinical outcome events, whether related or not related to study drug, must be collected from the time of randomization until the end of the study. All SAEs other than clinical outcome events, and AEs of special interest, whether related or not related to study drug, must be collected from the time of randomization until 30 days after permanent study drug discontinuation.

NDMC and NCC Responsibilities (Sites should ignore these two paragraphs)

All SAEs and AEs of special interest that occur during the time periods established in Section 8.3 will be reported to BMS via a Safety Report generated in WebDCU™ for review by their Global Pharmacovigilance group (GPV&E) in accordance with global regulatory reporting requirements. Such SAEs and AEs of special interest, whether related or unrelated to the study drug, must be reported to BMS by NDMC within 72 hours. These events must be recorded on an approved form and sent to the BMS SAE Email Address: Worldwide.Safety@BMS.com, SAE Fax Number: 609-818-3804, or other address as provided by BMS. If only limited information is initially available, follow-up reports are required. Note: Follow-up safety reports should include the same investigator term(s) initially reported. If an ongoing event changes in its intensity or relationship to study drug or if new information becomes available, a follow-up safety report should be sent within 72 hours to the BMS (or designee) using the same procedure used for transmitting the initial report. The NCC will reconcile the clinical database with safety events transmitted to BMS GPV&E. Reconciliation will occur every 3 months and once just prior to database lock/final study report. The investigator will request a safety data reconciliation report from aepbusinessprocess@bms.com or other address provided by BMS. BMS GPV&E will email, upon request from the investigator, the GPV&E reconciliation report. The data elements listed on the GPV&E safety data reconciliation report will be used for case identification purposes. If the investigator determines a case was not transmitted to BMS GPV&E, the case will be sent immediately.

Via a separate safety monitoring mechanism, the MSM will conduct an independent review of each SAE to determine its seriousness, relationship to the study treatment, and expectedness. If the MSM adjudicates the event to be serious, unexpected, and study related, the event will be reported expeditiously to the cIRB and other IRB/REBs with study oversight and to Health Canada according to local regulations via a Safety Report generated in WebDCU™.

8.4.3 UNANTICIPATED EVENT REPORTING

Clinical sites will report unanticipated events, including UPs and protocol deviations, in the WebDCU™ per the guidelines listed in the MOP. Unanticipated events that are unexpected, related to study participation, and place the subject/others at increased risk will require prompt reporting to the cIRB and other IRB/REBs with study oversight and to Health Canada. All other unanticipated events which do meet the above criteria will be reported to the cIRB and other IRB/REBs with study oversight at the time of continuing review.

8.5 STUDY OVERSIGHT

Safety oversight will be under the direction of the NIH StrokeNet DSMB, which is composed of individuals with the appropriate expertise in overseeing stroke clinical trials. The DSMB will meet at least semiannually to assess safety data on each arm of the study. The DSMB will review data quality and completeness, monitor fidelity to the study protocol, review the adequacy of participant recruitment and retention, review SAEs, clinical outcome events, and AEs of special interest and make recommendations to the NINDS and the study co-PIs concerning trial continuation, modification, or conclusion.

8.6 STUDY HALTING RULES

The MSM is responsible for ongoing monitoring of reports of SAEs by the clinical centers within 72 hours to ensure Good Clinical Practices (GCP) and to identify safety concerns quickly. The MSM may suggest protocol modifications to prevent the occurrence of particular SAEs, such as modifying the protocol to require frequent measurement of laboratory values predictive of the event or to improve expeditious identification of SAEs. To minimize bias, the MSM will evaluate SAEs blinded to treatment assignment. The NDMC will prepare regular reports concerning SAEs and submit them to the DSMB. In the event of unexpected SAEs or an unduly high rate of SAEs, the MSM will promptly contact the co-PIs and the NINDS Program Official and, if applicable, the NINDS DSMB liaison, who will notify the DSMB Chair. The DSMB will convene an ad hoc meeting by teleconference or in writing as soon as possible. The DSMB will provide recommendations for continuing or halting the study to the NIH and the study co-PIs.

9 CLINICAL MONITORING

Clinical site monitoring is conducted to ensure that the rights and well-being of human subjects are protected, that the reported trial data are accurate, complete, and verifiable, and that the conduct of the trial is in compliance with the currently approved protocol/amendment(s), with GCP, with applicable FDA regulations (21 CFR 312), and with the FDA's "Guidance for Industry Oversight of Clinical Investigations — A Risk-Based Approach to Monitoring."

- Monitoring for this study will be performed by the NDMC centrally, on site, and remotely.
- Per the study's monitoring plan, monitoring will include a combination of **on-site monitoring** (to verify data entered into the WebDCU™ database against source documents and query inaccuracies between the source documents and WebDCU™ database), **remote monitoring** (source document verification, including verification of written consent, may be performed remotely by reviewing source documents that have been uploaded into WebDCU™ or via remote access to electronic medical records), and **central monitoring** (using web-based data validation rules, data manager review of entered data, statistical analysis, and on-going review of site metrics).
- The NDMC, study co-PIs, and the appropriate site PIs will be provided copies of monitoring reports within 30 days of site visits.

Further details of clinical site monitoring are documented in the study's Monitoring Plan. The Monitoring Plan describes in detail who will conduct the monitoring, at what frequency monitoring will be done, at what level of detail monitoring will be performed, and the distribution of monitoring reports.

10 STATISTICAL CONSIDERATIONS

10.1 STATISTICAL AND ANALYTICAL PLAN

Statistical analysis details are documented in a formal statistical and analytical plan (SAP). The SAP will be finalized prior to the first site being released to enroll subjects.

10.2 STATISTICAL HYPOTHESES

Primary efficacy endpoint: The null hypothesis (H_0) is that the hazard ratio comparing apixaban versus aspirin for the primary endpoint will be 1.

Secondary efficacy endpoint: The null hypothesis (H_0) is that the hazard ratio comparing apixaban versus aspirin for the secondary endpoint will be 1.

10.3 ANALYSIS DATASETS

The analysis datasets will include:

- Intention-to-treat analysis dataset, which will include all randomized participants.
- Safety analysis dataset, which will include all participants who took at least one dose of study drug.
- Per-protocol analysis dataset, which will include all randomized participants who do not have an eligibility violation and who received at least one dose of study drug.

10.4 DESCRIPTION OF STATISTICAL METHODS

10.4.1 GENERAL APPROACH

Efficacy outcomes will be analyzed in the intention-to-treat analysis dataset. Safety outcomes will be analyzed in the safety analysis dataset.

10.4.2 ANALYSIS OF THE PRIMARY AND SECONDARY ENDPOINTS

The log-rank statistic will be used to test the null hypothesis that the hazard ratio is 1, comparing apixaban to aspirin, at a two-tailed alpha level of 0.05.

10.4.3 SAFETY ANALYSES

A standard frequency table will be used to report the number (%) of SAEs, stratified by treatment group. Based on prior reports,[32,38] the following approximate annual rates of major safety endpoints are expected:

**Adverse Event**	**Apixaban**	**Aspirin**
Symptomatic intracranial hemorrhage	1.5%	1.5%
Major hemorrhage other than intracranial hemorrhage	4.0%	3.0%
All-cause mortality	3.5%	3.5%

Log-rank tests will be utilized to assess the treatment group differences in the rates of: (1) first symptomatic intracranial hemorrhage; (2) first major hemorrhage other than intracranial hemorrhage; and (3) death from any cause.

10.4.4 PLANNED INTERIM ANALYSES

The ARCADIA investigators propose one interim analysis for efficacy (or harm) and futility to be performed when 1/2 of the expected events have occurred (75 events), with the details specified in the SAP.

10.4.5 ADDITIONAL SUBGROUP ANALYSES

The primary efficacy and safety analyses will be stratified by age (<75 years versus ≥75 years), sex/gender, and race/ethnicity (non-Hispanic white versus others). Differential effects of apixaban versus aspirin across these subgroups are not anticipated and therefore the trial is not powered specifically for these subgroup analyses, but these analyses will allow any unexpectedly large variations to be identified.

10.4.6 EXPLORATORY AND SENSITIVITY ANALYSES

The SAP lists additional prespecified exploratory and sensitivity analyses, including analyses related to the secondary objective of the trial, which is to test the hypothesis that the relative efficacy of apixaban over aspirin increases with the severity of atrial cardiopathy.

10.5 SAMPLE SIZE

10.5.1 ASSUMPTIONS

The estimated sample size of 1,100 subjects is based on several important assumptions:

- 30-month uniform accrual period and 18-month minimum follow-up period;
- 7% annual risk of recurrent stroke of any type in aspirin-treated subjects;
- 40% relative reduction (hazard ratio, 0.6) in the stroke recurrence rate with apixaban compared to aspirin;
- 3% annual rate of cross-over from blinded aspirin to open-label apixaban because of AF detection;
- 3% annual rate of cross-over from blinded apixaban to open-label aspirin because of bleeding or other SAEs; and
- 5% annual rate of loss to follow-up or death in each group.

10.5.2 SAMPLE SIZE CALCULATION

Given the assumptions above, enrollment of 1,100 subjects (150 recurrent stroke events anticipated) will provide 80% power to demonstrate a statistically significant difference in the hazard for recurrent stroke with apixaban compared to aspirin at a two-sided alpha level of 0.05 while allowing for an interim look for efficacy (or harm) and futility. An O'Brien-Fleming type Lan-DeMets error spending function[39] will be used to perform one interim analysis at the halfway point of the trial. The sample size estimate assumes that the futility boundary will be nonbinding.

10.6 MEASURES TO MINIMIZE BIAS

10.6.1 ENROLLMENT, RANDOMIZATION, AND MASKING PROCEDURES

Eligible patients will be allocated in a 1:1 ratio to apixaban or aspirin using a scheme that balances randomization by regional coordinating center.

To maintain blinding, the BMS-Pfizer Partnership will provide the NCC Central Pharmacy with apixaban and aspirin tablets along with an equal number of matching placebo tablets. Site pharmacists and all investigators and subjects will be blinded throughout the course of the study. Switching to open-label anticoagulation, if indicated, should not result in unblinding of previous treatment status up until that point. Subjects who switch to open-label anticoagulation because of AF or other indication, or who switch to open-label antiplatelet therapy, will continue to be followed and will be analyzed according to the intention-to-treat principle with blinded outcome adjudication. Unblinding should only occur if a compelling clinical reason arises, such as active major bleeding or a pending decision on whether to administer intravenous thrombolysis for acute ischemic stroke, in which case the site investigator can perform emergency unblinding via procedures detailed in the MOP. In the case of unblinding, adjudication of any study endpoints that led to the unblinding will be performed centrally by adjudicators who remain blinded to the subject's treatment status. Once a participant's treatment is unblinded, that participant will no longer receive study drug.

10.6.2 BREAKING THE STUDY BLIND

This is a blinded study. All co-PIs, site study team members including site pharmacists, and study subjects will be fully blinded to treatment assignment. Unblinding is unlikely to be needed, but in the event that emergency unblinding is requested, unblinding procedures are specified in the MOP. The site investigator should only request unblinding when it is essential for the subject's safety (e.g., when considering administration of intravenous thrombolysis for recurrent acute ischemic stroke, managing life-threatening bleeding, or undertaking emergency surgery).

Participants will be provided with identification that includes study contact information and indicates that they are participating in a study of aspirin versus apixaban for recurrent stroke. This will be useful in the event of a medical emergency. A notification will also be sent to participants' primary care physicians to inform them of their subject's participation in this trial.

11 SOURCE DOCUMENTS AND ACCESS TO SOURCE DATA

Each participating site will maintain appropriate medical and research records for this trial, in compliance with ICH E6 and regulatory and institutional requirements for the protection of confidentiality of participants. As part of participating in an NINDS-sponsored trial, each site will permit authorized representatives of NINDS and regulatory agencies to examine (and when permitted by applicable law, to copy) clinical records for the purposes of quality assurance reviews, audits, and evaluation of the study safety, progress, and data validity.

Data will be collected using CRFs whenever possible, but source data to be collected will also include copies of provider notes, laboratory results, and imaging reports.

Subjects' participation in the study will be documented in the electronic medical record unless prohibited by local regulations.

In an effort to review informed consent forms in a timely manner, enrolling sites will upload a PDF of the signed informed consent form into the password protected clinical trial management system, WebDCU™. The PDF file will be linked to the Subject ID but will be stored on a secure server separate from the study's CRF data. The secure server on which these files are stored is not backed up to prevent copies of files containing individually identifiable health information from being copied and stored on non-NDMC back up servers. The files on these servers can only be accessed by designated NDMC study personnel. NDMC staff will remotely monitor the informed consent forms and issues identified will be relayed to the clinical site for corrective and preventative action. After remote monitoring is complete, the PDF file containing the informed consent form will be permanently deleted from the secure server. If a subject must be re-consented, the process will repeat itself.

12 QUALITY ASSURANCE AND QUALITY CONTROL

Quality control (QC) procedures will be implemented beginning with the data entry system, and data QC checks that will be run on the database will be generated. Any missing data or data anomalies will be communicated to the site(s) for clarification/resolution.

Following written procedures as detailed in the monitoring plan, the monitors will verify that the clinical trial is conducted and data are generated, documented (recorded), and reported in compliance with the protocol, GCP, and the applicable regulatory requirements (e.g., Good Laboratory Practices (GLP), Good Manufacturing Practices (GMP)).

The investigational site will provide direct access to all trial-related sites, source data/documents, and reports for the purpose of monitoring and auditing by the sponsor, and inspection by local and regulatory authorities.

13 ETHICS/PROTECTION OF HUMAN SUBJECTS

13.1 ETHICAL STANDARD

The co-PIs will ensure that this study is conducted in full conformity with Regulations for the Protection of Human Subjects of Research codified in 45 CFR Part 46, 21 CFR Part 50, 21 CFR Part 56, and/or the ICH E6.

13.2 INSTITUTIONAL REVIEW BOARD

The protocol, informed consent form, recruitment materials, and all participant materials will be submitted to applicable central and local IRB/REBs for review and approval. Approval of both the protocol and the consent form will be obtained before any participant is enrolled. Any amendment to the protocol will require review and approval by the cIRBs/IRB/REBs before the changes are implemented to the study. All changes to the template consent form will be cIRB/IRB/REBs approved; a determination will be made regarding whether previously consented participants need to be re-consented.

13.3 INFORMED CONSENT PROCESS

13.3.1 CONSENT AND OTHER INFORMATION DOCUMENTS PROVIDED TO PARTICIPANTS

Consent forms describing in detail the study agent, study procedures, and risks will be given to the participant and written documentation of informed consent will be performed prior to starting intervention/administering study product. The following consent materials are submitted with this protocol: informed consent form with included HIPAA authorization form.

13.3.2 CONSENT PROCEDURES AND DOCUMENTATION

In accordance with FDA regulations (21 CFR 50) and ICH-GCP Consolidated Guidelines, cIRB/IRB/REB-approved, informed consent is required from all patients prior to participating in this study. At the initial contact with a potential candidate, the investigative team (investigator or coordinator) will provide a comprehensive explanation of the purpose, procedures, possible risks/benefits of the study in language that is understandable to a non-medically trained person; as well as participant responsibilities and the fact that his/her participation is voluntary, that he or she may withdraw from the study at any time, and that the decision not to participate or to withdraw will not affect the patient's care in any way. Potential participants will be given ample opportunity to ask questions and to consider their decision. If the patient expresses a sustained interest, a signed and dated written informed consent will be obtained. Patients with a known history of dementia will not provide self-consent, thereby minimizing the possibility of invalid informed consent. A copy of the consent form will be given to the participant, and another copy placed in his or her medical record unless prohibited by local regulations. The informed consent will be obtained by either the clinical site PI or other members of the study team who are qualified to perform this task and whose names are listed on the Delegation of Authority Log. Informed consent will be performed in a language in which the patient or surrogate (see below) is fluent. Participants will be asked to explain back the study to confirm their understanding of the study and its procedures.

13.3.3 USE OF SURROGATES FOR CONSENT

Since this is a long-term secondary stroke prevention trial, the goal is to enroll subjects who will be able to participate actively in their care over the duration of the trial. However, recognizing that otherwise suitable subjects will often have neurological deficits that may impair their ability to provide written, informed consent, especially soon after their index stroke, the trial will allow inclusion of subjects via the use of surrogate consent. Loss of capacity to provide consent is a common, though hardly universal, feature in stroke patients. Neurologists and other physicians who care for stroke patients, as well as coordinators involved in stroke trials, are familiar with the signs and symptoms of stroke that can render a stroke patient unable to provide informed consent. All investigators and coordinators will be trained in the process of enrolling stroke patients using informed consent.

The use of surrogates for consent will conform with determinations required by federal regulations for the cognitively impaired:

- 45 CFR 46.111(a): Selection of subjects is equitable. Use of surrogate consent leads to inclusion of a more representative cohort of subjects with stroke and is therefore more equitable than

excluding subjects who are otherwise eligible except for their inability to provide consent themselves.

- 45 CFR 46.111(b): Additional safeguards have been included to protect their rights and welfare. Although the active treatment arm of this trial imposes a risk of injury from increased bleeding, compelling preliminary data exists to suggest that subjects receiving apixaban will benefit from improved stroke prevention and that the probability of benefit is greater than the probability of harm. Subjects assigned to aspirin will receive the current standard of care for stroke prevention.
- 45 CFR 46.116: Informed consent must be obtained from the subject or the subject's legally authorized representative.

Our protocol will be the following: First, the lack of capacity will be determined. Acute stroke patients frequently lack capacity due to language deficits, inattention to their health (neglect and anosognosia, or lack of awareness of their deficit), or other deficits. A trained study investigator will make these initial assessments and this process of determination of lack of capacity will be documented in the medical record.

Second, once the patient is determined to lack capacity, a member of the investigative team will seek to identify a court-appointed legally authorized representative/guardian or appropriately executed health care proxy. This individual will serve as the legally authorized representative to provide surrogate consent.

Third, if no court-appointed legally authorized representative/guardian or appropriately executed health care proxy is available, then a member of the investigative team will obtain surrogate consent from another person on the following list: (a) the spouse (if not legally separated from the subject) or the domestic partner; (b) a son or daughter eighteen (18) years of age or older; (c) a parent; (d) a brother or sister eighteen (18) years of age or older; (e) a close friend (meaning a person eighteen [18] years of age or older who has maintained such regular contact with the subject as to be familiar with the subject's activities, health, and beliefs). Determination of the appropriate individual to provide informed consent will be determined through means considered possible and appropriate by the study investigator or coordinator, including but not limited to presentation and review of documents, discussion with family members present, review of administrative and medical records, and discussion with the patient's primary care physician, who is likely to know these individuals. The discussion will include the patient when possible, even if the patient is unable to provide formal consent due to language or other deficits. In this circumstance, the patient's assent will be sought, when possible. In addition, the discussion will include multiple family members as appropriate in the investigator's judgment, and agreement among family members will be sought.

Finally, if a subject previously determined to lack capacity to consent regains capacity during the study, the investigator will obtain the consent of the individual for the remaining part of the study.

Detailed procedures for determining subjects' capacity for consent and obtaining consent from a surrogate, if necessary, are specified in the MOP. Investigators and coordinators will be trained in the use of surrogates for informed consent.

13.4 PARTICIPANT AND DATA CONFIDENTIALITY

13.4.1 CONFIDENTIALITY AND DATA STORAGE

Participant confidentiality is strictly held in trust by the participating investigators, their staff, and the sponsor(s) and their agents. This confidentiality is extended to cover testing of biological samples in addition to clinical information relating to participants. Therefore, the study protocol, documentation, data, and other information generated will be held in strict confidence. No information about the study or data will be released to any unauthorized third party without prior written approval of the sponsor.

The study monitor, other authorized representatives of the sponsor, and representatives of the NDMC, applicable cIRB/IRB/REBs, and pharmaceutical company supplying study product may inspect all documents and records required to be maintained by the investigator, including but not limited to, medical records and pharmacy records for the participants in this study. The clinical study site will permit access to such records.

The study participant's contact information will be securely stored at each clinical site for internal use during the study. At the end of the study, all records will continue to be kept in a secure location for the duration specified by the StrokeNet Standard Operating Procedure (SOP) or longer as dictated by applicable cIRB/IRB/REBs and local institutional regulations.

Study participant research data, which is for purposes of statistical analysis and scientific reporting, will be transmitted to and stored for the duration of the study and analysis at the NDMC. The study data entry and study management systems used by clinical sites and by the NDMC research staff will be secured and password protected. At the end of the study, all study databases will be de-identified and a Public Use Dataset (PUDS) will be archived at the NINDS data repository.

13.4.2 RESEARCH USE OF STORED HUMAN SAMPLES

Intended Use: Samples collected from participants at the time of screening will be stored and will be used to assay NT-proBNP to determine eligibility for the study. Samples may also be used for other studies of stroke and cardiac disease (see Section 13.5 below). No genetic testing will be performed without further amendment of this protocol and informed consent form.

Storage: The biobank repository will be kept at the Laboratory Core for the study, in the Center for Advanced Laboratory Medicine (CALM) at Columbia University Medical Center (CUMC), under the supervision of Dr. Marshall, Contact PI for the study, and Dr. Eldad Hod, a clinical pathologist and Director of CALM. A dedicated -80° C freezer will be used for this study. Drs. Marshall and Hod, and their delegates, will have responsibility for the integrity of the repository. Access to the repository for future research studies will be limited to those approved by the ARCADIA Executive Committee.

Tracking: Samples and data will be stored using codes. Data will be kept in password-protected computers. Only investigators will have access to the samples and data. Study participants who request destruction of samples will be notified of compliance with such request and all supporting details will be maintained for tracking.

13.5 FUTURE USE OF STORED SPECIMENS

All ARCADIA ancillary studies, including those that include the use of blood specimens, will be reviewed by the Publications and Executive Committees for approval. Specimens will be used only for purposes related to research described in this protocol, i.e., understanding the causes and optimal prevention, treatment, and outcomes of unexplained strokes. The purpose of the Executive and Publications Committee reviews will be to assure that future uses of subject specimens are consistent with the informed consent obtained at the time the specimen was collected. A letter of approval for use of biobank specimens will be provided to the ancillary study PI. Once approved and, if necessary, funded, Dr. Hod's delegated staff will retrieve the samples for assays to be performed locally or for shipping to offsite laboratories as requested and appropriate for the particular study. Investigators who receive specimens from the study will sign forms to indicate that the samples are solely for their use and they will be expressly forbidden from transferring samples to any third parties. The CALM biosample repository will also be provided with a code-link that will allow linking the biological specimens with the phenotypic data from each participant, maintaining the masking of the identity of the participant.

Informed consent for collection and storage of blood samples for the biobank (i.e. blood samples above and beyond that needed to determine NT-pro BNP for study eligibility) is incorporated into the primary ARCADIA consent form, but as a separate section requiring separate approval. Participants will thus have the opportunity to participate in ARCADIA without participating in the biobank. No additional consent will be obtained so as to minimize inconvenience to the participant and the local investigator's staff by collecting the blood samples at a single time. Because collection of a sample for the assay for NT-pro BNP is being performed to determine eligibility for randomization, the collection of limited additional samples for the purpose of this biobank is expected to cause minimal risk and inconvenience, thereby limiting the possibility for interference with the parent ARCADIA study. Participants can withdraw consent for research on stored specimens at any time and the specimens will be discarded; this information is also included in the consent form. However, data already collected about the participants will be retained and analyzed even if the subjects choose to withdraw from the research. If a subject revokes authorization in writing for continued use or disclosure of blood sample data or protected health information (PHI) that was already obtained in the research, analysis of that data will continue only to the extent necessary to protect the integrity of the research study. Furthermore, withdrawal of consent with regard to biosample storage will not be possible after the study is completed.

The privacy and confidentiality of the biobank will be ensured through the use of standard CALM procedures. All individuals who handle specimens will be trained in accordance with GCP and on specific modules related to the handling of biospecimens. This training will include safeguards to prevent accidental or inappropriate release of information. A list of those with access to the samples will be kept with Dr. Hod as Director of the CALM Laboratory. The samples themselves will be kept in a dedicated freezer in a locked room in the CALM Laboratory; access will be limited to Drs. Hod, Marshall, and their delegates. Any clinical data collected will be handled through WebDCU™. Samples will be shipped to CALM to arrive during business hours. Samples will be accompanied by labels on tubes and a sample shipping form that will include no patient identifiers other than the study ID number. The samples will be stored at CALM using this study ID number only, and samples will be bar coded for ease of storage and retrieval. Any laboratory results will be provided to WebDCU™. Further use of this information will be according to the study-wide procedures for maintenance of confidentiality and privacy. Depending on funding, analyses for ancillary studies may be performed by the NDMC, or, if they are performed locally by an ancillary study investigator, a limited data set, including key clinical variables needed for

the analyses, may be provided by the NDMC to enable the analyses to be performed. PHI will not be provided without further authorization by the Executive Committee. A Certificate of Confidentiality will be obtained prior to the beginning of enrollment.

Specimens will be destroyed 10 years after the publication of the primary manuscript describing the results of the ARCADIA study. Samples will be discarded in red medical waste bags specially made to contain medical or biohazardous waste and certification will be provided to the co-PIs and other requestors.

In the event that an ancillary study proposal is approved that uses the specimens and does not involve any investigators related to the main protocol, a sample use agreement will be required. In this case, to minimize the possibility that data in the biobank could be used to identify individuals in the study, we will add to the agreement an additional statement stating that the recipient may not attempt to identify subjects by any means.

Specimens may be made available to commercial organizations. Commercial organizations will need to complete an ancillary study proposal form, which will be reviewed by the Executive and Publications Committees, which includes NINDS representation. Final agreements with commercial organizations will need to be reviewed with legal counsel of the study sponsor, as well, in the event of potential development of commercial products.

14 DATA HANDLING AND RECORD KEEPING

14.1 DATA COLLECTION AND MANAGEMENT RESPONSIBILITIES

Data collection is the responsibility of the clinical trial staff at the site under the supervision of the site PI. The investigator is responsible for ensuring the accuracy, completeness, legibility, and timeliness of the data reported.

All source documents should be completed in a neat, legible manner to ensure accurate interpretation of data. Black ink is required to ensure clarity of reproduced copies. When making changes or corrections, cross out the original entry with a single line, and initial and date the change. DO NOT ERASE, OVERWRITE, OR USE CORRECTION FLUID OR TAPE ON THE ORIGINAL.

Copies of the electronic CRF (eCRF) will be provided for use as source documents and maintained for recording data for each participant enrolled in the study. Data reported in the eCRF derived from source documents should be consistent with the source documents or the discrepancies should be explained and captured in a progress note and maintained in the participant’s official electronic study record.

Clinical data will be entered into WebDCU™. The data system includes password protection and internal quality checks, such as automatic range checks, to identify data that appear inconsistent, incomplete, or inaccurate. Clinical data will be entered directly from the source documents.

14.2 STUDY RECORDS RETENTION

Study documents should be retained for the duration specified by the StrokeNet SOP or for a longer period if required by local regulations.

14.3 PROTOCOL DEVIATIONS

A protocol deviation is any noncompliance with the clinical trial protocol, GCP, or MOP requirements. The noncompliance may be either on the part of the participant, the investigator, or the study site staff. As a result of deviations, corrective action plans are to be developed by the site after review and approval by the NCC.

These practices are consistent with ICH E6:

- 4.5 Compliance with Protocol, sections 4.5.1, 4.5.2, and 4.5.3.
- 5.1 Quality Assurance and Quality Control, section 5.1.1.
- 5.20 Noncompliance, sections 5.20.1, and 5.20.2.

It is the responsibility of the site to use continuous vigilance to identify and report deviations within 10 working days of identification of a protocol deviation meeting criteria for prompt reporting, or within 10 working days of the scheduled protocol-required activity. These protocol deviations must be addressed in study source documents and reported via WebDCU™ for review by the ARCADIA Project Manager. All other protocol deviations must be reported to the applicable cIRB/IRB/REBs at the time of annual reviews. The site PI and study staff are responsible for knowing and adhering to applicable cIRB/IRB/REB requirements. Further details about the handling of protocol deviations are included in the MOP.

14.4 PUBLICATION AND DATA SHARING POLICY

This study will comply with the NIH Public Access Policy, which ensures that the public has access to the published results of NIH funded research. It requires scientists to submit final peer-reviewed journal manuscripts that arise from NIH funds to the digital archive PubMed Central upon acceptance for publication. As required by International Committee of Medical Journal Editors (ICMJE) member journals and Section 801 of the Food and Drug Administration Amendments Act (FDAAA) of 2007, this trial will be registered prior to its initiation at clinicaltrials.gov, a public trials registry sponsored by the National Library of Medicine. The co-PIs will take specific steps to ensure compliance with NIH implementation of FDAAA.

Data generated through this project will be shared according to StrokeNet SOP ADM 04 regarding the Network Data Sharing Policy Data Sharing Policy. In summary, the goals of this Data and Resource Sharing Policy are to make available final data from StrokeNet clinical trials to the research community, while safeguarding the privacy of trial participants and protecting confidential and proprietary data.

Upon database lock, the NDMC statisticians will generate data files from each data table corresponding to each electronic CRF in the database. In compliance with the HIPAA regulations, each data table will be stripped of any and all personal identifiers and will undergo a deidentification process. Furthermore, the NDMC statisticians will create a minimum number of derived variables that would be necessary to ensure reproducibility of the primary analysis.

Within 1 year from the acceptance of the primary manuscript for publication OR no later than 2 years from the database lock the PUDS will be submitted to the NINDS data repository, along with the final version of the study protocol, the data dictionary, and a user guide (or a “Readme” file) regarding the data files, including an explanation of any derived variables. Once PUDS are available, any researcher (study investigator or otherwise) wishing to receive them can contact the NINDS (CRLiaison@ninds.nih.gov). All manuscripts, abstracts, and press releases using the study data must acknowledge the StrokeNet investigators and the NINDS as the study sponsor with the relevant grant numbers.

In order to expedite and track external data sharing, the ARCADIA website will serve as a resource and informational site for potential external investigators and collaborators. The website will include the ARCADIA publication policy, the data sharing procedures and policies, timelines, and contact information. All published ARCADIA manuscripts, abstracts, and brief descriptions of ongoing projects will be posted on the website, maintained by the StrokeNet NCC.

15 STUDY ADMINISTRATION

15.1 OVERVIEW

This trial is being conducted as part of the NIH-funded StrokeNet network for stroke clinical trials. Trial administration will be handled by the StrokeNet NCC at the University of Cincinnati, and data management and monitoring by the NDMC at the Medical University of South Carolina. The ARCADIA co-PIs will have overall responsibility for study design, site selection, analysis and interpretation of final data, and dissemination of study results.

15.2 STUDY MANAGEMENT CORES (NCC and CCC)

For participating U.S. sites, the NCC will perform study management tasks such as site management and contracting, cIRB oversight, trial-wide communications, orchestration of training activities, coordination of site initiation visits, collection of regulatory documents, and site performance analyses. This core will also receive study drug from BMS-Pfizer, label drugs to maintain blinding, and distribute study drug to sites on a regular schedule.

For participating Canadian sites, the NCC will perform study management tasks such as trial-wide communications, review and approval of regulatory documents, and protocol support. The NCC will also receive study drug from BMS-Pfizer, label drugs to maintain blinding, and distribute study drug to the Canadian depot. The Canadian Coordinating Centre (CCC) will perform study management tasks such as site management and contracting, collection of regulatory documents, cIRB/IRB/REB oversight, orchestration of training activities, coordination of site initiation visits, and site performance analyses. The CCC will oversee distribution of study drug from the central Canadian depot to sites on a regular schedule.

15.3 DATA MANAGEMENT CORE (NDMC)

The study database will reside at Medical University of South Carolina according to the procedures governing the StrokeNet. This core consists of those responsible for maintenance of the database, logic

checks, edit requests, quality assurance, merging of datasets, creating and exporting data subsets, reliability and validity substudies, programming reports and data arrays needed for tracking enrollment and follow-up, and supporting other operational, project management, and analysis needs. This core will interface closely with the ARCADIA co-PIs, co-investigators, and all the committees. This core will provide unblinded reports to the DSMC for their meetings, and will provide a final locked database to the blinded study statistician after data collection and cleaning have been completed.

15.4 EXECUTIVE COMMITTEE

The Executive Committee will consist of the following voting members: the four ARCADIA co-PIs, the blinded study statistician, the study cardiologist, the PIs of the NCC and NDMC, the NINDS project scientist, the Canadian Sites Lead PI, and the CCC Lead PI. Key blinded members of the Trial Operations Committee will also attend. The Executive Committee in its entirety will meet twice annually, and more frequently as needed, to review and if necessary vote on key study issues, including but not limited to: 1) approving modification of the protocol or operations manual, 2) reviewing study progress, 3) mediating budgetary disputes, 4) reviewing and approving issues surrounding local projects and ancillary studies, and 5) reviewing and approving all abstracts and publications.

15.5 TRIAL OPERATIONS COMMITTEE

The Trial Operations Committee will include the four ARCADIA co-PIs, the PIs of the NCC and NDMC, the NINDS project scientist, the unblinded study statistician, and key members of the NCC, NDMC, CCC, and study Cores involved in study operations. Membership will also include individuals representing additional networks of study investigators that may join this trial. This committee will meet weekly to review recruitment and discuss execution, budget, and other day-to-day issues arising in the conduct of the trial.

15.6 PUBLICATIONS COMMITTEE

Membership of the Publications Committee will be determined by the Executive Committee. The Publications Committee will convene quarterly, and more frequently as needed (i.e., during the dissemination phase after trial completion), to review and make recommendations to the Executive Committee with regard to: 1) paper proposals, 2) prioritization of analysis requests, 3) writing group membership and authorship issues, 4) other manuscript-related issues. This Committee will function in accordance with the StrokeNet SOP regarding publications policies.

15.7 ANCILLARY STUDIES COMMITTEE

Membership of the Ancillary Studies Committee will be determined by the Executive Committee. The Ancillary Studies Committee is responsible for soliciting and reviewing ancillary study proposals and interfacing with all approved ancillary study teams to ensure their coordination with the parent trial. As needed, the Ancillary Study Committee will interface with the study cores to ensure equitable distribution of samples and data, and with the Publications Committee to review proposed analyses and manuscripts. The Ancillary Study Committee will function in accordance with the StrokeNet SOP regarding ancillary studies.

16 CONFLICT OF INTEREST POLICY

The independence of this study from any actual or perceived external influence is critical. Therefore any actual conflict of interest of persons who have a role in the design, conduct, analysis, publication, or any aspect of this trial will be disclosed and managed. Furthermore, persons who have a perceived conflict of interest will be required to have such conflicts managed in a way that is appropriate to their participation in the trial. The study leadership in conjunction with the NINDS has established policies and procedures for all study group members to disclose all conflicts of interest and will establish a mechanism for the management of all reported dualities of interest.

17 LITERATURE REFERENCES

1. Marnane M, Duggan CA, Sheehan OC, et al. Stroke subtype classification to mechanism-specific and undetermined categories by TOAST, A-S-C-O, and Causative Classification system. *Stroke.* 2010;41(8):1579-1586.
2. Hart RG, Diener HC, Coutts SB, et al. Embolic strokes of undetermined source: the case for a new clinical construct. *Lancet Neurol.* 2014;13(4):429-438.
3. Healey JS, Connolly SJ, Gold MR, et al. Subclinical atrial fibrillation and the risk of stroke. *N Engl J Med.* 2012;366(2):120-129.
4. Brambatti M, Connolly SJ, Gold MR, et al. Temporal relationship between subclinical atrial fibrillation and embolic events. *Circulation.* 2014;129(21):2094-2099.
5. Martin DT, Bersohn MM, Waldo AL, et al. Randomized trial of atrial arrhythmia monitoring to guide anticoagulation in patients with implanted defibrillator and cardiac resynchronization devices. *Eur Heart J.* 2015;36(26):1660-1668.
6. Kamel H, Elkind MS, Bhave PD, et al. Paroxysmal supraventricular tachycardia and the risk of ischemic stroke. *Stroke.* 2013;44(6):1550-1554.
7. Hancock EW, Deal BJ, Mirvis DM, et al. AHA/ACCF/HRS recommendations for the standardization and interpretation of the electrocardiogram: part V: electrocardiogram changes associated with cardiac chamber hypertrophy: a scientific statement from the American Heart Association Electrocardiography and Arrhythmias Committee, Council on Clinical Cardiology; the American College of Cardiology Foundation; and the Heart Rhythm Society: endorsed by the International Society for Computerized Electrocardiology. *Circulation.* 2009;119(10):e251-261.
8. Kamel H, Soliman EZ, Heckbert SR, et al. P-wave morphology and the risk of incident ischemic stroke in the Multi-Ethnic Study of Atherosclerosis. *Stroke.* 2014;45(9):2786-2788.
9. Kamel H, O'Neal WT, Okin PM, Loehr LR, Alonso A, Soliman EZ. Electrocardiographic left atrial abnormality and stroke subtype in the Atherosclerosis Risk In Communities study. *Ann Neurol.* 2015;78(5):670-678.
10. Kamel H, Bartz TM, Longstreth WT, Jr., et al. Association between left atrial abnormality on ECG and vascular brain injury on MRI in the Cardiovascular Health Study. *Stroke.* 2015;46(3):711-716.
11. Kamel H, Hunter M, Moon YP, et al. Electrocardiographic left atrial abnormality and risk of stroke: Northern Manhattan Study. *Stroke.* 2015;46(11):3208-3212.
12. Benjamin EJ, D'Agostino RB, Belanger AJ, Wolf PA, Levy D. Left atrial size and the risk of stroke and death. The Framingham Heart Study. *Circulation.* 1995;92(4):835-841.
13. Barnes ME, Miyasaka Y, Seward JB, et al. Left atrial volume in the prediction of first ischemic stroke in an elderly cohort without atrial fibrillation. *Mayo Clin Proc.* 2004;79(8):1008-1014.

14. Di Tullio MR, Sacco RL, Sciacca RR, Homma S. Left atrial size and the risk of ischemic stroke in an ethnically mixed population. *Stroke.* 1999;30(10):2019-2024.
15. Yaghi S, Moon YP, Mora-McLaughlin C, et al. Left atrial enlargement and stroke recurrence: the northern Manhattan stroke study. *Stroke.* 2015;46(6):1488-1493.
16. Folsom AR, Nambi V, Bell EJ, et al. Troponin T, N-terminal pro-B-type natriuretic peptide, and incidence of stroke: the Atherosclerosis Risk In Communities study. *Stroke.* 2013;44(4):961-967.
17. Cushman M, Judd SE, Howard VJ, et al. N-terminal pro-B-type natriuretic peptide and stroke risk: the Reasons for Geographic and Racial Differences in Stroke Cohort. *Stroke.* 2014;45(6):1646-1650.
18. Kamel H, Okin PM, Elkind MS, Iadecola C. Atrial fibrillation and mechanisms of stroke: time for a new model. *Stroke.* 2016;47(3):895-900.
19. Sanna T, Diener HC, Passman RS, et al. Cryptogenic stroke and underlying atrial fibrillation. *N Engl J Med.* 2014;370(26):2478-2486.
20. Longstreth WT, Jr., Kronmal RA, Thompson JL, et al. Amino terminal pro-B-type natriuretic peptide, secondary stroke prevention, and choice of antithrombotic therapy. *Stroke.* 2013;44(3):714-719.
21. Connolly SJ, Eikelboom J, Joyner C, et al. Apixaban in patients with atrial fibrillation. *N Engl J Med.* 2011;364(9):806-817.
22. Ruff CT, Giugliano RP, Braunwald E, et al. Comparison of the efficacy and safety of new oral anticoagulants with warfarin in patients with atrial fibrillation: a meta-analysis of randomised trials. *Lancet.* 2014;383(9921):955-962.
23. Granger CB, Alexander JH, McMurray JJ, et al. Apixaban versus warfarin in patients with atrial fibrillation. *N Engl J Med.* 2011;365(11):981-992.
24. Kernan WN, Ovbiagele B, Black HR, et al. Guidelines for the prevention of stroke in patients with stroke and transient ischemic attack: a guideline for healthcare professionals from the American Heart Association/American Stroke Association. *Stroke.* 2014;45(7):2160-2236.
25. Dennis M. Embolic stroke of undetermined source: a therapeutic target? *Lancet Neurol.* 2014;13(4):344-346.
26. Freilinger TM, Schindler A, Schmidt C, et al. Prevalence of nonstenosing, complicated atherosclerotic plaques in cryptogenic stroke. *JACC Cardiovasc Imaging.* 2012;5(4):397-405.
27. Gupta A, Gialdini G, Lerario MP, et al. Magnetic resonance angiography detection of abnormal carotid artery plaque in patients with cryptogenic stroke. *J Am Heart Assoc.* 2015;4(6):e002012.
28. Gupta A, Gialdini G, Giambrone AE, et al. Association between nonstenosing carotid artery plaque on MR angiography and acute ischemic stroke. *JACC Cardiovasc Imaging.* 2016;9(10):1228-1229.
29. Chimowitz MI, Lynn MJ, Howlett-Smith H, et al. Comparison of warfarin and aspirin for symptomatic intracranial arterial stenosis. *N Engl J Med.* 2005;352(13):1305-1316.
30. Chimowitz MI, Lynn MJ, Derdeyn CP, et al. Stenting versus aggressive medical therapy for intracranial arterial stenosis. *N Engl J Med.* 2011;365(11):993-1003.
31. Amarenco P, Davis S, Jones EF, et al. Clopidogrel plus aspirin versus warfarin in patients with stroke and aortic arch plaques. *Stroke.* 2014;45(5):1248-1257.
32. Diener HC, Eikelboom J, Connolly SJ, et al. Apixaban versus aspirin in patients with atrial fibrillation and previous stroke or transient ischaemic attack: a predefined subgroup analysis from AVERROES, a randomised trial. *Lancet Neurol.* 2012;11(3):225-231.
33. Schulman S, Kearon C, Subcommittee on Control of Anticoagulation of the S, Standardization Committee of the International Society on T, Haemostasis. Definition of major bleeding in

clinical investigations of antihemostatic medicinal products in non-surgical patients. *J Thromb Haemost.* 2005;3(4):692-694.
34. Meschia JF, Brott TG, Chukwudelunzu FE, et al. Verifying the stroke-free phenotype by structured telephone interview. *Stroke.* 2000;31(5):1076-1080.
35. Katzan IL, Fan Y, Uchino K, Griffith SD. The PROMIS physical function scale: A promising scale for use in patients with ischemic stroke. *Neurology.* 2016;86(19):1801-1807.
36. Jauch EC, Saver JL, Adams HP, Jr., et al. Guidelines for the early management of patients with acute ischemic stroke: a guideline for healthcare professionals from the American Heart Association/American Stroke Association. *Stroke.* 2013;44(3):870-947.
37. Armstrong MJ, Gronseth G, Anderson DC, et al. Summary of evidence-based guideline: periprocedural management of antithrombotic medications in patients with ischemic cerebrovascular disease: report of the Guideline Development Subcommittee of the American Academy of Neurology. *Neurology.* 2013;80(22):2065-2069.
38. Nam HS, Kim HC, Kim YD, et al. Long-term mortality in patients with stroke of undetermined etiology. *Stroke.* 2012;43(11):2948-2956.
39. Lan KKG, DeMets DL. Discrete sequential boundaries for clinical trials. *Biometrika.* 1983;70(3):659-663.

CHANGE LOG

Version	Date	Significant Revisions
1.1	7 July 2017	• Added the composite outcome of stroke of any type or death from any cause as a secondary efficacy outcome (4.2.2).<br>• Clarified inclusion and exclusion criteria (5.1 and 5.2).<br>• Clarified that patients not eligible for randomization may still be followed as part of ancillary studies (7.2.2).<br>• Revised schedule of assessments (7.2.6).<br>• Clarified procedures for study drug administration after a potential primary endpoint (7.9).<br>• Only SAEs will be recorded (8.1.1).<br>• Clarified SAE reporting procedures from NDMC to BMS (8.4.2).<br>• Certificate of confidentiality will be obtained (13.5).<br>• Clarified reporting of protocol deviations (14.3).<br>• Corrected typographical errors and made minor revisions throughout to improve clarity.
2.0	22 Sep 2017	• Updated safety reporting language to distinguish between SAEs, clinical outcome events, and AEs of special interest, rather than lumping them together in one category.<br>• Added clarification about study medication refills (7.2.3).
3.0	01 Dec 2017	• Modified time of randomization based on stroke severity. In general, randomization can occur as early as post-stroke day 3. However, randomization must be delayed until at least post-stroke day 14 for patients with severe strokes (initial NIHSS score ≥11), hemorrhagic transformation of the index stroke, or uncontrolled hypertension (7.2.2).<br>• Added information about buffer or number of extra pills provided. (6.14)<br>• Added route of medication "by mouth" (6.16)<br>• Clarified what patient should do for a missed dose of study medication (i.e., when to take and when to skip). (6.16)<br>• Changed exclusion criteria to exclude patients with an indication for any antiplatelet therapy (5.2) and added requirement that study drug be stopped while any antiplatelet therapy is indicated after randomization (7.3.1). Clarified that all patients stopping study drug due to need for open-label anticoagulant or antiplatelet therapy will continued to be followed and analyzed according to the intention-to-treat principle (10.6.1).<br>• Follow-up visit window (7.2.3 and 7.2.6) window of time reduced from +/-14 days to +/-5 days to prevent subjects from running out of study medication and to keep them within the guidelines for follow-up visits based on time of randomization.
4.0	05 Feb 2019	• Changed number of sites from 120 to up to 200 (Protocol Summary).<br>• Updated study team members (pgs 11,12)

		• Clarified that mitral stenosis as an exclusion criterion applies only to moderate or severe mitral stenosis (5.1).<br>• Provided a procedure for obtaining written consent from surrogates via fax or other HIPAA-compliant method for transmitting written documents (5.3).<br>• Removed a general physical examination from study-specific procedures (7.1.1).<br>• Clarified that tests to establish ESUS must be performed no earlier than 3 months prior to the index stroke (7.1.2).<br>• Clarified requirements for contacting potential subjects by phone (7.2.1).<br>• Allowed follow-up visits via telephone or other HIPAA-compliant telehealth technology (7.2.3).<br>• Clarified that study drug must be stopped if a site identifies an event as a possible primary efficacy outcome (7.9).
4.1	24 Apr 2019	• Corrected an error in description of procedure for obtaining written consent from surrogates via fax or other HIPAA-compliant method for transmitting written documents (5.3).
4.1 (Canada only)	22 Aug 2019	• Updated personnel in Key Roles.<br>• Throughout: added U.S. and Canada to highlight that this is now a collaboration of sites in the two countries.<br>• List of Abbreviations: added REB (Regulatory Ethics Board)<br>• Throughout: revised cIRB to applicable cIRB and other IRB/REBs with study oversight to include other Canadian regulatory bodies.<br>• Section 8.4.2 Safety Reporting – we added reporting to Health Canada, cIRB and other IRB/REBs with study oversight according to local regulations.<br>• Section 8.4.3 -- reporting of unanticipated events to the cIRB and other IRB/REBs with study oversight and to Health Canada.<br>• Section 15.2 Study Management Cores – now includes Canadian Central Coordinating Center responsibilities
4.2 (Canada only)	12 Sep 2019	• Added an exclusion for eCrCl <15 mL/min (5.2).<br>• Specified that subjects with eCrCl 15-24 mL/min will receive apixaban 2.5 mg twice daily, regardless of other dose adjustment criteria (6.1.5, 6.1.7).<br>• Specified that laboratory determination of renal function must occur at least once per year as part of standard care (6.1.7).<br>• Added strong inhibitors and inducers of CYP3A4 and P-gp to the list of prohibited medications (7.3.3).
5.0	3 Apr 2020	• Incorporated all changes from Canadian protocol version 4.1 and 4.2 into combined U.S./Canada version 5.0.<br>• Updated study/participant duration in Protocol Summary and Schematic of Study Design.<br>• Streamlined and updated personnel list in Key Roles (Section 1).<br>• Changed inclusion criteria to require randomization no later than 180 days after stroke onset, rather than 120 days (5.1, 7.2.1, 7.2.2).

		• Specified that remote consent can be obtained from patients as well as their surrogates (5.3).<br>• Specified that eConsent can be used as allowed by local regulations and IRB/REB requirements (5.3).<br>• Specified procedures for reconsenting subjects (5.3).<br>• Updated time period of recruitment (5.5).<br>• Clarified procedure for handling subject withdrawal from study (5.6.1).<br>• First dose of study medication is now required within 48 hours of randomization, rather than 24 hours (6.1.5).<br>• Clarified that study-specific procedures do not need to be performed in person (7.1.1, 7.2.1).<br>• Clarified which echocardiogram should be used to determine the ECHO criterion for atrial cardiopathy (7.2.1).<br>• Specified that remote randomization is allowed (7.2.2).<br>• Added COVID-19 as an Adverse Event of Special Interest (8.1.3).<br>• Updated membership of Executive Committee and clarified key issues to be reviewed by Executive Committee (15.4).<br>• Updated membership of Trial Operations Committee (15.5).
5.1	12 Jan 2021	• Clarified nature of exploratory endpoints (4.2, 8.1).<br>• Clarified procedures for subjects who wish to withdraw from the study (5.6.1).<br>• Changed the time period for assessment of SAEs other than clinical outcome events as well as AEs of special interest. These must now be reported from randomization until 30 days after permanent study drug discontinuation. Clinical outcome events will still be ascertained from randomization until the end of study participation (7.1, 7.2, 8.1, 8.3, and 8.4).<br>• Clarified procedure for ascertaining presence of AF on ECG/heart-rhythm monitoring (7.1.2).<br>• Added procedure for collecting Medicare information (7.2).<br>• Clarify that last dose of open-label antiplatelet therapy allowed is on the day before randomization (7.3.1).
6.0	17 Aug 2022	• Updated Contact PI from Dr. Mitchell Elkind to Dr. Randolph Marshall.<br>• Updated eligibility criteria in light of recently updated ESUS criteria which exclude PFO-associated stroke (5.1).<br>• Minor clarification of follow-up and end-of-study visits (7.2.3., 7.2.4).<br>• Minor clarification of adjudication process after emergency unblinding (10.6.1).

ARCADIA

AtRial Cardiopathy and Antithrombotic Drugs In prevention After cryptogenic stroke

Protocol Identifying Number: NCT03192215

Principal Investigators:
Hooman Kamel, MD
Mitchell S.V. Elkind, MD, MS
W.T. Longstreth Jr, MD, MPH
David L. Tirschwell, MD, MSc

Funded by: NINDS
Supported by: BMS-Pfizer Partnership and Roche Diagnostics

Version Number: v3.0

1 Dec 2017

Table of Contents

LIST OF ABBREVIATIONS 5
STATEMENT OF COMPLIANCE 7
PROTOCOL SUMMARY 9
SCHEMATIC OF STUDY DESIGN 10
1 KEY ROLES 11
2 INTRODUCTION: BACKGROUND AND SCIENTIFIC RATIONALE 13
2.1 BACKGROUND 13
2.2 RATIONALE 14
2.3 POTENTIAL RISKS AND BENEFITS 14
2.3.1 KNOWN POTENTIAL RISKS 14
2.3.2 KNOWN POTENTIAL BENEFITS 15
3 OBJECTIVES AND PURPOSE 15
4 STUDY DESIGN AND ENDPOINTS 16
4.1 DESCRIPTION OF THE STUDY DESIGN 16
4.2 STUDY ENDPOINTS 16
4.2.1 PRIMARY ENDPOINTS 16
4.2.2 SECONDARY ENDPOINTS 16
4.2.3 EXPLORATORY ENDPOINTS 16
4.2.4 DEFINITIONS OF PRIMARY AND SECONDARY ENDPOINTS 16
4.2.5 ADJUDICATION OF ENDPOINTS 17
5 STUDY ENROLLMENT AND WITHDRAWAL 17
5.1 PARTICIPANT INCLUSION CRITERIA 17
5.2 PARTICIPANT EXCLUSION CRITERIA 18
5.3 INFORMED CONSENT FOR SCREENING AND RANDOMIZATION 19
5.4 ATRIAL CARDIOPATHY CRITERIA REQUIRED FOR RANDOMIZATION 19
5.5 STRATEGIES FOR RETENTION AND RECRUITMENT 19
5.5.1 TIME PERIOD FOR RECRUITMENT 19
5.5.2 INCLUSION OF WOMEN AND MINORITIES 19
5.6 PARTICIPANT WITHDRAWAL OR TERMINATION 20
5.6.1 REASONS FOR WITHDRAWAL OR TERMINATION 20
5.6.2 HANDLING OF PARTICIPANT WITHDRAWALS OR TERMINATION 21
5.7 PREMATURE TERMINATION OR SUSPENSION OF STUDY 21
6 STUDY AGENT 21
6.1 STUDY AGENT AND CONTROL DESCRIPTION 21
6.1.1 ACQUISITION 21
6.1.2 FORMULATION, APPEARANCE, PACKAGING, AND LABELING 21
6.1.3 PRODUCT STORAGE AND STABILITY 21
6.1.4 DISTRIBUTION 22
6.1.5 DOSING 22
6.1.6 ADMINISTRATION 22
6.1.7 LABORATORY MONITORING AND DOSE ADJUSTMENTS/INTERRUPTIONS 22
6.1.8 TRACKING OF DOSE 23

6.2 STUDY DRUG ACCOUNTABILITY PROCEDURES 23
7 STUDY PROCEDURES AND SCHEDULE 23
7.1 STUDY PROCEDURES/EVALUATIONS 23
7.1.1 STUDY-SPECIFIC PROCEDURES 23
7.1.2 STANDARD-OF-CARE STUDY PROCEDURES 24
7.2 STUDY SCHEDULE 25
7.2.1 SCREENING 25
7.2.2 RANDOMIZATION 26
7.2.3 FOLLOW-UP 27
7.2.4 FINAL STUDY VISIT 28
7.2.5 UNSCHEDULED VISIT 28
7.2.6 SCHEDULE OF EVENTS TABLE 29
7.3 CONCOMITANT AND PROHIBITED MEDICATIONS 30
7.3.1 ANTIPLATELET MEDICATIONS 30
7.3.2 ANTICOAGULANT MEDICATIONS 30
7.4 DETECTION AND MANAGEMENT OF ATRIAL FIBRILLATION 30
7.5 INTERRUPTION OF STUDY DRUG FOR ELECTIVE INVASIVE PROCEDURES 30
7.6 MANAGEMENT OF BLEEDING 31
7.7 MANAGEMENT OF ACUTE ISCHEMIC STROKE IN SUBJECTS ON STUDY DRUG 31
7.8 VASCULAR RISK FACTOR MANAGEMENT 32
7.9 PARTICIPANT ACCESS TO STUDY DRUG AFTER STUDY CLOSURE 32
8 ASSESSMENT OF SAFETY 32
8.1 SPECIFICATION OF SAFETY PARAMETERS 32
8.1.1 DEFINITION OF ADVERSE EVENTS 32
8.1.2 DEFINITION OF SERIOUS ADVERSE EVENTS 33
8.1.3 ADVERSE EVENTS OF SPECIAL INTEREST 34
8.1.4 UNANTICIPATED PROBLEMS AND UNANTICIPATED EVENTS 34
8.2 CLASSIFICATION OF AN ADVERSE EVENT 35
8.2.1 SEVERITY OF EVENT 35
8.2.2 RELATIONSHIP TO STUDY AGENT 35
8.2.3 EXPECTEDNESS 36
8.3 TIME PERIOD AND FREQUENCY FOR EVENT ASSESSMENT AND FOLLOW-UP 36
8.4 REPORTING PROCEDURES 37
8.4.1 ADVERSE EVENT REPORTING 37
8.4.2 SAFETY REPORTING 37
8.4.3 UNANTICIPATED EVENT REPORTING 38
8.5 STUDY OVERSIGHT 38
8.6 STUDY HALTING RULES 38
9 CLINICAL MONITORING 38
10 STATISTICAL CONSIDERATIONS 39
10.1 STATISTICAL AND ANALYTICAL PLAN 39
10.2 STATISTICAL HYPOTHESES 39
10.3 ANALYSIS DATASETS 39

10.4 DESCRIPTION OF STATISTICAL METHODS ... 40
10.4.1 GENERAL APPROACH ... 40
10.4.2 ANALYSIS OF THE PRIMARY AND SECONDARY ENDPOINTS ... 40
10.4.3 SAFETY ANALYSES ... 40
10.4.4 PLANNED INTERIM ANALYSES ... 40
10.4.5 ADDITIONAL SUBGROUP ANALYSES ... 40
10.4.6 EXPLORATORY AND SENSITIVITY ANALYSES ... 40
10.5 SAMPLE SIZE ... 41
10.5.1 ASSUMPTIONS ... 41
10.5.2 SAMPLE SIZE CALCULATION ... 41
10.6 MEASURES TO MINIMIZE BIAS ... 41
10.6.1 ENROLLMENT, RANDOMIZATION, AND MASKING PROCEDURES ... 41
10.6.2 BREAKING THE STUDY BLIND ... 42
11 SOURCE DOCUMENTS AND ACCESS TO SOURCE DATA ... 42
12 QUALITY ASSURANCE AND QUALITY CONTROL ... 42
13 ETHICS/PROTECTION OF HUMAN SUBJECTS ... 43
13.1 ETHICAL STANDARD ... 43
13.2 INSTITUTIONAL REVIEW BOARD ... 43
13.3 INFORMED CONSENT PROCESS ... 43
13.3.1 CONSENT AND OTHER INFORMATION DOCUMENTS PROVIDED TO PARTICIPANTS ... 43
13.3.2 CONSENT PROCEDURES AND DOCUMENTATION ... 43
13.3.3 USE OF SURROGATES FOR CONSENT ... 44
13.4 PARTICIPANT AND DATA CONFIDENTIALITY ... 45
13.4.1 CONFIDENTIALITY AND DATA STORAGE ... 45
13.4.2 RESEARCH USE OF STORED HUMAN SAMPLES ... 46
13.5 FUTURE USE OF STORED SPECIMENS ... 46
14 DATA HANDLING AND RECORD KEEPING ... 48
14.1 DATA COLLECTION AND MANAGEMENT RESPONSIBILITIES ... 48
14.2 STUDY RECORDS RETENTION ... 48
14.3 PROTOCOL DEVIATIONS ... 48
14.4 PUBLICATION AND DATA SHARING POLICY ... 49
15 STUDY ADMINISTRATION ... 49
15.1 OVERVIEW ... 49
15.2 STUDY MANAGEMENT CORE (NCC) ... 50
15.3 DATA MANAGEMENT CORE (NDMC) ... 50
15.4 EXECUTIVE COMMITTEE ... 50
15.5 TRIAL OPERATIONS COMMITTEE ... 50
15.6 PUBLICATIONS COMMITTEE ... 50
15.7 ANCILLARY STUDIES COMMITTEE ... 51
16 CONFLICT OF INTEREST POLICY ... 51
17 LITERATURE REFERENCES ... 51
CHANGE LOG ... 54

LIST OF ABBREVIATIONS

AE	Adverse event
AF	Atrial fibrillation/flutter
AHA/ASA	American Heart Association/American Stroke Association
ALT	Alanine aminotransferase
ARCADIA	AtRial Cardiopathy and Antithrombotic Drugs In prevention After cryptogenic stroke
AST	Aspartate aminotransferase
BMI	Body mass index
CALM	Center for Advanced Laboratory Medicine at Columbia University Medical Center
CFR	Code of Federal Regulations
cIRB	StrokeNet central institutional review board
CRF	Case report form
CT	Computed tomography
CTA	CT angiogram
CTCAE	Common Terminology Criteria for Adverse Events
CUMC	Columbia University Medical Center
DSMB	Data and safety monitoring board
ECG	Electrocardiogram
ESUS	Embolic stroke of undetermined source
FDA	Food and Drug Administration
FDAAA	FDA Amendments Act
GFR	Glomerular filtration rate
GI	Gastrointestinal
GCP	Good clinical practices
GLP	Good laboratory practices
GMP	Good manufacturing practices
HIPAA	Health Insurance Portability and Accountability Act
ICH	International Council for Harmonization
ICMJE	International Committee of Medical Journal Editors
ID	Identification
IRB	Institutional review board
INR	International normalized ratio
MOP	Manual of procedures
MRA	MR angiogram
MRI	Magnetic resonance imaging
MRS	Modified Rankin Scale
MSM	Medical safety monitor
NCC	NIH StrokeNet National Coordinating Center
NDMC	NIH StrokeNet National Data Management Center
NIH	National Institutes of Health
NIHSS	National Institutes of Health Stroke Scale
NINDS	National Institute of Neurological Disorders and Stroke
NOAC	Non-vitamin K antagonist oral anticoagulant drug
NT-proBNP	Amino terminal pro-B-type natriuretic peptide

OHRP	Office of Human Research Protections
PHI	Protected health information
PI	Principal Investigator
PM	Project manager
PROMIS	Patient-Reported Outcomes Measurement Information System
PT	Prothrombin time
$PTFV_1$	P-wave terminal force in ECG lead V_1
PTT	Partial thromboplastin time
PUDS	Public use data sets
QC	Quality control
QVSFS	Questionnaire for Verification of Stroke-Free Status
SAE	Serious adverse event
SAP	Statistical analysis plan
SOP	Standard operating procedure
TEE	Transesophageal echocardiogram
TTE	Transthoracic echocardiogram
UP	Unanticipated problem

STATEMENT OF COMPLIANCE

The trial will be conducted in accordance with the International Council for Harmonization (ICH) E6, the Code of Federal Regulations on the Protection of Human Subjects (45 CFR Part 46), and the NINDS Terms of Award. The co-Principal Investigators (co-PIs) will assure that no deviation from or changes to the protocol will take place without prior agreement from NINDS and documented approval from the central institutional review board (cIRB), except where necessary to eliminate an immediate hazard to the trial participants. All personnel involved in the conduct of this study have completed human subjects protection training.

We agree to ensure that all staff members involved in the conduct of this study are informed about their obligations in meeting the above commitments.

PI: Hooman Kamel, MD

Signed: ____ Date: 1 Dec 2017

PI: Mitchell S.V. Elkind, MD, MS

Signed: ____ Date: 1 Dec 2017

PI: W.T. Longstreth, Jr, MD, MPH

Signed: ____ Date: 1 Dec 2017

PI: David Tirschwell, MD, MSc

Signed: ____ Date: 1 Dec 2017

AtRial Cardiopathy and Antithrombotic Drugs In prevention After cryptogenic stroke
Protocol Identifying Number: NCT03192215

PROTOCOL ACCEPTANCE FORM / SIGNATURE PAGE

Version: 3.0
Date: 1 Dec 2017

By signing below I confirm that:

1. I have read this protocol and it contains all necessary details for conducting this study

AND

2. I agree to conduct the trial in compliance with this protocol and to adhere to all regulations that govern the conduct of the study.

______________________________ ______________
Principal Investigator's Signature **Date**

____
Principal Investigator's Name (Print)

____
Site Name

PROTOCOL SUMMARY

Title: AtRial Cardiopathy and Antithrombotic Drugs In prevention After cryptogenic stroke.

Précis: ARCADIA is a multicenter, biomarker-driven, randomized, double-blind, active-control, phase 3 clinical trial of apixaban versus aspirin in patients who have evidence of atrial cardiopathy and a recent stroke of unknown cause. Eleven hundred subjects will be recruited over 2.5 years at 120 sites in the NIH StrokeNet consortium. Subjects will be followed for a minimum of 1.5 years and a maximum of 4 years for the primary efficacy outcome of recurrent stroke and the primary safety outcomes of symptomatic intracranial hemorrhage and major hemorrhage other than intracranial hemorrhage.

Objectives:

- *Primary*: To test the hypothesis that apixaban is superior to aspirin for the prevention of recurrent stroke in patients with cryptogenic ischemic stroke and atrial cardiopathy.
- *Secondary*: To test the hypothesis that the relative efficacy of apixaban over aspirin increases with the severity of atrial cardiopathy.

Endpoints:

- *Primary efficacy outcome*: Recurrent stroke of any type (ischemic, hemorrhagic, or of undetermined type).
- *Primary safety outcomes*: (A) Symptomatic intracranial hemorrhage (including symptomatic hemorrhagic transformation of an ischemic stroke), and (B) major hemorrhage other than intracranial hemorrhage.
- *Secondary efficacy outcomes*: (A) Composite outcome of recurrent ischemic stroke or systemic embolism, and (B) composite outcome of recurrent stroke of any type or death from any cause.
- *Secondary safety outcome*: All-cause mortality.

Population: 1,100 patients with a recent embolic stroke of undetermined source (ESUS) and evidence of atrial cardiopathy.

Phase: Phase 3.

Number of sites: 120.

Study agent:

- *Active agent*: Apixaban (trade name Eliquis®) 5 mg by mouth twice daily (2.5 mg twice daily if standard criteria for adjusted dose are met).
- *Active control*: Aspirin 81 mg by mouth once daily.

Study duration: 5 years.

Participant duration: Minimum of 1.5 years and maximum of 4 years.

SCHEMATIC OF STUDY DESIGN

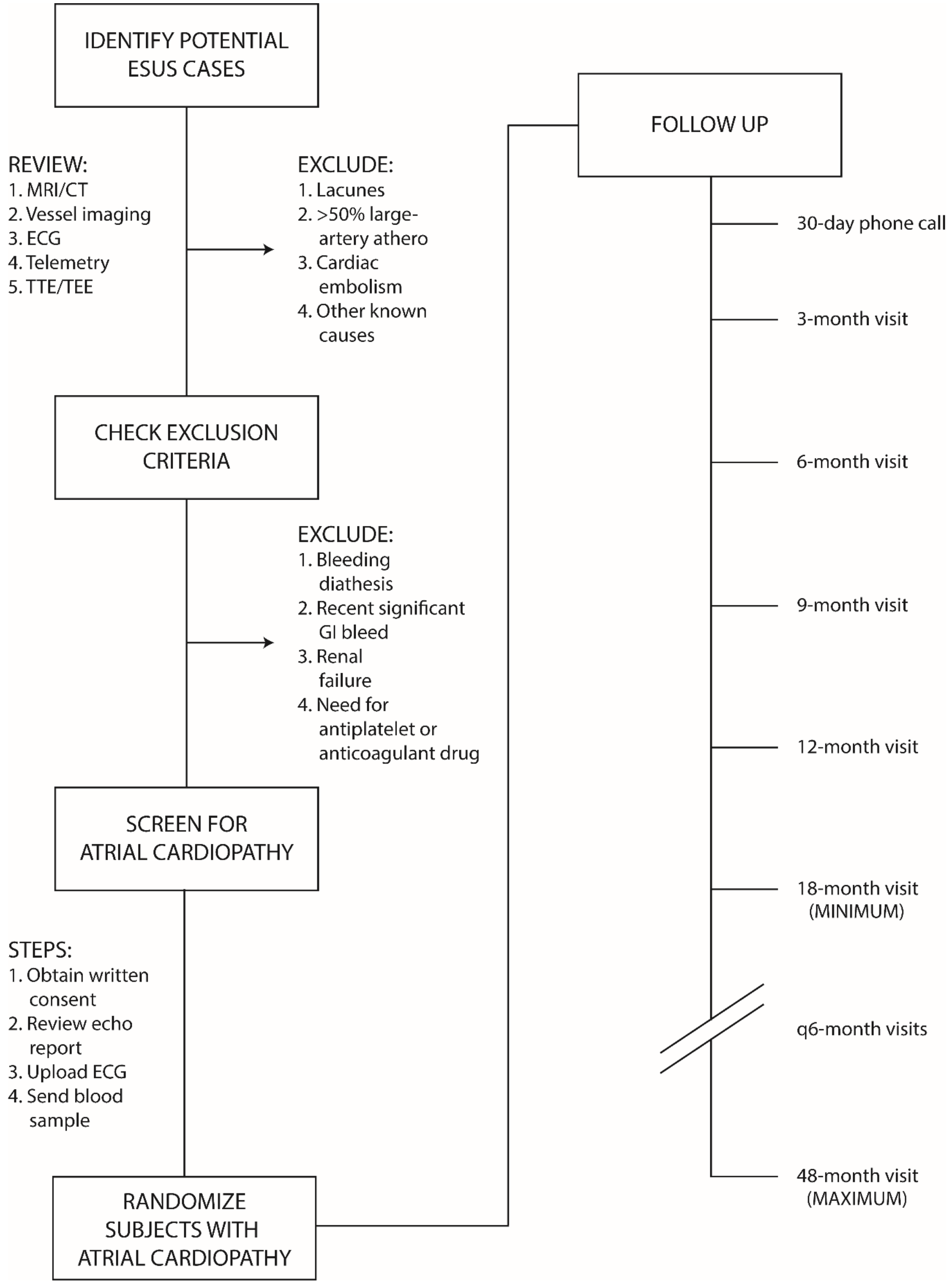

1 KEY ROLES

**Contact PI**	Mitchell S. V. Elkind, MD, MS<br>mse13@cumc.columbia.edu
**Protocol PI**	Hooman Kamel, MD<br>hok9010@med.cornell.edu
**Co-PI, Director of Eligibility Core and Recruitment Core**	David Tirschwell, MD<br>tirsch@uw.edu
**Co-PI, Director of Outcomes Adjudication Core**	W.T. Longstreth, Jr, MD, MPH<br>wl@uw.edu
**Blinded Statistician**	Richard Kronmal, PhD<br>kronmal@uw.edu
**StrokeNet NCC PI**	Joseph Broderick, MD<br>broderjp@ucmail.uc.edu
**StrokeNet NCC Administrative Director**	Judith Spilker, RN, BSN<br>judith.spilker@uc.edu
**ARCADIA NCC Project Manager**	Irene Ewing, RN<br>ewingi@ucmail.uc.edu
**StrokeNet NDMC PI**	Yuko Palesch, PhD<br>paleschy@musc.edu
**Unblinded Statistician, ARCADIA NDMC PI**	Caitlyn Ellerbe, PhD<br>ellerbcn@musc.edu
**NDMC Data Operations Manager**	Catherine Dillon, MS, CCRP<br>rileycp@musc.edu
**ARCADIA NDMC Data Manager**	Cassidy Conner<br>connerc@musc.edu
**ARCADIA NDMC Site Monitoring Manager**	Erin Klintworth<br>klintwor@musc.edu
**ARCADIA Central Pharmacy Manager**	Elizabeth Costea, MS, PharmD<br>strokenetcpharm@ucmail.uc.edu
**Medical Safety Monitor**	David Gladstone, MD, PhD<br>david.gladstone@sunnybrook.ca

Laboratory Director
Eldad A. Hod, MD
eh2217@cumc.columbia.edu

Director of Echocardiography Core
Marco R. Di Tullio, MD
md42@cumc.columbia.edu

Director of ECG Core
Elsayed Z. Soliman, MD, MAS
esoliman@wakehealth.edu

Contact Person for VA Sites
Seemant Chaturvedi, MD
schaturvedi@med.miami.edu

NINDS Project Scientists
Claudia Moy, PhD, MPH
moyc@ninds.nih.gov

Scott Janis, PhD
janiss@ninds.nih.gov

2 INTRODUCTION: BACKGROUND AND SCIENTIFIC RATIONALE

2.1 BACKGROUND

In one-third of ischemic strokes, a specific cause cannot be identified.[1] Many of these cryptogenic strokes appear to arise from a distant embolic source.[2] Recent evidence suggests that some cryptogenic strokes may arise from left atrial thromboembolism that goes unrecognized because it has not manifested with atrial fibrillation/flutter (AF). In the ASSERT study, which showed that even a single 6-minute episode of subclinical AF was associated with a heightened risk of stroke,[3] 31% of patients with both subclinical AF and stroke had no episodes of AF during a median 8 months of continuous cardiac monitoring before the stroke, only manifesting AF for the first time after the stroke.[4] These findings were closely replicated in the more recent IMPACT trial.[5] Such a lack of temporal relationship between AF and stroke undermines the concept that AF itself directly causes cardiac embolism, and is more consistent with AF as a marker of a diseased left atrium characterized by inflammation, endothelial dysfunction, fibrosis, contractile dysfunction, structural derangement, or some combination of these factors. Supporting this possibility, other markers of left atrial abnormality are associated with stroke in the absence of AF. Clinical diagnoses of paroxysmal supraventricular tachycardia have been associated with stroke risk in patients without AF.[6] More recently, increased P-wave terminal force in electrocardiogram (ECG) lead V_1 ($PTFV_1$)—a well-established marker of left atrial abnormality[7]—has been associated with stroke even in the absence of AF.[8-11] Left atrial size on echocardiogram has also been correlated with stroke risk in the absence of AF.[12-15] Lastly, serum levels of NT-pro-BNP, a marker of cardiac myonecrosis and elevated filling pressures, correlate with stroke risk independent of AF.[16,17] These data suggest that an underlying atrial cardiopathy may provide the substrate for thrombus formation and embolization even in the absence of AF.[18] These observations may also explain why fewer than one-third of patients with cryptogenic stroke in the CRYSTAL-AF trial manifested AF even after 3 years of continuous heart-rhythm monitoring.[19]

In parallel with these new insights about cryptogenic stroke, new therapeutic options for stroke prevention have become available. Non-vitamin K antagonist oral anticoagulant (NOAC) drugs such as apixaban may be more effective than aspirin for treatment of cryptogenic stroke. A benefit is especially likely in cryptogenic stroke patients with atrial cardiopathy because of parallels with AF and because an analysis of data from the WARSS/APASS studies suggests that cryptogenic stroke patients with one marker of atrial cardiopathy (elevated NT-proBNP) and no obvious AF benefit from anticoagulant therapy.[20] Apixaban is a particularly attractive choice because it has a low bleeding risk,[21,22] lowers mortality more than warfarin in patients with AF,[23] and is the only NOAC drug with a Class 1A recommendation in recent AHA/ASA guidelines.[24]

Among patients with causes of cryptogenic stroke other than atrial cardiopathy, data are less compelling on the efficacy of anticoagulant therapy.[25] Many cryptogenic strokes may be due to artery-to-artery embolism from non-stenotic and unrecognized large-artery atherosclerosis.[26-28] In patients with stroke from known intracranial atherosclerosis, the WASID trial found no benefit of warfarin over aspirin[29] and the SAMMPRIS trial found historically low rates of recurrence with dual antiplatelet therapy.[30] The ARCH trial demonstrated a trend towards fewer recurrent strokes with antiplatelet therapy compared to anticoagulant therapy in those with aortic arch atheroma.[31] These data suggest that new oral anticoagulant agents are unlikely to be superior to antiplatelet therapy in cryptogenic stroke patients without atrial cardiopathy.

2.2 RATIONALE

These data justify a randomized trial of apixaban versus aspirin specifically in patients with cryptogenic stroke who have evidence of atrial cardiopathy. This trial design will address several important knowledge gaps. First, it will advance our understanding of stroke pathophysiology by assessing whether atrial cardiopathy is a valid therapeutic target, which may set the stage for a primary prevention trial. Second, this trial will advance our understanding of optimal secondary stroke prevention therapy. One industry-sponsored trial (NAVIGATE-ESUS) that compared rivaroxaban to aspirin in patients with cryptogenic stroke was stopped early for futility. Another industry-sponsored trial (RESPECT-ESUS) is comparing dabigatran versus aspirin in patients with cryptogenic stroke. By including all cryptogenic stroke patients and by including those with up to 6 minutes of AF per day, these trials have mixed patients with heterogeneous stroke mechanisms. These trials may thus fail to show an overall benefit (as in NAVIGATE-ESUS), or may show an overall benefit driven mostly by patients with known AF and atrial cardiopathy, resulting in an overly broad indication for anticoagulant therapy. In the first instance, our trial may ensure that a valuable treatment in a specifically targeted subgroup will not be prematurely abandoned. In the second instance, the results of our trial would provide a compelling rationale to perform subgroup analyses of the industry-sponsored trials and conduct future trials to determine the risks and benefits of anticoagulant therapy across biologically distinct subgroups of stroke patients. Given the expense and risks of bleeding associated with anticoagulant drugs, it is imperative to define as precisely as possible the groups of stroke patients who would and would not benefit from their use. Therefore, our approach comports well with the general move toward precision medicine.

2.3 POTENTIAL RISKS AND BENEFITS

2.3.1 KNOWN POTENTIAL RISKS

Major hemorrhage. Both treatment arms (apixaban versus aspirin) will involve antithrombotic medications that necessarily increase the risk for hemorrhage. Despite its associated bleeding risks, the benefit of aspirin in secondary stroke prevention has long been established to outweigh these risks, and aspirin receives a Class I, Level of Evidence A recommendation in American Heart Association guidelines on secondary stroke prevention.[24] Apixaban is a much newer drug than aspirin, and as an anticoagulant drug, it has a theoretically higher risk of bleeding than an antiplatelet drug such as aspirin. However, in the secondary stroke prevention substudy of the AVERROES trial, which enrolled a comparable population of patients as in our proposed trial, the rate of major hemorrhage was not significantly higher with apixaban compared to aspirin.[32]

Recurrent stroke. A 7% annual risk of recurrent stroke is anticipated. It is hypothesized that patients in the intervention arm (apixaban) will face a 40% lower relative risk of recurrent stroke compared to those in the active-control arm receiving standard therapy (aspirin).

Delay of or exclusion from treatment with thrombolytic drugs for recurrent ischemic stroke. Patients actively taking oral anticoagulant drugs such as apixaban are generally excluded from treatment with intravenous thrombolysis for acute ischemic stroke.

Discovery of incidental conditions. This risk is expected to be low because the only diagnostic test that will be performed solely for the purposes of the proposed trial is the collection of a blood sample that will be assayed for NT-proBNP. NT-proBNP can reflect ventricular as well as atrial dysfunction, but all

patients will undergo echocardiography and those with significant heart failure will be excluded prior to the collection of this blood sample. Therefore, an elevated NT-proBNP level in this study will be most likely to reflect atrial cardiopathy rather than another incidental condition. The other diagnostic tests used in this trial—echocardiography and ECG—will have been done as part of standard care and will have been interpreted by clinicians at each site, so that usual clinical protocols will be in place to document and manage incidental findings.

Loss of confidentiality. It is expected that this risk will be low because of numerous safeguards that will be in place, including standard data management procedures at the NDMC.

Discomfort from phlebotomy. Patients who consent to screening for atrial cardiopathy will need to undergo collection of a blood sample for an NT-proBNP assay. Discomfort from this procedure will be minimized by adding on this blood sample to already-scheduled phlebotomies for routine, clinically indicated laboratory tests whenever possible.

Allergic reaction or liver injury. It is expected that these risks will be low based on existing data.

2.3.2 KNOWN POTENTIAL BENEFITS

A successful outcome in the proposed trial would have immediate implications for secondary stroke prevention by identifying a new group of stroke patients who benefit from anticoagulant therapy. Such a result would answer a significant unmet need by providing robust evidence to guide directed treatments for cryptogenic stroke.[2] Such a result would also have near-term implications for primary stroke prevention. Any success of anticoagulant therapy in reducing stroke risk in this high-risk population with atrial cardiopathy would suggest the possibility of benefit in patients with atrial cardiopathy and no history of stroke. If anticoagulation can prevent recurrent stroke from atrial cardiopathy, it should also prevent stroke from occurring in the first place. Thus, validation of atrial cardiopathy as a therapeutic target may set the stage for primary prevention trials. These likely potential benefits, for which we have compelling preliminary data, are highly likely to outweigh the potential risks of the interventions in this trial, both of which involve FDA-approved antithrombotic agents in widespread clinical use and with good safety data in comparable populations.[32] In addition to the benefits to public health, this information could also be of benefit to individual participants in the study, as their own future secondary stroke prevention treatment after the completion of this trial would be influenced by its results.

3 OBJECTIVES AND PURPOSE

The primary aim is to test the hypothesis that apixaban is superior to aspirin for the prevention of recurrent stroke in subjects with cryptogenic ischemic stroke and atrial cardiopathy.

The secondary aim is to test the hypothesis that the relative efficacy of apixaban over aspirin increases with the severity of atrial cardiopathy.

4 STUDY DESIGN AND ENDPOINTS

4.1 DESCRIPTION OF THE STUDY DESIGN

Multicenter, biomarker-driven, randomized, double-blind, active-control, phase 3 clinical trial of apixaban versus aspirin.

4.2 STUDY ENDPOINTS

4.2.1 PRIMARY ENDPOINTS

Efficacy: Recurrent stroke of any type (ischemic, hemorrhagic, or of undetermined type).
Safety: (A) Symptomatic intracranial hemorrhage (including symptomatic hemorrhagic transformation of an ischemic stroke), and (B) major hemorrhage other than intracranial hemorrhage.

4.2.2 SECONDARY ENDPOINTS

Efficacy: (A) Composite of recurrent ischemic stroke or systemic embolism, and (B) composite of recurrent stroke of any type or death from any cause.
Safety: All-cause mortality.

4.2.3 EXPLORATORY ENDPOINTS

See the manual of procedures (MOP) for definitions of other endpoints used for exploratory efficacy and safety analyses: AF, any intracranial hemorrhage, major hemorrhage including any intracranial hemorrhage, symptomatic hemorrhagic transformation of an ischemic stroke, transient ischemic attack, myocardial infarction, minor hemorrhage, systemic embolism, symptomatic deep venous thrombosis, symptomatic pulmonary embolism, ischemic vascular death, and hemorrhagic vascular death.

4.2.4 DEFINITIONS OF PRIMARY AND SECONDARY ENDPOINTS

**Stroke:**	• Composite of ischemic stroke, hemorrhagic stroke, or stroke of undetermined type.
**Ischemic stroke:**	• Rapid onset of a new focal neurological deficit: 1) imaging or other evidence of infarction in a part of the central nervous system consistent with symptoms OR 2) lasting ≥24 hours without imaging evidence of infarction, AND 3) not attributable to a non-ischemic etiology, such as intracranial hemorrhage, edema, infection, trauma, tumor, seizure, severe metabolic disease, or degenerative neurological disease.
**Hemorrhagic stroke:**	• Acute extravasation of blood into the brain parenchyma, subarachnoid space, or intraventricular space, judged to be non-traumatic and not in the area of an acute or subacute ischemic infarct but associated with and identified as the predominant

	cause of new neurologic symptoms, including headache, or leading to death.
**Stroke of undetermined type:**	• Rapid onset of a new focal neurological deficit: 1) lasting ≥24 hours AND 2) not meeting criteria for ischemic stroke or hemorrhagic stroke AND 3) not attributable to a non-vascular etiology, such as edema, infection, trauma, tumor, seizure, severe metabolic disease, or degenerative neurological disease.
**Symptomatic intracranial hemorrhage:**	• Any extravascular blood within the cranium, including subdural and epidural blood, associated with and identified as the predominant cause of new neurologic symptoms, including headache, or leading to death.
**Major hemorrhage other than intracranial hemorrhage:**	• Clinically overt bleeding accompanied by a ≥2 g/dL decrease in the hemoglobin level during a 24-hour period, transfusion of ≥2 units of whole blood or red cells, involvement of a critical non-intracranial site (intraspinal, intraocular, pericardial, intraarticular, intramuscular with compartment syndrome, or retroperitoneal), or death.[33]
**Systemic embolism:**	• Clinical history consistent with an acute loss of blood flow to a peripheral artery or arteries and supported by evidence of embolism from surgical specimens, autopsy, angiography, or other objective testing.

4.2.5 ADJUDICATION OF ENDPOINTS

Study endpoints will be adjudicated using procedures defined in the MOP and approved by the NINDS-appointed DSMB. Regulatory reporting of SAEs and AEs of special interest will not be delayed for this adjudication to occur.

5 STUDY ENROLLMENT AND WITHDRAWAL

5.1 PARTICIPANT INCLUSION CRITERIA

- Age ≥45 years.
- Clinical diagnosis of ischemic stroke + brain imaging to rule out hemorrhagic stroke.
- Modified Rankin Scale (MRS) score ≤4.
- Ability to be randomized no later than 120 days after stroke onset.
- ESUS, defined as **all** of the following[2]:
  - Stroke that is not lacunar. Lacunar is defined as a subcortical (this includes pons and midbrain) infarct in the distribution of the small, penetrating cerebral arteries whose largest dimension is ≤1.5 cm on CT, ≤2.0 cm on MRI diffusion images, or ≤1.5 cm on MRI T2-weighted images. The following are not considered lacunes: multiple simultaneous small

deep infarcts, lateral medullary infarcts, and cerebellar infarcts. Patients with a clinical lacunar stroke syndrome and no infarct on imaging are excluded.

- Absence of extracranial or intracranial atherosclerosis causing ≥50% luminal stenosis of the artery supplying the area of ischemia. Patients must undergo vascular imaging of the extracranial and intracranial vessels using either catheter angiography, CT angiogram (CTA), MR angiogram (MRA), or ultrasound, as considered appropriate by the treating physician and local principal investigator. Training will be provided to the investigators that will encourage the use of CTA and MRA over ultrasound for the evaluation of patients to minimize operator-dependent variation.
- No major-risk cardioembolic source of embolism, including AF, intracardiac thrombus, mechanical prosthetic cardiac valve, atrial myxoma or other cardiac tumors, mitral stenosis, myocardial infarction within the last 4 weeks, left ventricular ejection fraction <30%, valvular vegetations, or infective endocarditis). Patent foramen ovale is not an exclusion. All patients must undergo electrocardiogram, transthoracic or transesophageal echocardiography (TTE or TEE), and at least 24 hours of cardiac rhythm monitoring (Holter monitor or telemetry or equivalent). Additional cardiac imaging, such as cardiac MRI or cardiac CT, will be performed at the discretion of the local treating physician and principal investigator. Additional cardiac rhythm monitoring, such as monitored cardiac outpatient telemetry (MCOT) or an implanted cardiac monitor, will be at the discretion of the treating physician and local principal investigator. Although standard-of-care echocardiography will be used to make diagnoses of cryptogenic stroke and for atrial cardiopathy criteria, these echocardiograms will be centrally read as well to confirm local readings and provide feedback to local sites.
- No other specific cause of stroke identified, such as arteritis, dissection, migraine, vasospasm, drug abuse, or hypercoagulability. Special testing, such as toxicological screens, serological testing for syphilis, and tests for hypercoagulability, will be performed at the discretion of the treating physician and local principal investigator. Training will be provided to local investigators to consider obtaining hypercoagulability tests among patients with patent foramen ovale.

5.2 PARTICIPANT EXCLUSION CRITERIA

- History of AF, AF on 12-lead ECG, or any AF of any duration during heart-rhythm monitoring prior to randomization.
- Clear indication for treatment-dose anticoagulant therapy, such as venous thromboembolism or a mechanical heart valve.
- Left ventricular ejection fraction <30%.
- Definite indication for antiplatelet agent (e.g., aspirin or clopidogrel after implantation of a coronary artery stent).
- History of spontaneous intracranial hemorrhage.
- Chronic kidney disease with serum creatinine ≥2.5 mg/dL.
- Active hepatitis or hepatic insufficiency with Child-Pugh score B or C (see MOP for definition).
- Clinically significant bleeding diathesis.
- Anemia (hemoglobin <9 g/dL) or thrombocytopenia (<100 x 10^9/L) that is chronic in the judgment of the investigator.

- GI bleeding within the past year considered clinically significant by the investigator.
- Pregnancy risk:
    - Female patient who is known to be pregnant.
    - Female patient who is sexually active and premenopausal without a negative pregnancy test performed after stroke onset.
    - Female patient who is sexually active and premenopausal, and who does not commit to adequate birth control.
    - Male patient who is sexually active with a premenopausal female partner, and who does not commit to adequate birth control.
- Known allergy or intolerance to aspirin or apixaban.
- Concomitant participation in another clinical trial involving a drug or acute stroke intervention.
- Considered by the investigator to have a condition that precludes follow-up or safe participation in the trial.
- Inability to obtain written, informed consent from patient or surrogate for trial participation.

5.3 INFORMED CONSENT FOR SCREENING AND RANDOMIZATION

If a patient fulfills all of the inclusion/exclusion criteria, they will be approached for consent, according to local regulations.

Patients who provide consent at this stage will be considered consented but not randomized. Patients will be randomized only if they meet ≥1 of the atrial cardiopathy criteria below.

5.4 ATRIAL CARDIOPATHY CRITERIA REQUIRED FOR RANDOMIZATION

To be eligible for randomization, patients must meet criteria for atrial cardiopathy in addition to the inclusion/exclusion criteria above. Atrial cardiopathy is defined as ≥1 of the following:

- $PTFV_1$ >5,000 µV*ms on 12-lead ECG (ECG criterion).
- Serum NT-proBNP >250 pg/mL (NT-proBNP criterion).
- Left atrial diameter index ≥3 cm/m^2 on echocardiogram (i.e., severe left atrial enlargement) (ECHO criterion).

5.5 STRATEGIES FOR RETENTION AND RECRUITMENT

5.5.1 TIME PERIOD FOR RECRUITMENT

Subject recruitment is planned to be carried out over 2.5 years. Study subjects will be followed for a minimum of 1.5 years and a maximum of 4 years.

5.5.2 INCLUSION OF WOMEN AND MINORITIES

Inclusion or exclusion of subjects will not differ based on sex/gender, and the aim is to recruit similar numbers of men and women. The absence of an upper age limit will aid recruitment of women given that they are generally older than men at the time of a first cardiovascular event such as stroke.

Inclusion or exclusion of subjects will not differ based on race/ethnicity. To ensure representative enrollment of minority groups, site PIs and their clinical research staff will be trained and periodically reminded to:

- Be mindful of cultural, historical, social, and political factors that may influence minority participation. It should be noted that many individuals (particularly certain racial/ethnic groups) need clarification on what a clinical trial is; understanding of what type of research is being conducted; who is conducting the research study, benefits to individuals and the community; place of worship's encouragement, etc.
- Emphasize that the goal is to recruit patients regardless of race or ethnicity.
- Clearly explain that the purpose of informed consent is to protect, not relinquish, participants' rights.
- Provide time if necessary between screening and randomization for patients to reflect and discuss participation with family.
- Emphasize the proven track record of the study drugs.
- Emphasize the trial's potential to help find better treatments to prevent stroke.
- Mitigate concern about randomization by clearly explaining its rationale and highlighting the possibility of cross over if clinically indicated.
- Emphasize that participants will be kept up to date on scientific developments in this field throughout their participation in the trial.
- Think about modes of communication for outreach and retention, such as follow-up phone calls, social media campaigns, text messaging, to identify what works best for the community/population (as allowed by local regulations).
- Provide translation services or bilingual staff, if needed for recruiting populations for which English is not the primary language.
- Use motivational interviewing techniques as a method to improve communication between the research coordinator and patient population to enhance recruitment efforts. This includes investigators and staff taking the time to understand the patient's personal circumstances that may well affect enrollment and follow-up.

5.6 PARTICIPANT WITHDRAWAL OR TERMINATION

5.6.1 REASONS FOR WITHDRAWAL OR TERMINATION

An investigator may discontinue study drug if, in the investigator's opinion, any clinical AE, laboratory abnormality, or other medical condition or situation occurs such that continued use would not be in the best interest of the participant. Additionally, participants are free to discontinue study drug at any time. Subjects who discontinue study drug will continue to be followed and study data collected through the end of the study.

Alternatively, participants are free to withdraw entirely from participation in the study at any time. Subjects who withdraw consent for study participation will not be followed and further study data will not collected. Every effort must be made to undertake protocol-specified safety follow-up procedures to capture SAEs, clinical outcomes, and AEs of special interest prior to study discontinuation.

5.6.2 HANDLING OF PARTICIPANT WITHDRAWALS OR TERMINATION

All randomized participants will be analyzed according to the intention-to-treat principle and therefore will be analyzed in the group they were assigned to regardless of premature discontinuation of study drug, withdrawal from the study, or changes in study treatment.

5.7 PREMATURE TERMINATION OR SUSPENSION OF STUDY

This study may be temporarily suspended or prematurely terminated if there is sufficient reasonable cause. Written notification, documenting the reason for study suspension or termination, will be provided by the suspending or terminating party to site investigators, NINDS, and the StrokeNet Data and Safety Monitoring Board (DSMB). If the study is prematurely terminated or suspended, the co-PIs will promptly inform the cIRB and will provide the reason(s) for the termination or suspension.

Circumstances that may warrant termination or suspension include but are not limited to:

- Determination of unexpected, significant, or unacceptable risk to participants.
- Insufficient compliance to protocol requirements.
- Data that are not sufficiently complete and/or evaluable.
- Demonstration of efficacy or futility (see Section 10.4.4).

The study may resume once concerns about safety, protocol compliance, and data quality are addressed and satisfy any concerns of NINDS, the DSMB, and the cIRB.

6 STUDY AGENT

6.1 STUDY AGENT AND CONTROL DESCRIPTION

6.1.1 ACQUISITION

Study drug and matching placebo will be supplied by the BMS-Pfizer Partnership. Study drug will be sent from BMS-Pfizer to the NCC Central Pharmacy, which will label the study drug bottles, distribute study drug to the sites as they are released to enroll, and resupply sites with study drug as recruitment progresses.

6.1.2 FORMULATION, APPEARANCE, PACKAGING, AND LABELING

Study treatments to be administered are active apixaban or matching placebo and active aspirin or matching placebo. Blinding will be provided by the use of the same color of formulations, same drug packaging, and same method of drug administration in every subject.

6.1.3 PRODUCT STORAGE AND STABILITY

Study product should be stored under controlled room temperature at 15°C to 25°C (59°F-77°F). The NCC Central Pharmacy must be notified of known temperature excursions beyond this range. Excursions

must be reviewed by the study team before product that has experienced a known excursion may be used.

6.1.4 DISTRIBUTION

Local site pharmacies will distribute study drugs to enrolled participants every 3 months either in person or by mail depending on each site's local practice in accordance with local laws and regulations. Study drug will be provided in 90-day supply bottles, with a buffer of 10 day supply of extra pills to account for a delayed resupply visit. At the time of each resupply, subjects will return unused study drug to the investigator/coordinator.

6.1.5 DOSING

Active treatment will be either apixaban 5 mg or aspirin 81 mg. An adjusted dose of apixaban 2.5 mg will be used for subjects with ≥2 of the following: age ≥80 years, body weight ≤60 kg, or known serum creatinine ≥1.5 mg/dL. Thus, there will be six possible study tablets: apixaban 5 mg (regular dose), apixaban 2.5 mg (adjusted dose), apixaban 5 mg placebo, apixaban 2.5 mg placebo, aspirin 81 mg, and aspirin placebo. This dosing schema is consistent with previous trials of stroke prevention in AF.[21,32] Aspirin will be used as the active control agent since it is the proven, standard-of-care antithrombotic drug recommended for patients with stroke.[24]

6.1.6 ADMINISTRATION

All subjects will be randomized to receive active treatment with either active apixaban or active aspirin. Study treatments will be supplied in a double-dummy fashion as aspirin 81 mg or matching placebo, and as apixaban 5 mg (2.5 mg for the adjusted dose) or matching placebo. Study participants will take 1 tablet of aspirin or placebo once daily by mouth from Bottle 1 and 1 tablet of apixaban or placebo twice daily by mouth from Bottle 2. Starting at the randomization visit and then at regular intervals thereafter until study completion, subjects will be provided with two high-density polyethylene pill bottles. Depending on the treatment assignment, Bottle 1 will contain placebo aspirin and Bottle 2 will contain active apixaban, or Bottle 1 active aspirin and Bottle 2 will contain placebo apixaban.

The first doses of study medication must be initiated within 24 hours of randomization.

If a dose of study drug is not taken at the scheduled time, the dose should be taken as soon as possible on the same day,except if less than 6 hours from the next dose, then the missed dose should be skipped and the usual schedule of administration should be resumed. The dose should not be doubled to make up for a missed dose.

6.1.7 LABORATORY MONITORING AND DOSE ADJUSTMENTS/INTERRUPTIONS

The package insert for apixaban does not recommend regular monitoring of laboratory parameters such as creatinine or liver function tests. Thus, such tests are not required as part of this study. Site investigators and study coordinators will be educated, via training at the start of the study and regular study newsletters, that a reduced dose of apixaban study drug is required if subjects meet two of the following three criteria, either at baseline or as identified during the course of their participation in the

trial (e.g., clinically identified change in renal function or age change): age ≥80 years, body weight ≤60 kg, or serum creatinine ≥1.5 mg/dL. The MOP provides details on switching doses if necessary.

6.1.8 TRACKING OF DOSE

Study drug adherence will be assessed using pill counts at each study visit. For subjects who have intentionally stopped study drug, the reason for discontinuation or temporary interruption will be collected (e.g., elective procedure, new contraindication to treatment, etc).

6.2 STUDY DRUG ACCOUNTABILITY PROCEDURES

Upon receipt of the study drug, the site pharmacist or designee will inspect the study drug supply and confirm receipt of the study drug in the WebDCU™, the study's clinical trial management system. The pharmacist or pharmacy designee at each clinical site must also document in WebDCU™ study drug dispensing, return, and destruction. Drug accountability records and storage temperature logs may be inspected by the study monitor or subjected to inspection by relevant authorities.

7 STUDY PROCEDURES AND SCHEDULE

7.1 STUDY PROCEDURES/EVALUATIONS

7.1.1 STUDY-SPECIFIC PROCEDURES

**Medical history:**	• Obtained at baseline.<br>• Should focus on inclusion/exclusion criteria and vascular comorbidities.<br>• Can be obtained from up-to-date medical records and/or interview.
**Physical examination:**	• Obtained at baseline.<br>• Should focus on inclusion/exclusion criteria.
**Vital signs:**	• Obtained at baseline.<br>• Should include height and weight to allow calculation of the body mass index (BMI).
**NIHSS:**	• Obtained in person at baseline by a certified investigator at baseline.
**QVSFS:**	• Validated screening tool for identifying strokes.[34]<br>• Obtained at the randomization visit and all follow-up visits.<br>• Should be obtained via telephone if subjects cannot make in-person study visits.<br>• Should be obtained from a proxy if necessary.

	• Medical records should be reviewed to confirm reports of possible strokes.
**mRS:**	• Obtained at baseline and at the q6-month follow-up visits by a certified investigator.<br>• Should be obtained via telephone if subjects cannot make an in-person q6-month study visit.<br>• Should be obtained from a proxy if necessary.
**Blood sample:**	• Obtained at baseline and sent to Laboratory Core.<br>• See MOP for instructions for collection and shipment.
**PROMIS scales:**	• Global Health Form and Physical Function Short Form.[35]<br>• Obtained in person at the 12-month follow-up visit.
**SAEs, clinical outcome events, and AEs of special interest:**	• Obtained at all follow-up visits.<br>• Should be obtained via telephone if subjects cannot make in-person study visits.<br>• Should be obtained from a proxy if necessary.
**Medication adherence:**	• Pill counts should be documented at each follow-up visit.<br>• Reasons for discontinuation/interruption should be documented if applicable.
**Concomitant medications:**	• Subjects should be asked about antiplatelet and anticoagulant drugs at baseline and each follow-up visit.

7.1.2 STANDARD-OF-CARE STUDY PROCEDURES

All tests below must be performed after the index stroke. Results of tests performed prior to stroke onset cannot be used even if recent.

**Brain imaging:**	• Either CT or MRI is acceptable.
**Vascular imaging:**	• Acceptable modalities include ultrasound, CTA, MRA, or catheter angiogram.<br>• Both the cervical and intracranial cerebral circulation must be evaluated.
**12-lead ECG:**	• The first technically adequate ECG done after stroke onset should be used.<br>• See MOP for instructions on uploading a copy of the ECG to WebDCU™.
**Continuous heart-rhythm monitoring:**	• ≥24 hours required.

- Either hospital telemetry or formal Holter monitoring is acceptable.
- Hospital telemetry does not require an automated AF detection algorithm if reviewed by investigators or other clinical staff.

Echocardiogram:

- The first technically adequate echocardiogram done after stroke onset should be used.
- Either TTE or TEE is acceptable.
- See MOP for instructions on sending echocardiogram images.

Laboratory tests:

- Chemistry: creatinine.
- CBC: white blood cell count, hemoglobin, hematocrit, platelet count.
- Coagulation studies: PT, PTT, INR.
- Liver function tests: total bilirubin, ALT, AST, alkaline phosphatase.
- Pregnancy test, if applicable.

7.2 STUDY SCHEDULE

7.2.1 SCREENING

Study subjects are expected to be recruited from both the inpatient setting, such as from an acute stroke service, and the outpatient setting, such as from a stroke clinic.

The investigators at each site will be required to maintain a screen failure log for patients with ESUS who are found ineligible to participate in the study, documenting the patients' age, demographics, and the reason(s) for exclusion from the current study. The study coordinator at each site is required to enter the screen failure log data into WebDCU™ on a monthly basis.

Screening for Atrial Cardiopathy

Among patients who provide consent for trial participation, only those who meet at least one atrial cardiopathy criterion will be randomized. A hotline coordinated via the Eligibility Core will be available to help with reviewing screening data and determining eligibility.

- To determine whether a patient meets the ECHO criterion, the site investigator will determine from the report of the clinically performed echocardiogram whether the patient has severe left atrial enlargement, defined as left atrial diameter index ≥3 cm/m^2. All echocardiograms will be sent via procedures outlined in the MOP to the Echocardiography Core at Columbia for additional measurements to be used in secondary analyses.

- To determine whether a patient meets the ECG criterion, the first ECG done as part of the standard stroke evaluation will be used. A copy of this ECG will be sent via procedures outlined in the MOP to the ECG Core at Wake Forest for standardized measurement of $PTFV_1$. The ECG

Core will enter the assessment of $PTFV_1$ into WebDCU™ within 2 business days of receipt of the ECG so that eligibility can be determined.

- To determine whether a patient meets the BNP criterion, a blood sample will be sent via procedures outlined in the MOP to the study Laboratory Core at Columbia for NT-proBNP measurement. The Laboratory Core will enter the NT-proBNP measurement into WebDCU™ within 2 business days of receipt of the blood sample so that eligibility can be determined.

Baseline Visit (Day 0-120 after stroke onset)

- Ensure that required standard-of-care tests have been performed (see Section 7.1.2).
- Review medical history, medications, and standard-of-care tests to confirm eligibility based on inclusion/exclusion criteria (see Sections 5.1 and 5.2).
- Obtain informed consent of potential participant verified by signature on written informed consent form.
- Perform physical examination including vital signs and NIHSS, as well as mRS.
- Collect blood samples for NT-proBNP assay and potential future use and ship to Laboratory Core (see MOP).
- Upload copy of 12-lead ECG to WebDCU™ (see MOP).
- Send a copy of echocardiogram images to the Echocardiography Core (see MOP).
- Schedule Randomization Visit. This visit can occur later during the index hospitalization or at a subsequent clinic visit (randomization must occur on or before Day 120 after stroke onset).

7.2.2 RANDOMIZATION

Randomization should occur as close to the first administration of study drug as possible. In general, randomization can occur as early as post-stroke day 3. However, randomization must be delayed until at least post-stroke day 14 for patients with severe strokes (initial NIHSS score ≥11), hemorrhagic transformation of the index stroke, or uncontrolled hypertension. For all strokes, randomization must occur no later than post-stroke day 120. The calendar day (12:00 a.m. through 11:59 p.m.) of stroke onset is considered post-stroke day 0. If the time of onset of the stroke is unclear, the day of the first presentation for medical care will be considered post-stroke day 0.

Randomization Visit (Day 3-120 after stroke onset)

- Rescreen participants immediately prior to randomization. Review medical history, medications, QVSFS, and physical examination including vital signs. Participants must continue to meet all inclusion and exclusion criteria at the time of randomization. None of the tests specified in Section 5.1 need to be repeated as part of the rescreening process; investigators should simply screen for interval events that would make the patient ineligible (e.g., development of spontaneous intracranial hemorrhage, AF, recurrent stroke). If any of the tests in Section 5.1 have been repeated as part of standard clinical care, those results should be reviewed to ensure continued eligibility.
- Once it is determined that a patient meets all the randomization criteria, the site investigator or their designee will log on to WebDCU™ to enter the required randomization data. The database

will generate the randomization which will correlate to the appropriate study drug to be retrieved from the study pharmacy.

- Those who are not considered eligible for randomization due to the absence of all of the three atrial cardiopathy biomarkers may still be followed, as part of ancillary studies, for clinical outcomes through telephone calls and review of medical records and insurance claims. Permission for follow up of this group is included in the study consent form.

7.2.3 FOLLOW-UP

Follow-Up Visit 1 (30 ± 7 days after randomization)

- Contact participants via telephone and assess eligibility for study medication, study drug safety, QVSFS, SAEs, clinical outcome events, AEs of special interest, medication adherence, and concomitant medications.

Follow-up Visit 2 (90 days ± 5 days after randomization)

- Schedule in-person visit to assess eligibility for study medication, study drug safety, QVSFS, SAEs, clinical outcome events, AEs of special interest, medication adherence, and concomitant medications.

Follow-up Visit 3 (180 days ± 5 days after randomization)

- Schedule in-person visit to assess eligibility for study medication, study drug safety, QVSFS, mRS, SAEs, clinical outcome events, AEs of special interest, medication adherence, and concomitant medications.

Follow-up Visit 4 (270 days ± 5 days after randomization)

- Schedule in-person visit to assess eligibility for study medication, study drug safety, QVSFS, SAEs, clinical outcome events, AEs of special interest, medication adherence, and concomitant medications.

Follow-up Visit 5 (360 days ± 5 days after randomization)

- Schedule in-person visit to assess eligibility for study medication, study drug safety, QVSFS, mRS, SAEs, clinical outcome events, AEs of special interest, medication adherence, concomitant medications, ambulatory status, and PROMIS quality-of-life scales.

Follow-up Visits 6 (through completion of subject participation, minimum 540 days)

- Schedule in-person visits every 180 days ± 5 days to assess eligibility for study medication, study drug safety, QVSFS, mRS, SAEs, clinical outcome events, AEs of special interest, medication adherence, and concomitant medications.
- Medication resupply will need to occur at the 3-month mid-point which will require assessing QVSFS, SAEs, clinical outcome events, and AEs of special interest.

7.2.4 FINAL STUDY VISIT

At the end of the study, or at the time of subject withdrawal from the study or premature treatment discontinuation, the site investigator will be responsible for choosing and initiating appropriate antithrombotic therapy. Subjects should be treated at least with aspirin (or open-label anticoagulation if they have documented AF). There will also be a close-out telephone visit 30 days after the end of study drug treatment to ascertain SAEs, clinical outcome events, and AEs of special interest.

7.2.5 UNSCHEDULED VISIT

An unscheduled visit should be arranged for SAEs, clinical outcome events, AEs of special interest, or other developments that in the opinion of the site investigator necessitate an in-person evaluation to ensure the subject's safe ongoing participation in the study.

7.2.6 SCHEDULE OF EVENTS TABLE

Procedure	B	R	30 ±7 days*	90 ±5 days	180 ±5 days	270 ±5 days	360 ±5 days	q180 ±5 days# afterward	Close out$
Eligibility check^	x	x	x	x	x	x	x	x	
Consent	x								
Randomization form		x							
Medical history	x	x							
QVSFS		x	x	x	x	x	x	x	
Modified Rankin Scale	x				x		x	x	
Vital signs	x	x							
Physical examination	x	x							
NIHSS	x								
Brain imaging	o								
Vascular imaging	o								
12-lead ECG	o								
≥24 hrs cardiac monitoring	o								
Echocardiogram	o								
Serum chemistry	o								
Complete blood count	o								
Coagulation studies	o								
Serum liver function tests	o								
Pregnancy test, if applicable	o								
Blood sample to core lab	x								
ECG to core lab	x								
Echo to core lab	x								
PROMIS Global Health							x		
PROMIS Phys. Func. Short							x		
Ambulatory status							x		
Safety assessment			x	x	x	x	x	x	x
Study drug safety			x	x	x	x	x	x	
Medication adherence			x	x	x	x	x	x	
Medication resupply				x	x	x	x	x	
Concomitant med.	x	x	x	x	x	x	x	x	

B = Baseline visit; R = Randomization visit; QVSFS = Questionnaire for Verification of Stroke-Free Status.
* Telephone visit.
# This visit includes medication resupply at mid-point (3 months) which will require assessing QVSFS SAEs, clinical outcomes, and AEs of special interest. These should occur within ±5 days of the 3-month mark.
$ Telephone visit 30 days after study drug discontinuation. On average participants will have approximately 33 months of follow-up (minimum 18 months, maximum 48 months).
^ After randomization, eligibility check will involve ensuring continued suitability for study drug administration.
o Cost billed as standard of care.
x Cost covered by study.

7.3 CONCOMITANT AND PROHIBITED MEDICATIONS

7.3.1 ANTIPLATELET MEDICATIONS

If an antiplatelet agent is newly indicated after randomization (e.g., aspirin or clopidogrel after implantation of a coronary artery stent), then study drug must be stopped until the antiplatelet agent is stopped.

For subjects who were receiving antiplatelet therapy prior to their qualifying stroke, there is no high-quality evidence to support switching to another antiplatelet agent empirically or based on the results of platelet resistance assays.[24] Therefore, subjects receiving aspirin, clopidogrel, or aspirin/dipyridamole should be considered eligible for this trial and randomization to either aspirin or apixaban monotherapy. All baseline antiplatelet therapy will be stopped after randomization.

If the site investigator feels that a short course of dual antiplatelet therapy is indicated, randomization cannot occur until after this course is completed.

7.3.2 ANTICOAGULANT MEDICATIONS

The first dose of study drug cannot be given until at least 12 hours after the last dose of an anticoagulant (heparin, enoxaparin, etc), even if at a prophylactic dose. Guidelines from the AHA/ASA recommend prophylactic-dose anticoagulation for "treatment of immobilized subjects to prevent DVT."[36] For immobilized subjects who are receiving prophylactic-dose anticoagulation per these guidelines, randomization should be performed at a time such that study drug is not started until after discontinuation of prophylactic-dose anticoagulation.

After randomization, if open-label anticoagulant therapy is newly indicated at a full treatment dose, such as for newly diagnosed venous thromboembolism, or at a prophylactic dose during an intercurrent hospitalization and/or a rehabilitation stay, then study drug must be stopped until open-label anticoagulation is stopped.

7.4 DETECTION AND MANAGEMENT OF ATRIAL FIBRILLATION

The use of continuous heart-rhythm monitoring after randomization is allowed. Subjects who, as part of standard-of-care follow-up/testing after randomization, manifest AF of any duration, as determined by the judgment of the site investigator and treating physicians, should be switched to open-label anticoagulant therapy per the discretion of the site investigator and treating physicians. We recommend but do not mandate switching to open-label apixaban using the same dosing as the study protocol. These subjects will continue to be followed for outcome events in the study according to the intention-to-treat paradigm.

7.5 INTERRUPTION OF STUDY DRUG FOR ELECTIVE INVASIVE PROCEDURES

Unblinding will not be performed for elective procedures. Investigators will be educated and reminded that subjects have a 50% chance of being on anticoagulation, and investigators will be provided with and referred to guidelines from the American Academy of Neurology on periprocedural management of

antithrombotic medications in subjects with ischemic cerebrovascular disease.[37] For further reference, the FDA label for apixaban states: "ELIQUIS should be discontinued at least 48 hours prior to elective surgery or invasive procedures with a moderate or high risk of unacceptable or clinically significant bleeding. ELIQUIS should be discontinued at least 24 hours prior to elective surgery or invasive procedures with a low risk of bleeding or where the bleeding would be non-critical in location and easily controlled." Site investigators and study coordinators will be educated about these guidelines through training at study initiation and through regular study newsletters.

7.6 MANAGEMENT OF BLEEDING

In cases of bleeding that do not meet the definition of serious bleeding, subjects should be advised to not take further doses of study drugs until the bleeding has stopped and the investigator judges that the benefits of resuming study drug outweigh the risk of recurrent bleeding.

The following steps should be taken in all cases of serious bleeding:

- Further doses of study drug should be held until the bleeding is controlled and the investigator judges that the benefits of resuming study drug outweigh the risk of recurrent bleeding.
- Standard measures should be taken to control and mitigate the effects of bleeding, such as local control of the bleeding source if possible and administration of intravenous fluids and blood products as necessary.

If it is considered likely that bleeding cannot be managed with only the steps above, and that measures specific to reversal of apixaban are required, treating physicians and/or site investigator can perform unblinding by calling the study hotline. The MOP provides detailed guidance on management of serious bleeding in subjects assigned to apixaban.

7.7 MANAGEMENT OF ACUTE ISCHEMIC STROKE IN SUBJECTS ON STUDY DRUG

If intravenous thrombolysis is being considered for the acute treatment of recurrent ischemic stroke, the treating physicians and/or site investigators can call the study hotline for unblinding. After unblinding, subjects assigned to aspirin can be treated with intravenous thrombolysis if indicated per each site's standard practice.

Subjects assigned to apixaban may be at an increased risk of bleeding if treated with intravenous thrombolysis unless all of the following conditions are met:

- The subject or surrogate can confirm that no study drug has been taken for the past 48 hours;
- The subject's renal function is normal (GFR ≥60);
- The subject's INR and PTT values are normal;
- Intravenous thrombolysis is otherwise indicated per the site's standard practice.

Unblinding should only occur for subjects who would be eligible for treatment only if they were on aspirin and not on apixaban. Unblinding is discouraged for subjects who are not eligible regardless of being on aspirin or apixaban, or subjects who meet all of the criteria above and may be able to receive thrombolysis while being on apixaban.

7.8 VASCULAR RISK FACTOR MANAGEMENT

Investigators will be provided with and referred to the most recent guidelines from the AHA/ASA on secondary stroke prevention.[24] Site investigators and study coordinators will be educated about these guidelines through training at study initiation and through regular study newsletters.

7.9 PARTICIPANT ACCESS TO STUDY DRUG AFTER STUDY CLOSURE

Study drug will not be provided free of charge to participants after study closure. Similarly, participants who switch to open-label anticoagulant therapy due to interval diagnosis of AF will no longer receive study drug free of charge.

If a site identifies an event as a possible primary efficacy outcome, the subject will either continue, pause, or stop study medication at the discretion of the treating physician. If the adjudication committee determines the event meets the primary efficacy definition the subject will stop study medication; otherwise they may continue or resume treatment at the discretion of the treating physician.

8 ASSESSMENT OF SAFETY

8.1 SPECIFICATION OF SAFETY PARAMETERS

Primary safety endpoints: (A) Symptomatic intracranial hemorrhage (including symptomatic hemorrhagic transformation of an ischemic stroke), and (B) major hemorrhage other than intracranial hemorrhage.

Secondary safety endpoints: All-cause mortality.

The following exploratory safety endpoints will also be recorded: AF, any intracranial hemorrhage, major hemorrhage including any intracranial hemorrhage, symptomatic hemorrhagic transformation of an ischemic stroke, transient ischemic attack, myocardial infarction, minor hemorrhage, systemic embolism, symptomatic deep venous thrombosis, symptomatic pulmonary embolism, ischemic vascular death, and hemorrhagic vascular death. The endpoint of any intracranial hemorrhage will be subclassified as: 1) symptomatic versus asymptomatic, and 2) consisting of hemorrhagic transformation of the index brain infarct versus not.

8.1.1 DEFINITION OF ADVERSE EVENTS

An AE is defined as any new untoward medical occurrence or worsening of a preexisting medical condition in a clinical investigation participant administered study drug and that does not necessarily have a causal relationship with this treatment. An AE can therefore be any unfavorable and unintended sign (such as an abnormal laboratory finding), symptom, or disease temporally associated with the use of investigational product, whether or not considered related to the investigational product. Adverse events can be spontaneously reported or elicited during open-ended questioning, examination, or evaluation of a subject. For the purposes of this trial, only AEs that meet the definition of serious, clinical outcomes, and AE of special interest will be reported.

8.1.2 DEFINITION OF SERIOUS ADVERSE EVENTS

An SAE is any untoward medical occurrence that at any dose:

- results in death;
- is life-threatening (defined as an event in which the participant was at risk of death at the time of the event; it does not refer to an event which hypothetically might have caused death if it were more severe);
- requires inpatient hospitalization or causes prolongation of existing hospitalization;
- results in persistent or significant disability/incapacity;
- is a congenital anomaly/birth defect;
- is an important medical event (defined as a medical event(s) that may not be immediately life-threatening or result in death or hospitalization but, based upon appropriate medical and scientific judgment, may jeopardize the subject or may require intervention [e.g., medical, surgical] to prevent one of the other serious outcomes listed in the definition above. Examples of such events include, but are not limited to, intensive treatment in an emergency room or at home for allergic bronchospasm; blood dyscrasias or convulsions that do not result in hospitalization.)
- results in suspected transmission of an infectious agent (e.g., pathogenic or nonpathogenic) via the study drug.

The definition of SAE excludes the following hospitalizations:

- A visit to the emergency room or other hospital department < 24 hours, that does not result in admission (unless considered an important medical or life-threatening event);
- Elective surgery, planned prior to signing consent;
- Admissions as per protocol for a planned medical/surgical procedure;
- Routine health assessment requiring admission for baseline/trending of health status (e.g., routine colonoscopy);
- Medical/surgical admission other than to remedy ill health and planned prior to entry into the study (appropriate documentation is required in these cases);
- Admission encountered for another life circumstance that carries no bearing on health status and requires no medical/surgical intervention (e.g., lack of housing, economic inadequacy, caregiver respite, family circumstances, administrative reason).

A life-threatening adverse event is defined as an event in which the participant was at risk of death at the time of the event; it does not refer to an event that hypothetically might have caused death if it were more severe.

Important medical events that may not result in death, be life-threatening, or require hospitalization may be considered serious when, based upon appropriate medical judgment, they may jeopardize the patient or subject and may require medical or surgical intervention to prevent one of the outcomes listed in this definition. Examples of such medical events include allergic bronchospasm requiring intensive treatment in an emergency room or at home, blood dyscrasias or convulsions that do not result in inpatient hospitalization, or the development of drug dependency or drug abuse. Other study-specific SAEs are defined in the MOP.

8.1.3 ADVERSE EVENTS OF SPECIAL INTEREST

Pregnancy, overdose, potential drug-induced liver injury, and cancer are AEs of special interest and will be assessed at each visit and reported.

Potential or suspected cases of liver injury:
This includes but is not limited to liver test abnormalities, jaundice, hepatitis, or cholestasis.

Pregnancy:
If, following initiation of the investigational product, it is subsequently discovered that a study participant is pregnant or may have been pregnant at the time of investigational product exposure, including during at least 5 half-lives after product administration, the investigational product will be permanently discontinued in an appropriate manner (e.g., dose tapering if necessary for participant).

Protocol-required procedures for study discontinuation and follow-up must be performed on the participant. Follow-up information regarding the course of the pregnancy, including perinatal and neonatal outcome and, where applicable, offspring information must be reported on a Pregnancy Surveillance Form.

Any pregnancy that occurs in a female partner of a male study participant will also be reported. Information on this pregnancy will be collected on the Pregnancy Surveillance Form. In order for the investigators to collect any pregnancy surveillance information from the female partner, the female partner must sign an informed consent form for disclosure of this information.

Overdose:
An overdose is defined as the accidental or intentional administration of any dose of a product that is considered both excessive and medically important.

8.1.4 UNANTICIPATED PROBLEMS AND UNANTICIPATED EVENTS

The Office for Human Research Protections (OHRP) considers *unanticipated problems* (UPs) involving risks to participants or others to include, in general, any incident, experience, or outcome that meets **all** of the following criteria:

- Unexpected in terms of nature, severity, or frequency given (a) the research procedures that are described in the protocol-related documents, such as the cIRB-approved research protocol and informed consent document; and (b) the characteristics of the participant population being studied;
- Related or possibly related to participation in the research (“possibly related” means there is a reasonable possibility that the incident, experience, or outcome may have been caused by the procedures involved in the research); and
- Suggests that the research places participants or others at a greater risk of harm (including physical, psychological, economic, or social harm) than was previously known or recognized.

This study will use the OHRP definition of UP.

Unanticipated events include UPs, but may also include other events that do not rise to the level of UPs as outlined above. These include protocol deviations or other unexpected problems that do not necessarily pose a safety concern, and will be reported according to cIRB requirements and StrokeNet Standard Operating Procedures as defined in detail in the MOP.

8.2 CLASSIFICATION OF AN ADVERSE EVENT

8.2.1 SEVERITY OF EVENT

The severity of SAEs, clinical outcome events, and AEs of special interest will be reported using the grading system outlined in the NCI Common Terminology Criteria for Adverse Events Version 4.03 (CTCAE). The CTCAE provides a grading (severity) scale for each AE term and AEs are listed alphabetically within categories based on anatomy or pathophysiology. The CTCAE (v4.03) displays Grades 1-5 with unique clinical descriptions of severity for each AE based on this general guidance:

CTCAE Severity Grading Summary	
Grade 1:	Mild AE
Grade 2:	Moderate AE
Grade 3:	Severe or Disabling AE
Grade 4:	Life-Threatening AE
Grade 5:	Death related to AE

The complete definitions of these grades are:

- Grade 1: Mild; asymptomatic or mild symptoms; clinical or diagnostic observations only; intervention not indicated AE.
- Grade 2: Moderate; minimal, local or noninvasive intervention indicated; limiting age-appropriate instrumental activities of daily living (preparing meals, shopping for groceries or clothes, using the telephone, managing money, etc.).
- Grade 3: Severe or medically significant but not immediately life-threatening; hospitalization or prolongation of hospitalization indicated; disabling; limiting self-care activities of daily living (bathing, dressing and undressing, feeding self, using the toilet, taking medications, and not bedridden).
- Grade 4: Life-threatening consequences; urgent intervention indicated.
- Grade 5: Death related to AE.

8.2.2 RELATIONSHIP TO STUDY AGENT

For each SAE, clinical outcome event, and AE of special interest, the relationship to the study treatment will be assessed and documented using the following algorithm:

Algorithm to Determine Relatedness of Adverse Event to Study Agent	
**Not Related**	The temporal relationship between treatment exposure and the adverse event is unreasonable or incompatible and/or adverse event is clearly due to extraneous causes (e.g., underlying disease, environment)
**Unlikely**	**Must have both of the following** 2 conditions, but may have reasonable or only tenuous temporal relationship to intervention:
	1. Could readily have been produced by the subject's clinical state, or environmental or other interventions.<br>2. Does not follow known pattern of response to intervention.
**Reasonable Possibility**	**Must have at least 2** of the following 3 conditions:
	1. Has a reasonable temporal relationship to intervention.<br>2. Could not readily have been produced by the subject's clinical state or environmental or other interventions.<br>3. Follows a known pattern of response to intervention.
**Definitely**	**Must have all 3** of the following conditions:
	1. Has a reasonable temporal relationship to intervention.<br>2. Could not possibly have been produced by the subject's clinical state or have been due to environmental or other interventions.<br>3. Follows a known pattern of response to intervention.

8.2.3 EXPECTEDNESS

The independent Medical Safety Monitor (MSM) will be responsible for determining whether an SAE is **expected** or **unexpected**. An SAE will be considered unexpected if the nature, severity, or frequency of the event is not consistent with the risk information previously described for the study agent.

8.3 TIME PERIOD AND FREQUENCY FOR EVENT ASSESSMENT AND FOLLOW-UP

At each study visit, the investigator will inquire about the occurrence of SAEs, clinical outcome events, and AEs of special interest since the last visit. Any significant worsening noted during interim or final physical examinations, electrocardiograms, x-rays, and any other potential safety assessments, whether or not these procedures are required by the protocol, may meet the definition of SAE, clinical outcome event, or AE of special interest, and should be reported accordingly.

The site investigator will report all SAEs, clinical outcome events, and AEs of special interest occurring after randomization and prior to the last day of study participation. All such events will be captured on the appropriate CRF and entered into WebDCU™. Information to be collected includes event description, time of onset, clinician's assessment of severity, relationship to study product (assessed only by those with the training and authority to make a diagnosis), time of resolution/stabilization of the event, a description of the event, relevant history, and concomitant medications/procedures. All SAEs, clinical outcome events, and AEs of special interest will be followed for outcome information until resolution or until 30 days after the subject's participation in the study ends. The information in WebDCU™ will be updated as more information becomes available.

8.4 REPORTING PROCEDURES

8.4.1 ADVERSE EVENT REPORTING

This study is being conducted as an investigator-initiated, NINDS-funded protocol. The FDA has granted a waiver for an investigational new drug (IND) application. Thus, no formal safety reporting to FDA will be done.

Only AEs that meet the definition of an SAE, clinical outcome event, or AE of special interest will be recorded and reported per the reporting timelines. All reporting will be done in accordance with StrokeNet Standard Operating Procedures for Safety Monitoring and Reporting as outlined in the administrative documents available on the website (https://www.nihstrokenet.org/docs/default-source/default-document-library/adm13-safety-monitoring-and-reporting-12-19-16.pdf?sfvrsn=0).

8.4.2 SAFETY REPORTING

Site Responsibilities

Site investigators or their designees must report SAEs, clinical outcome events, and AEs of special interest through WebDCU™ within 24 hours of site awareness of the event. The investigators are required to provide relevant information such as a description of the event, date/time of onset and resolution, severity, suspected relationship to the study treatment, and action taken. Supporting documentation of the event should be provided as soon as possible. Additional supporting documentation may be requested by the NCC and should also be provided as soon as possible.

All SAEs, clinical outcome events, and AEs of special interest, whether related or not related to study drug, must be collected from the time of randomization through 30 days post study drug discontinuation or end of study, whichever occurs later.

All SAEs, clinical outcome events, and AEs of special interest should be followed to resolution or stabilization.

NDMC and NCC Responsibilities (Sites should ignore these two paragraphs)

All SAEs and AEs of special interest will be reported to BMS via a Safety Report generated in WebDCU™ for review by their Global Pharmacovigilance group (GPV&E) in accordance with global regulatory reporting requirements. SAEs and AEs of special interest, whether related or unrelated to the study drug, and pregnancies must be reported to BMS by NDMC within 72 hours. These events must be recorded on an approved form and sent to the BMS SAE Email Address: Worldwide.Safety@BMS.com, SAE Fax Number: 609-818-3804, or other address as provided by BMS. If only limited information is initially available, follow-up reports are required. Note: Follow-up safety reports should include the same investigator term(s) initially reported. If an ongoing event changes in its intensity or relationship to study drug or if new information becomes available, a follow-up safety report should be sent within 72 hours to the BMS (or designee) using the same procedure used for transmitting the initial report. The NCC will reconcile the clinical database with safety events transmitted to BMS GPV&E. Reconciliation will occur every 3 months and once just prior to database lock/final study report. The investigator will request a safety data reconciliation report from aepbusinessprocess@bms.com or other address

provided by BMS. BMS GPV&E will email, upon request from the investigator, the GPV&E reconciliation report. The data elements listed on the GPV&E safety data reconciliation report will be used for case identification purposes. If the investigator determines a case was not transmitted to BMS GPV&E, the case will be sent immediately.

Via a separate safety monitoring mechanism, the MSM will conduct an independent review of each SAE to determine its seriousness, relationship to the study treatment, and expectedness. If the MSM adjudicates the event to be serious, unexpected, and study related, the event will be reported expeditiously to the cIRB via a Safety Report generated in WebDCU™.

8.4.3 UNANTICIPATED EVENT REPORTING

Clinical sites will report unanticipated events, including UPs and protocol deviations, in the WebDCU™ per the guidelines listed in the MOP. Unanticipated events that are unexpected, related to study participation, and place the subject/others at increased risk will require prompt reporting to the cIRB. All other unanticipated events which do meet the above criteria will be reported to the cIRB at the time of continuing review.

8.5 STUDY OVERSIGHT

Safety oversight will be under the direction of the NIH StrokeNet DSMB, which is composed of individuals with the appropriate expertise in overseeing stroke clinical trials. The DSMB will meet at least semiannually to assess safety data on each arm of the study. The DSMB will review data quality and completeness, monitor fidelity to the study protocol, review the adequacy of participant recruitment and retention, review SAEs, clinical outcome events, and AEs of special interest and make recommendations to the NINDS and the study co-PIs concerning trial continuation, modification, or conclusion.

8.6 STUDY HALTING RULES

The MSM is responsible for ongoing monitoring of reports of SAEs by the clinical centers within 72 hours to ensure Good Clinical Practices (GCP) and to identify safety concerns quickly. The MSM may suggest protocol modifications to prevent the occurrence of particular SAEs, such as modifying the protocol to require frequent measurement of laboratory values predictive of the event or to improve expeditious identification of SAEs. To minimize bias, the MSM will evaluate SAEs blinded to treatment assignment. The NDMC will prepare regular reports concerning SAEs and submit them to the DSMB. In the event of unexpected SAEs or an unduly high rate of SAEs, the MSM will promptly contact the co-PIs and the NINDS Program Official and, if applicable, the NINDS DSMB liaison, who will notify the DSMB Chair. The DSMB will convene an ad hoc meeting by teleconference or in writing as soon as possible. The DSMB will provide recommendations for continuing or halting the study to the NIH and the study co-PIs.

9 CLINICAL MONITORING

Clinical site monitoring is conducted to ensure that the rights and well-being of human subjects are protected, that the reported trial data are accurate, complete, and verifiable, and that the conduct of the trial is in compliance with the currently approved protocol/amendment(s), with GCP, with applicable

FDA regulations (21 CFR 312), and with the FDA's "Guidance for Industry Oversight of Clinical Investigations — A Risk-Based Approach to Monitoring."

- Monitoring for this study will be performed by the NDMC centrally, on site, and remotely.
- Per the study's monitoring plan, monitoring will include a combination of **on-site monitoring** (to verify data entered into the WebDCU™ database against source documents and query inaccuracies between the source documents and WebDCU™ database), **remote monitoring** (source document verification, including verification of written consent, may be performed remotely by reviewing source documents that have been uploaded into WebDCU™ or via remote access to electronic medical records), and **central monitoring** (using web-based data validation rules, data manager review of entered data, statistical analysis, and on-going review of site metrics).
- The NDMC, study co-PIs, and the appropriate site PIs will be provided copies of monitoring reports within 30 days of site visits.

Further details of clinical site monitoring are documented in the study's Monitoring Plan. The Monitoring Plan describes in detail who will conduct the monitoring, at what frequency monitoring will be done, at what level of detail monitoring will be performed, and the distribution of monitoring reports.

10 STATISTICAL CONSIDERATIONS

10.1 STATISTICAL AND ANALYTICAL PLAN

Statistical analysis details are documented in a formal statistical and analytical plan (SAP). The SAP will be finalized prior to the first site being released to enroll subjects.

10.2 STATISTICAL HYPOTHESES

Primary efficacy endpoint: The null hypothesis (H_0) is that the hazard ratio comparing apixaban versus aspirin for the primary endpoint will be 1.

Secondary efficacy endpoint: The null hypothesis (H_0) is that the hazard ratio comparing apixaban versus aspirin for the secondary endpoint will be 1.

10.3 ANALYSIS DATASETS

The analysis datasets will include:

- Intention-to-treat analysis dataset, which will include all randomized participants.
- Safety analysis dataset, which will include all participants who took at least one dose of study drug.
- Per-protocol analysis dataset, which will include all randomized participants who do not have an eligibility violation and who received at least one dose of study drug.

10.4 DESCRIPTION OF STATISTICAL METHODS

10.4.1 GENERAL APPROACH

Efficacy outcomes will be analyzed in the intention-to-treat analysis dataset. Safety outcomes will be analyzed in the safety analysis dataset.

10.4.2 ANALYSIS OF THE PRIMARY AND SECONDARY ENDPOINTS

The log-rank statistic will be used to test the null hypothesis that the hazard ratio is 1, comparing apixaban to aspirin, at a two-tailed alpha level of 0.05.

10.4.3 SAFETY ANALYSES

A standard frequency table will be used to report the number (%) of SAEs, stratified by treatment group. Based on prior reports,[32,38] the following approximate annual rates of major safety endpoints are expected:

Adverse Event	Apixaban	Aspirin
Symptomatic intracranial hemorrhage	1.5%	1.5%
Major hemorrhage other than intracranial hemorrhage	4.0%	3.0%
All-cause mortality	3.5%	3.5%

Log-rank tests will be utilized to assess the treatment group differences in the rates of: (1) first symptomatic intracranial hemorrhage; (2) first major hemorrhage other than intracranial hemorrhage; and (3) death from any cause.

10.4.4 PLANNED INTERIM ANALYSES

The ARCADIA investigators propose one interim analysis for efficacy (or harm) and futility to be performed when 1/2 of the expected events have occurred (75 events), with the details specified in the SAP.

10.4.5 ADDITIONAL SUBGROUP ANALYSES

The primary efficacy and safety analyses will be stratified by age (<75 years versus ≥75 years), sex/gender, and race/ethnicity (non-Hispanic white versus others). Differential effects of apixaban versus aspirin across these subgroups are not anticipated and therefore the trial is not powered specifically for these subgroup analyses, but these analyses will allow any unexpectedly large variations to be identified.

10.4.6 EXPLORATORY AND SENSITIVITY ANALYSES

The SAP lists additional prespecified exploratory and sensitivity analyses, including analyses related to the secondary objective of the trial, which is to test the hypothesis that the relative efficacy of apixaban over aspirin increases with the severity of atrial cardiopathy.

10.5 SAMPLE SIZE

10.5.1 ASSUMPTIONS

The estimated sample size of 1,100 subjects is based on several important assumptions:

- 30-month uniform accrual period and 18-month minimum follow-up period;
- 7% annual risk of recurrent stroke of any type in aspirin-treated subjects;
- 40% relative reduction (hazard ratio, 0.6) in the stroke recurrence rate with apixaban compared to aspirin;
- 3% annual rate of cross-over from blinded aspirin to open-label apixaban because of AF detection;
- 3% annual rate of cross-over from blinded apixaban to open-label aspirin because of bleeding or other SAEs; and
- 5% annual rate of loss to follow-up or death in each group.

10.5.2 SAMPLE SIZE CALCULATION

Given the assumptions above, enrollment of 1,100 subjects (150 recurrent stroke events anticipated) will provide 80% power to demonstrate a statistically significant difference in the hazard for recurrent stroke with apixaban compared to aspirin at a two-sided alpha level of 0.05 while allowing for an interim look for efficacy (or harm) and futility. An O'Brien-Fleming type Lan-DeMets error spending function[39] will be used to perform one interim analysis at the halfway point of the trial. The sample size estimate assumes that the futility boundary will be nonbinding.

10.6 MEASURES TO MINIMIZE BIAS

10.6.1 ENROLLMENT, RANDOMIZATION, AND MASKING PROCEDURES

Eligible patients will be allocated in a 1:1 ratio to apixaban or aspirin using a scheme that balances randomization by regional coordinating center.

To maintain blinding, the BMS-Pfizer Partnership will provide the NCC Central Pharmacy with apixaban and aspirin tablets along with an equal number of matching placebo tablets. Site pharmacists and all investigators and subjects will be blinded throughout the course of the study. Switching to open-label anticoagulation, if indicated, should not result in unblinding of previous treatment status up until that point. Subjects who switch to open-label anticoagulation because of AF or other indication, or who switch to open-label antiplatelet therapy, will continue to be followed and will be analyzed according to the intention-to-treat principle with blinded outcome adjudication. Unblinding should only occur if a compelling clinical reason arises, such as active major bleeding or a pending decision on whether to administer intravenous thrombolysis for acute ischemic stroke, in which case the site investigator can perform emergency unblinding via procedures detailed in the MOP. In the case of unblinding, adjudication of any AEs that led to the unblinding will be performed centrally by adjudicators who remain blinded to the subject's treatment status. Once a participant's treatment is unblinded, that participant will no longer receive study drug.

10.6.2 BREAKING THE STUDY BLIND

This is a blinded study. All co-PIs, site study team members including site pharmacists, and study subjects will be fully blinded to treatment assignment. Unblinding is unlikely to be needed, but in the event that emergency unblinding is requested, unblinding procedures are specified in the MOP. The site investigator should only request unblinding when it is essential for the subject's safety (e.g., when considering administration of intravenous thrombolysis for recurrent acute ischemic stroke, managing life-threatening bleeding, or undertaking emergency surgery).

Participants will be provided with identification that includes study contact information and indicates that they are participating in a study of aspirin versus apixaban for recurrent stroke. This will be useful in the event of a medical emergency. A notification will also be sent to participants' primary care physicians to inform them of their subject's participation in this trial.

11 SOURCE DOCUMENTS AND ACCESS TO SOURCE DATA

Each participating site will maintain appropriate medical and research records for this trial, in compliance with ICH E6 and regulatory and institutional requirements for the protection of confidentiality of participants. As part of participating in an NINDS-sponsored trial, each site will permit authorized representatives of NINDS and regulatory agencies to examine (and when permitted by applicable law, to copy) clinical records for the purposes of quality assurance reviews, audits, and evaluation of the study safety, progress, and data validity.

Data will be collected using CRFs whenever possible, but source data to be collected will also include copies of provider notes, laboratory results, and imaging reports.

Subjects' participation in the study will be documented in the electronic medical record unless prohibited by local regulations.

To allow timely review of informed consent forms, enrolling sites will upload a PDF copy of the signed informed consent form into the password-protected clinical trial management system, WebDCU™. The PDF file will be linked to the subject ID number but will be stored on a secure server separate from the study's CRF data. The secure server on which these files are stored is not backed up, in order to prevent copies of files containing individually identifiable health information from being copied and stored on non-NDMC back up servers. The files on these servers can only be accessed by designated study personnel upon entry of a second password. NDMC staff will remotely monitor the informed consent form. Issues identified during monitoring of these documents will be relayed to the clinical site for corrective and preventative action. After remote monitoring is complete, the PDF file containing the informed consent form will be permanently deleted from the secure server. If a subject must be re-consented, the process will repeat itself.

12 QUALITY ASSURANCE AND QUALITY CONTROL

Quality control (QC) procedures will be implemented beginning with the data entry system, and data QC checks that will be run on the database will be generated. Any missing data or data anomalies will be communicated to the site(s) for clarification/resolution.

Following written procedures as detailed in the monitoring plan, the monitors will verify that the clinical trial is conducted and data are generated, documented (recorded), and reported in compliance with the protocol, GCP, and the applicable regulatory requirements (e.g., Good Laboratory Practices (GLP), Good Manufacturing Practices (GMP)).

The investigational site will provide direct access to all trial-related sites, source data/documents, and reports for the purpose of monitoring and auditing by the sponsor, and inspection by local and regulatory authorities.

13 ETHICS/PROTECTION OF HUMAN SUBJECTS

13.1 ETHICAL STANDARD

The co-PIs will ensure that this study is conducted in full conformity with Regulations for the Protection of Human Subjects of Research codified in 45 CFR Part 46, 21 CFR Part 50, 21 CFR Part 56, and/or the ICH E6.

13.2 INSTITUTIONAL REVIEW BOARD

The protocol, informed consent form, recruitment materials, and all participant materials will be submitted to the StrokeNet and VA cIRBs for review and approval. Approval of both the protocol and the consent form will be obtained before any participant is enrolled. Any amendment to the protocol will require review and approval by the cIRBs before the changes are implemented to the study. All changes to the template consent form will be cIRBs approved; a determination will be made regarding whether previously consented participants need to be re-consented.

13.3 INFORMED CONSENT PROCESS

13.3.1 CONSENT AND OTHER INFORMATION DOCUMENTS PROVIDED TO PARTICIPANTS

Consent forms describing in detail the study agent, study procedures, and risks will be given to the participant and written documentation of informed consent will be performed prior to starting intervention/administering study product. The following consent materials are submitted with this protocol: informed consent form with included HIPAA authorization form.

13.3.2 CONSENT PROCEDURES AND DOCUMENTATION

In accordance with FDA regulations (21 CFR 50) and ICH-GCP Consolidated Guidelines, a witnessed, cIRB-approved, informed consent is required from all patients prior to participating in this study. At the initial contact with a potential candidate, the investigative team (investigator or coordinator) will provide a comprehensive explanation of the purpose, procedures, possible risks/benefits of the study in language that is understandable to a non-medically trained person; as well as participant responsibilities and the fact that his/her participation is voluntary, that he or she may withdraw from the study at any time, and that the decision not to participate or to withdraw will not affect the patient’s care in any way. Potential participants will be given ample opportunity to ask questions and to consider their decision. If the patient expresses a sustained interest, a signed and dated written informed consent will be obtained.

Patients with a known history of dementia will not provide self-consent, thereby minimizing the possibility of invalid informed consent. A copy of the consent form will be given to the participant, and another copy placed in his or her medical record unless prohibited by local regulations. The informed consent will be obtained by either the clinical site PI or other members of the study team who are qualified to perform this task and whose names are listed on the Delegation of Authority Log. Informed consent will be performed in a language in which the patient or surrogate (see below) is fluent. Participants will be asked to explain back the study to confirm their understanding of the study and its procedures.

13.3.3 USE OF SURROGATES FOR CONSENT

Since this is a long-term secondary stroke prevention trial, the goal is to enroll subjects who will be able to participate actively in their care over the duration of the trial. However, recognizing that otherwise suitable subjects will often have neurological deficits that may impair their ability to provide written, informed consent, especially soon after their index stroke, the trial will allow inclusion of subjects via the use of surrogate consent. Loss of capacity to provide consent is a common, though hardly universal, feature in stroke patients. Neurologists and other physicians who care for stroke patients, as well as coordinators involved in stroke trials, are familiar with the signs and symptoms of stroke that can render a stroke patient unable to provide informed consent. All investigators and coordinators will be trained in the process of enrolling stroke patients using informed consent.

The use of surrogates for consent will conform with determinations required by federal regulations for the cognitively impaired:

- 45 CFR 46.111(a): Selection of subjects is equitable. Use of surrogate consent leads to inclusion of a more representative cohort of subjects with stroke and is therefore more equitable than excluding subjects who are otherwise eligible except for their inability to provide consent themselves.
- 45 CFR 46.111(b): Additional safeguards have been included to protect their rights and welfare. Although the active treatment arm of this trial imposes a risk of injury from increased bleeding, compelling preliminary data exists to suggest that subjects receiving apixaban will benefit from improved stroke prevention and that the probability of benefit is greater than the probability of harm. Subjects assigned to aspirin will receive the current standard of care for stroke prevention.
- 45 CFR 46.116: Informed consent must be obtained from the subject or the subject's legally authorized representative.

Our protocol will be the following: First, the lack of capacity will be determined. Acute stroke patients frequently lack capacity due to language deficits, inattention to their health (neglect and anosagnosia, or lack of awareness of their deficit), or other deficits. A trained study investigator will make these initial assessments and this process of determination of lack of capacity will be documented in the medical record.

Second, once the patient is determined to lack capacity, a member of the investigative team will seek to identify a court-appointed legally authorized representative/guardian or appropriately executed health care proxy. This individual will serve as the legally authorized representative to provide surrogate consent.

Third, if no court-appointed legally authorized representative/guardian or appropriately executed health care proxy is available, then a member of the investigative team will obtain surrogate consent from another person on the following list: (a) the spouse (if not legally separated from the subject) or the domestic partner; (b) a son or daughter eighteen (18) years of age or older; (c) a parent; (d) a brother or sister eighteen (18) years of age or older; (e) a close friend (meaning a person eighteen [18] years of age or older who has maintained such regular contact with the subject as to be familiar with the subject's activities, health, and beliefs). Determination of the appropriate individual to provide informed consent will be determined through means considered possible and appropriate by the study investigator or coordinator, including but not limited to presentation and review of documents, discussion with family members present, review of administrative and medical records, and discussion with the patient's primary care physician, who is likely to know these individuals. The discussion will include the patient when possible, even if the patient is unable to provide formal consent due to language or other deficits. In this circumstance, the patient's assent will be sought, when possible. In addition, the discussion will include multiple family members as appropriate in the investigator's judgment, and agreement among family members will be sought.

Finally, if a subject previously determined to lack capacity to consent regains capacity during the study, the investigator will obtain the consent of the individual for the remaining part of the study.

Detailed procedures for determining subjects' capacity for consent and obtaining consent from a surrogate, if necessary, are specified in the MOP. Investigators and coordinators will be trained in the use of surrogates for informed consent.

13.4 PARTICIPANT AND DATA CONFIDENTIALITY

13.4.1 CONFIDENTIALITY AND DATA STORAGE

Participant confidentiality is strictly held in trust by the participating investigators, their staff, and the sponsor(s) and their agents. This confidentiality is extended to cover testing of biological samples in addition to clinical information relating to participants. Therefore, the study protocol, documentation, data, and other information generated will be held in strict confidence. No information about the study or data will be released to any unauthorized third party without prior written approval of the sponsor.

The study monitor, other authorized representatives of the sponsor, and representatives of the NDMC, cIRB, and pharmaceutical company supplying study product may inspect all documents and records required to be maintained by the investigator, including but not limited to, medical records and pharmacy records for the participants in this study. The clinical study site will permit access to such records.

The study participant's contact information will be securely stored at each clinical site for internal use during the study. At the end of the study, all records will continue to be kept in a secure location for the duration specified by the StrokeNet Standard Operating Procedure (SOP) or longer as dictated by cIRB and local institutional regulations.

Study participant research data, which is for purposes of statistical analysis and scientific reporting, will be transmitted to and stored for the duration of the study and analysis at the NDMC. The study data entry and study management systems used by clinical sites and by the NDMC research staff will be

secured and password protected. At the end of the study, all study databases will be de-identified and a Public Use Dataset (PUDS) will be archived at the NINDS data repository.

13.4.2 RESEARCH USE OF STORED HUMAN SAMPLES

Intended Use: Samples collected from participants at the time of screening will be stored and will be used to assay NT-proBNP to determine eligibility for the study. Samples may also be used for other studies of stroke and cardiac disease (see Section 13.5 below). No genetic testing will be performed without further amendment of this protocol and informed consent form.
Storage: The biobank repository will be kept at the Laboratory Core for the study, in the Center for Advanced Laboratory Medicine (CALM) at Columbia University Medical Center (CUMC), under the supervision of Dr. Elkind, Contact PI for the study, and Dr. Eldad Hod, a clinical pathologist and Director of CALM. A dedicated -80° C freezer will be used for this study. Drs. Elkind and Hod, and their delegates, will have responsibility for the integrity of the repository. Access to the repository for future research studies will be limited to those approved by the ARCADIA Executive Committee.

Tracking: Samples and data will be stored using codes. Data will be kept in password-protected computers. Only investigators will have access to the samples and data. Study participants who request destruction of samples will be notified of compliance with such request and all supporting details will be maintained for tracking.

13.5 FUTURE USE OF STORED SPECIMENS

All ARCADIA ancillary studies, including those that include the use of blood specimens, will be reviewed by the Publications and Executive Committees for approval. Specimens will be used only for purposes related to research described in this protocol, i.e., understanding the causes and optimal prevention, treatment, and outcomes of unexplained strokes. The purpose of the Executive and Publications Committee reviews will be to assure that future uses of subject specimens are consistent with the informed consent obtained at the time the specimen was collected. A letter of approval for use of biobank specimens will be provided to the ancillary study PI. Once approved and, if necessary, funded, Dr. Hod's delegated staff will retrieve the samples for assays to be performed locally or for shipping to offsite laboratories as requested and appropriate for the particular study. Investigators who receive specimens from the study will sign forms to indicate that the samples are solely for their use and they will be expressly forbidden from transferring samples to any third parties. The CALM biosample repository will also be provided with a code-link that will allow linking the biological specimens with the phenotypic data from each participant, maintaining the masking of the identity of the participant.

Informed consent for collection and storage of blood samples for the biobank (i.e. blood samples above and beyond that needed to determine NT-pro BNP for study eligibility) is incorporated into the primary ARCADIA consent form, but as a separate section requiring separate approval. Participants will thus have the opportunity to participate in ARCADIA without participating in the biobank. No additional consent will be obtained so as to minimize inconvenience to the participant and the local investigator's staff by collecting the blood samples at a single time. Because collection of a sample for the assay for NT-pro BNP is being performed to determine eligibility for randomization, the collection of limited additional samples for the purpose of this biobank is expected to cause minimal risk and inconvenience, thereby limiting the possibility for interference with the parent ARCADIA study. Participants can withdraw consent for research on stored specimens at any time and the specimens will be discarded; this

information is also included in the consent form. However, data already collected about the participants will be retained and analyzed even if the subjects choose to withdraw from the research. If a subject revokes authorization in writing for continued use or disclosure of blood sample data or protected health information (PHI) that was already obtained in the research, analysis of that data will continue only to the extent necessary to protect the integrity of the research study. Furthermore, withdrawal of consent with regard to biosample storage will not be possible after the study is completed.

The privacy and confidentiality of the biobank will be ensured through the use of standard CALM procedures. All individuals who handle specimens will be trained in accordance with GCP and on specific modules related to the handling of biospecimens. This training will include safeguards to prevent accidental or inappropriate release of information. A list of those with access to the samples will be kept with Dr. Hod as Director of the CALM Laboratory. The samples themselves will be kept in a dedicated freezer in a locked room in the CALM Laboratory; access will be limited to Drs. Hod, Elkind, and their delegates. Any clinical data collected will be handled through WebDCU™. Samples will be shipped to CALM to arrive during business hours. Samples will be accompanied by labels on tubes and a sample shipping form that will include no patient identifiers other than the study ID number. The samples will be stored at CALM using this study ID number only, and samples will be bar coded for ease of storage and retrieval. Any laboratory results will be provided to WebDCU™. Further use of this information will be according to the study-wide procedures for maintenance of confidentiality and privacy. Depending on funding, analyses for ancillary studies may be performed by the NDMC, or, if they are performed locally by an ancillary study investigator, a limited data set, including key clinical variables needed for the analyses, may be provided by the NDMC to enable the analyses to be performed. PHI will not be provided without further authorization by the Executive Committee. A Certificate of Confidentiality will be obtained prior to the beginning of enrollment.

Specimens will be destroyed 10 years after the publication of the primary manuscript describing the results of the ARCADIA study. Samples will be discarded in red medical waste bags specially made to contain medical or biohazardous waste and certification will be provided to the co-PIs and other requestors.

In the event that an ancillary study proposal is approved that uses the specimens and does not involve any investigators related to the main protocol, a sample use agreement will be required. In this case, to minimize the possibility that data in the biobank could be used to identify individuals in the study, we will add to the agreement an additional statement stating that the recipient may not attempt to identify subjects by any means.

Specimens may be made available to commercial organizations. Commercial organizations will need to complete an ancillary study proposal form, which will be reviewed by the Executive and Publications Committees, which includes NINDS representation. Final agreements with commercial organizations will need to be reviewed with legal counsel of the study sponsor, as well, in the event of potential development of commercial products.

14 DATA HANDLING AND RECORD KEEPING

14.1 DATA COLLECTION AND MANAGEMENT RESPONSIBILITIES

Data collection is the responsibility of the clinical trial staff at the site under the supervision of the site PI. The investigator is responsible for ensuring the accuracy, completeness, legibility, and timeliness of the data reported.

All source documents should be completed in a neat, legible manner to ensure accurate interpretation of data. Black ink is required to ensure clarity of reproduced copies. When making changes or corrections, cross out the original entry with a single line, and initial and date the change. DO NOT ERASE, OVERWRITE, OR USE CORRECTION FLUID OR TAPE ON THE ORIGINAL.

Copies of the electronic CRF (eCRF) will be provided for use as source documents and maintained for recording data for each participant enrolled in the study. Data reported in the eCRF derived from source documents should be consistent with the source documents or the discrepancies should be explained and captured in a progress note and maintained in the participant's official electronic study record.

Clinical data will be entered into WebDCU™. The data system includes password protection and internal quality checks, such as automatic range checks, to identify data that appear inconsistent, incomplete, or inaccurate. Clinical data will be entered directly from the source documents.

14.2 STUDY RECORDS RETENTION

Study documents should be retained for the duration specified by the StrokeNet SOP or for a longer period if required by local regulations.

14.3 PROTOCOL DEVIATIONS

A protocol deviation is any noncompliance with the clinical trial protocol, GCP, or MOP requirements. The noncompliance may be either on the part of the participant, the investigator, or the study site staff. As a result of deviations, corrective action plans are to be developed by the site after review and approval by the NCC.
These practices are consistent with ICH E6:

- 4.5 Compliance with Protocol, sections 4.5.1, 4.5.2, and 4.5.3.
- 5.1 Quality Assurance and Quality Control, section 5.1.1.
- 5.20 Noncompliance, sections 5.20.1, and 5.20.2.

It is the responsibility of the site to use continuous vigilance to identify and report deviations within 10 working days of identification of a protocol deviation meeting criteria for prompt reporting, or within 10 working days of the scheduled protocol-required activity. These protocol deviations must be addressed in study source documents and reported via WebDCU™ for review by the ARCADIA Project Manager. All other protocol deviations must be reported to the cIRB at the time of annual reviews. The site PI and study staff are responsible for knowing and adhering to cIRB requirements. Further details about the handling of protocol deviations are included in the MOP.

14.4 PUBLICATION AND DATA SHARING POLICY

This study will comply with the NIH Public Access Policy, which ensures that the public has access to the published results of NIH funded research. It requires scientists to submit final peer-reviewed journal manuscripts that arise from NIH funds to the digital archive PubMed Central upon acceptance for publication. As required by International Committee of Medical Journal Editors (ICMJE) member journals and Section 801 of the Food and Drug Administration Amendments Act (FDAAA) of 2007, this trial will be registered prior to its initiation at clinicaltrials.gov, a public trials registry sponsored by the National Library of Medicine. The co-PIs will take specific steps to ensure compliance with NIH implementation of FDAAA.

Data generated through this project will be shared according to StrokeNet SOP ADM 04 regarding the Network Data Sharing Policy Data Sharing Policy. In summary, the goals of this Data and Resource Sharing Policy are to make available final data from StrokeNet clinical trials to the research community, while safeguarding the privacy of trial participants and protecting confidential and proprietary data.

Upon database lock, the NDMC statisticians will generate data files from each data table corresponding to each electronic CRF in the database. In compliance with the HIPAA regulations, each data table will be stripped of any and all personal identifiers and will undergo a deidentification process. Furthermore, the NDMC statisticians will create a minimum number of derived variables that would be necessary to ensure reproducibility of the primary analysis.

Within 1 year from the acceptance of the primary manuscript for publication OR no later than 2 years from the database lock the PUDS will be submitted to the NINDS data repository, along with the final version of the study protocol, the data dictionary, and a user guide (or a "Readme" file) regarding the data files, including an explanation of any derived variables. Once PUDS are available, any researcher (study investigator or otherwise) wishing to receive them can contact the NINDS (CRLiaison@ninds.nih.gov). All manuscripts, abstracts, and press releases using the study data must acknowledge the StrokeNet investigators and the NINDS as the study sponsor with the relevant grant numbers.

In order to expedite and track external data sharing, the ARCADIA website will serve as a resource and informational site for potential external investigators and collaborators. The website will include the ARCADIA publication policy, the data sharing procedures and policies, timelines, and contact information. All published ARCADIA manuscripts, abstracts, and brief descriptions of ongoing projects will be posted on the website, maintained by the StrokeNet NCC.

15 STUDY ADMINISTRATION

15.1 OVERVIEW

This trial is being conducted as part of the NIH-funded StrokeNet network for stroke clinical trials. Trial administration will be handled by the StrokeNet NCC at the University of Cincinnati, and data management and monitoring by the NDMC at the Medical University of South Carolina. The ARCADIA co-PIs will have overall responsibility for study design, site selection, analysis and interpretation of final data, and dissemination of study results.

15.2 STUDY MANAGEMENT CORE (NCC)

This core will perform study management tasks such as site management and contracting, cIRB oversight, trial-wide communications, orchestration of training activities, coordination of site initiation visits, collection of regulatory documents, and site performance analyses. This core will also receive study drug from BMS-Pfizer, label drugs to maintain blinding, and distribute study drug to sites on a regular schedule.

15.3 DATA MANAGEMENT CORE (NDMC)

The study database will reside at Medical University of South Carolina according to the procedures governing the StrokeNet. This core consists of those responsible for maintenance of the database, logic checks, edit requests, quality assurance, merging of datasets, creating and exporting data subsets, reliability and validity substudies, programming reports and data arrays needed for tracking enrollment and follow-up, and supporting other operational, project management, and analysis needs. This core will interface closely with the ARCADIA co-PIs, co-investigators, and all the committees. This core will provide unblinded reports to the DSMC for their meetings, and will provide a final locked database to the blinded study statistician after data collection and cleaning have been completed.

15.4 EXECUTIVE COMMITTEE

The Executive Committee will consist of the following voting members: the four ARCADIA co-PIs, the blinded study statistician, the study cardiologist, the PIs of the NCC and NDMC, and the NINDS project scientist. Key blinded members of the Trial Operations Committee will also attend. The Executive Committee in its entirety will meet twice annually, and more frequently as needed, to review and if necessary vote on key study issues, including but not limited to: 1) approving modification of the protocol or operations manual, 2) reviewing study progress, 3) mediating budgetary disputes, 4) considering probation or suspension of a clinical center from further patient entry, 5) reviewing and approving issues surrounding local projects and ancillary studies, and 6) reviewing and approving all abstracts and publications.

15.5 TRIAL OPERATIONS COMMITTEE

The Trial Operations Committee will include the four ARCADIA co-PIs, the NCC study pharmacist, the study cardiologist and laboratory director, the PIs of the NCC and NDMC, the NINDS project scientist, the unblinded study statistician, and key members of the NCC and NDMC involved in study operations. Membership will also include individuals representing additional networks of study investigators that may join this trial. This committee will meet twice a month to review recruitment and discuss execution, budget, and other day-to-day issues arising in the conduct of the trial.

15.6 PUBLICATIONS COMMITTEE

Membership of the Publications Committee will be determined by the Executive Committee. The Publications Committee will convene quarterly, and more frequently as needed (i.e., during the dissemination phase after trial completion), to review and make recommendations to the Executive Committee with regard to: 1) paper proposals, 2) prioritization of analysis requests, 3) writing group

membership and authorship issues, 4) other manuscript-related issues. This Committee will function in accordance with the StrokeNet SOP regarding publications policies.

15.7 ANCILLARY STUDIES COMMITTEE

Membership of the Ancillary Studies Committee will be determined by the Executive Committee. The Ancillary Studies Committee is responsible for soliciting and reviewing ancillary study proposals and interfacing with all approved ancillary study teams to ensure their coordination with the parent trial. As needed, the Ancillary Study Committee will interface with the study cores to ensure equitable distribution of samples and data, and with the Publications Committee to review proposed analyses and manuscripts. The Ancillary Study Committee will function in accordance with the StrokeNet SOP regarding ancillary studies.

16 CONFLICT OF INTEREST POLICY

The independence of this study from any actual or perceived external influence is critical. Therefore any actual conflict of interest of persons who have a role in the design, conduct, analysis, publication, or any aspect of this trial will be disclosed and managed. Furthermore, persons who have a perceived conflict of interest will be required to have such conflicts managed in a way that is appropriate to their participation in the trial. The study leadership in conjunction with the NINDS has established policies and procedures for all study group members to disclose all conflicts of interest and will establish a mechanism for the management of all reported dualities of interest.

17 LITERATURE REFERENCES

1. Marnane M, Duggan CA, Sheehan OC, et al. Stroke subtype classification to mechanism-specific and undetermined categories by TOAST, A-S-C-O, and Causative Classification system. *Stroke.* 2010;41(8):1579-1586.
2. Hart RG, Diener HC, Coutts SB, et al. Embolic strokes of undetermined source: the case for a new clinical construct. *Lancet Neurol.* 2014;13(4):429-438.
3. Healey JS, Connolly SJ, Gold MR, et al. Subclinical atrial fibrillation and the risk of stroke. *N Engl J Med.* 2012;366(2):120-129.
4. Brambatti M, Connolly SJ, Gold MR, et al. Temporal relationship between subclinical atrial fibrillation and embolic events. *Circulation.* 2014;129(21):2094-2099.
5. Martin DT, Bersohn MM, Waldo AL, et al. Randomized trial of atrial arrhythmia monitoring to guide anticoagulation in patients with implanted defibrillator and cardiac resynchronization devices. *Eur Heart J.* 2015;36(26):1660-1668.
6. Kamel H, Elkind MS, Bhave PD, et al. Paroxysmal supraventricular tachycardia and the risk of ischemic stroke. *Stroke.* 2013;44(6):1550-1554.
7. Hancock EW, Deal BJ, Mirvis DM, et al. AHA/ACCF/HRS recommendations for the standardization and interpretation of the electrocardiogram: part V: electrocardiogram changes associated with cardiac chamber hypertrophy: a scientific statement from the American Heart Association Electrocardiography and Arrhythmias Committee, Council on Clinical Cardiology; the American College of Cardiology Foundation; and the Heart Rhythm Society: endorsed by the International Society for Computerized Electrocardiology. *Circulation.* 2009;119(10):e251-261.

8. Kamel H, Soliman EZ, Heckbert SR, et al. P-wave morphology and the risk of incident ischemic stroke in the Multi-Ethnic Study of Atherosclerosis. *Stroke.* 2014;45(9):2786-2788.
9. Kamel H, O'Neal WT, Okin PM, Loehr LR, Alonso A, Soliman EZ. Electrocardiographic left atrial abnormality and stroke subtype in the Atherosclerosis Risk In Communities study. *Ann Neurol.* 2015;78(5):670-678.
10. Kamel H, Bartz TM, Longstreth WT, Jr., et al. Association between left atrial abnormality on ECG and vascular brain injury on MRI in the Cardiovascular Health Study. *Stroke.* 2015;46(3):711-716.
11. Kamel H, Hunter M, Moon YP, et al. Electrocardiographic left atrial abnormality and risk of stroke: Northern Manhattan Study. *Stroke.* 2015;46(11):3208-3212.
12. Benjamin EJ, D'Agostino RB, Belanger AJ, Wolf PA, Levy D. Left atrial size and the risk of stroke and death. The Framingham Heart Study. *Circulation.* 1995;92(4):835-841.
13. Barnes ME, Miyasaka Y, Seward JB, et al. Left atrial volume in the prediction of first ischemic stroke in an elderly cohort without atrial fibrillation. *Mayo Clin Proc.* 2004;79(8):1008-1014.
14. Di Tullio MR, Sacco RL, Sciacca RR, Homma S. Left atrial size and the risk of ischemic stroke in an ethnically mixed population. *Stroke.* 1999;30(10):2019-2024.
15. Yaghi S, Moon YP, Mora-McLaughlin C, et al. Left atrial enlargement and stroke recurrence: the northern Manhattan stroke study. *Stroke.* 2015;46(6):1488-1493.
16. Folsom AR, Nambi V, Bell EJ, et al. Troponin T, N-terminal pro-B-type natriuretic peptide, and incidence of stroke: the Atherosclerosis Risk In Communities study. *Stroke.* 2013;44(4):961-967.
17. Cushman M, Judd SE, Howard VJ, et al. N-terminal pro-B-type natriuretic peptide and stroke risk: the Reasons for Geographic and Racial Differences in Stroke Cohort. *Stroke.* 2014;45(6):1646-1650.
18. Kamel H, Okin PM, Elkind MS, Iadecola C. Atrial fibrillation and mechanisms of stroke: time for a new model. *Stroke.* 2016;47(3):895-900.
19. Sanna T, Diener HC, Passman RS, et al. Cryptogenic stroke and underlying atrial fibrillation. *N Engl J Med.* 2014;370(26):2478-2486.
20. Longstreth WT, Jr., Kronmal RA, Thompson JL, et al. Amino terminal pro-B-type natriuretic peptide, secondary stroke prevention, and choice of antithrombotic therapy. *Stroke.* 2013;44(3):714-719.
21. Connolly SJ, Eikelboom J, Joyner C, et al. Apixaban in patients with atrial fibrillation. *N Engl J Med.* 2011;364(9):806-817.
22. Ruff CT, Giugliano RP, Braunwald E, et al. Comparison of the efficacy and safety of new oral anticoagulants with warfarin in patients with atrial fibrillation: a meta-analysis of randomised trials. *Lancet.* 2014;383(9921):955-962.
23. Granger CB, Alexander JH, McMurray JJ, et al. Apixaban versus warfarin in patients with atrial fibrillation. *N Engl J Med.* 2011;365(11):981-992.
24. Kernan WN, Ovbiagele B, Black HR, et al. Guidelines for the prevention of stroke in patients with stroke and transient ischemic attack: a guideline for healthcare professionals from the American Heart Association/American Stroke Association. *Stroke.* 2014;45(7):2160-2236.
25. Dennis M. Embolic stroke of undetermined source: a therapeutic target? *Lancet Neurol.* 2014;13(4):344-346.
26. Freilinger TM, Schindler A, Schmidt C, et al. Prevalence of nonstenosing, complicated atherosclerotic plaques in cryptogenic stroke. *JACC Cardiovasc Imaging.* 2012;5(4):397-405.
27. Gupta A, Gialdini G, Lerario MP, et al. Magnetic resonance angiography detection of abnormal carotid artery plaque in patients with cryptogenic stroke. *J Am Heart Assoc.* 2015;4(6):e002012.

28. Gupta A, Gialdini G, Giambrone AE, et al. Association between nonstenosing carotid artery plaque on MR angiography and acute ischemic stroke. *JACC Cardiovasc Imaging.* 2016;9(10):1228-1229.
29. Chimowitz MI, Lynn MJ, Howlett-Smith H, et al. Comparison of warfarin and aspirin for symptomatic intracranial arterial stenosis. *N Engl J Med.* 2005;352(13):1305-1316.
30. Chimowitz MI, Lynn MJ, Derdeyn CP, et al. Stenting versus aggressive medical therapy for intracranial arterial stenosis. *N Engl J Med.* 2011;365(11):993-1003.
31. Amarenco P, Davis S, Jones EF, et al. Clopidogrel plus aspirin versus warfarin in patients with stroke and aortic arch plaques. *Stroke.* 2014;45(5):1248-1257.
32. Diener HC, Eikelboom J, Connolly SJ, et al. Apixaban versus aspirin in patients with atrial fibrillation and previous stroke or transient ischaemic attack: a predefined subgroup analysis from AVERROES, a randomised trial. *Lancet Neurol.* 2012;11(3):225-231.
33. Schulman S, Kearon C, Subcommittee on Control of Anticoagulation of the S, Standardization Committee of the International Society on T, Haemostasis. Definition of major bleeding in clinical investigations of antihemostatic medicinal products in non-surgical patients. *J Thromb Haemost.* 2005;3(4):692-694.
34. Meschia JF, Brott TG, Chukwudelunzu FE, et al. Verifying the stroke-free phenotype by structured telephone interview. *Stroke.* 2000;31(5):1076-1080.
35. Katzan IL, Fan Y, Uchino K, Griffith SD. The PROMIS physical function scale: A promising scale for use in patients with ischemic stroke. *Neurology.* 2016;86(19):1801-1807.
36. Jauch EC, Saver JL, Adams HP, Jr., et al. Guidelines for the early management of patients with acute ischemic stroke: a guideline for healthcare professionals from the American Heart Association/American Stroke Association. *Stroke.* 2013;44(3):870-947.
37. Armstrong MJ, Gronseth G, Anderson DC, et al. Summary of evidence-based guideline: periprocedural management of antithrombotic medications in patients with ischemic cerebrovascular disease: report of the Guideline Development Subcommittee of the American Academy of Neurology. *Neurology.* 2013;80(22):2065-2069.
38. Nam HS, Kim HC, Kim YD, et al. Long-term mortality in patients with stroke of undetermined etiology. *Stroke.* 2012;43(11):2948-2956.
39. Lan KKG, DeMets DL. Discrete sequential boundaries for clinical trials. *Biometrika.* 1983;70(3):659-663.

CHANGE LOG

Version	Date	Significant Revisions
1.1	7 July 2017	• Added the composite outcome of stroke of any type or death from any cause as a secondary efficacy outcome (4.2.2).<br>• Clarified inclusion and exclusion criteria (5.1 and 5.2).<br>• Clarified that patients not eligible for randomization may still be followed as part of ancillary studies (7.2.2).<br>• Revised schedule of assessments (7.2.6).<br>• Clarified procedures for study drug administration after a potential primary endpoint (7.9).<br>• Only SAEs will be recorded (8.1.1).<br>• Clarified SAE reporting procedures from NDMC to BMS (8.4.2).<br>• Certificate of confidentiality will be obtained (13.5).<br>• Clarified reporting of protocol deviations (14.3).<br>• Corrected typographical errors and made minor revisions throughout to improve clarity.
2	22 Sep 2017	• Updated safety reporting language to distinguish between SAEs, clinical outcome events, and AEs of special interest, rather than lumping them together in one category.<br>• Added clarification about study medication refills (7.2.3).
3.0	01 DEC 2017	• Modified time of randomization based on stroke severity. In general, randomization can occur as early as post-stroke day 3. However, randomization must be delayed until at least post-stroke day 14 for patients with severe strokes (initial NIHSS score ≥11), hemorrhagic transformation of the index stroke, or uncontrolled hypertension (7.2.2).<br>• Added information about buffer or number of extra pills provided. (6.14)<br>• Added route of medication "by mouth" (6.16)<br>• Clarified what patient should do for a missed dose of study medication ie when to take and when to skip. (6.16)<br>• Changed exclusion criteria to exclude patients with an indication for any antiplatelet therapy (5.2) and added requirement that study drug be stopped while any antiplatelet therapy is indicated after randomization (7.3.1). Clarified that all patients stopping study drug due to need for open-label anticoagulant or antiplatelet therapy will continued to be followed and analyzed according to the intention-to-treat principle (10.6.1).<br>• Follow-up visit window (7.2.3 and 7.2.6) window of time reduced from +/-14 days to +/-5 days to prevent subjects from running out of study medication and to keep them within the guidelines for follow-up visits based on time of randomization.

ARCADIA

AtRial Cardiopathy and Antithrombotic Drugs In prevention After cryptogenic stroke

Clinicaltrial.gov: NCT03192215

STATISTICAL ANALYSIS PLAN

SAP Version 1.4
Protocol Version 6
Date: 15 Feb 2023

Primary Trial Statistician, Blinded
Richard A. Kronmal, PhD
School of Public Health
University of Washington
Seattle, WA 98101

NDMC Trial Statistician, Unblinded
Christy Cassarly, PhD
Department of Public Health Sciences
Medical University of South Carolina
Charleston, SC 29425

TABLE OF CONTENTS

1 LIST OF ABBREVIATIONS 4
2 KEY ROLES 5
3 STATISTICAL ANALYSIS PLAN AND STATISTICAL REPORTS 5
4 BACKGROUND AND RATIONALE 6
5 SYNOPSIS OF THE STUDY 7
6 OUTCOME VARIABLES 7
6.1 PRIMARY EFFICACY OUTCOME 7
6.2 SECONDARY EFFICACY OUTCOMES 7
6.3 PRIMARY SAFETY OUTCOMES 7
6.4 SECONDARY SAFETY OUTCOMES 8
7 SAMPLE SIZE DETERMINATION FOR PRIMARY EFFICACY OBJECTIVE 8
7.1 SAMPLE SIZE ESTIMATION 8
7.2 REVISIONS TO THE GROUP SEQUENTIAL BOUNDARIES 8
8 DEFINITION OF TARGET POPULATION AND STUDY SAMPLES 9
8.1 TARGET POPULATION 9
8.2 INTENT-TO-TREAT SAMPLE 9
8.3 SAFETY-ANALYSIS SAMPLE 9
8.4 PER-PROTOCOL SAMPLE 9
9 GENERAL STATISTICAL CONSIDERATIONS 10
9.1 PARTICIPANT ACCOUNTABILITY 10
9.2 RANDOMIZATION 10
9.3 BLINDING 10
9.4 CENSORING PARTICIPANTS 12
9.4.1 PRIMARY OUTCOME 12
9.4.2 DEATH 12
9.4.3 CROSSOVER TO OPEN-LABEL DRUG 12
9.4.4 WITHDRAWN CONSENT AND LOSS TO FOLLOW-UP 12
9.4.5 SECONDARY AND SAFETY OUTCOMES 12
9.5 TREATMENT GROUP COMPARABILITY 12
9.6 SITE EFFECTS 13
9.7 PARTICIPANT ADHERENCE 13
10 EFFICACY ANALYSIS 13
10.1 PRIMARY EFFICACY ANALYSIS 13
10.1.1 PARAMETER DESCRIPTION 13
10.1.2 STATISTICAL HYPOTHESES 13
10.1.3 STATISTICAL MODEL 14
10.1.4 INTERIM ANALYSIS METHOD 14
10.1.5 FINAL ANALYSIS METHOD 15
10.1.6 ADDITIONAL ANALYSES OF THE PRIMARY EFFICACY OUTCOME 15
10.2 INTERACTION OF ATRIAL CARDIOPATHY SEVERITY AND TREATMENT EFFECT 16
10.2.1 TESTS OF INTERACTION 16
10.2.2 IDENTIFICATION OF HIGH-RISK SUBGROUPS 17
10.2.3 ADDITIONAL EXPLORATORY MODELING 17
10.2.4 STATISTICAL MODEL 17
10.3 SECONDARY EFFICACY ANALYSIS 18

10.3.1 PARAMETER DESCRIPTION 18
10.3.2 STATISTICAL HYPOTHESES 18
10.3.3 EXPLORATORY EFFICACY ANALYSES 18
10.3.4 EXPLORATORY ENDPOINTS 19
11 SAFETY ANALYSES 19
11.1 SAFETY MONITORING 19
11.2 DESCRIPTIVE SUMMARY OF EVENTS 19
11.3 ANALYSIS OF SAFETY OUTCOMES AT TRIAL COMPLETION 19
11.3.1 PRIMARY SAFETY ENDPOINTS 20
11.3.2 SECONDARY SAFETY ENDPOINT 20
11.4 ADJUDICATION OF SAFETY ENDPOINTS 20
12 SUBGROUP ANALYSES 20
13 QUALITY-OF-LIFE ANALYSES 20
13.1 QUALITY-OF-LIFE MEASURES 20
13.2 ANALYSES OF QUALITY-OF-LIFE MEASURES 20
14 COST-EFFECTIVENESS ANALYSIS 21
15 UPDATE OF TRIAL OPERATING PARAMETERS 21
16 CHANGE LOG 22
17 REFERENCES 23

1 LIST OF ABBREVIATIONS

AE	Adverse event
AF	Atrial fibrillation
AHA/ASA	American Heart Association/American Stroke Association
ARCADIA	AtRial Cardiopathy and Antithrombotic Drugs In prevention After cryptogenic stroke
CRF	Case report form
CI	Confidence interval
DCU	Data Coordination Unit
DCR	Data clarification request
DSMB	Data and safety monitoring board
ECG	Electrocardiogram
ESUS	Embolic stroke of undetermined source
HR	Hazard ratio
ITT	Intention to treat
MOP	Manual of procedures
NCC	NIH StrokeNet National Coordinating Center
NDMC	NIH StrokeNet National Data Management Center
NIH	National Institutes of Health
NIHSS	National Institutes of Health Stroke Scale
NINDS	National Institute of Neurological Disorders and Stroke
NOAC	Non-vitamin K antagonist oral anticoagulant drug
NT-proBNP	Amino terminal pro-B-type natriuretic peptide
PI	Principal Investigator
PROMIS	Patient-Reported Outcomes Measurement Information System
$PTFV_1$	P-wave terminal force in ECG lead V_1
QVSFS	Questionnaire for Verification of Stroke-Free Status
RCC	Regional Coordinating Center
SAE	Serious adverse event
SD	Standard deviation
SOP	Standard operating procedure

2 KEY ROLES

Primary Trial Statistician, Blinded
Richard A. Kronmal, PhD
School of Public Health
University of Washington
Seattle, WA 98101

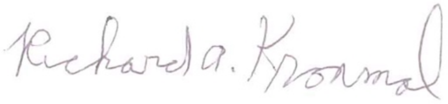

NDMC Trial Statistician, Unblinded
Caitlyn Meinzer, PhD
Department of Public Health Science
Medical University of South Carolina,
Charleston, SC, 29425

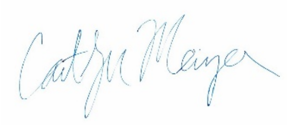

Contact PI
Mitchell S.V. Elkind, MD, MS

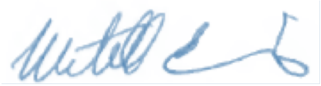

Protocol PI
Hooman Kamel, MD

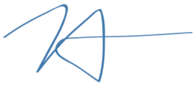

3 STATISTICAL ANALYSIS PLAN AND STATISTICAL REPORTS

This document provides the details of statistical analyses planned for the ARCADIA trial, including interim analyses for efficacy and futility. In addition, it discusses the statistical issues relevant to these analyses (e.g., sample data to be used, missing data, adjustments for multiplicity, etc.).

The DCU generates two statistical reports: an open report to be distributed to the ARCADIA Executive Committee and the DSMB, and a closed report to be distributed only to the DSMB. The timing of these reports is determined in consultation with the DSMB. Reports will be sent from the DCU to the NINDS liaison two weeks in advance of the scheduled meeting.

Each report provides cumulative summary statistics on enrollment; participant status in the study (e.g., number who completed each study visit assessment); baseline characteristics; protocol violations; data management/quality information (e.g., timeliness and completeness of data entry by the clinical sites; number of DCRs generated and resolved); and safety data, including SAEs by AE code, severity, expectedness (anticipated/unanticipated), and relatedness to the study medication. Aggregate rates of SAEs including atrial fibrillation/flutter and excluding clinical outcomes (stroke [ischemic, hemorrhagic, or undetermined type], symptomatic hemorrhagic transformation of ischemic stroke, intracranial hemorrhage excluding either hemorrhagic stroke or symptomatic hemorrhagic transformation of ischemic stroke, transient ischemic attack, major hemorrhage excluding intracranial hemorrhage, minor hemorrhage, myocardial infarction, systemic embolism, symptomatic deep venous thrombosis, and symptomatic pulmonary embolism) are provided in the open report; data on all other endpoints are restricted to the closed report. The statistics are provided for the overall study as well as by site or RCC, where appropriate, in the open report. For the closed report only, the statistics are also provided by treatment group (A vs B). If a report coincides in timing with a planned interim analysis, the analysis results are appended to the report.

The DCU also generates a blinded quarterly report to be distributed to the ARCADIA Executive Committee which contains updated aggregate data on enrollment, baseline, safety information, and loss to follow-up and crossovers. In addition, this report contains a table that shows what proportion of consented patients were randomized and on which atrial cardiopathy marker(s) these participants were deemed eligible, including summary statistics of the atrial cardiopathy marker values.

4 BACKGROUND AND RATIONALE

In one-third of ischemic strokes, a specific cause cannot be identified. Many of these cryptogenic strokes appear to arise from a distant embolic source. Recent evidence suggests that some cryptogenic strokes may arise from left atrial thromboembolism that goes unrecognized because it has not manifested with atrial fibrillation/flutter (AF). These data suggest that an underlying atrial cardiopathy may provide the substrate for thrombus formation and embolization even in the absence of AF.

In parallel with these new insights about cryptogenic stroke, new therapeutic options for stroke prevention have become available. Non-vitamin K antagonist oral anticoagulant (NOAC) drugs such as apixaban may be more effective than aspirin for treatment of cryptogenic stroke. A benefit is especially likely in cryptogenic stroke patients with atrial cardiopathy because of parallels with AF and because an analysis of data from the WARSS/APASS studies suggests that cryptogenic stroke patients with one marker of atrial cardiopathy (elevated NT-proBNP) and no obvious AF benefit from anticoagulant therapy.[1] Apixaban is a particularly attractive choice because it has a low bleeding risk,[2,3] lowers mortality more than warfarin in patients with AF,[4] and is the only NOAC drug with a Class 1A recommendation in recent AHA/ASA guidelines.[5]

The ARCADIA trial is designed to address several important knowledge gaps. First, it will advance knowledge about stroke pathophysiology by assessing whether atrial cardiopathy is a valid therapeutic target, which may set the stage for a primary prevention trial. Second, this trial will advance knowledge about optimal secondary stroke prevention therapy. One industry-sponsored trial (NAVIGATE-ESUS) that compared rivaroxaban to aspirin in patients with cryptogenic stroke was stopped early for futility. Another industry-sponsored trial (RESPECT-ESUS) is comparing dabigatran versus aspirin in patients with cryptogenic stroke. By including all cryptogenic stroke patients and by including those with up to 6 minutes of AF per day, these trials have mixed patients with heterogeneous stroke mechanisms. These trials may thus fail to show an overall benefit (as in NAVIGATE-ESUS), or may show an overall benefit driven mostly by patients with known AF and atrial cardiopathy, resulting in an overly broad indication for anticoagulant therapy. In the first instance, the ARCADIA trial may ensure that a valuable treatment in a specifically targeted subgroup will not be prematurely abandoned. In the second instance, the results of the ARCADIA trial would provide a compelling rationale to perform subgroup analyses of the industry-sponsored trials and conduct future trials to determine the risks and benefits of anticoagulant therapy across biologically distinct subgroups of stroke patients. Given the expense and risks of bleeding associated with anticoagulant drugs, it is imperative to define as precisely as possible the groups of stroke patients who would and would not benefit from their use. Therefore, the approach taken by the ARCADIA trial comports well with the general move toward precision medicine.

5 SYNOPSIS OF THE STUDY

ARCADIA is a multicenter, biomarker-driven, randomized, double-blind, active controlled Phase III clinical trial of 1,100 participants, conducted at up to 200 enrolling sites. Participants will be followed for a minimum of 1.5 years. Patients who have evidence of atrial cardiopathy and a recent stroke of undetermined source by current criteria will be randomly assigned to apixaban or aspirin (active control therapy) in a 1:1 ratio. Atrial cardiopathy will be defined as one or more of the following biomarkers: P-wave terminal force in electrocardiogram lead V1 >5,000 µV*ms, left atrial size index ≥3.0 cm/m2 on echocardiogram, and serum amino terminal pro-B-type natriuretic peptide >250 pg/mL. Standard heart-rhythm monitoring will be performed before enrollment to exclude as thoroughly as possible those patients with atrial fibrillation. Therapy must be initiated within 3-180 days of enrolling stroke. The primary efficacy endpoint is time to recurrent stroke (ITT analysis). Secondary efficacy endpoints are time to (1) recurrent stroke or death and (2) recurrent ischemic stroke or systemic embolism. There will be a test of the interaction of the atrial cardiopathy selection variables with treatment arm for the primary outcome. The primary safety outcomes are: (1) symptomatic intracranial hemorrhage (including symptomatic hemorrhagic transformation of an ischemic stroke) and (2) major hemorrhage other than intracranial hemorrhage. Additional safety endpoints include all-cause mortality.

6 OUTCOME VARIABLES

6.1 PRIMARY EFFICACY OUTCOME

The primary efficacy outcome is recurrent stroke of any type (ischemic, hemorrhagic, or of undetermined type). Possible stroke events will be centrally adjudicated, and the primary outcome measure will be defined as the centrally adjudicated stroke event, NOT the site determined event.

If a site identifies an event as a possible primary efficacy outcome, the participant will stop study medication. If the adjudication committee determines the event meets the primary efficacy definition, the participant will stop participation in the study, including stopping study drug; otherwise they may stop, continue, or resume study drug at the discretion of the treating physician.

6.2 SECONDARY EFFICACY OUTCOMES

The secondary efficacy outcomes are: (A) composite of recurrent ischemic stroke or systemic embolism, and (B) composite of recurrent stroke of any type or death from any cause. Possible events will be centrally adjudicated, and the outcome measure will be defined as the centrally adjudicated event, NOT the site determined event. Note that recurrent ischemic stroke is a subset of the primary outcome definition and thus the participant would stop participation in the study, including stopping study drug. In the case of systemic embolism, the participant may continue in the trial until the end of follow-up or occurrence of the primary endpoint.

6.3 PRIMARY SAFETY OUTCOMES

The primary safety outcomes are: (A) symptomatic intracranial hemorrhage (including symptomatic hemorrhagic transformation of an ischemic stroke), and (B) major hemorrhage other than intracranial

hemorrhage. Symptomatic intracranial hemorrhage is defined as any extravascular blood within the cranium, including subdural and epidural blood, associated with and identified as the predominant cause of new neurologic symptoms, including headache, or leading to death. Major hemorrhage other than intracranial hemorrhage is defined as clinically overt bleeding accompanied by a ≥2 g/dL decrease in the hemoglobin level during a 24-hour period, transfusion of ≥2 units of whole blood or red cells, involvement of a critical non-intracranial site (intraspinal, intraocular, pericardial, intraarticular, intramuscular with compartment syndrome, or retroperitoneal), or death.

Note that symptomatic intracranial hemorrhage may not meet the primary outcome definition of stroke, in which case the participant is eligible to continue in the trial; this is true for other safety outcomes as well.

6.4 SECONDARY SAFETY OUTCOMES

The secondary safety endpoint is all-cause mortality. Note that death is included as part of a composite secondary efficacy endpoint.

7 SAMPLE SIZE DETERMINATION FOR PRIMARY EFFICACY OBJECTIVE

7.1 SAMPLE SIZE ESTIMATION

The primary outcome variable is the time to recurrent stroke of any type. Preliminary data suggest a 7% annual risk of recurrent stroke of any type in aspirin-treated patients, with a clinically relevant relative risk reduction of 40% (hazard ratio of 0.6, hazard rate of 4.2%) for patients treated with apixaban. Based on preliminary feasibility data and 120 sites, ARCADIA plans to recruit 1,100 participants during a 30-month uniform accrual period and follow these participants to the end of the study (i.e., 18-month minimum follow-up period). In addition, it is anticipated that there will be a 3% annual rate of crossover from blinded aspirin to open-label anticoagulation due to AF detection, and a 3% annual rate of crossover from blinded apixaban to open-label antiplatelet therapy due to bleeding or other adverse events. Finally, it is estimated that there will be a 5% annual rate of loss to follow-up or death in each group.

Given the assumptions above, enrollment of 1,100 participants (approximately 150 recurrent stroke events) will provide 80% power to demonstrate a statistically significant difference in the hazard for recurrent stroke with apixaban compared to aspirin at an alpha level of 0.05 while allowing for one interim look for efficacy (or harm) and futility after 1/2 of expected events have occurred (75 events). To maintain the power as planned and the overall alpha of the study at 0.05, an O'Brien-Fleming type Lan-DeMets error spending function will be used. The sample size was calculated using the "Design: Survival Endpoint: Two-Sample Test – Parallel Design – Logrank" program in EAST® v6 software (Cytel Corporation) (Lan and DeMets, 1983).

7.2 REVISIONS TO THE GROUP SEQUENTIAL BOUNDARIES

It is possible that the information fraction at the interim analysis may be incorrect due to incorrect estimation of the total number of events in the trial. However, given this trial's extremely conservative interim analysis boundaries, it is unlikely that such misestimation would substantively affect the final

test statistic. Furthermore, there is inherent difficulty in accurately estimating the total number of events even at the time of the interim analysis. Therefore, there will be no revisions to the group sequential boundaries.

8 DEFINITION OF TARGET POPULATION AND STUDY SAMPLES

8.1 TARGET POPULATION

The population that will be randomized in the ARCADIA trial comprises patients 45 years of age and older who have a clinical diagnosis of embolic stroke of undetermined source (ESUS) within the prior 180 days, a modified Rankin Scale score ≤4 at the time of screening, and at least one of the following criteria for atrial cardiopathy:

- [ECG]: PTFV1 >5,000 µV*ms on 12-lead ECG.
- [ECHO]: Left atrial size index ≥3 cm/m^2 on echocardiogram.
- [NT-proBNP]: Serum NT-proBNP >250 pg/mL.

8.2 INTENT-TO-TREAT SAMPLE

Under the ITT principle, the evaluable sample includes all participants who are randomized. Each participant will be analyzed according to the treatment group to which they were randomly assigned at the time of randomization.

8.3 SAFETY-ANALYSIS SAMPLE

The safety sample includes all participants who are randomized and receive at least one dose of study medication. The safety sample will include all safety outcomes from randomization until 30 days after permanent discontinuation of study drug. The primary safety analysis will define treatment arm as the treatment to which a participant was randomized. Additional sensitivity analyses will define a time-dependent treatment covariate, and possibly a lagged time-dependent treatment covariate.

8.4 PER-PROTOCOL SAMPLE

In addition to the defined ITT analysis sample, a per-protocol sample is defined as a subset of the ITT sample. This sample will be used for sensitivity analyses of the primary and secondary efficacy outcomes. The per-protocol sample will include all randomized participants who do not have the following protocol deviations:

- Eligibility violation.
- Treatment never started.

For these sensitivity analyses, treatment will first be defined as randomized, but a time-dependent treatment covariate with concurrent treatment effects may be considered.

9 GENERAL STATISTICAL CONSIDERATIONS

9.1 PARTICIPANT ACCOUNTABILITY

A flowchart will be created to present a summary of participants' status. This flowchart will first list the number of patients who were eligible and consented to be screened for the biomarkers. Of consented patients, the chart will then delineate those who did not meet criteria for any of the three biomarkers, those who were positive for at least one biomarker but no longer eligible at the time of randomization or declined randomization or were not randomized for another reason, and those randomized to a treatment group. Then, within each treatment group, it will list the number of participants who were lost to follow-up, the number of participants who withdrew, and the number of participants who were included in the primary analysis.

9.2 RANDOMIZATION

Eligible participants will be allocated in a 1:1 ratio to apixaban or aspirin using a randomization method that controls the maximum tolerated imbalance within each RCC. Implementation details are provided in the randomization plan and validation documentation.

A web-based central randomization system will be developed by the NDMC and installed on the WebDCU™ ARCADIA study website. The randomization algorithm and its implementation will be fully detailed in the ARCADIA Randomization Plan and the Randomization Validation documents. These documents will be developed prior to enrollment initiation and stored in a secure location at the NDMC. The documents will be archived with the study database at the end of the trial.

The participant randomization system is fully integrated into the ARCADIA study database, and presented as a special CRF in the electronic data capture module. Data edit permission for the randomization CRF is centrally managed based on user groups. Site study team members are limited to viewing and editing data only for participants in their own site. Site enrollment status, participant eligibility, and site study drug availability are verified prior to the performance of participant randomization in order to protect data integrity. The participant randomization system checks the drug availability at both the site level and the individual participant level and triggers site study drug shipment and participant drug resupply requests accordingly.

Participant treatment assignment information is masked by using the Study Drug ID, which is a globally unique no-sequence 6-digit number placed on each study drug kit. Access to treatment assignment information is prohibited for all blinded investigators. Authorized emergency unblinding for medical safety purposes is provided through a medical emergency hotline with one of the trial PIs responding to the treating physician. All cases of unblinding are recorded in the study database.

9.3 BLINDING

The study is conducted in a double-blinded manner. Study drug administration, clinical care, and outcomes assessments are made by the clinical site staff blinded to the treatment assignment. Participants will not be told of their actual treatment assignment until the trial is complete, at which point assignments are available upon request, or during the trial in the event of a medical emergency

that requires unblinding. The Executive Committee and the MSM are also blinded to the treatment assignment.

The NDMC biostatistician and statistical programmer will be partially unblinded; they know the participants' group classification (A or B), but not whether A is apixaban or aspirin. During the course of the trial it may be necessary for them to become unblinded if the DSMB requests. The clinical adjudicators will attempt to remain blinded, but information in the narrative may on rare occasions unblind specific cases. The Executive Committee members are completely blinded.

The DCU's unblinded staff generates: (1) a hard copy list (obtained from the WebDCU™) of study drug kit numbers and their corresponding study drug (apixaban or aspirin) as well as the lot numbers and expiration dates; and (2) two identical sealed envelopes that contain identification of treatment codes (e.g., A = apixaban, B = aspirin). Prior to initiation of the trial, one envelope is given to the NINDS liaison to the DSMB. Another is maintained in a locked file cabinet in a limited access central file room along with the hard copy list described in (1).

The central pharmacy personnel at the StrokeNet NCC at the University of Cincinnati are completely unblinded. They have access to the WebDCU™ to match the study drug (apixaban or aspirin) to its corresponding study drug kit number.

The DSMB (as well as the NINDS liaison to the DSMB) is partially unblinded. However, if it so wishes, it may be completely unblinded at any time during the trial. One of the sealed envelopes described in (2) above is given to the NINDS liaison to the DSMB prior to initiation of the trial. If the DSMB wishes to be unblinded on a particular participant only, the NINDS liaison to the DSMB can email the request to the NDMC biostatistician.

In case of a medical emergency for a study participant who requires unblinding, clinical staff can become unblinded only to that participant's treatment by calling the emergency hotline. Clinical staff would then request permission for unblinding to occur and provide a brief clinical summary to one of the responding trial PIs. The PI would perform unblinding via the WebDCU™ ARCADIA study website and inform the clinical staff. Unblinding should only occur if a compelling clinical reason arises, such as active major bleeding or a pending decision on whether to administer intravenous thrombolysis for acute ischemic stroke. Participants who undergo unblinding will stop study drug but continue to be followed as part of the study and will be analyzed according to the intention-to-treat principle.

Details such as how kits will be labeled and how the study drug is blinded are provided in the Study Drug Plan.

9.4 CENSORING PARTICIPANTS

9.4.1 PRIMARY OUTCOME

Participants will be followed until the end of the trial follow-up period, which will be 18 months after enrollment of the last participant. Due to regulatory reasons, participants will have a final telephone visit 30 days after the trial follow-up period to assess SAEs related to discontinuation of study drug, but this 30-day period will not contribute to participants' time-at-risk for the purposes of analysis. Under the ITT principle, all participants who are randomized are included in the analysis. Participants will be censored at the date of their last follow-up visit in cases of loss to follow-up or withdrawn consent, or at the date of death in cases of death.

9.4.2 DEATH

The primary analysis codes death as a censoring event. Sensitivity analyses will be conducted where death is treated as a competing risk.

9.4.3 CROSSOVER TO OPEN-LABEL DRUG

If a participant is known to have permanently crossed over onto open-label drug (e.g., an anticoagulant in the case of AF diagnosis, antiplatelet drug or no antithrombotic therapy in the case of bleeding events on apixaban, antiplatelet therapy for a new coronary event), the participant will continue to be followed and analyzed in the treatment group they are assigned. A sensitivity analysis using a time-dependent treatment effect, as well as an analysis accounting for pill count compliance data, will be considered.

9.4.4 WITHDRAWN CONSENT AND LOSS TO FOLLOW-UP

Using recommended methods,[6] sensitivity analyses will be conducted to assess the effect of any censoring that occurs due to a participant being lost to follow-up or withdrawing consent. Based on other large stroke prevention trials, we anticipate no more than a 4% lost to follow up rate. At the time of each planned analysis (interim and final), the unblinded statistician will report the amount of missing primary outcome data.

9.4.5 SECONDARY AND SAFETY OUTCOMES

In cases where observation of the primary outcome precludes observation of secondary or safety outcomes, the primary analysis will treat the participant as censored. Sensitivity analyses will explore alternative modeling strategies, in particular competing risk, Markov models, or joint modeling approaches.

9.5 TREATMENT GROUP COMPARABILITY

A description of the baseline characteristics of trial participants will be presented by treatment group. Dichotomous variables will be summarized as number (%). Percentages will be calculated based on the number of participants with available data for that variable. Continuous variables will be summarized by the mean and SD. In the case of variables with missing values, the denominator will be stated in the

summary table or in a footnote to the summary table. The baseline characteristics of interest include age, sex, race, time from qualifying stroke, CHA_2DS_2-VASc score, weight, and the value of each of the biomarker inclusion criteria. Statistical significance of covariates will not be assessed. In addition, all of the above covariates, regardless of imbalances between groups, will be included as covariates in multivariable regression in a secondary analysis of the primary aim.

9.6 SITE EFFECTS

Several procedures have been incorporated into the study design (i.e., procedure manual, training and certification programs, protocol violation monitoring, blinding) to reduce trial site effects; however, possible effects will not be ignored for this trial. The distribution of demographics will be examined by RCC using means (SD) and proportions (95% CIs), and RCC and RCC*treatment interaction terms will be included as a random effect in a secondary analysis of the primary outcome.

9.7 PARTICIPANT ADHERENCE

Participants will be requested to bring in their study pill bottles at study visits for pill counts. For each visit interval, adherence by pill count will be measured as (# pills supplied - # remaining pills)/# pills supplied. Adherence will be classified as low (<60%), medium (60-79%), or high (≥80%). A sensitivity analysis of the primary endpoint will use a time-dependent compliance indicator to assess the impact of non-compliance on hazard of stroke. Note that participants are supplied with a buffer of 10 pills per bottle. The calculation listed above will adjust for the number of days between visits (i.e., a participant with 100% compliance and 90 days between study visits will be listed as 100% compliant).

10 EFFICACY ANALYSIS

10.1 PRIMARY EFFICACY ANALYSIS

10.1.1 PARAMETER DESCRIPTION

The primary efficacy analysis will use the ITT sample to assess time to recurrent stroke of any type. Censoring of participants is described in Section 0.

10.1.2 STATISTICAL HYPOTHESES

The null hypothesis (H_0) is that the hazard ratio comparing apixaban versus aspirin for the primary endpoint will be 1. The alternative (H_A) is that the hazard ratio is not 1. That is,

$$H_0: h_{apix}(t) = h_{asp}(t) \quad \text{versus} \quad H_A: h_{apix}(t) \neq h_{asp}(t)$$

Where $h_{apix}(t)$ and $h_{asp}(t)$ are the hazard rates at time t for the apixaban and aspirin groups, respectively. The minimum effect size of clinical interest is a relative HR of 0.6. The estimated yearly event rate in the placebo group is assumed to be 7%.

10.1.3 STATISTICAL MODEL

$$Z = \frac{\sum_{i=1}^{D} W(t_i)\left[d_{i1} - Y_{i1}\left(\frac{d_i}{Y_i}\right)\right]}{\sqrt{\sum_{i=1}^{D} W(t_i)^2 \frac{Y_{i1}}{Y_i}\left(1-\frac{Y_{i1}}{Y_i}\right)\left(\frac{Y_i - d_i}{Y_i - 1}\right) d_i}}$$

Which has a standard normal distribution for large samples when H_0 is true. Using this statistic, an α level test of the alternative hypothesis $H_A : \mathrm{h}_1(t) \neq h_2(t)$, for some $t \neq \tau$, is rejected when $|Z| > Z_{\alpha/2}$. Let W(t)=1 for all t, which represents the log-rank test.

**Efficacy Outcome**	**Analysis Method to Compare Apixaban and Aspirin Groups (SAS® v9 Procedure)**
Log-Rank Test of Survival	PROC PHREG

To test the null hypothesis, we will use the log-rank test.
In the above model, an event (Status) and event time (Time) are defined as follows:

First Event	Status	Time
Recurrent stroke of any type	1	Time of first stroke of any type
Systemic embolism	N/A (continue to follow)	
Death	0	Time of death
Diagnosis of AF	N/A (continue to follow)	
Study drug stopped	N/A (continue to follow)	
Loss to follow-up or consent withdrawn	0	Last known contact
Study team requires unblinding and recurrent stroke of any type has not yet occurred	N/A (continue to follow)	

10.1.4 INTERIM ANALYSIS METHOD

The study is designed using one interim look for both efficacy and futility for the primary outcome, and one final look, for a total of two planned analyses of the primary outcome. The interim analysis plan uses the error spending function method with O'Brien-Fleming type stopping guidelines. The error spending function distributes the type I and II error rates across the interim monitoring points giving the flexibility of changing the intervals of monitoring while still preserving the overall type I and II error rates. The O'Brien-Fleming type boundary is considered conservative as its boundaries make it difficult to terminate a study early by requiring extreme early evidence of efficacy or futility. It spends smaller amounts of alpha at the first look and gradually increases the spending as more information is acquired. The trial may be stopped for overwhelming efficacy of one treatment group over the other or for futility at the planned interim analyses if the test statistic crosses the respective boundaries.

The prespecified plan is to conduct the first interim analysis after 75 cumulative adjudicated primary outcome events (estimated information fraction of ½), or approximately 800 participants have been

randomized. Assuming an accrual rate of 36.7 participants per month, it is anticipated that the first look will occur roughly 3.5 years from the start of enrollment. The interval may be altered if requested by the DSMB. The stopping boundaries are defined using the Lans-DeMets O'Brien-Fleming type spending functions (Lan and DeMets, 1983). The futility boundaries are derived as non-binding, meaning that if a futility boundary is crossed it can be overruled without inflation of the type I error rate. If the crossing of an efficacy boundary is overruled, then this decision can impact the type II error rate but not the type I error rate. EAST® v6 software (Cytel Corporation) was used for the boundary calculations. The following table lists the test statistics at a particular look. The NDMC will be responsible for conducting these analyses and compiling the reports for the DSMB. Since several factors need to be taken into consideration before stopping a study, safety and study progress will also be taken into consideration by the DSMB and Executive Committee when deciding whether to stop the study if an efficacy or futility boundary is crossed.

Table. Nominal Critical Values and Alpha Levels for One Interim Analysis for Overwhelming Efficacy and Futility (O'Brien-Fleming, two-sided α = 0.05)

	Exp. # events under H_0	Cum. α	Efficacy Boundary		Cum. β	Futility Boundary	
			Z Scale	HR		Z Scale	HR
Interim Analysis	75	0.003	|Z|>2.963	HR>2.123 -OR- HR<0.471	0.04	|Z|<0.356	HR>0.914 -AND- HR<1.095
Final Analysis	150	0.05	|Z|>1.969	HR>1.424 -or- HR<0.702	0.20		

10.1.5 FINAL ANALYSIS METHOD

The primary outcome analysis of the time to stroke of any type will use a log-rank test statistic to assess whether there is a difference in the hazard comparing apixaban to aspirin. Outcome differences will be analyzed under the ITT principle, so all randomized participants will be included in the primary analysis sample. To assess efficacy, the treatment groups will be compared with respect to the hazard of experiencing a primary outcome event. A log-rank test will be performed to compare the treatment group proportions using a cumulative two-tailed significance level of 0.05. An unadjusted HR will be reported with two-sided 95% CIs. At the final analysis, the null hypothesis will be rejected if the HR is greater than 1.424 or less than 0.702; we will fail to reject the null hypothesis at the 0.05 level of significance otherwise.

10.1.6 ADDITIONAL ANALYSES OF THE PRIMARY EFFICACY OUTCOME

10.1.6.1 Adjusting for Key Covariates

In secondary analyses of the primary outcome, we will adjust for the prognostic variables listed in Section 9.5. A Cox proportional hazards model will be used, after the verification of variable and model assumptions and goodness-of-fit assessments accompanying each analysis. Schoenfeld residuals will be used to assess the assumption of proportional hazards, and if significant deviations are found, we will

analyze treatment effect according to time period. These secondary analyses will be reported in the paper reporting the primary results of the trial.

10.1.6.2 Accounting for Crossover to Open-Label Therapy

In a secondary analysis, we will censor participants at the time of AF detection, when they are switched to open-label anticoagulation. This analysis will be used to explore the utility of anticoagulation in participants with atrial cardiopathy before they have manifested AF and therefore remain “cryptogenic” based on the prevailing clinical paradigm. Alongside this analysis, we will also perform an alternative analysis treating AF as a competing risk rather than a censoring point, given very recent theoretical data suggesting that apixaban may reduce the risk of AF itself, not just stroke. Lastly, we will perform another alternative analysis setting the outcome as stroke of any type occurring before the time of AF detection.

10.1.6.3 Accounting for Unblinding

In a secondary analysis, we will censor participants at the time of unblinding, when they are switched to open-label antithrombotic therapy per the discretion of the treating physician.

10.2 INTERACTION OF ATRIAL CARDIOPATHY SEVERITY AND TREATMENT EFFECT

This secondary analysis will explore the relationship between the severity of atrial cardiopathy and the relative efficacy of apixaban over aspirin. The objective of this analysis is two-fold. First, it will be used to assess the degree to which the severity of atrial cardiopathy modifies the efficacy of anticoagulation. Second, it will be used, regardless of whether or not apixaban is superior to aspirin in the overall trial population, to identify high-risk subgroups of participants with severe atrial cardiopathy who substantially benefit from apixaban.

10.2.1 TESTS OF INTERACTION

A Cox regression model will be used to assess interactions between each of the three atrial cardiopathy biomarkers and treatment status. The dependent variable will be recurrent stroke of any type. Independent variables will be a categorical variable for treatment status (apixaban versus aspirin) and three continuous variables representing each atrial cardiopathy biomarker. The atrial cardiopathy biomarkers will be appropriately transformed if necessary (e.g., log-NT-proBNP). Two-way interaction terms will be included between the treatment status variable and each atrial cardiopathy biomarker variable.

The z statistics for the individual interaction hazard ratios will be used to test the significance of the interaction terms. Each of the tests for the three biomarker-treatment interactions will be considered statistically significant if its individual P value is <0.016. This conservative P value is chosen to account for the multiple hypothesis testing in this exploratory analysis. Given the hypothesis test for the primary endpoint, the tests of the three interactions will exceed an experiment-wide alpha threshold of 0.05, but the alpha threshold of 0.016 for each interaction test will provide a conservative approach to the evaluation of interactions. Given that these tests of interaction are exploratory analyses, formal power calculations are not provided.

10.2.2 IDENTIFICATION OF HIGH-RISK SUBGROUPS

If one or more of the interactions are statistically significant, a prediction score computed from the Cox model will be used to predict the degree of benefit of apixaban over aspirin in a given participant based on the severity of atrial cardiopathy. Such a risk score will be important regardless of the main findings of this trial. If Aim 1 is negative (no benefit of apixaban versus aspirin in the overall trial cohort), the prediction score will be used to identify subgroups of patients with severe atrial cardiopathy who may still benefit from apixaban despite the negative findings in the overall trial population. This would inform follow-up studies of antithrombotic therapy in patients with cryptogenic stroke. If Aim 1 is positive (apixaban proves superior to aspirin in the overall trial cohort), the prediction score will be used to assess whether absolute stroke risk reductions in the highest-risk atrial cardiopathy subgroups are large enough to justify a primary prevention trial.

10.2.3 ADDITIONAL EXPLORATORY MODELING

Since the effect of the atrial cardiopathy biomarkers or their interactions with the treatment assignment may be nonlinear, analyses will also be carried out using the generalized additive model in the Cox regression to allow for nonlinearities in the interactions of the biomarkers with treatment status. For this purpose, the approach and methodology described by Royston (Royston, 2000; Royston and Sauerbrei, 2004) and the associated functions available in Stata (MVRS and FP2) will be used. If there is evidence of important nonlinearities in the biomarker-treatment interactions, the risk score derived from this more complex model will be evaluated and compared to the linear model described in in 11.2.2. Even if there is no effect modification of the atrial cardiopathy variables on the efficacy of apixaban versus aspirin, we will evaluate these markers as predictors of stroke risk in the full sample of participants in whom we have measured these markers and for whom we have follow-up for stroke events. We will construct a prediction risk score which will also include the other prognostic variables defined in Section 9.5. Such a risk score will potentially be valuable for identifying subgroups of patients with atrial cardiopathy in whom the absolute benefit of apixaban is high enough to justify a primary prevention trial.

10.2.4 STATISTICAL MODEL

The following is the right hand side of the full Cox proportional hazards model:

$$= \beta_0 + \beta_1 I_{Tx} + \beta_2 X_{ECG} + \beta_3 X_{Echo} + \beta_4 X_{BNP} + \beta_5 I_{Tx} * X_{ECG} + \beta_6 I_{Tx} * X_{Echo} + \beta_7 I_{Tx} * X_{BNP} + \left[\sum \beta_j X_j \right]$$

Where I_{TX} is an indicator with the value of 1 if the participant is on apixaban or 0 for aspirin, $X_{ECG, ECHO, BNP}$ are the continuous values of the three biomarkers, and X_j are the values of other prognostic variables.

From the full model, standard backward elimination regression modeling procedures will be used to identify the final model. Specifically, we will first test the interaction term:

H_0: $\beta_5 = 0$; H_0: $\beta_6 = 0$; H_0: $\beta_7 = 0$

If it is not statistically significant at $\alpha = 0.016$, these terms will be dropped from the model and the covariates β_j will be tested in a stepwise approach. For the risk-based modeling, the fitted estimates of the final model will be reported given a participant's covariates, e.g. $\left\{ X_{ECG}, X_{Echo}, X_{BNP}, X_j \right\}$, under apixaban and under aspirin, as well as the hazard ratio.

10.3 SECONDARY EFFICACY ANALYSIS

10.3.1 PARAMETER DESCRIPTION

The secondary efficacy outcomes are: (A) composite of recurrent ischemic stroke or systemic embolism, and (B) composite of recurrent stroke of any type or death from any cause. Each of these two outcomes will be analyzed separately using the ITT sample (Section 8.2).

10.3.2 STATISTICAL HYPOTHESES

The set of statistical hypotheses can be stated in terms of hazard functions as:

H_0: $h_{apix}(t)=h_{asp}(t)$ versus H_A: $h_{apix}(t) \neq h_{asp}(t)$

Where $h_{apix}(t)$ and $h_{asp}(t)$ are the hazard rates for the apixaban and aspirin groups, respectively.

STATISTICAL MODEL

Each of the secondary efficacy outcomes will be analyzed using the same model as described for the primary efficacy analysis (Section 10.1), but the status and time of events will be defined as follows:

Outcome: Composite of recurrent ischemic stroke or systemic embolism		
First Event	Status	Time
Recurrent ischemic stroke	1	Time of first ischemic stroke
Recurrent stroke of any type	0	Time of first stroke of any type
Systemic embolism	1	Time of first systemic embolism
Death	0	Time of death
Diagnosis of AF	N/A (continue to follow)	
Study drug stopped	N/A (continue to follow)	
Loss to follow-up or consent withdrawn	0	Last known contact

Outcome: Composite of recurrent stroke of any type or death from any cause		
First Event	Status	Time
Recurrent stroke of any type	1	Time of first stroke of any type
Systemic embolism	N/A (continue to follow)	
Death	1	Time of death
Diagnosis of AF	N/A (continue to follow)	
Study drug stopped	N/A (continue to follow)	
Loss to follow-up or consent withdrawn	0	Last known contact

10.3.3 EXPLORATORY EFFICACY ANALYSES

In a tertiary analysis, we will assess the effect of apixaban on the subset of ischemic stroke comprising only cardioembolic or cryptogenic stroke, as determined by Causative Classification of Stroke determinations performed by site investigators.

10.3.4 EXPLORATORY ENDPOINTS

The following exploratory endpoints will also be recorded: AF, any intracranial hemorrhage, major hemorrhage including any intracranial hemorrhage, symptomatic hemorrhagic transformation of an ischemic stroke, transient ischemic attack, myocardial infarction, minor hemorrhage, systemic embolism, symptomatic deep venous thrombosis, symptomatic pulmonary embolism, ischemic vascular death, and hemorrhagic vascular death. The endpoint of any intracranial hemorrhage will be subclassified as: 1) symptomatic versus asymptomatic, and 2) consisting of hemorrhagic transformation of the index brain infarct versus not.

11 SAFETY ANALYSES

11.1 SAFETY MONITORING

The MSM and DSMB will receive periodic safety reports of all SAEs. The detailed guidelines for monitoring for safety by the MSM, and the DSMB will be provided in a separate ARCADIA Safety Monitoring Plan.

11.2 DESCRIPTIVE SUMMARY OF EVENTS

All clinical safety endpoints and SAEs will be summarized by AE code (as provided on the AE CRF) in terms of frequency of the event, number of participants having the event, severity, and relatedness to the study treatment. Clinically important adverse events include:

- Symptomatic intracranial hemorrhage (including symptomatic hemorrhagic transformation of an ischemic stroke).
- Major hemorrhage other than intracranial hemorrhage.
- All-cause mortality.

The proportion of participants experiencing each of these events will be provided in the closed report to the DSMB by treatment arm with two-sided 95% CIs and unadjusted relative risks. Based on prior reports, the following approximate annual rates of major safety endpoints are expected:

**Adverse Event**	**Apixaban**	**Aspirin**
Symptomatic intracranial hemorrhage	1.5%	1.5%
Major hemorrhage other than intracranial hemorrhage	4.0%	3.0%
All-cause mortality	3.5%	3.5%

Log-rank tests will be utilized to assess the treatment group differences in the rates of: (1) first symptomatic intracranial hemorrhage; (2) first major hemorrhage other than intracranial hemorrhage; and (3) death from any cause.

11.3 ANALYSIS OF SAFETY OUTCOMES AT TRIAL COMPLETION

All safety outcome measurements are analyzed using the safety sample (Section 8.3). Sensitivity analyses will be conducted using the ITT population. At the end of the study, the cumulative incidences

of the pre-specified safety events will be compared between the two treatment groups at a two-sided alpha level of 0.05.

11.3.1 PRIMARY SAFETY ENDPOINTS

The primary safety outcomes are: (A) symptomatic intracranial hemorrhage (including symptomatic hemorrhagic transformation of an ischemic stroke), and (B) major hemorrhage other than intracranial hemorrhage.

11.3.2 SECONDARY SAFETY ENDPOINT

The secondary safety endpoint is all-cause mortality.

11.4 ADJUDICATION OF SAFETY ENDPOINTS

Although regulatory reporting of possible SAEs will be based on the MSM determination of the event, for the publication of the final trial results analyses will use events as determined by the outcomes adjudication committee or site investigator (in the case of endpoints not adjudicated).

12 SUBGROUP ANALYSES

In prespecified subgroup analyses, the analyses in Sections 10.1 (primary efficacy analyses) and 11 (safety analyses) will be stratified by age (≥75 years versus <75 years), sex/gender, race/ethnicity, and weight (<70 kg versus ≥70 kg). In particular, subgroup analyses of previous trials of anticoagulation in ESUS suggest a benefit in patients ≥75 years of age.[7]

13 QUALITY-OF-LIFE ANALYSES

13.1 QUALITY-OF-LIFE MEASURES

Participants' quality of life will be assessed at the Day 540 visit (18 months). Study investigators will elicit quality-of-life data from participants or their proxies using modules from PROMIS, a validated tool for assessing quality-of-life in patients with disorders including stroke. The Global Health Short Form v1.1 and the Physical Function Short Form v1.2 will be used. Missing data for quality-of-life measures will not be imputed.

13.2 ANALYSES OF QUALITY-OF-LIFE MEASURES

The scores from each PROMIS domain will be compared between treatment groups using generalized linear mixed models which can account for correlation between the physical and global health forms.

14 COST-EFFECTIVENESS ANALYSIS

The cost-effectiveness of treatment with apixaban as compared with aspirin in patients with cryptogenic stroke and atrial cardiopathy will be assessed using previously published methods (Kamel, *Neurology*, 2012). In brief, a Markov decision model will be created based on the wholesale cost of apixaban and data from the ARCADIA trial. The model will quantify the cost and quality-adjusted life expectancy resulting from apixaban therapy compared with aspirin. The base case population will be a patient cohort with the mean age of the ARCADIA cohort and a history of prior stroke. The base case will include the following health states: No disability (history of transient ischemic attack or ischemic stroke with full recovery), ischemic stroke (fatal or resulting in moderate-to-severe, mild, or no disability), intracranial hemorrhage (fatal or resulting in moderate-to-severe or mild disability), recurrent stroke or combined stroke and intracranial hemorrhage, extracranial hemorrhage, myocardial infarction, and death. Model inputs on the rates of these adverse events (e.g., recurrent ischemic stroke, intracranial hemorrhage, major hemorrhage, myocardial infarction) will be taken directly from the average annual rates of these events in the apixaban versus aspirin arms of the ARCADIA trial. Costs and quality-of-life estimates will be based on previously published data as well as quality-of-life data from ARCADIA (see Section 13 above). The model will adopt a societal perspective and will exclude costs unrelated to the treatment strategy in question (apixaban versus aspirin) or other disease states besides those listed above. Quality-adjusted life expectancy and net costs will be quantified over 20 years or until death, whichever occurs first. Life-years and costs will be discounted at 3% per year and reported as quality-adjusted life-years (QALYs) and 2023 U.S. dollars (rounded to the nearest $100). TreeAge Pro software (TreeAge Software, Williamstown, Massachusetts) will be used to build the model and perform all analyses.

15 UPDATE OF TRIAL OPERATING PARAMETERS

As a trial with time-to-event outcome, the power can be affected by aspects other than the observed event rate (i.e. recruitment rate leading to decreased follow-up time for participants). At the recommendation of the DSMB, trial design modifications to rectify this problem may be considered, namely:

- Increased follow-up period.
- Increased sample size (i.e. observed accrual rate and increased accrual duration).
- Increased number of sites.

These estimates will be made using the assumptions above (Section 7), namely:

- 7% annual risk of recurrent stroke of any type in aspirin-treated participants.
- Clinically relevant relative risk reduction of 40% (hazard ratio of 0.6, hazard rate of 4.2%) for participants treated with apixaban.
- 3% annual rate of crossover from blinded aspirin to open-label anticoagulation due to AF detection.
- 3% annual rate of crossover from blinded apixaban to open-label antiplatelet therapy due to bleeding or other adverse events.
- 4% annual rate of loss to follow-up or death in each group.

16 CHANGE LOG

Date	Relation to interim or other analysis	Issue	Trigger	Solution
3 Jan 2018	Changes preceded start of recruitment	Elimination of plan for sample size re-estimation	Not logistically feasible	No sample size re-estimation
3 Jan 2018	Changes preceded start of recruitment	Elimination of plan for revision to group sequential boundaries	Inherent difficulty in accurately estimating	No plan for revision of group sequential boundaries
3 Jan 2018	Changes preceded start of recruitment	Randomization method	Balancing variables felt to be arbitrary	Randomization will only control the maximum tolerated imbalance within each RCC
9 Nov 2018	Changes preceded first interim analysis	Typographical error in Section 7.1	Loss-to-follow-up/mortality rate incorrectly written as 4%	Corrected to 5%
31 Mar 2021	Changes preceded first interim analysis	Safety-analysis sample	Protocol change	Safety-analysis sample changed to include safety events from randomization through 30 days after permanent study drug discontinuation
31 Mar 2021	Changes preceded first interim analysis	Subgroup analysis plan	Recent preliminary data that efficacy of aspirin may vary by weight	Subgroup analysis by weight added
31 Mar 2021	Changes preceded first interim analysis	Miscellaneous	Protocol change	Several minor edits to maintain consistency with protocol
29 Oct 2021	Changes preceded first interim analysis	Miscellaneous	Clarifications ahead of interim analysis	Several minor edits to maintain consistency with Manual of Procedures; clarified that log-log plots will be used to examine

				proportional hazards assumption in secondary Cox models; added additional secondary analysis in section 10.1.6.2
15 Feb 2023	Changes preceded final analysis	Subgroup analyses plan	Recent preliminary data that efficacy of apixaban in ESUS may vary by age	Subgroup analysis by age added; also clarified patient accountability flowchart and fixed typographical errors, including inadvertent holdover of section about adjusting for site effects in primary analysis

17 REFERENCES

1. Longstreth WT, Jr., Kronmal RA, Thompson JL, Christenson RH, Levine SR, Gross R, Brey RL, Buchsbaum R, Elkind MS, Tirschwell DL, et al. Amino Terminal Pro-B-Type Natriuretic Peptide, Secondary Stroke Prevention, and Choice of Antithrombotic Therapy. *Stroke*. 2013;44:714-719.
2. Connolly SJ, Eikelboom J, Joyner C, Diener HC, Hart R, Golitsyn S, Flaker G, Avezum A, Hohnloser SH, Diaz R, et al. Apixaban in Patients with Atrial Fibrillation. *N Engl J Med*. 2011;364:806-817.
3. Ruff CT, Giugliano RP, Braunwald E, Hoffman EB, Deenadayalu N, Ezekowitz MD, Camm AJ, Weitz JI, Lewis BS, Parkhomenko A, et al. Comparison of the Efficacy and Safety of New Oral Anticoagulants with Warfarin in Patients with Atrial Fibrillation: A Meta-Analysis of Randomised Trials. *Lancet*. 2014;383:955-962.
4. Granger CB, Alexander JH, McMurray JJ, Lopes RD, Hylek EM, Hanna M, Al-Khalidi HR, Ansell J, Atar D, Avezum A, et al. Apixaban Versus Warfarin in Patients with Atrial Fibrillation. *N Engl J Med*. 2011;365:981-992.
5. Kernan WN, Ovbiagele B, Black HR, Bravata DM, Chimowitz MI, Ezekowitz MD, Fang MC, Fisher M, Furie KL, Heck DV, et al. Guidelines for the Prevention of Stroke in Patients with Stroke and Transient Ischemic Attack: A Guideline for Healthcare Professionals from the American Heart Association/American Stroke Association. *Stroke*. 2014;45:2160-2236.
6. Little RJ, D'Agostino R, Cohen ML, Dickersin K, Emerson SS, Farrar JT, Frangakis C, Hogan JW, Molenberghs G, Murphy SA, et al. The Prevention and Treatment of Missing Data in Clinical Trials. *N Engl J Med*. 2012;367:1355-1360.
7. Diener H-C, Sacco RL, Easton JD, Granger CB, Bar M, Bernstein RA, Brainin M, Brueckmann M, Cronin L, Donnan G, et al. Antithrombotic Treatment of Embolic Stroke of Undetermined Source. *Stroke*. 2020;51:1758-1765.

ADDENDUM 25 JUL 2023

Definitions of the censoring times for the primary and secondary analyses

For primary efficacy analysis:

Participants will be censored on the earliest of:

1. *Date of primary efficacy outcome event.*
2. *Date of death.*
3. *Date of last contact. A reported primary efficacy outcome event or death will count as "contact". Chart review visits with the patient found in the EMR will count as "contact", but chart review visits with the patient not found in the EMR will not count as "contact". If no "contact" is available, the date of early termination (F126q03) will count as the date of last contact. For subjects did not have a signed informed consent V7 or V8, last contact on or before 48 Month visit will counted as last contact.*
4. *Date of first contact of any kind (follow up or EOS visit) on or after December 21, 2022.*
5. *February 28, 2023.*

Any primary efficacy outcomes occurring after this censoring date will not count for the primary efficacy analysis.

For secondary efficacy analyses:

Participants will be censored on the earliest of:

1. *Date of secondary efficacy outcome event.*
2. *Date of primary efficacy outcome event.*
3. *Date of death.*
4. *Date of last contact. A reported primary efficacy outcome event, secondary efficacy outcome event or death will count as "contact". Chart review visits with the patient found in the EMR will count as "contact", but chart review visits with the patient not found in the EMR will not count as "contact". If no "contact" is available, the date of early termination (F126q03) will count as the date of last contact. For subjects did not have a signed informed consent V7 or V8, last contact on or before 48 Month visit will counted as last contact.*
5. *Date of first contact of any kind (follow up or EOS visit) on or after December 21, 2022.*
6. *February 28, 2023.*

Any secondary efficacy outcomes occurring after this censoring date will not count for the secondary efficacy analyses.

For safety analyses:

The safety analyses will be conducted in both safety sample and ITT population (sensitivity analysis).

For the **safety sample**, the censoring time will be the earliest of:

1. *Date of safety outcome event.*
2. *Date of primary efficacy outcome event.*
3. *Date of death.*

4. *Date of last contact. A reported primary efficacy outcome event, safety outcome event or death will count as "contact". Chart review visits with the patient found in the EMR will count as "contact", but chart review visits with the patient not found in the EMR will not count as "contact". If no "contact" is available, the date of early termination (F126q03) will count as the date of last contact. For subjects did not have a signed informed consent V7 or V8, last contact on or before 48 Month visit will counted as last contact.*
5. *Date of first contact of any kind (follow up or EOS visit) on or after December 21, 2022.*
6. *February 28, 2023.*
7. *30 days after permanently discontinuation of study drug or if the stop date is unknown, 130 days after last study drug dispensing date will be used.*

Any safety outcomes occurring after this censoring date will not count for safety sample analyses.

For the **ITT population**, the censoring time will be the <u>earliest</u> of

1. *Date of safety outcome event.*
2. *Date of primary efficacy outcome event.*
3. *Date of death.*
4. *Date of last contact. A reported primary efficacy outcome event, safety outcome event, or death will count as "contact". Chart review visits with the patient found in the EMR will count as "contact", but chart review visits with the patient not found in the EMR will not count as "contact". If no "contact" is available, the date of early termination (F126q03) will count as the date of last contact. For subjects did not have a signed informed consent V7 or V8, last contact on or before 48 Month visit will counted as last contact.*
5. *Date of first contact of any kind (follow up or EOS visit) on or after December 21, 2022.*
6. *February 28, 2023.*

Any safety outcomes occurring after this censoring date will not count for ITT safety analyses.

ARCADIA

AtRial Cardiopathy and Antithrombotic Drugs In prevention After cryptogenic stroke

Clinicaltrial.gov: NCT03192215

STATISTICAL ANALYSIS PLAN

SAP Version 1.1
Protocol Version 3.0
Date: 8 Jan 2018

Primary Trial Statistician, Blinded
Richard A. Kronmal, PhD
School of Public Health
University of Washington
Seattle, WA 98101

NDMC Trial Statistician, Unblinded
Caitlyn Meinzer, PhD
Department of Public Health Sciences
Medical University of South Carolina
Charleston, SC 29425

TABLE OF CONTENTS

1 LIST OF ABBREVIATIONS 4
2 KEY ROLES 5
3 STATISTICAL ANALYSIS PLAN AND STATISTICAL REPORTS 5
4 BACKGROUND AND RATIONALE 6
5 SYNOPSIS OF THE STUDY 7
6 OUTCOME VARIABLES 7
6.1 PRIMARY EFFICACY OUTCOME 7
6.2 SECONDARY EFFICACY OUTCOMES 7
6.3 PRIMARY SAFETY OUTCOMES 8
6.4 SECONDARY SAFETY OUTCOMES 8
7 SAMPLE SIZE DETERMINATION FOR PRIMARY EFFICACY OBJECTIVE 8
7.1 SAMPLE SIZE ESTIMATION 8
7.2 REVISIONS TO THE GROUP SEQUENTIAL BOUNDARIES 9
8 DEFINITION OF TARGET POPULATION AND STUDY SAMPLES 9
8.1 TARGET POPULATION 9
8.2 INTENT-TO-TREAT SAMPLE 9
8.3 SAFETY-ANALYSIS SAMPLE 9
8.4 PER-PROTOCOL SAMPLE 9
9 GENERAL STATISTICAL CONSIDERATIONS 10
9.1 PARTICIPANT ACCOUNTABILITY 10
9.2 RANDOMIZATION 10
9.3 BLINDING 10
9.4 CENSORING PARTICIPANTS 12
9.4.1 PRIMARY OUTCOME 12
9.4.2 DEATH 12
9.4.3 CROSSOVER TO OPEN-LABEL DRUG 12
9.4.4 WITHDRAWN CONSENT AND LOSS TO FOLLOW-UP 12
9.4.5 SECONDARY AND SAFETY OUTCOMES 12
9.5 TREATMENT GROUP COMPARABILITY 12
9.6 SITE EFFECTS 13
9.7 PARTICIPANT ADHERENCE 13
10 EFFICACY ANALYSIS 13
10.1 PRIMARY EFFICACY ANALYSIS 13
10.1.1 PARAMETER DESCRIPTION 13
10.1.2 STATISTICAL HYPOTHESES 13
10.1.3 STATISTICAL MODEL 13
10.1.4 INTERIM ANALYSIS METHOD 14
10.1.5 FINAL ANALYSIS METHOD 15
10.1.6 ADDITIONAL ANALYSES OF THE PRIMARY EFFICACY OUTCOME 16
10.2 INTERACTION OF ATRIAL CARDIOPATHY SEVERITY AND TREATMENT EFFECT 16
10.2.1 TESTS OF INTERACTION 16
10.2.2 IDENTIFICATION OF HIGH-RISK SUBGROUPS 17
10.2.3 ADDITIONAL EXPLORATORY MODELING 17
10.2.4 STATISTICAL MODEL 18

10.3 SECONDARY EFFICACY ANALYSIS 18
10.3.1 PARAMETER DESCRIPTION 18
10.3.2 STATISTICAL HYPOTHESES 18
10.3.3 STATISTICAL MODEL 19
10.3.4 EXPLORATORY EFFICACY ANALYSES 19
11 SAFETY ANALYSES 19
11.1 SAFETY MONITORING 19
11.2 DESCRIPTIVE SUMMARY OF EVENTS 19
11.3 ANALYSIS OF SAFETY OUTCOMES AT TRIAL COMPLETION 20
11.3.1 PRIMARY SAFETY ENDPOINTS 20
11.3.2 SECONDARY SAFETY ENDPOINT 20
11.3.3 EXPLORATORY SAFETY ENDPOINTS 20
11.4 ADJUDICATION OF SAFETY ENDPOINTS 21
12 SUBGROUP ANALYSES BY SEX/GENDER AND RACE/ETHNICITY 21
13 QUALITY-OF-LIFE ANALYSES 21
13.1 QUALITY-OF-LIFE MEASURES 21
13.2 ANALYSES OF QUALITY-OF-LIFE MEASURES 21
14 COST-EFFECTIVENESS ANALYSIS 21
15 UPDATE OF TRIAL OPERATING PARAMETERS 22
16 CHANGE LOG 22
17 REFERENCES 23

1 LIST OF ABBREVIATIONS

AE	Adverse event
AF	Atrial fibrillation
AHA/ASA	American Heart Association/American Stroke Association
ARCADIA	AtRial Cardiopathy and Antithrombotic Drugs In prevention After cryptogenic stroke
CRF	Case report form
CI	Confidence interval
DCU	Data Coordination Unit
DCR	Data clarification request
DSMB	Data and safety monitoring board
ECG	Electrocardiogram
ESUS	Embolic stroke of undetermined source
HR	Hazard ratio
ITT	Intention to treat
MOP	Manual of procedures
NCC	NIH StrokeNet National Coordinating Center
NDMC	NIH StrokeNet National Data Management Center
NIH	National Institutes of Health
NIHSS	National Institutes of Health Stroke Scale
NINDS	National Institute of Neurological Disorders and Stroke
NOAC	Non-vitamin K antagonist oral anticoagulant drug
NT-proBNP	Amino terminal pro-B-type natriuretic peptide
PI	Principal Investigator
PROMIS	Patient-Reported Outcomes Measurement Information System
$PTFV_1$	P-wave terminal force in ECG lead V_1
QVSFS	Questionnaire for Verification of Stroke-Free Status
RCC	Regional Coordinating Center
SAE	Serious adverse event
SD	Standard deviation
SOP	Standard operating procedure

2 KEY ROLES

Primary Trial Statistician, Blinded
Richard A. Kronmal, PhD
School of Public Health
University of Washington
Seattle, WA 98101

NDMC Trial Statistician, Unblinded
Caitlyn Meinzer, PhD
Department of Public Health Science
Medical University of South Carolina,
Charleston, SC, 29425

Contact PI
Mitchell S.V. Elkind, MD, MS

Protocol PI
Hooman Kamel, MD

3 STATISTICAL ANALYSIS PLAN AND STATISTICAL REPORTS

This document provides the details of statistical analyses planned for the ARCADIA trial, including interim analyses for efficacy and futility. In addition, it discusses the statistical issues relevant to these analyses (e.g., sample data to be used, missing data, adjustments for multiplicity, etc.).

The DCU generates two statistical reports: an open report to be distributed to the ARCADIA Executive Committee and the DSMB, and a closed report to be distributed only to the DSMB. The timing of these reports is determined in consultation with the DSMB. Reports will be sent from the DCU to the NINDS liaison two weeks in advance of the scheduled meeting.

Each report provides cumulative summary statistics on enrollment; participant status in the study (e.g., number who completed each study visit assessment); baseline characteristics; protocol violations; data management/quality information (e.g., timeliness and completeness of data entry by the clinical sites; number of DCRs generated and resolved); and safety data, including SAEs by AE code, severity, expectedness (anticipated/unanticipated), and relatedness to the study medication. Aggregate rates of SAEs excluding clinical outcomes (stroke [ischemic, hemorrhagic, or undetermined type], symptomatic hemorrhagic transformation of ischemic stroke, intracranial hemorrhage excluding either hemorrhagic stroke or symptomatic hemorrhagic transformation of ischemic stroke, transient ischemic attack, major hemorrhage excluding intracranial hemorrhage, minor hemorrhage, atrial fibrillation/flutter, myocardial infarction, systemic embolism, symptomatic deep venous thrombosis, and symptomatic pulmonary embolism) are provided in the open report; data on all other endpoints are restricted to the closed report. The statistics are provided for the overall study as well as by site or RCC, where appropriate, in the open report. For the closed report only, the statistics are also provided by treatment group (A vs B). If a report coincides in timing with a planned interim analysis, the analysis results are appended to the report.

The DCU also generates a blinded quarterly report to be distributed to the ARCADIA Executive Committee which contains updated aggregate data on enrollment, baseline, safety information, and loss to follow-up and crossovers. In addition, this report contains a table that shows what proportion of consented patients were randomized and on which atrial cardiopathy marker(s) these participants were deemed eligible, including summary statistics of the atrial cardiopathy marker values.

4 BACKGROUND AND RATIONALE

In one-third of ischemic strokes, a specific cause cannot be identified. Many of these cryptogenic strokes appear to arise from a distant embolic source. Recent evidence suggests that some cryptogenic strokes may arise from left atrial thromboembolism that goes unrecognized because it has not manifested with atrial fibrillation/flutter (AF). These data suggest that an underlying atrial cardiopathy may provide the substrate for thrombus formation and embolization even in the absence of AF.

In parallel with these new insights about cryptogenic stroke, new therapeutic options for stroke prevention have become available. Non-vitamin K antagonist oral anticoagulant (NOAC) drugs such as apixaban may be more effective than aspirin for treatment of cryptogenic stroke. A benefit is especially likely in cryptogenic stroke patients with atrial cardiopathy because of parallels with AF and because an analysis of data from the WARSS/APASS studies suggests that cryptogenic stroke patients with one marker of atrial cardiopathy (elevated NT-proBNP) and no obvious AF benefit from anticoagulant therapy.[1] Apixaban is a particularly attractive choice because it has a low bleeding risk,[2,3] lowers mortality more than warfarin in patients with AF,[4] and is the only NOAC drug with a Class 1A recommendation in recent AHA/ASA guidelines.[5]

The ARCADIA trial is designed to address several important knowledge gaps. First, it will advance knowledge about stroke pathophysiology by assessing whether atrial cardiopathy is a valid therapeutic target, which may set the stage for a primary prevention trial. Second, this trial will advance knowledge about optimal secondary stroke prevention therapy. One industry-sponsored trial (NAVIGATE-ESUS) that compared rivaroxaban to aspirin in patients with cryptogenic stroke was stopped early for futility. Another industry-sponsored trial (RESPECT-ESUS) is comparing dabigatran versus aspirin in patients with cryptogenic stroke. By including all cryptogenic stroke patients and by including those with up to 6 minutes of AF per day, these trials have mixed patients with heterogeneous stroke mechanisms. These trials may thus fail to show an overall benefit (as in NAVIGATE-ESUS), or may show an overall benefit driven mostly by patients with known AF and atrial cardiopathy, resulting in an overly broad indication for anticoagulant therapy. In the first instance, the ARCADIA trial may ensure that a valuable treatment in a specifically targeted subgroup will not be prematurely abandoned. In the second instance, the results of the ARCADIA trial would provide a compelling rationale to perform subgroup analyses of the industry-sponsored trials and conduct future trials to determine the risks and benefits of anticoagulant therapy across biologically distinct subgroups of stroke patients. Given the expense and risks of bleeding associated with anticoagulant drugs, it is imperative to define as precisely as possible the groups of stroke patients who would and would not benefit from their use. Therefore, the approach taken by the ARCADIA trial comports well with the general move toward precision medicine.

5 SYNOPSIS OF THE STUDY

ARCADIA is a multicenter, biomarker-driven, randomized, double-blind, active controlled Phase III clinical trial of 1,100 participants, conducted at approximately 120 enrolling sites. Enrollment is anticipated to occur over 2.5 years, with participants followed for a minimum of 1.5 years and a maximum of 4 years. Patients who have evidence of atrial cardiopathy and a recent stroke of undetermined source by current criteria will be randomly assigned to apixaban or aspirin (active control therapy) in a 1:1 ratio. Atrial cardiopathy will be defined as one or more of the following biomarkers: P-wave terminal force in electrocardiogram lead V1 >5,000 µV*ms, left atrial size index ≥3.0 cm/m2 on echocardiogram, and serum amino terminal pro-B-type natriuretic peptide >250 pg/mL. Standard heart-rhythm monitoring will be performed before enrollment to exclude as thoroughly as possible those patients with atrial fibrillation. Therapy must be initiated within 3-120 days of enrolling stroke. The primary efficacy endpoint is time to recurrent stroke (ITT analysis). Secondary efficacy endpoints are time to (1) recurrent stroke or death and (2) recurrent ischemic stroke or systemic embolism. There will be a test of the interaction of the atrial cardiopathy selection variables with treatment arm for the primary outcome. The primary safety outcomes are: (1) symptomatic intracranial hemorrhage (including symptomatic hemorrhagic transformation of an ischemic stroke) and (2) major hemorrhage other than intracranial hemorrhage. Additional safety endpoints include all-cause mortality.

6 OUTCOME VARIABLES

6.1 PRIMARY EFFICACY OUTCOME

The primary efficacy outcome is recurrent stroke of any type (ischemic, hemorrhagic, or of undetermined type). Possible stroke events will be centrally adjudicated, and the primary outcome measure will be defined as the centrally adjudicated stroke event, NOT the site determined event.

If a site identifies an event as a possible primary efficacy outcome, the participant will either continue, pause, or stop study medication at the discretion of the treating physician. If the adjudication committee determines the event meets the primary efficacy definition, the participant will stop participation in the study, including stopping study drug; otherwise they may stop, continue, or resume study drug at the discretion of the treating physician.

6.2 SECONDARY EFFICACY OUTCOMES

The secondary efficacy outcomes are: (A) composite of recurrent ischemic stroke or systemic embolism, and (B) composite of recurrent stroke of any type or death from any cause. Possible events will be centrally adjudicated, and the outcome measure will be defined as the centrally adjudicated event, NOT the site determined event. Note that recurrent ischemic stroke is a subset of the primary outcome definition and thus the participant would stop participation in the study, including stopping study drug. In the case of systemic embolism, the participant may continue in the trial until the end of follow-up or occurrence of the primary endpoint.

6.3 PRIMARY SAFETY OUTCOMES

The primary safety outcomes are: (A) symptomatic intracranial hemorrhage (including symptomatic hemorrhagic transformation of an ischemic stroke), and (B) major hemorrhage other than intracranial hemorrhage. Symptomatic intracranial hemorrhage is defined as any extravascular blood within the cranium, including subdural and epidural blood, associated with and identified as the predominant cause of new neurologic symptoms, including headache, or leading to death. Major hemorrhage other than intracranial hemorrhage is defined as clinically overt bleeding accompanied by a ≥2 g/dL decrease in the hemoglobin level during a 24-hour period, transfusion of ≥2 units of whole blood or red cells, involvement of a critical non-intracranial site (intraspinal, intraocular, pericardial, intraarticular, intramuscular with compartment syndrome, or retroperitoneal), or death.

Note that major symptomatic intracranial hemorrhage may not meet the primary outcome definition of stroke, in which case the participant is eligible to continue in the trial; this is true for other safety outcomes as well.

6.4 SECONDARY SAFETY OUTCOMES

The secondary safety endpoint is all-cause mortality. Note that death is included as part of a composite secondary efficacy endpoint.

7 SAMPLE SIZE DETERMINATION FOR PRIMARY EFFICACY OBJECTIVE

7.1 SAMPLE SIZE ESTIMATION

The primary outcome variable is the time to recurrent stroke of any type. Preliminary data suggest a 7% annual risk of recurrent stroke of any type in aspirin-treated patients, with a clinically relevant relative risk reduction of 40% (hazard ratio of 0.6, hazard rate of 4.2%) for patients treated with apixaban. Based on preliminary feasibility data and 120 sites, ARCADIA plans to recruit 1,100 participants during a 30-month uniform accrual period and follow these participants to the end of the study (i.e., 18-month minimum follow-up period). In addition, it is anticipated that there will be a 3% annual rate of crossover from blinded aspirin to open-label anticoagulation due to AF detection, and a 3% annual rate of crossover from blinded apixaban to open-label antiplatelet therapy due to bleeding or other adverse events. Finally, it is estimated that there will be a 4% annual rate of loss to follow-up or death in each group.

Given the assumptions above, enrollment of 1,100 participants (approximately 150 recurrent stroke events) will provide 80% power to demonstrate a statistically significant difference in the hazard for recurrent stroke with apixaban compared to aspirin at an alpha level of 0.05 while allowing for one interim look for efficacy (or harm) and futility after 1/2 of expected events have occurred (75 events). To maintain the power as planned and the overall alpha of the study at 0.05, an O'Brien-Fleming type Lan-DeMets error spending function will be used. The sample size was calculated using the "Design: Survival Endpoint: Two-Sample Test – Parallel Design – Logrank" program in EAST® v6 software (Cytel Corporation) (Lan and DeMets, 1983).

7.2 REVISIONS TO THE GROUP SEQUENTIAL BOUNDARIES

It is possible that the information fraction at the interim analysis may be incorrect due to incorrect estimation of the total number of events in the trial. However, given this trial's extremely conservative interim analysis boundaries, it is unlikely that such misestimation would substantively affect the final test statistic. Furthermore, there is inherent difficulty in accurately estimating the total number of events even at the time of the interim analysis. Therefore, there will be no revisions to the group sequential boundaries.

8 DEFINITION OF TARGET POPULATION AND STUDY SAMPLES

8.1 TARGET POPULATION

The population that will be randomized in the ARCADIA trial comprises patients 45 years of age and older who have a clinical diagnosis of embolic stroke of undetermined source (ESUS) within the prior 120 days, a modified Rankin Scale score ≤4 at the time of screening, and at least one of the following criteria for atrial cardiopathy:

- [ECG]: PTFV1 >5,000 μV*ms on 12-lead ECG.
- [ECHO]: Left atrial size index ≥3 cm/m^2 on echocardiogram.
- [NT-proBNP]: Serum NT-proBNP >250 pg/mL.

8.2 INTENT-TO-TREAT SAMPLE

Under the ITT principle, the evaluable sample includes all participants who are randomized. Each participant will be analyzed according to the treatment group to which they were randomly assigned at the time of randomization.

8.3 SAFETY-ANALYSIS SAMPLE

The safety sample includes all participants who are randomized and receive at least one dose of study medication. The primary safety analysis will define treatment arm as the treatment to which a participant was randomized. Additional sensitivity analyses will define a time-dependent treatment covariate, and possibly a lagged time-dependent treatment covariate.

8.4 PER-PROTOCOL SAMPLE

In addition to the defined ITT analysis sample, a per-protocol sample is defined as a subset of the ITT sample. This sample will be used for sensitivity analyses of the primary and secondary efficacy outcomes. The per-protocol sample will include all randomized participants that do not have the following protocol deviations:

- Eligibility violation.
- Treatment never started.

For these sensitivity analyses, treatment will first be defined as randomized, but a time-dependent treatment covariate with concurrent treatment effects may be considered.

9 GENERAL STATISTICAL CONSIDERATIONS

9.1 PARTICIPANT ACCOUNTABILITY

A flowchart will be created to present a summary of participants' status. This flowchart will first list the number of patients who were eligible and consented to be screened for the biomarkers. Of consented patients, the chart will then delineate those who did not meet criteria for any of the three biomarkers, those who were positive for at least one biomarker but no longer eligible at the time of randomization, and those randomized to a treatment group. Then, within each treatment group, it will list the number of participants who received at least one dose of study drug, the number of participants who did not receive any study drug, the number of participants who had already experienced an outcome event before receiving the first dose of study drug, and the number of participants who received the first dose of study drug before an outcome event.

9.2 RANDOMIZATION

Eligible participants will be allocated in a 1:1 ratio to apixaban or aspirin using a randomization method that controls the maximum tolerated imbalance within each RCC. Implementation details are provided in the randomization plan and validation documentation.

A web-based central randomization system will be developed by the NDMC and installed on the WebDCU™ ARCADIA study website. The randomization algorithm and its implementation will be fully detailed in the ARCADIA Randomization Plan and the Randomization Validation documents. These documents will be developed prior to enrollment initiation and stored in a secure location at the NDMC. The documents will be archived with the study database at the end of the trial.

The participant randomization system is fully integrated into the ARCADIA study database, and presented as a special CRF in the electronic data capture module. Data edit permission for the randomization CRF is centrally managed based on user groups. Site study team members are limited to viewing and editing data only for participants in their own site. Site enrollment status, participant eligibility, and site study drug availability are verified prior to the performance of participant randomization in order to protect data integrity. The participant randomization system checks the drug availability at both the site level and the individual participant level and triggers site study drug shipment and participant drug resupply requests accordingly.

Participant treatment assignment information is masked by using the Study Drug ID, which is a globally unique no-sequence 6-digit number placed on each study drug kit. Access to treatment assignment information is prohibited for all blinded investigators. Authorized emergency unblinding for medical safety purposes is provided through a medical emergency hotline with one of the trial PIs responding to provide permission for unblinding, followed by unblinding to the treating physician. The PIs remain blinded to study treatment. All cases of unblinding are recorded in the study database.

9.3 BLINDING

The study is conducted in a double-blinded manner. Study drug administration, clinical care, and outcomes assessments are made by the clinical site staff blinded to the treatment assignment.

Participants will not be told of their actual treatment assignment until the trial is complete, at which point assignments are available upon request, or during the trial in the event of a medical emergency that requires unblinding. The Executive Committee and the MSM are also blinded to the treatment assignment.

The NDMC biostatistician and statistical programmer will be partially unblinded; they know the participants' group classification (A or B), but not whether A is apixaban or aspirin. During the course of the trial it may be necessary for them to become unblinded if the DSMB requests. The clinical adjudicators will attempt to remain blinded, but information in the narrative may on rare occasions unblind specific cases. The Executive Committee members are completely blinded.

The DCU's unblinded staff generates: (1) a hard copy list (obtained from the WebDCU™) of study drug kit numbers and their corresponding study drug (apixaban or aspirin) as well as the lot numbers and expiration dates; and (2) two identical sealed envelopes that contain identification of treatment codes (e.g., A = apixaban, B = aspirin). Prior to initiation of the trial, one envelope is given to the NINDS liaison to the DSMB. Another is maintained in a locked file cabinet in a limited access central file room along with the hard copy list described in (1).

The central pharmacy personnel at the StrokeNet NCC at the University of Cincinnati are completely unblinded. They have access to the WebDCU™ to match the study drug (apixaban or aspirin) to its corresponding study drug kit number.

The DSMB (as well as the NINDS liaison to the DSMB) is partially unblinded. However, if it so wishes, it may be completely unblinded at any time during the trial. One of the sealed envelopes described in (2) above is given to the NINDS liaison to the DSMB prior to initiation of the trial. If the DSMB wishes to be unblinded on a particular participant only, the NINDS liaison to the DSMB can email the request to the NDMC biostatistician.

In case of a medical emergency for a study participant that requires unblinding, the clinical site staff can become unblinded only to that participant's treatment by calling the emergency hotline. Clinical site staff would then request permission for unblinding to occur and provide a brief clinical summary to one of the responding trial PIs. The PI would then approve or not approve the request and forward that decision to the NDMC, who then provide unblinded information to the site investigator, but not to the PI. The PI will remain blinded to treatment assignment. Unblinding should only occur if a compelling clinical reason arises, such as active major bleeding or a pending decision on whether to administer intravenous thrombolysis for acute ischemic stroke. Participants who undergo unblinding will stop study drug but continue to be followed as part of the study and will be analyzed according to the intention-to-treat principle.

Details such as how kits will be labeled and how the study drug is blinded are provided in the Study Drug Plan.

9.4 CENSORING PARTICIPANTS

9.4.1 PRIMARY OUTCOME

Participants will be followed until the end of the trial follow-up period, which will be 48 months after the start of recruitment. Due to regulatory reasons, participants will have a final telephone visit 30 days after the trial follow-up period to assess SAEs related to discontinuation of study drug, but this 30-day period will not contribute to participants' time-at-risk for the purposes of analysis. Under the ITT principle, all participants who are randomized are included in the analysis. Participants will be censored at the date of their last follow-up visit in cases of loss to follow-up or withdrawn consent, or at the date of death in cases of death.

9.4.2 DEATH

The primary analysis codes death as a censoring event. Sensitivity analyses will be conducted where death is treated as a competing risk.

9.4.3 CROSSOVER TO OPEN-LABEL DRUG

If a participant is known to have permanently crossed over onto open-label drug (e.g., an anticoagulant in the case of AF diagnosis, antiplatelet drug or no antithrombotic therapy in the case of bleeding events on apixaban, antiplatelet therapy for a new coronary event), the participant will continue to be followed and analyzed in the treatment group they are assigned. A sensitivity analysis using a time-dependent treatment effect, as well as an analysis accounting for pill count compliance data, will be considered.

9.4.4 WITHDRAWN CONSENT AND LOSS TO FOLLOW-UP

Using recommended methods,[6] sensitivity analyses will be conducted to assess the effect of any censoring that occurs due to a participant being lost to follow-up or withdrawing consent. Based on other large stroke prevention trials, we anticipate no more than a 4% lost to follow up rate. At the time of each planned analysis (interim and final), the unblinded statistician will report the amount of missing primary outcome data.

9.4.5 SECONDARY AND SAFETY OUTCOMES

In cases where observation of the primary outcome precludes observation of secondary or safety outcomes, the primary analysis will treat the participant as censored. Sensitivity analyses will explore alternative modeling strategies, in particular competing risk, Markov models, or joint modeling approaches.

9.5 TREATMENT GROUP COMPARABILITY

A description of the baseline characteristics of trial participants will be presented by treatment group. Dichotomous variables will be summarized as number (%). Percentages will be calculated based on the number of participants with available data for that variable. Continuous variables will be summarized by the mean and SD. In the case of variables with missing values, the denominator will be stated in the

summary table or in a footnote to the summary table. The baseline characteristics of interest are age, sex, race, time from qualifying stroke, CHA_2DS_2-VASc score, and the value of each of the biomarker inclusion criteria. Statistical significance of covariates will not be assessed; instead 95% CIs will be presented. In addition, all of these covariates, regardless of imbalances between groups, will be included as covariates in multivariable regression in a secondary analysis of the primary aim.

9.6 SITE EFFECTS

Several procedures have been incorporated into the study design (i.e., procedure manual, training and certification programs, protocol violation monitoring, blinding) to reduce trial site effects; however, possible effects will not be ignored for this trial. The distribution of demographics will be examined by RCC using means (SD) and proportions (95% CIs), and RCC and RCC*treatment interaction terms will be included as a random effect in a secondary analysis of the primary outcome.

9.7 PARTICIPANT ADHERENCE

Participants will be requested to bring in their study pill bottles at study visits for pill counts. For each visit interval, adherence by pill count will be measured as (# pills supplied - # remaining pills)/# pills supplied. Adherence will be classified as low (<60%), medium (60-79%), or high (≥80%). A sensitivity analysis of the primary endpoint will use a time-dependent compliance indicator to assess the impact of non-compliance on hazard of stroke. Note that participants are supplied with a buffer of 10 pills per bottle. The calculation listed above will adjust for the number of days between visits (i.e., a participant with 100% compliance and 90 days between study visits will be listed as 100% compliant).

10 EFFICACY ANALYSIS

10.1 PRIMARY EFFICACY ANALYSIS

10.1.1 PARAMETER DESCRIPTION

The primary efficacy analysis will use the ITT sample to assess time to recurrent stroke of any type. Censoring of participants is described in Section 0.

10.1.2 STATISTICAL HYPOTHESES

The null hypothesis (H_0) is that the hazard ratio comparing apixaban versus aspirin for the primary endpoint will be 1. The alternative (H_A) is that the hazard ratio is not 1. That is,

$$H_0: h_{apix}(t) = h_{asp}(t) \quad \text{versus} \quad H_A: h_{apix}(t) \neq n_{asp}(t)$$

Where $h_{apix}(t)$ and $h_{asp}(t)$ are the hazard rates at time t for the apixaban and aspirin groups, respectively. The minimum effect size of clinical interest is a relative HR of 0.6. The estimated yearly event rate in the placebo group is assumed to be 7%.

10.1.3 STATISTICAL MODEL

$$Z = \frac{\sum_{i=1}^{D} W(t_i)\left[d_{i1} - Y_{i1}\left(\frac{d_i}{Y_i}\right)\right]}{\sqrt{\sum_{i=1}^{D} W(t_i)^2 \frac{Y_{i1}}{Y_i}\left(1-\frac{Y_{i1}}{Y_i}\right)\left(\frac{Y_i-d_i}{Y_i-1}\right)d_i}}$$

Which has a standard normal distribution for large samples when H_0 is true. Using this statistic, an α level test of the alternative hypothesis $H_A : h_1(t) \neq h_2(t)$, for some $t \neq \tau$, is rejected when $|Z| > Z_{\alpha/2}$. Let W(t)=1 for all t, which represents the log-rank test.

**Efficacy Outcome**	**Analysis Method to Compare Apixaban and Aspirin Groups (SAS® v9 Procedure)**
Log-Rank Test of Survival	PROC PHREG

```
/* THIS CODE WILL BE USED TO MODEL THE PRIMARY
ENDPOINT
NOTE: THE CODE USES A RANDOM EFFECT FOR TREATMENT
SITE; THIS SHOULD BE THE SAME COVARIATE USED IN THE
RANDOMIZATION
*/
proc phreg data= <Analysis Dataset>;
      class <Treatment Group Indicator>;
      model Time*Status(0)= <Treatment Group
Indicator>;
      hazardratio 'Hazard Ratio' <Treatment Group
Indicator>;
   run;
```

To test the null hypothesis, we will use the log-rank test.
In the above model, an event (Status) and event time (Time) are defined as follows:

First Event	Status	Time
Recurrent stroke of any type	1	Time of first stroke of any type
Systemic embolism	N/A (continue to follow)	
Death	0	Time of death
Diagnosis of AF	N/A (continue to follow)	
Study drug stopped	N/A (continue to follow)	
Loss to follow-up or consent withdrawn	0	Last known contact
Study team requires unblinding and recurrent stroke of any type has not yet occurred		N/A (continue to follow)

10.1.4 INTERIM ANALYSIS METHOD

The study is designed using one interim look for both efficacy and futility for the primary outcome, and one final look, for a total of two planned analyses of the primary outcome. The interim analysis plan uses the error spending function method with O'Brien-Fleming type stopping guidelines. The error spending function distributes the type I and II error rates across the interim monitoring points giving the flexibility of changing the intervals of monitoring while still preserving the overall type I and II error rates. The O'Brien-Fleming type boundary is considered conservative as its boundaries make it difficult to terminate a study early by requiring extreme early evidence of efficacy or futility. It spends smaller amounts of alpha at the first look and gradually increases the spending as more information is acquired. The trial may be stopped for overwhelming efficacy of one treatment group over the other or for futility at the planned interim analyses if the test statistic crosses the respective boundaries.

The prespecified plan is to conduct the first interim analysis after 75 cumulative adjudicated primary outcome events (estimated information fraction of ½), or approximately 800 participants have been randomized. Assuming an accrual rate of 36.7 participants per month, it is anticipated that the first look will occur roughly 3.5 years from the start of enrollment. The interval may be altered if requested by the DSMB. The stopping boundaries are defined using the Lans-DeMets O'Brien-Fleming type spending functions (Lan and DeMets, 1983). The futility boundaries are derived as non-binding, meaning that if a futility boundary is crossed it can be overruled without inflation of the type 1 error rate. If the crossing of an efficacy boundary is overruled, then this decision can impact the type II error rate but not the type I error rate. EAST® v6 software (Cytel Corporation) was used for the boundary calculations. The following table lists the test statistics at a particular look. The NDMC will be responsible for conducting these analyses and compiling the reports for the DSMB. Since several factors need to be taken into consideration before stopping a study, safety and study progress will also be taken into consideration by the DSMB and Executive Committee when deciding whether to stop the study if an efficacy or futility boundary is crossed.

Table. Nominal Critical Values and Alpha Levels for One Interim Analysis for Overwhelming Efficacy and Futility (O'Brien-Fleming, two-sided α = 0.05)

	Exp. # events under H_0	Cum. α	Efficacy Boundary		Cum. β	Futility Boundary	
			Z Scale	HR		Z Scale	HR
Interim Analysis	75	0.003	|Z|>2.963	HR>2.123 -OR- HR<0.471	0.04	|Z|<0.356	HR>0.914 -AND- HR<1.095
Final Analysis	150	0.05	|Z|>1.969	HR>1.424 -or- HR<0.702	0.20		

10.1.5 FINAL ANALYSIS METHOD

The primary outcome analysis of the time to stroke of any type will use a log-rank test statistic to assess whether there is a difference in the hazard comparing apixaban to aspirin. Outcome differences will be analyzed under the ITT principle, so all randomized participants will be included in the primary analysis sample. To assess efficacy, the treatment groups will be compared with respect to the hazard of experiencing a primary outcome event. A log-rank test will be performed to compare the treatment group proportions using a cumulative two-tailed significance level of 0.05. An unadjusted HR will be

reported with two-sided 95% CIs. At the final analysis, the null hypothesis will be rejected if the HR is greater than 1.424 or less than 0.702; we will fail to reject the null hypothesis at the 0.05 level of significance otherwise.

10.1.6 ADDITIONAL ANALYSES OF THE PRIMARY EFFICACY OUTCOME

10.1.6.1 Adjusting for Key Covariates

In secondary analyses of the primary outcome, we will adjust for the prognostic variables listed in Section 9.5. A Cox proportional hazards model will be used, after the verification of variable and model assumptions (e.g., normality and homoscedasticity) and goodness-of-fit assessments accompanying each analysis. These secondary analyses will be reported in the paper reporting the primary results of the trial.

10.1.6.2 Accounting for Crossover to Open-Label Therapy

In a secondary analysis, we will censor participants at the time of AF detection, when they are switched to open-label anticoagulation. This analysis will be used to explore the utility of anticoagulation in participants with atrial cardiopathy before they have manifested AF and therefore remain "cryptogenic" based on the prevailing clinical paradigm. Alongside this analysis, we will also perform an alternative analysis treating AF as a competing risk rather than a censoring point, given very recent theoretical data suggesting that apixaban may reduce the risk of AF itself, not just stroke.

10.1.6.3 Accounting for Unblinding

In a secondary analysis, we will censor participants at the time of unblinding, when they are switched to open-label antithrombotic therapy per the discretion of the treating physician.

10.2 INTERACTION OF ATRIAL CARDIOPATHY SEVERITY AND TREATMENT EFFECT

This secondary analysis will explore the relationship between the severity of atrial cardiopathy and the relative efficacy of apixaban over aspirin. The objective of this analysis is two-fold. First, it will be used to assess the degree to which the severity of atrial cardiopathy modifies the efficacy of anticoagulation. Second, it will be used, regardless of whether or not apixaban is superior to aspirin in the overall trial population, to identify high-risk subgroups of participants with severe atrial cardiopathy who substantially benefit from apixaban.

10.2.1 TESTS OF INTERACTION

A Cox regression model will be used to assess interactions between each of the three atrial cardiopathy biomarkers and treatment status. The dependent variable will be recurrent stroke of any type. Independent variables will be a categorical variable for treatment status (apixaban versus aspirin) and three continuous variables representing each atrial cardiopathy biomarker. The atrial cardiopathy biomarkers will be appropriately transformed if necessary (e.g., log-NT-proBNP). Two-way interaction terms will be included between the treatment status variable and each atrial cardiopathy biomarker variable.

The z statistics for the individual interaction hazard ratios will be used to test the significance of the interaction terms. Each of the tests for the three biomarker-treatment interactions will be considered statistically significant if its individual *P* value is <0.016. This conservative *P* value is chosen to account for the multiple hypothesis testing in this exploratory analysis. Given the hypothesis test for the primary endpoint, the tests of the three interactions will exceed an experiment-wide alpha threshold of 0.05, but the alpha threshold of 0.016 for each interaction test will provide a conservative approach to the evaluation of interactions. Given that these tests of interaction are exploratory analyses, formal power calculations are not provided.

10.2.2 IDENTIFICATION OF HIGH-RISK SUBGROUPS

If one or more of the interactions are statistically significant, a prediction score computed from the Cox model will be used to predict the degree of benefit of apixaban over aspirin in a given participant based on the severity of atrial cardiopathy. Such a risk score will be important regardless of the main findings of this trial. If Aim 1 is negative (no benefit of apixaban versus aspirin in the overall trial cohort), the prediction score will be used to identify subgroups of patients with severe atrial cardiopathy who may still benefit from apixaban despite the negative findings in the overall trial population. This would inform follow-up studies of antithrombotic therapy in patients with cryptogenic stroke. If Aim 1 is positive (apixaban proves superior to aspirin in the overall trial cohort), the prediction score will be used to assess whether absolute stroke risk reductions in the highest-risk atrial cardiopathy subgroups are large enough to justify a primary prevention trial.

10.2.3 ADDITIONAL EXPLORATORY MODELING

Since the effect of the atrial cardiopathy biomarkers or their interactions with the treatment assignment may be nonlinear, analyses will also be carried out using the generalized additive model in the Cox regression to allow for nonlinearities in the interactions of the biomarkers with treatment status. For this purpose, the approach and methodology described by Royston (Royston, 2000; Royston and Sauerbrei, 2004) and the associated functions available in Stata (MVRS and FP2) will be used. If there is evidence of important nonlinearities in the biomarker-treatment interactions, the risk score derived from this more complex model will be evaluated and compared to the linear model described in in 11.2.2. Even if there is no effect modification of the atrial cardiopathy variables on the efficacy of apixaban versus aspirin, we will evaluate these markers as predictors of stroke risk in the full sample of participants in whom we have measured these markers and for whom we have follow-up for stroke events. We will construct a prediction risk score which will also include the other prognostic variables defined in Section 9.5. Such a risk score will potentially be valuable for identifying subgroups of patients with atrial cardiopathy in whom the absolute benefit of apixaban is high enough to justify a primary prevention trial.

10.2.4 STATISTICAL MODEL

The following is the right hand side of the full Cox proportional hazards model:

$$= \beta_0 + \beta_1 I_{Tx} + \beta_2 X_{ECG} + \beta_3 X_{Echo} + \beta_4 X_{BNP} + \beta_5 I_{Tx} * X_{ECG} + \beta_6 I_{Tx} * X_{Echo} + \beta_7 I_{Tx} * X_{BNP} + \left[\sum \beta_j X_j \right]$$

Where I_{TX} is an indicator with the value of 1 if the participant is on apixaban or 0 for aspirin, $X_{ECG, ECHO, BNP}$ are the continuous values of the three biomarkers, and X_j are the values of other prognostic variables.

From the full model, standard backward elimination regression modeling procedures will be used to identify the final model. Specifically, we will first test the interaction term:

H_0: $\beta_5 = 0$; H_0: $\beta_6 = 0$; H_0: $\beta_7 = 0$

If it is not statistically significant at $\alpha = 0.016$, these terms will be dropped from the model and the covariates β_j will be tested in a stepwise approach. For the risk-based modeling, the fitted estimates of the final model will be reported given a participant's covariates, e.g. $\{X_{ECG}, X_{Echo}, X_{BNP}, X_j\}$, under apixaban and under aspirin, as well as the hazard ratio.

10.3 SECONDARY EFFICACY ANALYSIS

10.3.1 PARAMETER DESCRIPTION

The secondary efficacy outcomes are: (A) composite of recurrent ischemic stroke or systemic embolism, and (B) composite of recurrent stroke of any type or death from any cause. Each of these two outcomes will be analyzed separately using the ITT sample (Section 8.2).

10.3.2 STATISTICAL HYPOTHESES

The set of statistical hypotheses can be stated in terms of hazard functions as:

H_0: $h_{apix}(t)=h_{asp}(t)$ versus H_A: $h_{apix}(t) \neq n_{asp}(t)$

Where $h_{apix}(t)$ and $h_{asp}(t)$ are the hazard rates for the apixaban and aspirin groups, respectively.

10.3.3 STATISTICAL MODEL

Each of the secondary efficacy outcomes will be analyzed using the same model as described for the primary efficacy analysis (Section 10.1), but the status and time of events will be defined as follows:

Outcome: Composite of recurrent ischemic stroke or systemic embolism		
First Event	Status	Time
Recurrent ischemic stroke	1	Time of first ischemic stroke
Recurrent stroke of any type	0	Time of first stroke of any type
Systemic embolism	1	Time of first systemic embolism
Death	0	Time of death
Diagnosis of AF	N/A (continue to follow)	
Study drug stopped	N/A (continue to follow)	
Loss to follow-up or consent withdrawn	0	Last known contact

Outcome: Composite of recurrent stroke of any type or death from any cause		
First Event	Status	Time
Recurrent stroke of any type	1	Time of first stroke of any type
Systemic embolism	N/A (continue to follow)	
Death	1	Time of death
Diagnosis of AF	N/A (continue to follow)	
Study drug stopped	N/A (continue to follow)	
Loss to follow-up or consent withdrawn	0	Last known contact

10.3.4 EXPLORATORY EFFICACY ANALYSES

In a tertiary analysis, we will assess the effect of apixaban on the subset of ischemic stroke comprising only cardioembolic or cryptogenic stroke, as determined by Causative Classification of Stroke determinations performed by site investigators.

11 SAFETY ANALYSES

11.1 SAFETY MONITORING

The MSM and DSMB will receive periodic safety reports of all SAEs. The detailed guidelines for monitoring for safety by the MSM, and the DSMB will be provided in a separate ARCADIA Safety Monitoring Plan.

11.2 DESCRIPTIVE SUMMARY OF EVENTS

All clinical safety endpoints and SAEs will be summarized by AE code (as provided on the AE CRF) in terms of frequency of the event, number of participants having the event, severity, and relatedness to the study treatment. Clinically important adverse events include:

- Symptomatic intracranial hemorrhage (including symptomatic hemorrhagic transformation of an ischemic stroke).
- Major hemorrhage other than intracranial hemorrhage.
    - All-cause mortality.

The proportion of participants experiencing each of these events will be provided in the closed report to the DSMB by treatment arm with two-sided 95% CIs and unadjusted relative risks. Based on prior reports, the following approximate annual rates of major safety endpoints are expected:

Adverse Event	Apixaban	Aspirin
Symptomatic intracranial hemorrhage	1.5%	1.5%
Major hemorrhage other than intracranial hemorrhage	4.0%	3.0%
All-cause mortality	3.5%	3.5%

Log-rank tests will be utilized to assess the treatment group differences in the rates of: (1) first symptomatic intracranial hemorrhage; (2) first major hemorrhage other than intracranial hemorrhage; and (3) death from any cause.

11.3 ANALYSIS OF SAFETY OUTCOMES AT TRIAL COMPLETION

All safety outcome measurements are analyzed using the safety sample (Section 8.3). Sensitivity analyses will be conducted using the ITT population. At the end of the study, the cumulative incidences of the pre-specified safety events will be compared between the two treatment groups at a two-sided alpha level of 0.05.

11.3.1 PRIMARY SAFETY ENDPOINTS

The primary safety outcomes are: (A) symptomatic intracranial hemorrhage (including symptomatic hemorrhagic transformation of an ischemic stroke), and (B) major hemorrhage other than intracranial hemorrhage.

11.3.2 SECONDARY SAFETY ENDPOINT

The secondary safety endpoint is all-cause mortality.

11.3.3 EXPLORATORY SAFETY ENDPOINTS

The following exploratory safety endpoints will also be recorded: AF, any intracranial hemorrhage, major hemorrhage including any intracranial hemorrhage, symptomatic hemorrhagic transformation of an ischemic stroke, transient ischemic attack, myocardial infarction, minor hemorrhage, systemic embolism, symptomatic deep venous thrombosis, symptomatic pulmonary embolism, ischemic vascular death, and hemorrhagic vascular death. The endpoint of any intracranial hemorrhage will be subclassified as: 1) symptomatic versus asymptomatic, and 2) consisting of hemorrhagic transformation of the index brain infarct versus not.

11.4 ADJUDICATION OF SAFETY ENDPOINTS

Although regulatory reporting of possible SAEs will be based on the MSM determination of the event, for the publication of the final trial results analyses will use events as determined by the outcomes adjudication committee or site investigator (in the case of endpoints not adjudicated).

12 SUBGROUP ANALYSES BY SEX/GENDER AND RACE/ETHNICITY

In prespecified subgroup analyses, the analyses in Sections 10.1 (primary efficacy analyses) and 11 (safety analyses) will be stratified by sex/gender and race/ethnicity. Differential effects of apixaban versus aspirin by sex/gender or race/ethnicity are not anticipated and therefore the trial is not powered specifically for these subgroup analyses, but these subgroup analyses will allow any unexpected large variations to come to light.

13 QUALITY-OF-LIFE ANALYSES

13.1 QUALITY-OF-LIFE MEASURES

Participants' quality of life will be assessed at the Day 540 visit (18 months). Study investigators will elicit quality-of-life data from participants or their proxies using modules from PROMIS, a validated tool for assessing quality-of-life in patients with disorders including stroke. The Global Health Short Form v1.1 and the Physical Function Short Form v1.2 will be used. Missing data for quality-of-life measures will not be imputed.

13.2 ANALYSES OF QUALITY-OF-LIFE MEASURES

The scores from each PROMIS domain will be compared between treatment groups using generalized linear mixed models which can account for correlation between the physical and global health forms.

14 COST-EFFECTIVENESS ANALYSIS

The cost-effectiveness of treatment with apixaban as compared with aspirin in patients with cryptogenic stroke and atrial cardiopathy will be assessed using previously published methods (Kamel, *Neurology*, 2012). In brief, a Markov decision model will be created based on the wholesale cost of apixaban and data from the ARCADIA trial. The model will quantify the cost and quality-adjusted life expectancy resulting from apixaban therapy compared with aspirin. The base case population will be a patient cohort with the mean age of the ARCADIA cohort and a history of prior stroke. The base case will include the following health states: No disability (history of transient ischemic attack or ischemic stroke with full recovery), ischemic stroke (fatal or resulting in moderate-to-severe, mild, or no disability), intracranial hemorrhage (fatal or resulting in moderate-to-severe or mild disability), recurrent stroke or combined stroke and intracranial hemorrhage, extracranial hemorrhage, myocardial infarction, and death. Model inputs on the rates of these adverse events (e.g., recurrent ischemic stroke, intracranial hemorrhage, major hemorrhage, myocardial infarction) will be taken directly from the average annual rates of these events in the apixaban versus aspirin arms of the ARCADIA trial. Costs and quality-of-life estimates will be based on previously published data as well as quality-of-life data from ARCADIA (see Section 13

above). The model will adopt a societal perspective and will exclude costs unrelated to the treatment strategy in question (apixaban versus aspirin) or other disease states besides those listed above. Quality-adjusted life expectancy and net costs will be quantified over 20 years or until death, whichever occurs first. Life-years and costs will be discounted at 3% per year and reported as quality-adjusted life-years (QALYs) and 2023 U.S. dollars (rounded to the nearest $100). TreeAge Pro software (TreeAge Software, Williamstown, Massachusetts) will be used to build the model and perform all analyses.

15 UPDATE OF TRIAL OPERATING PARAMETERS

As a trial with time-to-event outcome, the power can be affected by aspects other than the observed event rate (i.e. recruitment rate leading to decreased follow-up time for participants). At the recommendation of the DSMB, trial design modifications to rectify this problem may be considered, namely:

- Increased follow-up period.
- Increased sample size (i.e. observed accrual rate and increased accrual duration).
- Increased number of sites.

These estimates will be made using the assumptions above (Section 7), namely:

- 7% annual risk of recurrent stroke of any type in aspirin-treated participants.
- Clinically relevant relative risk reduction of 40% (hazard ratio of 0.6, hazard rate of 4.2%) for participants treated with apixaban.
- 3% annual rate of crossover from blinded aspirin to open-label anticoagulation due to AF detection.
- 3% annual rate of crossover from blinded apixaban to open-label antiplatelet therapy due to bleeding or other adverse events.
- 4% annual rate of loss to follow-up or death in each group.

16 CHANGE LOG

Date	Relation to interim or other analysis	Issue	Trigger	Solution
3 Jan 2018	Changes preceded start of recruitment	Elimination of plan for sample size re-estimation	Not logistically feasible	No sample size re-estimation
3 Jan 2018	Changes preceded start of recruitment	Elimination of plan for revision to group sequential boundaries	Inherent difficulty in accurately estimating	No plan for revision of group sequential boundaries
3 Jan 2018	Changes preceded start of recruitment	Randomization method	Balancing variables felt to be arbitrary	Randomization will only control the maximum tolerated

				imbalance within each RCC	

17 REFERENCES

1. Longstreth WT, Jr., Kronmal RA, Thompson JL, et al. Amino terminal pro-B-type natriuretic peptide, secondary stroke prevention, and choice of antithrombotic therapy. *Stroke.* 2013;44(3):714-719.
2. Connolly SJ, Eikelboom J, Joyner C, et al. Apixaban in patients with atrial fibrillation. *N Engl J Med.* 2011;364(9):806-817.
3. Ruff CT, Giugliano RP, Braunwald E, et al. Comparison of the efficacy and safety of new oral anticoagulants with warfarin in patients with atrial fibrillation: a meta-analysis of randomised trials. *Lancet.* 2014;383(9921):955-962.
4. Granger CB, Alexander JH, McMurray JJ, et al. Apixaban versus warfarin in patients with atrial fibrillation. *N Engl J Med.* 2011;365(11):981-992.
5. Kernan WN, Ovbiagele B, Black HR, et al. Guidelines for the prevention of stroke in patients with stroke and transient ischemic attack: a guideline for healthcare professionals from the American Heart Association/American Stroke Association. *Stroke.* 2014;45(7):2160-2236.
6. Little RJ, D'Agostino R, Cohen ML, et al. The prevention and treatment of missing data in clinical trials. *N Engl J Med.* 2012;367(14):1355-1360.
