# Supplementary material for: Hypertension With High-Risk Features in Cryptogenic Stroke: An Exploratory Analysis of the ARCADIA Randomized Clinical Trial
Source: JAMA Neurol. 2026 Apr 20;83(6):573–81. doi: 10.1001/jamaneurol.2026.0855 (PMC13097029; doi:10.1001/jamaneurol.2026.0855)
Supplement: Supplement 2. — eTable 1. Hypertension With High-Risk Features Definitions eTable 2. Baseline Characteristics Between Treatment Arms Stratified by Hypertension With High-Risk Features eTable 3. Systolic Blood Pressure by Hypertension-Mediated Organ Damage Definitions eTable 4. Crude and Alternatively Adjusted Interaction Models of Recurrent Ischemic Stroke or Systemic Embolism eTable 5. Fully Adjusted Interaction Models of Secondary Outcomes eTable 6. Interaction Models Using Alternative Systolic Blood Pressure Thresholds of Hypertension With High-Risk Features eTable 7. Additional Sensitivity Analyses eFigure 1. Cumulative Event Rates for Recurrent Ischemic Stroke In Patients With and Without High-Risk Features of Hypertension eFigure 2. Cumulative Event Rates for Stroke of Any Type In Patients With and Without High-Risk Features of Hypertension [file jamaneurol-e260855-s002.pdf]

Supplemental Online Content

Ridha M, Raed Hailat R, Stanton R, et al. Hypertension with high-risk features in cryptogenic stroke: an exploratory analysis of the ARCADIA randomized clinical trial. *JAMA Neurol*. Published online April 20, 2026. doi:10.1001/jamaneurol.2026.0855

eTable 1. Hypertension With High-Risk Features Definitions

eTable 2. Baseline Characteristics Between Treatment Arms Stratified by Hypertension With High-Risk Features

eTable 3. Systolic Blood Pressure by Hypertension-Mediated Organ Damage Definitions

eTable 4. Crude and Alternatively Adjusted Interaction Models of Recurrent Ischemic Stroke or Systemic Embolism

eTable 5. Fully Adjusted Interaction Models of Secondary Outcomes

eTable 6. Interaction Models Using Alternative Systolic Blood Pressure Thresholds of Hypertension With High-Risk Features

eTable 7. Additional Sensitivity Analyses

eFigure 1. Cumulative Event Rates for Recurrent Ischemic Stroke In Patients With and Without High-Risk Features of Hypertension

eFigure 2. Cumulative Event Rates for Stroke of Any Type In Patients With and Without High-Risk Features of Hypertension

This supplemental material has been provided by the authors to give readers additional information about their work.

© 2026 Ridha M et al. *JAMA Neurology*.

eTable 1. Hypertension With High-Risk Features Definitions

Hypertension With High-Risk Features Definition [a]	Systolic Blood Pressure Criteria	Hypertension Mediated Organ Damage Criteria
Primary definition	SBP ≥160 mmHg	*Cardiac HMOD:* LVH [b] on echocardiography
Secondary definition 1	SBP ≥160 mmHg	*Renal HMOD:* eGFR [c] <60 mL/min/1.73 m^2
Secondary definition 2	SBP ≥160 mmHg	*Any HMOD:* LVH and/or eGFR <60 mL/min/1.73 m^2

Abbreviations: eGFR, estimated glomerular filtration rate; HMOD, hypertension mediated organ damage; SBP, systolic blood pressure; LVH, left ventricular hypertrophy

[a] Presence of either systolic blood pressure criteria or hypertension mediated organ damage criteria meets definition of hypertension with high-risk features.

[b] LVH defined by sex-specific left ventricular mass index cutoffs (men: >115 g/m^2; women: >95 g/m^2)

[c] eGFR calculated using the 2021 CKD-EPI (race-free) formula

© 2026 Ridha M et al. *JAMA Neurology*.

eTable 2. Baseline Characteristics Between Treatment Arms Stratified by Hypertension With High-Risk Features

**Characteristic, N (%)**	**No hypertension with high-risk features** [a]			**Hypertension with high-risk features** [a]		
Treatment Assignment	Aspirin (n=296)	Apixaban (n=298)	P-value	Aspirin (n=184)	Apixaban (n=167)	P-value
Age, mean (SD), y	68.7 (10.8)	68.0 (10.7)	0.41	67.1 (11.2)	67.8 (10.7)	0.55
Sex						
Female	155 (52.4)	152 (51.0)	0.74	107 (58.2)	99 (59.3)	0.83
Male	141 (47.6)	146 (49.0)		77 (41.8)	68 (40.7)	
Race						
Black	47 (15.9)	48 (16.1)	0.98	55 (29.9)	51 (30.5)	0.61
White	237 (80.1)	236 (79.2)		119 (64.7)	111 (66.5)	
Other	10 (3.4)	12 (4.0)		4 (2.2)	1 (0.6)	
Unknown	2 (0.7)	2 (0.7)		6 (3.3)	4 (2.4)	
Ethnicity						
Hispanic	24 (8.1)	24 (8.1)	0.84	15 (8.2)	17 (10.2)	0.80
Non-Hispanic	270 (91.2)	273 (91.6)		168 (91.3)	149 (89.2)	
Unknown	2 (0.7)	1 (0.3)		1 (0.5)	1 (0.6)	
Medical Comorbidities						
Coronary artery disease	26 (8.9)	28 (9.4)	0.84	17 (9.3)	25 (15.1)	0.10
Heart failure	12 (4.1)	9 (3.1)	0.52	21 (11.4)	21 (13.8)	0.52
Diabetes	87 (29.6)	83 (27.9)	0.64	65 (35.3)	58 (34.9)	0.94
Hypertension	215 (72.9)	216 (72.7)	0.97	153 (83.2)	145 (87.3)	0.27
Peripheral vascular disease	4 (1.4)	4 (1.4)	1.00	3 (1.6)	5 (3.0)	0.48
Obstructive sleep apnea	32 (11.7)	45 (15.8)	0.16	23 (13.0)	24 (15.4)	0.53
Prior stroke or TIA	56 (19.0)	45 (15.1)	0.21	37 (20.2)	37 (22.2)	0.66
Cancer	48 (16.2)	39 (13.2)	0.30	23 (12.5)	18 (10.8)	0.63
Current tobacco use	40 (13.5)	50 (16.8)	0.27	41 (22.3)	28.16.8)	0.19
BMI, mean (SD), kg/m²	29.1 (6.2)	29.8 (7.0)	0.23	30.2 (7.0)	30.6 (6.8)	0.59
CHA_2DS_2VASc score, mean (SD)	3.0 (1.6)	2.9 (1.5)	0.16	3.3 (1.5)	3.4 (1.7)	0.28
NIHSS, mean (SD)	1.9 (2.9)	2.5 (3.9)	0.07	2.1 (3.1)	2.3 (3.6)	0.65
Days from qualifying stroke to enrollment, mean (SD)	41.5 (45.1)	38.3 (45.2)	0.39	36.7 (44.1)	33.4 (40.2)	0.46
Days from BP measurement to enrollment, mean (SD)	1.9 (13.7)	2.3 (14.0)	0.72	6.1 (23.5)	2.1 (14.7)	0.05
SBP, mean (SD), mm Hg	131.9 (14.7)	131.5 (16.3)	0.73	149.7 (23.9)	149.9 (23.6)	0.96
DBP, mean (SD), mm Hg	76.0 (11.9)	75.3 (11.6)	0.48	81.8 (14.6)	79.2 (15.4)	0.11
eGFR, mean (SD), mL/min/1.73 m²	78.8 (18.8)	78.2 (19.1)	0.67	74.8 (20.5)	72.9 (22.2)	0.41
Atrial cardiopathy biomarkers						
PTFV1, mean (SD), µV×ms	4635.5 (2630.2)	4866.6 (2384.4)	0.27	5021.3 (3303.7)	4571.5 (2715.6)	0.17
NT-proBNP, median (IQR), pg/mL	283.9 (98.7-527.9)	251.1 (60.9-404.9)	0.002	392.1 (218.8-685.2)	433.3 (175.3-896.7)	0.36

© 2026 Ridha M et al. *JAMA Neurology*.

LAD index, mean (SD), cm/m2	1.8 (0.5)	1.9 (0.4)	0.91	2.0 (0.4)	2.1 (0.5)	0.26
Type of echocardiogram						
Transthoracic	259 (87.5)	261 (87.6)	0.98	160 (87.0)	148 (88.6)	0.39
Transesophageal	0.0 (0.0)	0.0 (0.0)		2 (1.1)	0.0 (0.0)	
Both	37 (12.5)	36 (12.4)		22 (12.0)	19 (11.4)	
LV wall measurements						
Posterior wall thickness, mean (SD), cm	1.0 (0.2)	1.0 (0.2)	0.14	1.2 (0.3)	1.2 (0.3)	0.08
Septal thickness, mean (SD), cm	1.0 (0.2)	1.0 (0.2)	0.14	1.2 (0.3)	1.3 (0.3)	0.26
End diastolic diameter, mean (SD), cm	4.5 (0.6)	4.5 (0.6)	0.64	4.7 (0.7)	4.8 (0.7)	0.71
LV mass, mean (SD), g	150.4 (41.2)	154.0 (43.0)	0.30	224.4 (77.3)	236.0 (71.5)	0.15
LV mass index, mean (SD), g/m²	76.4 (15.8)	78.3 (17.6)	0.19	114.9 (34.3)	122.4 (35.7)	0.05
Relative wall thickness, mean (SD)	0.43 (0.12)	0.45 (0.14)	0.18	0.52 (0.15)	0.54 (0.16)	0.29
Ejection fraction, mean (SD), %	60.8 (6.5)	61.5 (6.5)	0.20	58.7 (9.8)	59.1 (8.5)	0.70
Infarct pattern						
Cortical	197 (66.6)	189 (63.4)	0.42	127 (69.0)	99 (59.3)	0.06
Cerebellar	34 (11.5)	35 (11.7)	0.92	23 (12.5)	21 (12.6)	0.98
Deep, small	67 (22.6)	61 (20.5)	0.52	42 (22.8)	41 (24.6)	0.70
Deep, large	30 (10.1)	35 (11.7)	0.53	21 (11.4)	22 (13.2)	0.62
Multiple	55 (18.6)	44 (14.8)	0.21	38 (20.7)	32 (19.2)	0.73
Atrial fibrillation detected on follow up	42 (14.2)	35 (11.7)	0.38	29 (15.8)	23 (13.8)	0.60
Per-protocol cohort	280 (94.6)	280 (94.0)	0.74	176 (95.7)	157 (94.0)	0.49
Follow up duration, mean (SD), days	628.2 (481.1)	682.8 (491.9)	0.17	687.3 (489.3)	626.4 (483.1)	0.24

Abbreviations: eGFR, estimated glomerular filtration rate; TIA, transient ischemic attack; BMI, body mass index; NIHSS, National Institute of Health Stroke Scale; SBP, systolic blood pressure; DBP, diastolic blood pressure; LAD, left atrial diameter; LV, left ventricle; LVH, left ventricular hypertrophy; NT-proBNP, N-terminal pro-B-type natriuretic peptide; $PTFV_1$, P-wave terminal force in lead V_1

[a] Hypertension with high-risk features defined as systolic blood pressure ≥160 mmHg and/or left ventricular hypertrophy

© 2026 Ridha M et al. *JAMA Neurology*.

eTable 3. Systolic Blood Pressure by Hypertension-Mediated Organ Damage Definitions

Hypertension Mediated Organ Damage Definition	Systolic Blood Pressure, mean (SD), mmHg	P-value	Systolic Blood Pressure ≥160 mmHg, n (%)	P-value
LVH [a]				
Present (n=263)	142.3 (22.0)	<0.001	53 (20.2)	0.01
Absent (n=682)	136.9 (20.2)		88 (12.9)	
eGFR [b] *<60 ml/min/1.73 m^2*				
Present (n=199)	140.7 (23.0)	0.12	41 (20.6)	0.01
Absent (n=745)	137.8 (20.3)		100 (13.4)	
Any HMOD (LVH or eGFR <60 ml/min/1.73 m^2)				
Present (n=386)	140.9 (21.9)	0.003	71 (18.4)	0.01
Absent (n=558)	136.7 (20.0)		70 (12.5)	

Abbreviations: eGFR, estimated glomerular filtration rate; HMOD, hypertension mediated organ damage; LVH, left ventricular hypertrophy

[a] Left ventricular hypertrophy defined by sex-specific left ventricular mass index cutoffs (men: >115 g/m^2; women: >95 g/m^2)

[b] Estimated glomerular filtration rate calculated using the 2021 CKD-EPI (race-free) formula

© 2026 Ridha M et al. *JAMA Neurology*.

eTable 4. Crude and Alternatively Adjusted Interaction Models of Recurrent Ischemic Stroke or Systemic Embolism

**Recurrent Ischemic Stroke or Systemic Embolism**	**HR (95% CI)**	**P-value**
Crude model [a]		
Apixaban versus aspirin	0.40 (0.21-0.79)	0.008
Hypertension with high-risk features	0.60 (0.30-1.20)	0.15
Apixaban* Hypertension with high-risk features	4.24 (1.52-11.79)	0.006
Alternative covariate adjustment [a,b]		
Apixaban versus aspirin	0.43 (0.22-0.86)	0.02
Hypertension with high-risk features	0.52 (0.26-1.06)	0.07
Apixaban* Hypertension with high-risk features	4.18 (1.50-11.65)	0.006

[a] Hypertension with high-risk features defined as systolic blood pressure ≥160 mmHg and/or left ventricular hypertrophy

[b] Adjusted for history of diabetes, prior stroke or transient ischemic attack, and Black race

© 2026 Ridha M et al. *JAMA Neurology*.

eTable 5. Fully Adjusted Interaction Models of Secondary Outcomes

Models	HR (95% CI)	P-value
Recurrent Ischemic Stroke [a]		
Apixaban versus aspirin	0.47 (0.24-0.93)	0.03
Hypertension with high-risk features	0.49 (0.23-1.02)	0.06
Apixaban* Hypertension with high-risk features	3.97 (1.39-11.33)	0.01
Any Stroke [a]		
Apixaban versus aspirin	0.53 (0.28-1.00)	0.05
Hypertension with high-risk features	0.58 (0.29-1.14)	0.12
Apixaban* Hypertension with high-risk features	3.15 (1.16-8.53)	0.02

[a] Hypertension with high-risk features defined as systolic blood pressure ≥160 mmHg and/or left ventricular hypertrophy

© 2026 Ridha M et al. *JAMA Neurology*.

eTable 6. Interaction Models Using Alternative Systolic Blood Pressure Thresholds of Hypertension With High-Risk Features

**Models [a]**	**HR (95% CI)**	**P-value**
High-risk features: SBP ≥140mm Hg and/or LVH		
Apixaban versus aspirin	0.79 (0.33-1.88)	0.60
High-risk hypertension	1.30 (0.66-2.59)	0.45
Apixaban*High-risk hypertension	0.96 (0.34-2.72)	0.93
High-risk features: SBP ≥150mm Hg and/or LVH		
Apixaban versus aspirin	0.49 (0.23-1.02)	0.06
High-risk hypertension	0.71 (0.37-1.34)	0.29
Apixaban*High-risk hypertension	2.38 (0.87-6.50)	0.09
High-risk features: SBP ≥160mm Hg and/or LVH		
Apixaban versus aspirin	0.43 (0.22-0.85)	0.02
High-risk hypertension	0.51 (0.25-1.03)	0.06
Apixaban*High-risk hypertension	3.87 (1.39-10.82)	0.01

Abbreviations: LVH, left ventricular hypertrophy; SBP, systolic blood pressure
[a] All models fully adjusted for CHA_2DS_2VASc score and Black race

© 2026 Ridha M et al. *JAMA Neurology*.

eTable 7. Additional Sensitivity Analyses

Model	HR (95% CI)	P-value
Hypertension with high-risk features definition: any HMOD [a,b]		
Apixaban versus aspirin	0.46 (0.23-0.94)	0.03
HMOD	0.63 (0.33-1.24)	0.18
Apixaban*HMOD	2.88 (1.06-7.86)	0.04
*Hypertension with high-risk features definition: SBP r*ange [c, d]		
Apixaban versus aspirin	0.63 (0.34-1.19)	0.15
SBP range	0.88 (0.65-1.18)	0.38
Apixaban*SBP range	1.23 (0.80-1.90)	0.35
Excluding small, deep infarcts [b,e]		
Apixaban versus aspirin	0.42 (0.21-0.85)	0.02
Hypertension with high-risk features	0.49 (0.24-1.01)	0.05
Apixaban*Hypertension with high-risk features	4.08 (1.43-11.61)	0.008
Per-protocol analysis [b,e]		
Apixaban versus aspirin	0.45 (0.23-0.89)	0.02
Hypertension with high-risk features	0.50 (0.25-1.03)	0.06
Apixaban*Hypertension with high-risk features	2.99 (1.04-8.61)	0.04

Abbreviations: HMOD, hypertension mediated organ damage; SBP: systolic blood pressure

[a] HMOD defined as left ventricular hypertrophy or estimated glomerular filtrate rate <60 ml/min/1.73 m^2

[b] Adjusted for CHA_2DS_2VASc score and Black race

[c] Adjusted for CHA_2DS_2VASc score, Black race, and days between qualifying stroke and blood pressure measurement

[d] Systolic blood pressure ranges defined as <140mmgh, 140-149mmHg, 150-159mmHg, and ≥160 mmHg

[e] Hypertension with high-risk features defined as systolic blood pressure ≥160 mmHg and/or left ventricular hypertrophy

© 2026 Ridha M et al. *JAMA Neurology*.

eFigure 1. Cumulative Event Rates for Recurrent Ischemic Stroke In Patients With and Without High-Risk Features of Hypertension

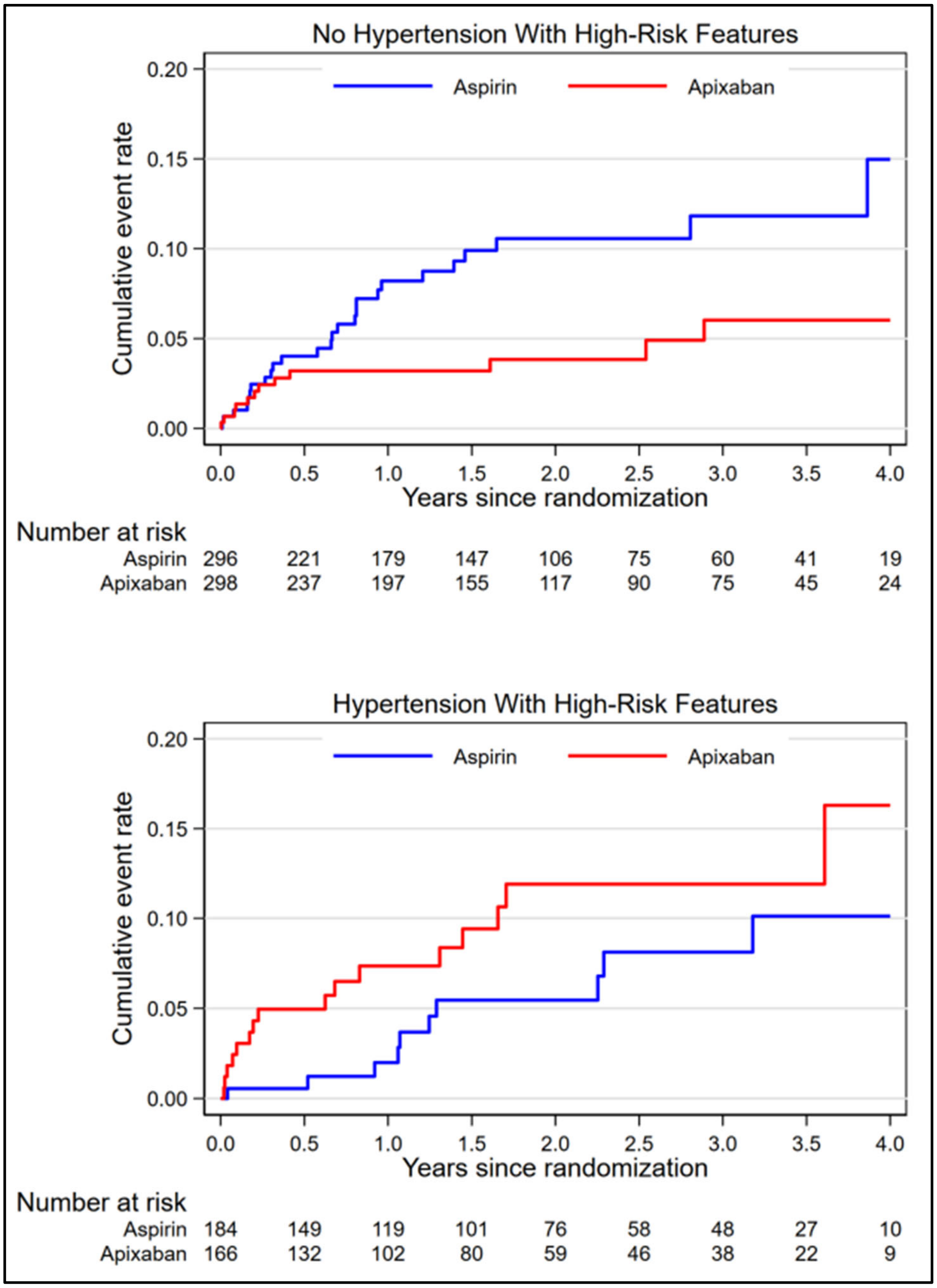

Top: Cumulative event rate curves for patients without high-risk features of hypertension.
Bottom: Cumulative event rate curves for patients with high-risk features of hypertension.

© 2026 Ridha M et al. *JAMA Neurology*.

eFigure 2. Cumulative Event Rates for Stroke of Any Type In Patients With and Without High-Risk Features of Hypertension

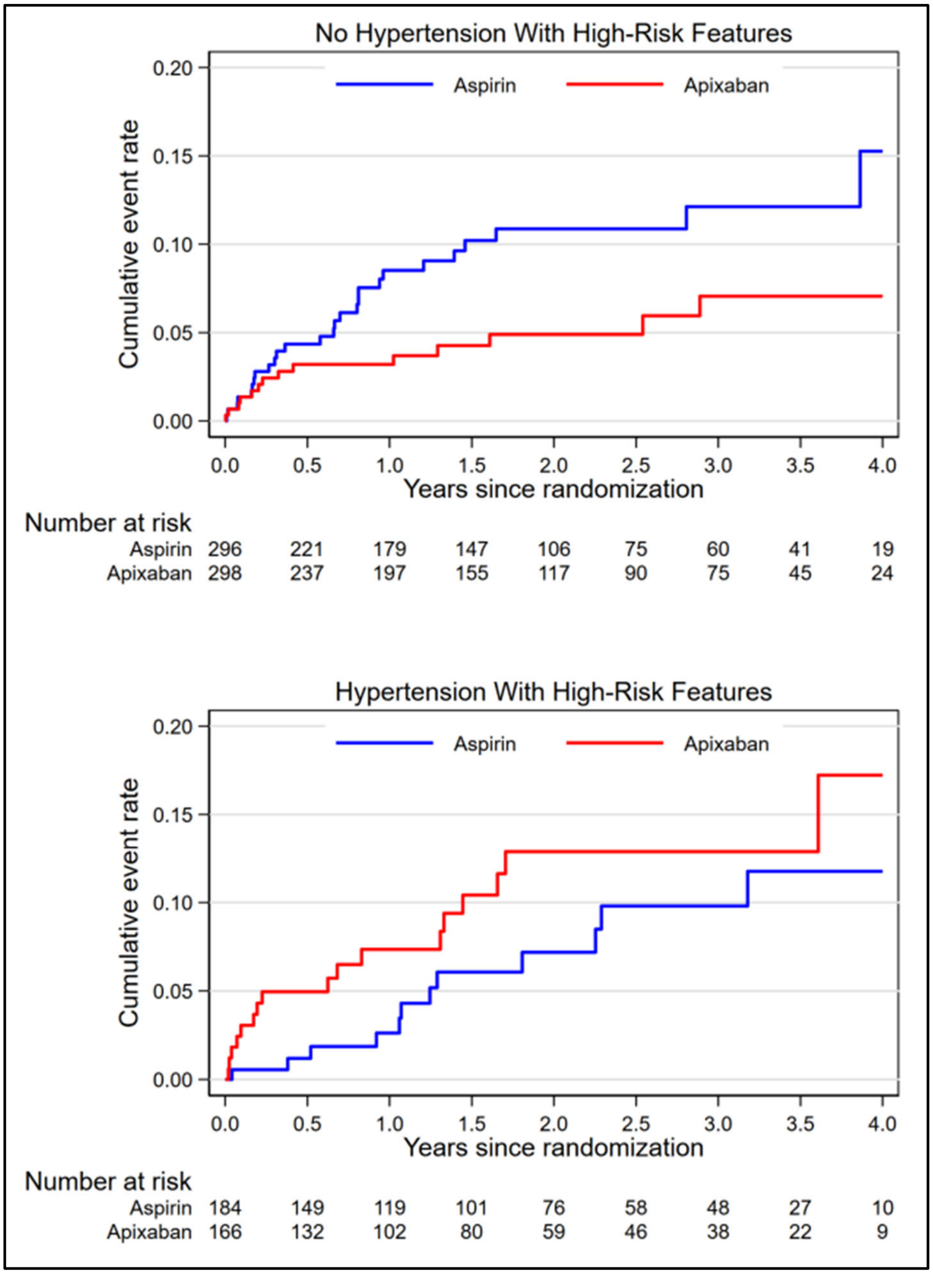

Top: Cumulative event rate curves for patients without high-risk features of hypertension.
Bottom: Cumulative event rate curves for patients with high-risk features of hypertension.

© 2026 Ridha M et al. *JAMA Neurology*.
