# Supplementary material for: Hypertension With High-Risk Features in Cryptogenic Stroke: An Exploratory Analysis of the ARCADIA Randomized Clinical Trial
Source: JAMA Neurol. 2026 Apr 20;83(6):573–81. doi: 10.1001/jamaneurol.2026.0855 (PMC13097029; doi:10.1001/jamaneurol.2026.0855)
Supplement: Supplement 3. — Data sharing statement [file jamaneurol-e260855-s003.pdf]

Data Sharing Statement

Ridha. Hypertension With High-Risk Features in Cryptogenic Stroke. *JAMA Neurol*. Published April 20, 2026. doi:10.1001/jamaneurol.2026.0855

Data
Additional Information: ClinicalTrials.gov Identifier NCT03192215
Data available: Yes
Data types: Deidentified participant data, Data dictionary
How to access data: Data for this study was received from the National Institute of Neurologic Disease and Stroke Archived Clinical Research Dataset. Access to the ARCADIA trial data is available upon request through the Archived Clinical Research Dataset webpage.
When available: With publication

Supporting Documents
Document types: None

Additional Information
Who can access the data: Researchers whose proposed use of the data has been approved by the National Institute of Neurologic Disease and Stroke
Types of analyses: For purposes approved by the National Institute of Neurologic Disease and Stroke
Mechanisms of data availability: After approval of proposal at the discretion of the National Institute of Neurologic Disease and Stroke
